# Revisions to the Classification, Nomenclature, and Diversity of Eukaryotes

**DOI:** 10.1111/jeu.12691

**Published:** 2019-01-19

**Authors:** Sina M. Adl, David Bass, Christopher E. Lane, Julius Lukeš, Conrad L. Schoch, Alexey Smirnov, Sabine Agatha, Cedric Berney, Matthew W. Brown, Fabien Burki, Paco Cárdenas, Ivan Čepička, Lyudmila Chistyakova, Javier del Campo, Micah Dunthorn, Bente Edvardsen, Yana Eglit, Laure Guillou, Vladimír Hampl, Aaron A. Heiss, Mona Hoppenrath, Timothy Y. James, Anna Karnkowska, Sergey Karpov, Eunsoo Kim, Martin Kolisko, Alexander Kudryavtsev, Daniel J.G. Lahr, Enrique Lara, Line Le Gall, Denis H. Lynn, David G. Mann, Ramon Massana, Edward A.D. Mitchell, Christine Morrow, Jong Soo Park, Jan W. Pawlowski, Martha J. Powell, Daniel J. Richter, Sonja Rueckert, Lora Shadwick, Satoshi Shimano, Frederick W. Spiegel, Guifré Torruella, Noha Youssef, Vasily Zlatogursky, Qianqian Zhang

**Affiliations:** ^1^ Department of Soil Sciences College of Agriculture and Bioresources, University of Saskatchewan Saskatoon S7N 5A8 SK Canada; ^2^ Department of Life Sciences The Natural History Museum Cromwell Road London SW7 5BD United Kingdom; ^3^ Centre for Environment, Fisheries and Aquaculture Science (CEFAS) Barrack Road, The Nothe Weymouth Dorset DT4 8UB United Kingdom; ^4^ Department of Biological Sciences University of Rhode Island Kingston Rhode Island 02881 USA; ^5^ Institute of Parasitology, Biology Centre Czech Academy of Sciences České Budějovice 37005 Czechia; ^6^ Faculty of Science University of South Bohemia České Budějovice 37005 Czechia; ^7^ National Institute for Biotechnology Information, National Library of Medicine, National Institutes of Health Bethesda Maryland 20892 USA; ^8^ Department of Invertebrate Zoology Faculty of Biology Saint Petersburg State University Saint Petersburg 199034 Russia; ^9^ Department of Biosciences University of Salzburg Hellbrunnerstrasse 34 Salzburg A‐5020 Austria; ^10^ CNRS, UMR 7144 (AD2M), Groupe Evolution des Protistes et Ecosystèmes Pélagiques Station Biologique de Roscoff Place Georges Teissier Roscoff 29680 France; ^11^ Department of Biological Sciences Mississippi State University Starkville 39762 Mississippi USA; ^12^ Institute for Genomics, Biocomputing & Biotechnology Mississippi State University Starkville 39762 Mississippi USA; ^13^ Department of Organismal Biology Program in Systematic Biology Science for Life Laboratory Uppsala University Uppsala 75236 Sweden; ^14^ Pharmacognosy, Department of Medicinal Chemistry Uppsala University BMC Box 574 Uppsala SE‐75123 Sweden; ^15^ Department of Zoology Faculty of Science Charles University Vinicna 7 Prague 128 44 Czechia; ^16^ Core Facility Centre for Culture Collection of Microorganisms Saint Petersburg State University Saint Petersburg 198504 Russia; ^17^ Institut de Ciències del Mar, CSIC Passeig Marítim de la Barceloneta, 37‐49 Barcelona 08003 Catalonia Spain; ^18^ Department of Ecology University of Kaiserslautern Erwin‐Schroedinger Street Kaiserslautern D‐67663 Germany; ^19^ Department of Eukaryotic Microbiology University of Duisburg‐Essen Universitätsstrasse 5 Essen D‐45141 Germany; ^20^ Department of Biosciences University of Oslo P.O. Box 1066 Blindern Oslo 0316 Norway; ^21^ Department of Biology Dalhousie University Halifax B3H 4R2 NS Canada; ^22^ Sorbonne Université, Université Pierre et Marie Curie ‐ Paris 6, CNRS, UMR 7144 (AD2M) Station Biologique de Roscoff Place Georges Teissier, CS90074 Roscoff 29688 France; ^23^ Department of Parasitology Faculty of Science Charles University, BIOCEV Průmyslová 595 Vestec 252 42 Czechia; ^24^ Department of Invertebrate Zoology American Museum of Natural History New York City New York 10024 USA; ^25^ Senckenberg am Meer, DZMB – German Centre for Marine Biodiversity Research Wilhelmshaven 26382 Germany; ^26^ Department of Ecology and Evolutionary Biology University of Michigan Ann Arbor Michigan 48109 USA; ^27^ Department of Molecular Phylogenetics and Evolution University of Warsaw Warsaw 02‐089 Poland; ^28^ Laboratory of Parasitic Worms and Protistology Zoological Institute RAS Saint Petersburg 199034 Russia; ^29^ Department of Zoology Institute of Biosciences University of Sao Paulo Matao Travessa 14 Cidade Universitaria Sao Paulo 05508‐090 Sao Paulo Brazil; ^30^ Laboratory of Soil Biodiversity University of Neuchâtel Rue Emile‐Argand 11 Neuchâtel 2000 Switzerland; ^31^ Real Jardín Botánico, CSIC Plaza de Murillo 2 Madrid 28014 Spain; ^32^ Institut de Systématique, Évolution, Biodiversité, Muséum National d'Histoire Naturelle Sorbonne Universités 57 rue Cuvier, CP 39 Paris 75005 France; ^33^ Department of Integrative Biology University of Guelph Summerlee Science Complex Guelph ON N1G 2W1 Canada; ^34^ Department of Zoology University of British Columbia 4200‐6270 University Blvd. Vancouver BC V6T 1Z4 Canada; ^35^ Royal Botanic Garden Edinburgh EH3 5LR United Kingdom; ^36^ Institute for Agrifood Research and Technology C/Poble Nou km 5.5 Sant Carles de La Ràpita E‐43540 Spain; ^37^ Jardin Botanique de Neuchâtel Chemin du Perthuis‐du‐Sault 58 Neuchâtel 2000 Switzerland; ^38^ Department of Natural Sciences National Museums Northern Ireland 153 Bangor Road Holywood BT18 OEU United Kingdom; ^39^ Department of Oceanography and Kyungpook Institute of Oceanography School of Earth System Sciences Kyungpook National University Daegu Korea; ^40^ Department of Genetics and Evolution University of Geneva 1211 Geneva 4 Switzerland; ^41^ Department of Biological Sciences The University of Alabama Tuscaloosa Alabama 35487 USA; ^42^ Institut de Biologia Evolutiva (CSIC‐Universitat Pompeu Fabra) Passeig Marítim de la Barceloneta 37‐49 Barcelona 08003 Catalonia Spain; ^43^ School of Applied Sciences Edinburgh Napier University Edinburgh EH11 4BN United Kingdom; ^44^ Department of Biological Sciences University of Arkansas Fayetteville Arkansas AR 72701 USA; ^45^ Science Research Centre Hosei University 2‐17‐1 Fujimi Chiyoda‐ku Tokyo 102‐8160 Japan; ^46^ Laboratoire Evolution et Systématique, Université Paris‐XI Orsay 91405 France; ^47^ Department of Microbiology and Molecular Genetics Oklahoma State University Stillwater Oklahoma 74074 USA; ^48^ Department of Organismal Biology Systematic Biology Program Uppsala University Uppsala SE‐752 36 Sweden; ^49^ Yantai Institute of Coastal Zone Research Chinese Academy of Science Yantai 264003 China

**Keywords:** Algae, amoebae, biodiversity, ciliate, ecology, flagellate, fungus, microbiology, parasite, plankton, protozoa, systematics, taxonomy

## Abstract

This revision of the classification of eukaryotes follows that of Adl et al., 2012 [*J. Euk. Microbiol*. 59(5)] and retains an emphasis on protists. Changes since have improved the resolution of many nodes in phylogenetic analyses. For some clades even families are being clearly resolved. As we had predicted, environmental sampling in the intervening years has massively increased the genetic information at hand. Consequently, we have discovered novel clades, exciting new genera and uncovered a massive species level diversity beyond the morphological species descriptions. Several clades known from environmental samples only have now found their home. Sampling soils, deeper marine waters and the deep sea will continue to fill us with surprises. The main changes in this revision are the confirmation that eukaryotes form at least two domains, the loss of monophyly in the Excavata, robust support for the Haptista and Cryptista. We provide suggested primer sets for DNA sequences from environmental samples that are effective for each clade. We have provided a guide to trophic functional guilds in an appendix, to facilitate the interpretation of environmental samples, and a standardized taxonomic guide for East Asian users.

THIS revision of the classification of eukaryotes updates that of the International Society of Protistologists (Adl et al. 2012). Since then, there has been a massive increase in DNA sequence information of phylogenetic relevance from environmental samples. We now have a much better sense of the undescribed biodiversity in our environment (De Vargas et al. 2015; Pawlowski et al. 2012). While significant, it still remains a partial estimation as several continents and soils in general are poorly sampled, and the deeper ocean is hard to reach. These new data clarified phylogenetic relationships and the new information is incorporated in this revision.

## Systematics

We assembled the classification according to the principles outlined elsewhere, and we refer the reader to the introductions of both Adl et al. 2005 and 2012 for background information, and to Adl et al. 2007 for a discussion. Briefly, we adopted a hierarchical system without formal rank designations. The hierarchy is represented by indented paragraphs. The nomenclatural priority is given to the oldest name (and its authority) that correctly assembled genera or higher groups together into a clade, except where its composition was substantially modified. In these cases, we have used a newer term and its appropriate authorship. In cases where ranks were created to include a single lower rank, the higher ranks were eliminated as superfluous. In this scheme, monotypic taxa are represented by the genus only. Nested clades represent monophyletic lineages as best we know and para‐ or polyphyletic groups are so indicated.

This system of hierarchical nameless ranks, that ignores endings of clade names, has proved its utility in providing name stability as we reconstructed a new phylogenetic classification during the past 20 years. Clade names in this system do not change when their rank or composition changes, and it is only the authority for the name that changes when each clade description is adjusted (Cantino, 1998 Pleijel and Rouse, 2003). Where a new term is introduced in this classification, it is identified with “Adl et al. 2019” as the authority, or by the submitting author (e.g. Mann in Adl et al., 2019), and they are to be cited as emended in this publication. The descriptions provided are not intended to substitute for formal diagnoses. They are provided primarily for the student and general users to identify common morphological features, such as synapomorphies and apomorphies, within monophyletic lineages.

**Figure 1:**
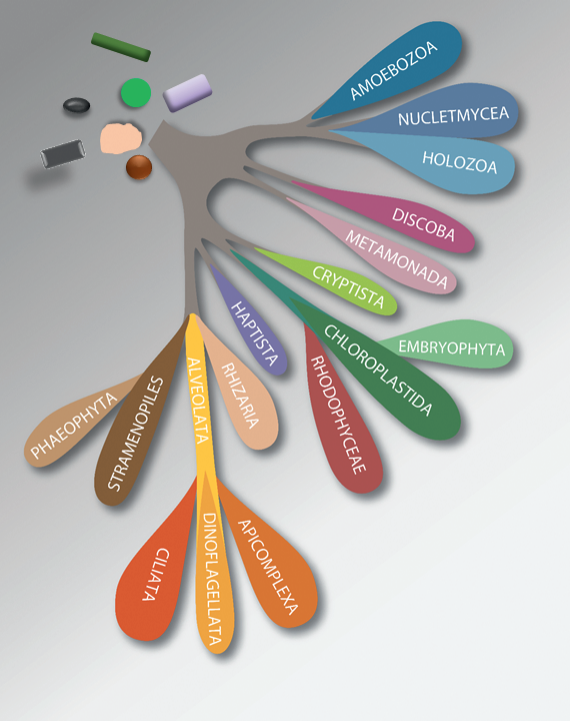
Overview of the diversity of protists among eukaryotes. Amoebozoa, Nucletmycea, and Holozoa together form the Amorphea; The Diaphoretickes includes Crypista, Chloroplastida and Embryophyta, Rhodophyceae, Haptista, Rhizaria, Alveolata (Apicomplexa, Dinoflagellata, Ciliata), Stramenopiles and Phaeophyta.

There are two novel components in this revision. First, we have provided trophic assignments for most taxa. This will prove useful in interpreting communities from environmental samples. Second, we informally suggest a phylum rank and classes in most clades to provide a point of reference in the classification hierarchy for the nonspecialist. This became possible, as there has been some stability at this level in the molecular phylogenetic reconstructions. It should be obvious that genera grouped into a clade then represent a family, and families into an order.

## Nomenclature

This committee of the Society has had the responsibility of arbitrating nomenclature for protists in general. Historically, the task was simpler as most groups fell under one or the other of the two Codes of Nomenclature (algae and some other protists under the “International Code of Nomenclature for algae, fungi, and plants”, and protozoa under the “International Code of Zoological Nomenclature”), and few were described under both Codes. The Society was represented on the relevant committees. Notwithstanding that both Codes are incompatible, some have proposed to provide parallel classifications in each Code, while others proposed to adopt a modern unified code of nomenclature. Since the rearrangement of the classification along monophyletic lineages during the 1990s, many clades now include a mixture of taxa from both Codes. Several taxa, such as diatoms, are described in parallel under both Codes with different names. This situation created and perpetuates anomalies, such as the recent redescription of the dictyostellid amoebae with the botanical Code (Sheikh et al. 2018) for genera that are unarguably in Amoebozoa governed by the Zoological Code. Issues such as these have been thoroughly discussed in the past (Adl et al. 2007; Lahr et al. 2012). It has been the responsibility of this committee to discuss and arbitrate published phylogenetic hypotheses, proposals for new names and name changes. Underlying these discussions are principles of nomenclatural priority in the spirit of the codes of nomenclature.

A classification is unlike a phylogenetic tree in a publication, where the discovery of new clades, branches, or robust nodes ultimately leads to proposing new names. Newly named clades and nodes have their utility in phylogenetic analysis and discussion, but do not need to be formalized in the classification immediately. An overwhelming number of spent names have thus accumulated, with an increasing frequency over the past four decades, most of which are no longer—or never were—in common use. Many of these names were ephemeral, as their monophyly did not stand the test of (time) statistical analysis. The proliferation of these names reflects a methodological error practiced by some. That is to formalize names prematurely and try to reorganize classifications single‐handedly. As we argued before (Adl et al. 2012), this must be done with care, respecting nomenclatural priority, published as a proposal or a phylogenetic hypothesis first, to be verified by the community, and only eventually considered for change in the classification. The task of refereeing and classifying falls on Society committees representing communities of professionals. The very formal and slow process of voting to conserve or reject names through the tradition of the botanical code takes years as it has to proceed through committees and then approved by vote on the floor of the congress at 6‐year intervals. That is, however, too slow for the pace of changes today given the rate at which new information is becoming available.

In contrast to a phylogenetic tree, a classification system belongs to a community of users, and it is generated through discussions of the available evidence, for pragmatic purposes of teaching, curation, organizing data, archiving and communicating with a common language. It is a commonly agreed point of reference. It is not to be reimagined or re‐done at will by one individual. The Linnaean system that we have inherited has detailed codes of nomenclature that guide and regulate how living organisms are named, names changed and classified. The elaborate rules arise from disputes and mistakes made in the past, in part out of respect for each other's work. Instead of providing a long list of rejected and invalid names, we can specify that those not selected in this classification were considered *nomina ambigua, nomina perplexa, nomina dubia, nomina nuda* or did not have nomenclatural priority and are declared *nomina rejicienda*.

Another proposed classification of prokaryotes and eukaryotes was published recently (Ruggiero et al. 2015). This effort may be reasonable in their classification of the prokaryotes, but the eukaryote section does not pass standards of modern biology. Specifically, it is their refusal to use monophyly as a guiding principle, but to argue to retain “ancestral (paraphyletic) taxa when it seemed beneficial to do so” instead, even where monophyletic clades are already established. Their insistence on using a hodge‐podge of names that do not have nomenclatural priority, and that poorly describe the taxa included, further reduces its usefulness.

## Classification

The super‐groups utilized since 2005 (Adl et al. 2005; Simpson and Roger 2004) are revised as follows (Fig. 1):

1. Eukaryotes now form two Domains called Amorphea and Diaphoretickes, with several additional clades that do not group into a third Domain.
2. In the Amorphea, the Opisthokonta, Breviatea and Apusomonadida now form a robust clade, as noted earlier (Adl et al. 2012), called Obazoa. Within the Opisthokonta, the Holozoa and Nucletmycea(/Holomycota) are robust clades with improved resolution of the basal sister lineages. In the Holozoa, the sponges and the other animals group together as the Metazoa (Porifera, Placozoa, Ctenophora, Cnidaria, Bilateria). In addition, a sister clade to the Amorphea comprising several genera was recently described as CRuMs (Brown et al. 2018).
3. There are two sister clades in Opisthokonta, the Holozoa and the Nucletmycea (/Holomycota). They share several characters, including synthesis of extracellular chitin in an exoskeleton, cyst/spore wall or cell wall of filamentous growth and hyphae; the extracellular digestion of substrates with osmotrophic absorption of nutrients; and other cell biosynthetic and metabolic pathways. Genera at the base of each clade are amoeboid and phagotrophic.
4. The Archaeplastida, Sar and several other clades remain a monophyletic clade under Diaphoretickes. The clade Cryptista comprising the cryptomonads, kathablepharids and *Palpitomonas* is well recognized and robust, although placement of its node within the Diaphoretickes remains problematic. In some but not all analyses, the clade appears inside the Archaeplastida. This position has always occurred from time to time in some phylogenies with weak support, but there is now stronger support for this association. We are not committed to their inclusion within the Archaeplastida but do note its likelihood. The inclusion of the Cryptista in the Archaeplastida would expand that group without affecting its defining criteria. Questioning the single origin of a plastid within the Archaeplastida is a rare minority opinion. Yet, the possibility of more than one plastid origin must not be ruled‐out until the cryptomonads are robustly positioned.
5. The new robust support for the Cryptista clade is accompanied by a similarly robust support for a clade comprising the Centroplasthelida and Haptophyta as the Haptista within the Diaphoretickes.
6. Nodes at the base of the Alveolata are better resolved with additional genera. The placeholder name Protalveolata is no longer required.
7. The Excavata comprise three clades: the Metamonada, the Discoba, and the Malawimonada. Their mutual relationships, as well as their relationships to other clades of eukaryotes, remain uncertain. We have dropped the supergroup Excavata in favour of the informal Excavates when referring to the “Discoba, Metamonada, Malawimonada”, as *Incertae sedis* in eukaryotes. The Excavates and several clades and genera fall outside of the two principal domains, but do not cluster together into a third domain.

This classification will serve as a primary starting reference for the taxonomic framework developed by UniEuk (unieuk.org; Berney et al. 2017), the Society supported, consensus‐driven, community‐based and expert‐driven international initiative to maintain a universal taxonomy for, at least, microbial eukaryotes. A specific aim of the UniEuk project is to apply one taxonomic framework to all genetic data in the International Nucleotide Sequence Database Collaboration (INSDC) repositories, which includes DDBJ (ddbj.nig.ac.jp), GenBank (ncbi.nlm.nih.gov) and ENA (ebi.ac.uk/ena) databases. The system's broad use and preservation will be ensured by a direct implementation of the UniEuk taxonomic framework into the ENA (European Nucleotide Archive) at EMBL‐EBI ( http://www.ebi.ac.uk/ena). The project will capture our collective knowledge on eukaryotic diversity, evolution, and ecology via three main modules (EukRef, EukBank and EukMap). EukRef (eukref.org; del Campo et al. 2018) uses a standardized, open‐source bioinformatics pipeline to generate homogenous, high‐quality curation of sequences (primarily 18S rDNA) available in INSDC databases. EukRef is fully operational; outputs include (on a lineage‐by‐lineage basis) taxonomically curated sequences, sequence alignments, phylogenetic trees and metadata. EukBank is a public repository of (primarily V4 18S rDNA) high‐throughput metabarcoding data sets, centralized at ENA, with standardized protocols for submitting data sets and metadata. EukMap (eukmap.unieuk.org) is an editable, user‐friendly representation of the UniEuk taxonomy in the form of a publicly navigable tree, where each node/taxon is associated with contextual data (taxonomic and ecological information, links to representative images, etc.). It will be operational by 2019 and will allow registered community members to directly interact with and inform the taxonomic framework, and to flag taxonomy issues requiring revision. As a whole, the UniEuk system will represent a community hub to centralize, standardize, and promote global knowledge on eukaryotic diversity, taxonomy and ecology.

## Clarification of terms for trophic functional groups

Several terms were clarified to correct misuse of terminology in publications. In 2005, these were: eukaryote, prokaryote, algae, zoosporic fungi, protozoa, zooplankton, phytoplankton, cyst, spore and cilium. In 2012, they were related to the cytoskeleton and motility: lobopodia, lamellipodia, filopodia, granuloreticulopodia, reticulopodia, axopodia, centriole, centrosome, microtubular organizing centre (MTOC), basal body, kinetosome, kinetid and mastigont. In this revision, they pertain to trophic functional groups.

In addition to descriptions of morphology that accompany specimen, which is critical for understanding cell function and interpreting phylogenetic trees, improved descriptions of site and food preferences are required for an ecological interpretation of the role in the community and ecosystem. Often species lack sufficient description of the collection site or feeding habit.

To compare environmental DNA data sets, adequate metadata is necessary to select appropriate samples for comparison. The same issue exists when trying to re‐isolate a species or to verify the type specimen. Therefore, it is important that the environment and habitat is sufficiently described. Merely stating marine, terrestrial or soil is grossly inadequate. The soil, for example, is heterogeneous horizontally at the sub‐millimetre to regional scales. It is also stratified through the profile, and across the diameter of each ped. Whether a soil or aquatic sample, solution chemistry and site physical parameters contribute to define the niche space.

Because we care about nomenclature and the exact meaning of words and of names of things, especially species and their groupings into nodes and stems on phylogenetic trees, it is equally important to care for how we describe sampling sites and feeding habits. There are two parts to describing the feeding habit: what is eaten and how it is eaten.

Species that release enzymes extracellularly to digest substrates in their habitat, are generally called *saprotrophic* or *lysotrophic*, and contribute to the decomposition of organic matter. One incredible resource is FunGuild (Nguyen et al. 2016, ( https://github.com/UMNFuN/FUNGuild) to determine substrate utilization for saprotrophic fungi. Probably all eukaryotes are capable of osmotrophy, the acquisition of soluble nutrients through the cell membrane. For example, plants obtain their carbon for *photosynthesis* from the air, as well as some oxygen—however, they rely on osmotrophy through the roots to obtain all the other elements they need. Osmotrophy occurs through the ciliary pit, by pinocytosis, by diffusion, and by various membrane transport proteins. Some species have no alternative form of acquiring energy, are very poor at decomposing substrates and are strict *osmotrophs* relying on dissolved nutrients. *Detritus* eaters ingest particles derived from cells and tissues, decomposing organic matter, starch granules, plant or animal debris, or wood (microchip) fragments.

Species that eat other species are called *consumers*, and there are a variety of terms to describe the functional groups. Some acquire suspended particles in the solution and accumulate the particles *by filtration* into an oral region or cytostome (not filter‐feeders, as they do not feed on filters). The size of particles filtered out of the liquid depend on the current generated, and the structure of the feeding apparatus (Fenchel 1986), and it is a good idea to specify what size prey are ingested. The remaining consumers fall into two categories, the grazers and predators. *Grazers*, like a cow in a field of grasses, browse and ingest from surfaces covered with potential food items (e.g. an amoeba on a lawn of bacteria, or on soil particle surfaces). *Predators* pursue scarce prey according to optimal foraging theory, typically handle one prey at a time, and it is mathematically distinct (e.g. a *Jakoba* ingesting one bacterium). Species gather bacteria by filtration prior to phagocytosis, or directly by phagocytosis; it is best to specify “bacteria by filtration” or “bacteria by phagocytosis”. A popular term bacterivore has the unintended implication of voraciously devouring (*voracitas* L.) which is a false description of how many bacteria eaters acquire their prey, and an incomplete description. Use it, but be aware that some readers and reviewers will be more discriminating. In contrast, the more appropriate term –trophy (*trophe* Gr.), to eat for food and nourishment, sounds more awkward in English. For species that ingest unicellular protists by phagotrophy, the correct term is *cytotrophy*. Bacterium (Ehrenberg 1838) has been the word used to refer to a prokaryotic cell, while cell (Dutrochet 1824; Schleiden 1838; Schwann 1839) has been used since to refer to a eukaryotic cell. *Mixotrophy* refers to photosynthetic species that also ingest food by phagocytosis, and heterotrophs that retain prey plastids and symbionts.

There are two distinct mechanisms to feed on algal filaments (cellulosic cell wall) or fungal hyphae (chitinous cell wall). One mechanism is to slurp the filaments like noodles and ingest them, and the other is to penetrate through the cell wall. Those that puncture through phagocytose cytoplasm, and some species even penetrate inside to ingest cytoplasm along the tube or in the spore. It is best to distinguish between the cell wall material to digest and the mechanism of ingestion. Thus, we have *mycotrophy* or *phycotrophy*, by either swallowing (*devoratis* L.) or by penetrating (*penetrando* L.).

In microbial food webs, there are also consumers of consumers, typically by predation, that are equivalent above‐ground or in aquatic systems to carnivores (meat eaters), or other functional groups. Although *2° consumers*,* 3° consumers*, and so on exist in microbial food webs, it is hardly correct to refer to carnivores in food webs where there is no meat.

Another poorly crafted term one encounters, albeit rarely, is eukaryovory. Although there are famous examples of eukaryovory (Saint‐Exupéry 1943), eukaryotes eating eukaryotes can include *parasitism*, as intracellular or extracellular parasites, on hosts that are protists or multicellular, with various grades of host specificity, and it is a poor substitute for cytotrophy.

We have summarized the higher level classification of eukaryotes in Table 1, with an estimate of the known number of genera, and providing informal phylum and class designations to help orient the student and users along the hierarchy, or nodes on a phylogenetic tree. The revised classification of eukaryotes is presented in Table 2, and genera that have not been studied enough to place in the classification are listed in Table 3 as *incertae sedis* Eukarya. Table 4 provides recommended primers for analysing DNA from environmental samples, noting that the choice of primers and depth of sequencing are important sources of variation between studies. Appendix S1 provides additional supporting literature that we considered important to understand the changes. Appendix S2 provides more detail about the trophic functional assignments across protists, by noting exceptions at the genus level. Appendix S3 provides a standardized guide to East Asian users for the new terminology.

**Table 1:** Higher ranks of the eukaryotes suggesting the position of Linnaean ranks, and the number of known genera

AMORPHEA
CRuMs (O) **11** Collodictyonidae (F) Rigifilida (F) *Mantamonas* (G) Amoebozoa **255** Incertae sedis **21** Tubulinea (P) **93** Corycida (C) Echinamoebida (C) Elardia (C) (Arcellinida **63**) Evosea (P) **106** Variosea (C) Eumycetozoa (C) Cutosea (C) Archamoebea (C) Discosea (P) **35** Flabellinia (C) Stygamoebida (C) Centramoebia (C) Obazoa Apusomonadida (F) **6** Breviatea (F) **4** Opisthokonta Holozoa **25** (without Choanoflagellata, Porifera and Metazoa) Incertae sedis Holozoa: *Corallochytrium*,* Syssomonas* Ichtyosporea (O) Choanoflagellata (C) **57** Metazoa Porifera (P) **742** Hexactinellida (C) Homoscleromorpha (C) Demospongiae (C) Calcarea (C) *Trichoplax* (G/F?) Cnidaria (P) Ctenophora (P) Bilateria (K, ˜35 P) Nucletmycea Rotosphaerida (O) **9** (Fungi) ˜**8,600** Opisthosporidia (O) Aphelidea (F) **4** Cryptomycota (F) **3** Microsporida (O?/F) >**150** Blastocladiales (O) **14** Chytridiomycetes (C) **140** Dikarya **˜8,000** Ascomycota (P) **˜6,400** Basidiomycota (P) **˜1,600** Mucoromycota (P) **˜140** Neocallimastigaceae (F) **11** *Olpidium* (G) Zoopagomycota (P) **˜200**
DIAPHORETICKES
*Incertae sedis*:* Microhelliela maris, Ancoracysta twista*, Rappemonads, Telonemia, Picozoa
Cryptista (C) **21** Cryptophyceae (O) *Palpitomonas* (G) Haptista (P) Haptophyta (C) **80** Pavlovales (F) Prymnesiophyceae (O) Centroplasthelida (C) **16** Pterocystida (O) Panacanthocystida (O) Archaeplastida Glaucophyta (F) **4** Rhodophyceae (P) **850** Cyanidiales (O) Proteorhodophytina (O/C) Eurhodophytina (C) Chloroplastida (72,000 sp + Embryophyta) Chlorophyta (P) Ulvophyceae (C) Trebouxiophyceae (C) Chlorophyceae (C) Chlorodendrophyceae (C) Pedinophyceae (C) Chloropicophyceae (C) Picocystophyceae (C) Pyramimonadales (C) Mamiellophyceae (C) *Nephroselmis* (G) Pycnococcaceae (C) Palmophyllophyceae (C) Streptophyta (P) *Chlorokybus atmophyticus* *Mesostigma viridae* Klebsomidiophyceae (F) Phragmoplastophyta (C) Zygnemataceae (F) Coleochaetophyceae (O) Characeae (F) Embryophyta (K) Sar Stramenopiles (P) Bigyra (C) **49** Opalinata (O) Placidida (F) Bicosoecida (O/F) Sagenista (C) Labyrinthulomycetes (O) Pseudophyllomitidae (F?) Gyrista (C) **31** excluding Peronosporomycetes and Ochrophyta Developea (F) Hyphochytriales (O) Peronosporomycetes (C/O) **46** *Pirsonia* (G) Actinophryidae (F) Ochrophyta^a^ Chrysista (C)^a^ Chrysophyceae (O) Eustigmatales (O) Phaeophyceae (O) Raphidophyceae (O) Schizocladia (O) Xanthophyceae (O) Diatomista Bolidophyceae (F) Diatomea (C) **˜400** Alveolata, others **26** Colpodellida (O) Perkinsidae (F) Colponemidia (F) *Acavomonas* (G) *Oxyrrhis marina* Dinoflagellata (P) **300** Syndiniales (O) Noctilucales (O) Dinophyceae (C) Apicomplexa (P) **˜350** Incertae sedis: Agamococcidiorida (F), Protococcidiorida (F) Aconoidasida (C) Conoidasida (C) Ciliophora (P) Karyorelictea (C) **18** Heterotrichida (O) **58** Spirotrichea (C) **139** (+Hypotrichia) Hypotrichia **183** Armophorea (O) **41** Litostomatea (C) **263** Phyllopharyngea (C) **263** Colpodea (C) **73** Nassophorea (C) **23** Plagyopylea (C) **15** Oligomymenophorea (C) **433** Rhizaria Cercozoa (P) **>>204** Cercomonadida (F) Paracercomonadida (F) Glissomonadida (O) Viridiraptoridae (F) Pansomonadidae (F) Sainouridae (F) Thecofilosea (C) Imbricatea (P) **89** Spongomonadida (F) Marimonadida (F) Variglissida (F) Silicofilosea (C) Metromonadea (F) Granofilosea (O) Chlorarachnea (F) Endomyxa (P) **>34** Vampyrellida (O) Phytomyxea (O) *Filoreta* (G) *Gromia* (G) Ascetosporea (C) Retaria Foraminifera (P) **˜950** Monothalamea (C/O?) Tubothalamea (C) Globothalamea (C) Radiolaria (P) Acantharea (C) **50** Taxopodida (F) **1**+environmental clades Polycystinea (C) **˜470** *Aquavalon* (G) *Tremula* (G) *Incertae sedis Eukarya*: Excavates Metamonada **133** Fornicata (P) Parabasalia (P) Preaxostyla (P) Discoba **94** Jakobida (P) *Tsukubamonas* (G) Heterolobosea (P) Euglenozoa (P) Euglenida (C) Diplonemea (O) Symbiontida (F) Kinetoplastea (C) Other incertae sedis Eukarya **158**

G = genus; F = family; O = order; C = class; P = phylum; K = kingdom.

a The state of the classification in online databases are too poor to evaluate or work with this clade.

**Table 2:** Classification of the higher ranks of the protists and multicellular organisms. The authority to whom the taxon name is attributed appears immediately after the taxon name. For purposes of nomenclature and stability of names in the classification, we have tried to retain the oldest term that correctly described the grouping, emended if necessary; in the square bracket following are inappropriate and incorrect names used in the literature, or that do not have nomenclatural priority. If the taxon name has been emended herein, the authority is indicated and the reference is to this manuscript (“emend. Adl et al. 2019”). Selected references to the literature since 2012 can be found in Appendix S1. Citations in the notes to this table can be found in the LITERATURE CITED. Named clades are monophyletic as best as we can determine; if paraphyly or polyphyly is suspected, it is indicated by P; robust clades recovered in phylogenetic analysis that do not have morphological diagnosis are indicated by R (ribo‐group); monotypic genera with only one described species are indicated by M; MTOC, microtubular organizing centre. * Denotes genera lacking DNA sequence information or known to require taxonomic revision

**AMORPHEA Adl et al.** **2012**
The least inclusive clade containing *Homo sapiens* Linnaeus 1758, *Neurospora crassa* Shear and Dodge 1927 (both Opisthokonta), and *Dictyostelium discoideum* Raper 1935 (Amoebozoa). This is a node‐based definition in which all of the specifiers are extant; it is intended to apply to a crown clade; qualifying clause—the name does not apply if any of the following fall within the specified clade—*Arabidopsis thaliana* (Linnaeus) Heynhold 1842 (Archaeplastida), *Tetrahymena thermophila* Nanney and McCoy 1976 (Alveolata), *Thalassiosira pseudonana* Hasle and Hiemdal 1970 (Stramenopiles), *Bigelowiella natans* Moestrup and Sengco 2001 (Rhizaria), *Euglena gracilis* Klebs 1883 (Excavata) and *Emiliania huxleyi* (Lohmann) Hay and Mohler 1967 (Haptophyta).
*Incertae sedis * **Amorphea: Obazoa** Brown et al. 2013 (R)
Obazoa is a clade that is robustly recovered in phylogenetic trees and consists of the Opisthokonta and two other clades, Apusomonadida and Breviatea. It is the least inclusive clade containing *Homo sapiens* Linnaeus 1758 (Opisthokonta), *Neurospora crassa* Shear & Dodge 1927 (Opisthokonta), *Pygsuia biforma* Brown et al. 2013 (Breviatea) and *Thecamonas trahens* Larsen & Patterson 1990 (Apusomonadida).
•**Apusomonadida** Karpov & Mylnikov 1989 Gliding cells (5–15 µm), with dorsal cell membrane underlain by thin theca extending laterally and ventrally as flanges that delimit a broad ventral region from which pseudopodia develop in most genera; with two heterodynamic cilia, the anterior enclosed by sleeve‐like extension of flanges to form a proboscis, and the posterior cilium lying within the ventral region; tubular mitochondrial cristae; phagocytosis of bacteria. *Amastigomonas*,* Apusomonas*,* Chelonemonas*,* Manchomonas*,* Multimonas*,* Podomonas*,* Thecamonas*. •**Breviatea** Cavalier‐Smith 2004 Amoeboid gliding cells (10–15 µm) with single anteriorly directed apical cilium and in some isolates a second posteriorly directed cilium; filopodia projecting unilaterally from cell, perpendicular to anteroposterior axis and direction of movement; filopodia forming at anterior end, moving posteriorly as cell moves forward (filopodia appearing attached to substrate), and resorbed at posterior; cell can also produce broad lamellopodia; anaerobic or microaerophilic, with large mitochondrion‐like organelle; ingests bacteria; can form cysts. *Breviata*,* Lenisia*,* Pygsuia*,* Subulatomonas*. **Amoebozoa Lühe 1913,** sensu Cavalier‐Smith 1998 Organisms almost all demonstrating ‘amoeboid activity’^1^ in all or in certain stage(s) of their life cycle. Amoeboid locomotion with steady flow of the cytoplasm or occasional eruptions in some groups; alternatively, amoeboid locomotion involving the extension and retraction of pseudopodia and/or subpseudopodia with little coordinated movement of the cytoplasm. Cells naked, often with well‐developed, differentiated glycocalyx; in several groups cells are covered with a tectum^2^ or a cuticle^3^. Two groups are testate (enclosed in a flexible or hard extracellular envelope with one to several opening(s)). Mitochondrial cristae tubular (ramicristate), with few exceptions; mitochondria secondarily reduced to mitochondrion‐related organelles (MRO) in archamoebians. Most only reported to be asexual, but sex and life cycles consistent with sex have been reported in all three major lineages—Tubulinea, Evosea and Discosea. Many taxa exhibit either sporocarpic^4^ or sorocarpic^5^ fruiting. Biciliated, uniciliated or multiciliated stages in the life cycle of some taxa; some taxa exhibit reduction of the bikont kinetid to a unikont kinetid. *Incertae sedis* Amoebozoa: Belonocystis*, Boveella*, Biomyxa, Corallomyxa, Gibbodiscus*, Hartmannia*, Malamoeba*, Malpighamoeba*, Microcorycia*, Microglomus*, Oscillosignum*, Parmulina*, Penardochlamys*, Pseudothecamoeba*, Rhabdamoeba*, Schoutedamoeba 6 , Stereomyxa*^7^, Subulamoeba*, Thecochaos*, Triaenamoeba*, Unda*^8^ , Zonomyxa* **• Tubulinea** Smirnov et al. 2005 Organisms producing lobose pseudopodia (lobopodia)^9^. The entire cell or individual pseudopodia (in polypodial cells) are tubular, cylindrical or subcylindrical, rounded in cross‐section. If cells are flattened or branched they are capable of altering the locomotive form from a flattened, expanded one to monopodial or polypodial, with subcylindrical pseudopodia. Monoaxial flow of the cytoplasm in every pseudopodium or in the entire cell. No convincing evidence of ciliate stages^10^. Two groups are testate, and two sorocarpic taxa are known. No sporocarpy has been reported. ••Corycida Kang et al. 2017 Cells covered with flexible, leather‐like coating forming one or several openings used to protrude pseudopodia or are enclosed in hard test made of spicules with multiple apertures. The least inclusive clade containing *Amphizonella* sp.^11^, *Diplochlamys* sp.^11^, *Trichosphaerium* sp.^11^, *Amphizonella*,* Diplochlamys*,* Trichosphaerium* 12. ••Echinamoebida Cavalier‐Smith 2004 (R) Cells tubular, vermiform or flattened, with or without spine‐like subpseudopodia; capable of adopting subcylindrical monopodial form under certain conditions. The least inclusive clade containing *Vermamoeba vermiformis, Echinamoeba silvestris* and *Micriamoeba tesseris. Echinamoeba, Micriamoeba*,* Vermamoeba*. ••Elardia Kang et al. 2017 (R) Cells naked or covered with a hard test; tubular or produce tubular pseudopodia; if flattened or branched, capable of altering the locomotive form to monopodial or polypodial, with tubular pseudopodia. The least inclusive clade containing *Amoeba proteus, Arcella intermedia* and *Rhizamoeba saxonica*. •••Leptomyxida Pussard & Pons 1976, sensu Smirnov et al. 2017 Naked amoebae with locomotive form altering from a flattened expanded or reticulate one to a subcylindrical monopodial one when in rapid movement or under specific conditions; adhesive uroidal structures always present. Flabellula, Gephyramoeba*, Leptomyxa, Rhizamoeba. •••Arcellinida Kent 1880 Cell covered with hard or highly rigid organic or mineral extracellular test consisting of either self‐secreted elements (calcareous, siliceous or chitinoid), a sheet‐like chitinoid structure, or recycled organic or mineral particles bound together, with a single main opening. *Incertae sedis* Arcellinida: *Argynnia*, Awerintzewia*, Geamphorella*, Jungia*, Lagenodifflugia*, Lamtoquadrula*, Leptochlamys*, Maghrebia*, Microquadrula*, Paraquadrula*, Pentagonia*, Pseudawerintzewia*, Pomoriella*, Pontigulasia*, Physochila, Schoenbornia*, Sexangularia*, Zivkovicia*. ••••Sphaerothecina Kosakyan 2016 Test rigid or more or less flexible, either completely chitinoid or comprising recycled organic or mineral particles held together by an organic cement, or composed of self‐secreted chitinoid or siliceous elements; always rounded in radial symmetry but varying in height from flattened saucer‐shaped, hemispheric or more elongated to egg‐shaped; pseudostome circular or lobed, surrounded by a collar; produce thick, digitate pseudopodia. Antarcella*, Arcella, Cornuapyxis*, Cucurbitella*, Cyclopyxis*, Distomatopyxis*, Ellipsopyxella*, Ellipsopyxis*, Geopyxella*, Lamptopyxis*, Netzelia, Protocucurbitella*, Pseudocucurbitella*, *Pyxidicula*, Suiadifflugia*, Trigonopyxis*^13^. ••••Difflugina Meisterfeld 2002, sensu Kosakyan et al. 2016 Test either completely chitinoid or comprising organic or mineral particles, or recycled diatom frustules, scales or plates (often from Euglyphida), or composed of siliceous, calcite or chitinoid self‐secreted plates (idiosomes) held together by an organic cement; may produce thick, digitate pseudopodia, or move using a flattened, disc‐like hyaline projection. Alocodera, Apodera, Bullinularia, Centropyxis, Certesella, Cornutheca, Difflugia, Geoplagiopyxis*, Gibbocarina, Hyalosphenia, Hoogenraadia*, Lesquereusia, Longinebela, Mrabella, Nebela, Oopyxis*, Padaungiella, Paracentropyxis*, Plagiopyxis*, Planhoogenraadia*, Planocarina, Porosia, Proplagiopyxis*, Protoplagipyxis*, *Quadrulella*,* Spumochlamys,* probably Conicocassis*, Microchlamys*, Pseudonebela*. ••••*Heleopera sphagni* Leidy 1874^14^ Test reinforced with mineral particles, slit‐like aperture, numerous small digitate pseudopodia; with symbiotic *Chlorella*. ••••Phryganellina Bovee 1985 Test proteinaceous, with calcified inner layer, or completely chitinoid with recycled mineral or organic particles; pseudopodia conical, pointed, consist solely of the hyaloplasm, sometimes branched and may anastomose. Cryptodifflugia, Meisterfeldia*, Phryganella, Wailesella*. •••Euamoebida Lepşi 1960, sensu Smirnov et al. 2011 Naked amoebae with tubular, subcylindrical pseudopodia (or the entire cell is monopodial and subcylindrical); no alteration of the locomotive form; no adhesive uroidal structures; sorocarpic development in some species. Amoeba, Cashia*, Chaos, Copromyxa, Copromyxella*, Deuteramoeba, Glaeseria, Hartmannella, Hydramoeba*, Nolandella, Parachaos 8 , Polychaos, Ptolemeba, Saccamoeba, Trichamoeba*.
**•Evosea** Kang et al. 2017 (R) Representatives of this clade can vary across almost the entire range of morphologies seen in Amoebozoa. Many members have complex life cycles^15^ that include amoeboid, ciliated and fruiting stages. Some species appear to be exclusively ciliated with no amoeboid features. Most taxa with only a subset of these life cycle stages. The least inclusive clade containing *Physarum polycephalum* (Eumycetozoa), *Protostelium nocturnum* (Variosea), *Squamamoeba japonica* (Cutosea), and *Entamoeba histolytica* (Archamoebea). ••Variosea Cavalier‐Smith et al. 2004 (R)^16^ Amoebae elongated or flabellate during locomotion and sometimes branched to reticulate, with long, pointed, often branching and occasionally anastomosing subpseudopodia; ciliated cells may be the sole state*,* or present as ciliated amoebaes, or be one state in a life cycle that also includes obligate amoebae; the kinetid of ciliates bikont or unikont, associated at least with one cone of microtubules; several taxa contain a sporocarp state. The least inclusive clade containing *Flamella balnearia, Protostelium nocturnum, Acramoeba dendroida* and *Phalansterium solitaruium*. •**•**•Flamellidae Cavalier‐Smith 2016 (R) Flattened amoebae capable of forming fan‐shaped or semicircular locomotive form with numerous fine, tapering hyaline subpseudopodia, directed anteriorly; ciliated stages unknown. The least inclusive clade containing *Flamella aegyptia* and *Telaepolella tubasferens. Flamella, Telaepolella*. •**•**•*Filamoeba* Page 1967 (R) Flattened amoebae, fan‐shaped, triangular or crescent‐shaped in locomotion, with numerous spine‐like hyaline subpseudopodia, directed anteriorly; ciliated stages unknown. The least inclusive clade containing *Filamoeba nolandi* and *F. sinensis. Filamoeba*. •**•**•*Heliamoeba* Berney, Bass & Geisen 2015 (R) (M) Binucleate amoebae with filose‐like pseudopodia; with clearly distinct cell body always present, the pronounced pseudopodia making up most of the total cell dimension; cell body rarely branching; never reticulate; pseudopodia often branching and present mostly in the anterior and posterior parts of fully extended cells, or all around the cell body in more condensed forms; cell movement slow; ciliated stages unknown. *Heliamoeba mirabilis*. •**•**•Protosteliida Olive & Stoianovitch 1966, sensu Shadwick et Spiegel in Adl et al. 2012; Sporocarpic amoebae with acutely pointed subpseudopodia and usually orange pigmentation contained in lipid droplets visible *en masse*; one taxon ciliated amoebae with 1–9 unikont kinetids not associated with nucleus; taxa without cilia with ring‐shaped component in a nucleus‐associated MTOC; sporocarps of variable morphology, with long, delicate stalk supporting single spore. The least inclusive clade containing *Protostelium nocturnum* and *Protostelium mycophaga*. *Protostelium* 17. ••**•**Fractovitellida Lahr et al. 2011, sensu Kang et al. 2017 (R)^18^ Uninucleate, flabellate to branching amoebae; several members sporocarpic, one species with ciliated amoebae and obligate amoebae. The least inclusive clade containing *Soliformovum irregularis, Nematostelium gracile* and *Acramoeba dendroida*. Acramoebidae, Schizoplasmodiidae, Soliformoviidae. •••**•**Acramoebidae Smirnov, Nassonova & Cavalier‐Smith 2008 (R) (M) Uninucleate amoebae, flattened highly branched, with very slender, pointed, sometimes branched hyaline subpseudopodia never forming a network; ciliated stages unknown. *Acramoeba dendroida*. •••**•**Schizoplasmodiidae Shadwick & Spiegel in Adl et al. 2012 Exclusively sporocarpic group with multinucleate, highly branching and reticulate amoebae, or plasmodia; plasmodia without directional streaming and a beaded appearance during mitosis; prespore cells developing from multinucleate fragments of plasmodia; sporocarp stalk with cup‐like apophysis that fits into annular hilum on spore; spores always multinucleate; one taxon (*Ceratiomyxella*) with scale‐covered ciliated amoebae that can develop from zoocysts derived from the plasmodium that germinates from the spore or from a fragment of a feeding plasmodium; kinetids bikont. The least inclusive clade containing *Ceratiomyxella tahitiensis, Nematostelium ovatum, Schizoplasmodium cavostelioides*. *Ceratiomyxella, Nematostelium, Schizoplasmodium*. •••**•**Soliformoviidae Lahr & Katz 2011 (R) Uninucleate amoebae, thin, flabellate, fan‐shaped to irregularly triangular with numerous finely pointed hyaline subpseudopodia often heavily concentrated at the leading edge during locomotion; lobed nucleoli present in at least one stage of the life cycle; multiple small contractile vacuoles; some species more branched than others; MTOC absent; ciliated stages unknown; two species sporocarpic; sporocarps deciduous in one species and ballistosporous in another. The least inclusive clade containing *Soliformovum irregularis* and *Grellamoeba robusta. Grellamoeba*,* Soliformovum*. ••**•** *Angulamoeba* Berney, Bass & Geisen 2015 (R) Uninucleate, branching amoebae with slender, pointed and/or filose‐like, sometimes branched pseudopodia; trophozoites moving slowly; main cell body elongated, consisting of several main branches often with smaller lateral branches, never forming a network; numerous fine pseudopodia concentrated mostly at the extremities of the lateral and terminal branches, but can be formed anywhere around the cell body; multiple contractile vacuoles; some species with ciliated amoebae stages. The least inclusive clade containing *Angulamoeba microcystivorans* and *A. fungorum*. *Angulamoeba*. ••**•**Cavosteliida Shadwick & Spiegel in Adl et al. 2012 (R) Sporocarpic group with various types of amoebae, from uninucleate amoebae to multinucleate reticulate plasmodia, all characterized by producing long, filose, subspeudopodia, anastomosing in some taxa; one taxon with ciliated amoebae and obligate amoeba with possible sex in the life cycle; ciliated amoebae possesses one to several, reduced unikont kinetids per cell, not associated with the nucleus; species without ciliated amoebae have akinetid amoebae that germinate from spores; sporocarps in all species with single, nondeciduous spores; morphology variable and taxon specific; spores of all species displaying some type of sculpturing; cysts of some species displaying sculpturing as well. *Cavostelium, Schizoplasmodiopsis, Tychosporium*. ••**•** *Ischnamoeba* Berney, Bass & Geisen 2015 (R) Uninucleate, branching naked amoebae, cells usually thin, extended and flat, showing no well‐defined cell body, except often a slight broadening in the area containing the nucleus; never reticulate, with whole cells often bent, but not extensively branched; branching more pronounced in condensed cells or in condensed parts of individual cells; very thin pseudopodia produced almost exclusively at distal parts of cells and more pronounced in condensed organisms, often branching; movement too slow to be directly observable; ciliated stages unknown. The least inclusive clade containing *Ischnamoeba montana* and *Ischnamoeba* sp. isolate F4 (Genbank: KP864094). *Ischnamoeba*. ••**•** *Darbyshirella* Berney, Bass & Geisen 2015 Multinucleate, highly branching and reticulate amoebae with slender, pointed, sometimes branched and anastomosing pseudopodia; the whole cell body is strongly branching and narrow, especially in the most extended parts, while more condensed parts are wider; posterior end usually pointed with no or few pseudopodia and no branching; many contractile vacuoles present; movement very slow; ciliated stages unknown. The least inclusive clade containing *Darbyshirella terrestris* and *Darbyshirella* sp. (Genbank KP864088). *Darbyshirella*. •**•**•Holomastigida Lauterborn 1895 Rounded cells with multiple radiating projections, which may be cilia arising from the solitary kinetosomes. Artodiscus*, Multicilia. •**•**•*Dictyamoeba* Berney, Bass & Geisen 2015 (M) Multinucleate, highly branching and reticulate naked amoebae with slender, pointed, sometimes branched pseudopodia; movement of entire cells very slow; the main cell body is multiply branched and anastomosing, and can grow into giant networks (up to several mm) with intersecting segments of varying width and numerous terminal branching areas; abundant fine pseudopodia are concentrated mostly at the extremity of lateral and terminal branches, especially in complex networks, but can be formed anywhere around the cell body in simpler forms; ciliated stages unknown. *Dictyamoeba vorax*. •**•**•*Arboramoeba* Berney, Bass & Geisen 2015 (M) Multinucleate, highly branching and reticulate amoebae; cell body indistinct; nuclei and other cytoplasmic contents are distributed across the whole network, with network significantly more complex at the anterior front, forming a wide, very densely reticulate, non‐permeable front where phagocytosis occurs; posterior part of the cells is much less reticulate and branching; branching, filose‐like, pseudopodia are mostly present at the anterior front of the cell; very strong vacuolar activity across the whole network; movement very slow; ciliated stages unknown. *Arboramoeba reticulata*. •**•**•*Phalansterium* Cienkowski 1870 Uniciliate sedentary cells, colonial or solitary; cilium arises from the apical part of the cell; one centriole per kinetid; ciliary pocket usually surrounded by a collar; some species form short tapering cytoplasmic projections and move over the substratum using the conformation of their body or producing cytoplasmic eruptions. The least inclusive clade containing *Phalansterium solitarium* and *P. filosum*. *Phalansterium*. •**•Eumycetozoa** Zopf 1884 sensu Kang et al. 2017 (R) All known members fruit, either sorocarpically (Dictyostelia), or sporocarpically (Myxogastria, Protosporangiida); with a life cycle having a single haploid amoeboid state (Dictyostelia); or a life cycle with a bikont ciliated amoebae state that gives rise to a non‐ciliate obligate amoeboid state from which sporocarps develop (Myxogastria and Protosporangiida); ciliated amoebae of myxogastrids and protosporangiids and amoebae of dictyostelids flat and form wide pseudopodia with acutely pointed subpseudopodia and no pronounced streaming of the granular cytoplasm; where sex is well studied, the zygote cannibalizes haploid amoebae. The least inclusive clade containing *Dictyostelium discoideum, Physarum polycephalum* and *Ceratiomyxa fruticulosa*. **•**••Dictyostelia Lister 1909, sensu Sheikh et al. 2018 (R) Sorocarpic amoebae, also known as cellular slime moulds or social amoebae, with stalked fruiting bodies developing from aggregation of amoebae; sorocarps consisting of stalks with terminal sori of haploid spores; stalks (sorophores) acellular (acytosteloid), cellular and unbranched or sparsely branched (dictyosteloid), or cellular and regularly branched with whorls of lateral branches (polysphondyloid); cells of stalks dead, consisting of walls, only, at maturity; spores usually ellipsoid, spherical in some species; cysts present in some species; sex, when present associated with a zygote that causes haploid amoebae to aggregate towards it such that the aggregate lays down a common cyst wall to form a macrocyst in which the haploid cells are ingested and digested by the zygote and meiosis occurring in the zygote prior to germination of the macrocyst; amoebae aciliate, haploid, with nucleus with peripheral reticulate nucleolus; upon starvation, amoebae aggregating, often in streams, towards an aggregation centre that signals with a chemical attractant (an acrasin) with aggregate developing into a slug‐shaped, multicellular mass that can migrate then fruit or fruit directly; anterior cells becoming stalk cells in dictyosteloid and polyspondyloid species and bulk of the remaining cells becoming spores. *Acytostelium, Cavenderia, Coremiostelium, Dictyostelium, Hagiwaria, Heterostelium, Polysphodylium, Raperiostelium, Rostrostelium, Speliostelium, Synstelium, Tieghemostelium*, probably—Coenonia* 19. **•**••Myxogastria Macbride 1899 [Myxomycetes Link 1833, sensu Haeckel 1866] (R) Sporocarpic amoebae where a multinucleate obligate amoeba—the plasmodium—differentiates into one or more multinucleate spore‐forming masses where the cell cleaves into individual, uninucleate spores that undergo meiosis after spore wall development in sexual species; sporocarps can be individual sporangia (with or without stalks), clustered sporangia, aethalia (massive fruiting derived from a whole plasmodium) or plasmodium‐shaped plasmodiocarps; fruiting bodies initially covered by an extracellular peridium and may contain thread‐like spore‐suspending capillitium; spores germinating as bikont ciliated amoebae with rootlets as with Eumycetozoa with rootlet 3 consisting of a band of several microtubules; ciliated amoebae developing into plasmodia (involving plasmogamy and karyogamy of gametic ciliated amoebaes in sexual species); plasmodia usually tubular in cross‐section with streaming of central granular cytoplasm. One species known to lack plasmodial state and one species known to lack ciliated amoebae. **••**••Lucisporidia Cavalier‐Smith 2013 (R) Containing taxa with light‐ or bright‐coloured spores, in mass. Alwisia, Arcyria, Calomyxa, Cornuvia, Cribraria, Dianema, Dictydiaethalium, Hemitrichia, Licea, Lindbladia, Lycogala, Metatrichia, Minakatella*, Oligonema, Perichaena, Prototrichia, Reticularia, Trichia, Tubifera.^20^ **••**••Columellidia Cavalier‐Smith 2015 (R) Containing taxa predominantly with dark coloured spores, in mass. Amaurochaete, Badhamia, Barbeyella, Brefeldia, Clastoderma, Collaria, Colloderma, Comatricha, Craterium, Diachaeopsis, Diachea, Diderma, Didymium, Echinosteliopsis, Echinostelium, Elaeomyxa, Enerthenema, Fuligo, Kelleromyxa, Lamproderma, Leocarpus, Lepidoderma, Leptoderma, Macbrideola, Meriderma, Mucilago, Paradiachea*, Paradiachaeopsis, Physarella, Physarina, Physarum, Protophysarum, Stemonaria, Stemonitis, Stemonitopsis, Symphytocarpus, Willkommlangea.^20^ **•**••Protosporangiida Shadwick & Spiegel in Adl et al. 2012 (R) Exclusively fruiting, with microscopic (protosteloid) sporocarps with a microscopic stalk with one to four, sometimes more, spores; life cycle with ciliated amoebae stage with rootlets as Eumycetozoa with rootlet 3 consisting of a band of only two microtubules; giving rise to a uninucleate to plurinucleate obligate amoeba that develops into one or more sporocarps; prespore cells site of meiotic prophase and meiosis completed in spore complement. **••**••Protosporangiidae Spiegel in Adl et al. 2012 With ciliated amoebae as Protosporangiida; obligate amoeba uninucleate to plurinucleate, often resembling very early developmental stages of myxogastrid plasmodia; individually developing into a single two to four‐spored sporocarp. *Clastostelium, Protosporangium*. **••**••*Ceratiomyxa* Schroeter 1889 With ciliated amoebaes developing from cleavage of germling from a tetranucleate spore; obligate amoeba is multinucleate plasmodium secreting an extracellular slime mound or columns upon which it cleaves into single uninucleate prespore cells that individually develop into a stalked sporocarp bearing a single, tetranucleate spore. *Ceratiomyxa*. ••Cutosea Cavalier‐Smith et al. 2016 Amoebae bounded by a continuous thin, somewhat flexible, envelope separated from the plasma membrane and having oval scale‐like substructure within a denser matrix; small pores penetrate the envelope, allowing subpseudopodia to protrude for very slow, occasional locomotion; locomotive cells flattened, oval, rounded or irregularly triangular. *Armaparvus, Sapocribrum, Squamamoeba*. ••Archamoebea Cavalier‐Smith 1983, sensu Cavalier‐Smith et al. 2004 Amoebae or ciliated amoebae, anaerobic or microaerophilic, free‐living or endobionts of different invertebrate or vertebrate hosts; ciliated amoebae usually with hyaline lateral pseudopodia; unikont, with single kinetosome at the base of cilia, connected to the microtubular cone, in some cases both the kinetosome and the axoneme have atypical complements of microtubules; without typical mitochondria, in several cases mitochondrial derivates, i.e. mitosomes, have been demonstrated. •••Mastigamoebida Frenzel 1897, sensu Cavalier‐Smith 2013 Ciliated amoebaes or amoeboid organisms without cilia. The single motile anterior cilium, when present, associated with microtubular cone connected to the nucleus. Ciliated amoebaes with hyaline lateral pseudopodia. Endamoeba*, Endolimax, Iodamoeba, Mastigamoeba, Mastigina*. •••Pelobiontida Page 1976, sensu Cavalier‐Smith 2013 Anaerobic or microaerophilic ciliated amoebae with slow‐beating monokinetid or immobile polykinetids; ciliated amoebae often with hyaline lateral pseudopodia. *Pelomyxa, Mastigella*. •••*Tricholimax* Frenzel 1897 Ciliated amoebaes with single immobile cilium, microtubular cone associated with nucleus, when several nuclei present, each nucleus is connected to its own microtubular cone; with rhizostyle, derived from the lateral microtubular root. Tricholimax*. •••*Entamoeba* Casagrandi & Barbagallo 1895 Cilia and kinetosomes absent; with mitosomes instead of classical mitochondria; peroxisomes absent; mitosis closed with endonuclear centrosome and spindle; reduced Golgi dictyosome. *Entamoeba*. •••*Rhizomastix* Alexeieff 1911 Ciliate ciliated, possessing a rhizostyle arising from the basal body of the cilium and probably derived from the microtubular cone. *Rhizomastix*.
**•Discosea** Cavalier‐Smith et al. 2004, sensu Smirnov et al. 2011 Flattened naked amoebae, never producing tubular, subcylindrical pseudopodia and never altering the locomotive form to the tubular, subcylindrical one; cytoplasmic flow polyaxial or without a pronounced axis; ciliated stages unknown; several taxa sporocarpic. ••Flabellinia Smirnov et al. 2005 Flattened generally fan‐shaped, oblong or irregularly triangular cells, never with pointed subpseudopodia. •••Thecamoebida Schaeffer 1926, sensu Smirnov et al. 2011 Flattened amoebae, oblong, lingulate or irregularly triangular amoebae, usually with dorsal folds and/or ridges; anterior hyaloplasm often forms an anterolateral crescent and rarely occupies more than half of the body length; never produce discrete pseudopodia or subpseudopodia. *Sappinia, Stenamoeba, Stratorugosa, Thecamoeba*. •••Dermamoebida Cavalier‐Smith 2004 Oblong, lancet‐shaped or irregularly triangular cells; with a smooth cell surface or with few wide ridges, never wrinkled; short, wide triangular pseudopodia and, in some, subpseudopodia of dactylopodial type; thick cell coat, multilayered or consisting of tightly packed helical structures. *Dermamoeba, Mayorella, Paradermamoeba*. •••*Mycamoeba* Blandenier et al. 2017^21^ (M) Flattened lingulate amoebae without differentiated glycocalyx; with complex life cycle where active cells transform into coccoid stages, which undergo subsequent buddings, eventually turning into ramified structures (pseudomycelia) with spherical cysts in a terminal position on the ramifications; these pseudomycelia disappear and cysts are released prior to germinating into active trophozoites. *Mycamoeba gemmipara*. •••Dactylopodida Smirnov et al. 2005, sensu Kang et al. 2017 Locomotive form mostly has a shape of an irregular triangle with basement directed forward; wide anterior hyaloplasm; fibrous axial cores both in dactylopodia and in the floating pseudopodia. Cunea, Janickina*^22^ *, Korotnevella*,* Neoparamoeba*,* Paramoeba, Pseudoparamoeba, Vexillifera*. •••Vannellida Smirnov et al. 2005 Locomotive form fan‐shaped to spatulate; cells do not form discrete pseudopodia or subpseudopodia; wide anterior hyaloplasm up to a half of the cell; posterior granuloplasm concentrated in a “hump”, often raised over the substratum; one species of *Vannella* (*V. fimicola*) with protosteloid sporocarps. Clydonella, Lingulamoeba, Paravannella, Pessonella*^23^ *, Ripella, Vannella*. ••Stygamoebida Smirnov et al. 2011 (P)^24^ Flattened, elongate amoebae resembling tooth‐picks or splinters, temporarily acquiring forked or branched form; elongate, expanded area of anterior hyaloplasm; mitochondrial cristae flattened, ribbon‐like; MTOC known in one species. *Stygamoeba, Vermistella*. ••Centramoebia Cavalier‐Smith et al. 2016 (R) MTOC located near the dictyosome; several taxa with protosteloid sporocarpy. The least inclusive clade containing *Pellita catalonica, Gocevia fonbrunei, Endostelium zonatum, Acanthamoeba castellanii*. •••Acanthopodida Page 1976 Flattened with prominent subpseudopodia, flexible and tapering to a fine tip and sometimes furcated near their base (acanthopodia); without adhesive uroid; trilaminate MTOC; some species in culture appear as a branched, flattened sheet; at least two taxa, *Acanthamoeba* and *Luapeleamoeba,* contain species with protosteloid sporocarpy. *Acanthamoeba, Balamuthia, Dracoamoeba, Luapeleamoeba, Protacanthamoeba, Vacuolamoeba*. •••Pellitida Smirnov & Cavalier‐Smith 2011, sensu Kang et al. 2017 Thick cell coat envelops the entire cell with the exception of subpseudopodial tips and is integrated with plasma membrane, or is located on the dorsal surface only, and is loosely attached to the plasma membrane; MTOC, when known, trilaminate. One genus, *Endostelium,* with several protosteloid sporocarpic species. Endostelium, Gocevia, Paragocevia*, Pellita. •••Himatismenida Page 1987 Dorsal surface covered with a flexible coat without defined aperture; ventral surface entirely or partly naked; can form ventral flattened sheet of hyaloplasm used for adhesion to the substratum; MTOC, when known, bar‐like. *Cochliopodium*,* Ovalopodium, Parvamoeba*. **Opisthokonta Cavalier‐Smith 1987**, emend. Adl et al. 2005 Single posterior cilium without mastigonemes, present in at least one life cycle stage or secondarily lost; with a pair of kinetosomes or centrioles, sometimes modified; flat (rarely tubular) mitochondrial cristae in the unicellular stage. •**Holozoa** Lang et al. 2002 (R) The most inclusive clade containing *Homo sapiens* Linnaeus 1758 (Metazoa), but not *Neurospora crassa* Shear and Dodge 1927 (Fungi). This is a branch‐based definition in which all the specifiers are extant. The apparent composition of Holozoa is Filasterea (*Ministeria, Capsaspora, Pigoraptor*), Ichthyosporea, *Corallochytrium*,* Syssomonas,* Choanoflagellata, and Metazoa. The primary reference phylogenies are Carr et al. (2017, Fig. 2), Hehenberger et al. (2017, Fig. 2), Torruella et al. (2015, Fig. 1), Simion et al. (2017, Fig. 3), Whelan et al. (2017, Fig. 2).*Incertae sedis* Holozoa: *Corallochytrium* 25 *limacisporum* Raghu‐Kumar 1987 (M) Spherical single cells 4.5–20 µm in diameter; binary fissions releasing numerous elongated amoeboid cells; marine saprotrophic, usually recovered from coral reefs in the Indian Ocean; free‐living cells grow as osmotrophic chitin cell‐walled schizont as ichthyosporeans; a ciliary apparatus has been observed in culture conditions and further demonstrated by molecular means (conserved ciliary toolkit expressed in transcriptome). *Syssomonas* 25 Tikhonenkov, Hehenberger, Mylnikov & Keeling 2017 (M) Predominantly unicellular, roundish uniciliated motile swimming cells; cilium emerges from the middle lateral point of the cell, ended by short acroneme and directs backward; cells naked; cells can form clusters of multiple cells; predatory phagotroph of heterotrophic chrysomonads and bodonids; life cycle includes unicilaited roundish motile swimming cells, ciliated amoeboid cells, amoeboid aciliated cells with filopodia and spherical cysts; known from freshwater. *Syssomonas multiforma*. ••Ichthyosporea^25^ Cavalier‐Smith 1998 [Mesomycetozoea Mendoza et al. 2002] Single‐celled trophic organisms, *Ichthyophonus* with hyphal multinucleated filaments; flat mitochondrial cristae but some may have tubular mitochondrial cristae; if present, single cilium; without collar or cortical alveoli; some species form only elongate amoeboid cells; most animal parasites, some free‐living and saprotrophic (*Sphaeroforma*, LKM51 isolate); chitin reported in cell wall (proven by staining with wheat germ agglutinin and molecular phylogeny of chitin synthases); both marine and freshwater. •••Dermocystida Cavalier‐Smith 1998 [Rhinosporidaceae Mendoza et al. 2001] Zoospore with posterior cilium; flat mitochondrial cristae; when parasite of animals, spherical phenotypes with several 2–20 µm endospores that are eventually released and become mature cells with endospores to continue the parasitic cycle. *Amphibiocystidium ranae, Amphibiothecum penneri, Chromosphaera perkinsii, Dermocystidium, Rhinosporidium seeberi, Sphaerothecum destruens*. •••Ichthyophonida Cavalier‐Smith 1998 [Ichthyophonae Mendoza et al. 2001; Amoebidiidae Reeves 2003] (R) Parasites of fish, arthropods and insects, or free‐living and saprotrophic; usually with flat mitochondrial cristae but *Ichthyophonus* with tubular mitochondrial cristae; some characteristic amoeboid cells, but in others, amoeboid cells absent or unreported. *Abeoforma whisleri, Amoebidium parasiticum, Anurofeca richardsi, Astreptonema, Caullerya mesnili, Creolimax fragrantissima, Eccrinidus flexilis, Enterobryus oxidi, Enteropogon sexuale, Ichthyophonus, Palavascia patagonica, Pseudoperkinsus tapetis, Psorospermium haeckeli, Sphaeroforma arctica; S. tapetis*. ••Filasterea Shalchian‐Tabrizi et al. 2008 Trophic cells naked, unicellular; uninucleate; aerobic with flat mitochondrial cristae; long nontapering tentacles supported by microfilaments, unlike collar in choanoflagellates; phagotrophic. *Capsaspora,* filose amoeba with cystic and aggregative stages; *Ministeria* and *Pigoraptor* with cilium, *Ministeria* is not motile but uses a stalk attached to the substrate; *Pigoraptor,* ciliated amoeba and predator, as *Capsaspora* it can present pluricellular clusters. *Capsaspora, Ministeria, Pigoraptor*. ••Choanoflagellata Kent 1880–1882 [Craspedomonadina Stein 1878; Craspedomonadaceae Senn 1900; Craspedophyceae Chadefaud 1960; Craspédomonadophycidées Bourrelly 1968; Craspedomonadophyceae Hibberd 1976; Choanomonadea Krylov et al. 1980; Choanoflagelliida Lee, Hutner, and Bovee 1985; Choanoflagellatea Cavalier‐Smith 1997 emend. Cavalier‐Smith 1998; Choanomonada^26^ Adl et al. 2005;]^27^ Phagotrophic with collar of actin‐supported microvilli around a single cilium; radial symmetry; solitary or colonial; flat mitochondrial cristae; ciliated basal body associated with ring or multiple arcs of cytoskeletal (cortical) microtubules, with second aciliated basal body located at an angle; fibrillar root if present minor and without obvious banding; central filament in kinetosome transition zone. This is a branch‐based definition including the most recent common ancestor of animals and choanoflagellates (the Urchoanozoan), along with all of its descendants, including *Homo sapiens* Linnaeus 1758 and *Monosiga brevicollis* Ruinen 1938. The Greek root “choanē” (or funnel) refers to the collar, which in the current state of knowledge is a synapomorphy of the clade. Although “Choanozoa” was used previously to refer to an assemblage of protists that later proved paraphyletic, that usage was not adopted, and the name is more appropriately applied as defined here. The informal term “choanimal” and the formal term Apoikozoa have both been previously proposed for the clade containing choanoflagellates and animals, but neither has been formally described nor adopted. In particular, the term “Apoikozoa” is incorrect as the root “apoiko‐” refers to colony formation, which is neither universally present in choanozoans, nor exclusive to them. •••Craspedida Cavalier‐Smith 1997, emend. Nitsche et al. 2011 Extracellular glycocalyx or theca that is entirely organic and does not project above the anterior end of the extended feeding cell; vegetative stage usually sedentary and stalked; brief motile stage for dispersal. ••••Salpingoecidae Kent 1880–1882, emend. sensu Nitsche et al. 2011 Vegetative cells with glycocalyx or theca that is entirely organic; glycocalyx is flexible, nonrestrictive and fibrous; theca is rigid and microfibril‐based; sedentary cells adhere to a surface by a peduncle extending from the base of the glycocalyx or theca; cell division with nonrestricting glycocalyx is longitudinal *in situ*, with restricting theca it is emergent and involves cell becoming amoeboid and dividing outside the theca; juvenile daughter cells disperse as naked; under certain conditions colonies of cells may develop. Type genus: *Salpingoeca* James‐Clark 1867. Recognized genera: *Astrosiga*,* Aulomonas*,* Choanoeca*,* Cladospongia*,* Codonocladium*,* Codonosigopsis*,* Codosiga* (junior synonym *Codonosiga*), *Desmarella* (junior synonyms *Codonodesmus* and *Kentrosiga*), *Dicraspedella*,* Diploeca*,* Diplosiga*,* Diplosigopsis*,* Hartaetosiga*,* Kentia*,* Lagenoeca*,* Microstomoeca*,* Monosiga*,* Mylnosiga*,* Pachysoeca*, Proterospongia*, Salpingoeca*, *Salpingorhiza*,* Sphaeroeca*,* Stagondoeca*,* Stelexomonas*,* Stylochromonas*. •••Acanthoecida Cavalier‐Smith 1997, emend. Nitsche et al. 2011 Cells surrounded by a basket‐like lorica of siliceous costae comprising rod‐shaped costal strips and a partial or entire organic matrix on inner surface. ••••Acanthoecidae Norris 1965, emend. sensu Nitsche et al. 2011 Lorica with costae arranged in two layers, outer longitudinal and inner helical; occasionally only one layer around cell body, in which case costae are helical; adult loricate cells sedentary; cell cycle, and lorica production accord to nudiform condition; cell division is diagonal resulting in both daughter cells facing forwards; upper daughter cell (juvenile) is naked with cilium for dispersal; juvenile attaches to surface, withdraws cilium and deposits costal strips in correct orientation; strips are accumulated in vertical bundles on the surface of the spindle‐shaped cell body; costal strips destined for longitudinal costae are deposited first followed by those for the inner layer of costae; lorica assembly is achieved by a forwards and clockwise rotation which extends costal strips to form mature pattern of costae. Type genus: *Acanthoeca* Ellis 1929. Recognized genera: *Acanthoeca, Helgoeca*,* Polyoeca*,* Savillea*. ••••Stephanoecidae Leadbeater 2011 Costae arranged in two layers with longitudinal costae outermost; internal costal layer is helical in posterior chamber of some species, but usually comprises transverse costae (rings) in the anterior chamber of lorica and some species only possess transverse rings; cell cycle and lorica production accords to tectiform condition; costal strips are deposited in inverted orientation; strips destined for the inner layer of costae are deposited first followed by those for the outer layer of longitudinal costae; costal strips are exocytosed through anterior end of cell and accumulated in bundles at the top of the inner surface of the collar; cell division is inverted with juvenile daughter cell being turned upside down and pushed into accumulated strips and emerging from parent lorica backwards; costal strips destined for longitudinal and helical costae are located vertically on juvenile cell, costal strips destined for transverse costae are located horizontally; once free of parent lorica, juvenile cell constructs new lorica immediately and there is no swimming dispersal stage. Type genus: *Stephanoeca* Ellis 1929. Recognized genera: *Acanthocorbis*,* Amoenoscopa*,* Apheloecion*,* Bicosta*,* Calliacantha*,* Calotheca*,* Campanoeca*,* Campyloacantha*,* Conion*,* Cosmoeca*,* Crinolina*,* Crucispina*,* Diaphanoeca*,* Didymoeca*,* Kakoeca*,* Monocosta*,* Nannoeca*,* Parvicorbicula*,* Platypleura*,* Pleurasiga*,* Polyfibula*,* Saepicula*,* Saroeca*,* Spinoeca*,* Spiraloecion*,* Stephanacantha*, Stephanoeca*, *Syndetophyllum*. ••Metazoa Haeckel 1874, emend. Adl et al. 2005 [Animalia Linnaeus 1758; Eumetazoa Bütschli 1910] Reproduction sexual through an egg cell, fertilized usually by a monociliated sperm cell with acrosome; embryonic development with blastula followed by gastrulation that begins the differentiation into endoderm, ectoderm, mesoderm, and neuroderm; tissues organized into organs that share tasks for the individual, unless secondarily lost; some secondarily reduced to small number of cells (e.g. Myxozoa Grassé 1970); coordination of cells and tissues by membrane receptors that respond to ligands through elaborate signal transduction; characteristic cell–cell junctions with belt desmosomes or zonulae adherentes; basal lamina and extracellular matrix with collagen and other fibrous proteins (laminin, nidogen, perlecan); heterotrophic nutrition with secretion of digestive enzymes and osmotrophy through a digestive tract; without cell wall; ectoderm completely surrounding body, and endoderm surrounding a digestive tract; sensory cells in epithelium; nervous tissue in organized network; epithelial actin–myosin‐based contractile cells between endoderm and ectoderm; some tissues with phagotrophic cells. Subdivisions beyond Porifera and *Trichoplax* not shown. •••Porifera^28^ Grant 1836 [Parazoa Sollas 1884] Flat mitochondrial cristae; sexual species, zygotes forming larva (nine known larval types) or juveniles; asexual reproduction by gemmules, budding or fragmentation; sessile adult; differentiation of larva to a variety of cell types, including choanocytes, amoeboid cells and cells with granular inclusions; cell types transformable into other types as necessary; cells more or less independent; without mesoderm, nervous tissue, desmosomes, localized gonad or glandular digestive cells. ••••Hexactinellida Schmidt 1870 Exclusively marine, and especially in the deep sea; siliceous spicules triaxonic, hexactinic; square axial proteinaceous filament in spicules, whole sponge formed by a single continuous multinucleate syncytium, with some differentiated cells; electrical conductance across body; reproduction when known is viviparous with a trichimella larvae. •••••Amphidiscophora Schulze 1886 Amphidisc spicules. Amphidiscosida Schrammen, 1924. •••••Hexasterophora Schmidt 1870 Hexaster spicules. Lychniscosida Schrammen 1903; Lyssacinosida Zittel 1877; Sceptrulophora Mehl 1992; Hexasterophora incertae sedis. ••••Demospongiae Sollas 1885 Verongimorpha and Keratosa do not have (for the most part) siliceous spicules, Heteroscleromorpha have a high diversity of siliceous spicules; spicules differentiated in meglascleres and microscleres, triangular axial proteinaceous filament in spicules; larva with outer ciliated cells; one family (Cladorhizidae) with extracellular digestion, by amoeboid cell aggregation of captured crustacean prey; one order Spongillida living in freshwater. •••••Verongimorpha Erpenbeck, Sutcliffe, De Cook, Dietzel, Maldonado, van Soest, Hooper and Wörheide 2012 Mostly with spongin skeleton, otherwise with siliceous spicules (Chondrilla), or no skeleton at all. Synapomorphies include the following ultrastructure characters: orientation of accessory centriole, the nuclear apex, the Golgi apparatus and similarities in embryonic development. Chondrillida Redmond, Morrow, Thacker, Diaz, Boury‐Esnault, Cárdenas, Hajdu, Lobo‐Hajdu, Picton, Pomponi, Kayal and Collins 2013; Chondrosiida Boury‐Esnault and Lopes 1985; Verongiida Bergquist 1978. •••••Keratosa Grant 1861 Demospongiae with a skeleton made of spongin fibre; spongin fibres are either homogenous or pithed and strongly laminated with pith grading into bark. One genus has a hypercalcified basal skeleton (Vaceletia). Dendroceratida Minchin 1900; Dictyoceratida Minchin, 1900. •••••Heteroscleromorpha Cárdenas, Pérez and Boury‐Esnault 2012 Demospongiae with a skeleton composed of siliceous spicules which can be monaxones and/or tetraxones and when they are present, microscleres are highly diversified. Agelasida Hartman 1980; Axinellida Lévi 1953; Biemnida Morrow and Cárdenas 2015; Bubarida Morrow and Cárdenas 2015 Clionaida Morrow and Cárdenas 2015; Desmacellida Morrow and Cárdenas 2015; Haplosclerida Topsent 1928; Merliida Vacelet 1979; Poecilosclerida Topsent 1928; Polymastiida Morrow and Cárdenas 2015; Scopalinida Morrow and Cárdenas 2015; Sphaerocladina Schrammen 1924; Spongillida Manconi and Pronzato 2002; Suberitida Chombard and Boury‐Esnault 1999; Tethyida Morrow and Cárdenas 2015; Tetractinellida Marshall 1876; Trachycladida Morrow and Cárdenas 2015. ••••Homoscleromorpha Bergquist 1978 Exclusively marine, from shallow depths to the deep sea; siliceous spicules or no spicules at all, tetraxonic, not differentiated between megascleres and microscleres, without defined axial proteinaceous filament in spicules (only observed in one species); true epithelium; hermaphroditic; viviparous cinctoblastula larva. Homosclerophorida Dendy, 1905. •••••Calcarea Bowerbank 1862 [Calcispongia Johnston 1842] Exclusively marine, from shallow depths to the deep sea; calcium carbonate spicules; viviparous, hermaphrodites. ••••••Calcinea Bidder 1898 Unambiguous characters congruent with molecular phylogenies unclear. Larva amphiblastula. Clathrinida Hartman, 1958; Murrayonida Vacelet1981. ••••••Calcaronea Bidder 1898 Unambiguous characters congruent with molecular phylogenies unclear. Larva calciblastula; hermaphroditic. Leucosolenida Hartman 1958; Lithonida Vacelet 1981. •••*Trichoplax* 29 von Schulze 1883 [Placozoa Grell 1971] (M) Two layers of epithelial cells, with a middle layer of syncytial contractile fibrous cells, and undifferentiated cells; with digestive glandular cells; belt desmosomes or zonulae adherentes connecting adjacent cells; without extracellular matrix; collagen fibres absent; without endoderm, ectoderm, mesoderm or nerve cells; ventral cells having ated kinetosomes with two horizontal fibrillar rootlets and one vertical rootlet; egg cell and aciliate sperm in mid‐layer; asexual binary division of body possible. *Trichoplax adhaerens*.
•**Nucletmycea** Brown et al. 2009 [syn. Holomycota Liu et al. 2009] (R)^30^^,^ 31 The most inclusive clade containing *Neurospora crassa* Shear and Dodge 1927 (Fungi) and not *Homo sapiens* Linnaeus 1758 (Metazoa). The composition of Nucletmycea is Fungi, Opisthosporidia, Nucleariida and *Fonticula*. The primary reference is Brown et al. (2009). Additional phylogenies are Brown et al. (2009, Fig. 3, 4).This is a branch‐based definition in which all the specifiers are extant. *Incertae sedis* Nucletmycea: Sanchytriaceae Karpov and Aleoshin 2017 Thallus monocentric, epibiotic, penetrates host wall with rhizoid in parasitic species; amoeboid zoospores with posterior pseudocilium; sporangia as in Rhizophydiales (Chytridiomycetes); sexual reproduction not observed. *Amoeboradix*. ••Rotosphaerida Rainer 1968 [junior syn. Cristidiscoidida Page 1987, Cavalier‐Smith 1993, 1998, Nucleariidae Patterson 1983, 1999] Aciliate predominantly spherical or flattened amoebae from which elongated actin‐based filopodia extend; with flat discoid mitochondrial cristae; uninucleate or polynucleate (with few nuclei); free‐living phagotrophs that feed on bacteria, cyanobacteria and algae. Some sorocarpic, e.g. *F. alba;* some have symbionts, e.g. *N. thermophila*. *Fonticula, Nuclearia, Parvularia*. *Incertae sedis* Rotosphaerida: *Pompholyxophrys, Lithocolla, Vampyrellidium, Pinaciophora, Rabdiophrys, Rabdiaster*. ••Fungi^32^ R.T. Moore 1980 This is a minimum‐crown clade definition:the smallest crown clade containing *Rozella allomycis* F.K. Faust 1937, *Batrachochytrium dendrobatidis* Longcore, Pessier and D.K. Nichols 1999, *Allomyces arbusculus* E.J. Butler 1911, *Entomophthora muscae* (Cohn) Fresen1856, *Coemansia reversa* Tiegh. and G. Le Monn 1873, *Rhizophagus intraradices* (N.C. Schenck and G.S. Sm.) C. Walker and A. Schüßler 2010, *Rhizopus oryzae* Went and Prins. Geerl. 1895, *Saccharomyces cerevisiae* Meyen 1838, and *Coprinopsis cinerea* (Schaeff.) Redhead, Vilgalys and Moncalvo 2001. The primary reference phylogeny is James et al. (2006: Fig. 1); see also James et al. (2013: Fig. 2), Karpov et al. (2013: Fig. 3), Paps et al. (2013: Fig. 1), Chang et al. (2015: Fig. 1), Torruella et al. (2015: Fig. 1) and Spatafora et al. (2016: Fig. 1). Its composition is *Rozella*, Microsporidia, Aphelida, Chytridiomycota, Neocallimastigomycota, Blastocladiomycota, Mucoromycota, Zoopagomycota, Ascomycota and Basidiomycota (Hibbett et al. 2007, Karpov et al. 2014, Spatafora et al. 2016). There are no unambiguous morphological, subcellular or biochemical synapomorphies of Fungi. Most Fungi are filamentous, have chitinous cell walls, lack cilia and have intranuclear mitosis with spindle pole bodies instead of centrioles. •••Opisthosporidia Karpov, Aleoshin & Mikhailov 2014 Opisthokont intracellular parasites/parasitoids with amoeboid vegetative stage. Invasive spores/cysts with chitin cell wall and specialized apparatus for penetration into host cell (penetration tube; posterior vacuole); if present, zoospores with filopodia and/or a posteriorly directed whiplash cilium (functional or rudimental); phagotrophic or osmotrophic. ••••Aphelidea Gromov 2000 [=Aphelida Karpov et al. 2014; =Aphelida Gromov 2000; =Aphelidiomyceta Tedersoo et al. 2018; =Aphelidiomycota Tedersoo et al. 2018; =Aphelidiomycotina Tedersoo et al. 2018; =Aphelidiomycetes Tedersoo et al. 2018;=Aphelidiaceae Tedersoo et al. 2018]^33^ Intracellular parasitoids of algae with phagotrophic amoeboid vegetative stage and complex life cycle; invasive cyst with short infective tube of penetration apparatus; zoospore with filose pseudopodia and/or lamellipodium; zoospores attach to host cell wall and encyst, then cyst penetrates host wall with chitin tube, its contents migrates into the host becoming the phagotrophic amoeba which engulfs host cell contents; this results in a characteristic central food vacuole with red‐brownish excretory body; parasite growth and subsequent nuclear divisions lead to multinucleate plasmodium which consequently produces either a resting spore rounded to oval with a thick smooth cell wall, or zoospores released from host wall; mitochondrial cristae from tubular to lamellar. *Amoeboaphelidium, Aphelidium, Pseudoaphelidium, Paraphelidium*. ••••Rozellida Lara et al. 2010, emend Karpov and Aleoshin 2014 [Cryptomycota M. D. M. Jones & T. A. Richards 2011; Rozellomycota Doweld 2013; Rozellosporidia Karpov et al. 2017; Rozellomycotina Tedersoo et al. 2018] (R) Unicellular, holocarpic, zoospores single‐celled with a single posterior cilium; cysts with chitin cell wall; endobiotic (intracellular or intranuclear) parasites; known to be parasites on, at least, Chytridiomycota, Blastocladiomycota, Peronosporomycetes, Basidiomycota and the green alga *Coleochaete*. Related environmental DNA sequences are known, found widely in soil, marine, and fresh water. Related environmental DNA sequences are known, found widely in soil, marine, and fresh water. Chitin or cell wall may be secondarily lost due to parasitic habit. *Rozella*. ••••Microsporidia Balbiani 1882 Obligate intracellular parasites, usually of animals but also protists such as Amoebozoa, Ciliophora or Apicomplexa; without mitochondria and peroxisomes, with mitosomes; spores with inner chitin wall and outer proteinaceous wall; without kinetosomes, centrioles or cilia; centrosomal plaque; extrusive specialized polar tube for host penetration; sexual, asexual or both. Contains numerous diverse lineages currently poorly defined by morphology, found ubiquitously in soil, marine and fresh water. Subdivisions uncertain. *Amblyospora, Amphiacantha, Buxtehudia, Caudospora, Chytridiopsis, Desportesia, Encephalitozoon, Enterocytozoon, Glugea, Hessea, Metchnikovella, Mitosporidium, Nosema, Nucleophaga, Paramicrosporidium, Spraguea, Vairimorpha*. •••Blastocladiales Petersen 1909 [= Blastocladiineae Petersen 1909, Blastocladiomycota T. Y. James 2007, Blastocladiomycetes T. Y. James 2007] Thallus monocentric or polycentric; aerobic to facultatively anaerobic, found in aquatic and terrestrial environments, saprobic and/or parasitic; uniciliated motile cells with microtubules radiating anteriorly from the proximal end of the kinetosome and continuing on to wrap around a cone‐shaped nucleus that also terminates near the kinetosome and is capped by a mass of membrane‐bound ribosomes; no electron‐opaque plug in kinetosome transition zone; one side‐body complex (= microbody–lipid globule complex); reproduces asexually by unciliated cells, while sexual reproduction occurs through fusion of planogametes with a sporic type of meiosis. *Allomyces, Blastocladia, Blastocladiella, Blastocladiopsis, Catenomyces, Catenophlyctis, Caternaria, Coelomomyces, Coelomomycidium, Paraphysoderma, Physoderma, Polycaryum, Sorochytrium, Urophlyctis*. •••Chytridiomycota Doweld 2001, emend. M. J. Powell in Adl et al. 2019 Thallus monocentric, polycentric or filamentous; uniciliated zoospores with a posteriorly directed cilium, nine ciliary props, microbody–lipid globule complex (MLC) consisting of a cisterna which may be simple or fenestrated (=rumposome), microbodies and mitochondria associated with lipid globules; Golgi apparatus with stacked cisternae; nuclear envelope fenestrated at poles during mitosis; reproduction asexual by uniciliated zoospores and where known sexually by zygotic meiosis; found in soil and water as saprotrophs but also parasitic on animals, plants, protists and other fungi. ••••Chytridiomycetes de Bary 1863, emend. Cavalier‐Smith 1998, emend. M. J. Powell in Adl et al. 2019 Thallus monocentric or rhizomycelial polycentric, endobiotic or epibiotic; aerobic. Zoospore ciliary apparatus posterior and includes a non‐ciliated centriole and a ciliated kinetosome, typically with kinetosome‐associated structures; the MLC cisterna is adjacent to the lipid globule; asexual reproduction by posteriorly uniciliated zoospores, sexual reproduction not oogamous. *Incertae sedis* Chytridiomycetes: *Blyttiomyces, Dangeardia*. •••••Caulochytriales Doweld 2014 Thallus monocentric, eucarpic with endogenous development; parasitic, penetrating host with haustorium; sporangium inoperculate, sessile or aerial at tip of hollow‐stalk. Zoospore with posteriorly directed, laterally inserted cilium; non‐ciliated centriole at 45° angle to kinetosome and joined by uniformly dense material; pulsating vacuole; scattered ribosomes; striated rhizoplast joining kinetosome and nuclear envelope; MLC with one to numerous lipid globules, branched microbody, simple membrane cisternae and spherical mitochondria. *Caulochytrium*. •••••Chytridiales Cohn 1879, emend. Schröter 1892, emend. D. J. S. Barr 1980, emend. D. J. S. Barr 2001, emend. Letcher and Powell 2006, emend. Mozl.‐Standr 2009, emend. Vélez et al. 2011 Thallus monocentric or polycentric rhizomycelial, sporangia operculate or inoperculate. Zoospores covered by cell coat over body; containing a MLC composed of microbodies, mitochondria and fenestrated or simple cisterna adjacent to lipid globules, paracrystalline inclusion, an electron‐opaque plug at base of cilium; microtubule root when present a bundle of parallel microtubules extending from the side of kinetosome to MLC cisterna; kinetosome‐associated structure a shield, saddle, globule, wing, stacked plates or combination; ribosomes aggregated around or near the nucleus; non‐ciliated centriole parallel to ciliated kinetosome and connected to it by fibrous material; nucleus not associated with kinetosome; *Avachytrium, Chytridium, Chytriomyces, Delfinachytrium, Dendrochytrium, Dinochytrium, Fayochytriomyces, Irineochytrium, Obelidium, Odontochytrium, Pendulichytrium, Physocladia, Podochytrium, Pseudorhizidium, Siphonaria, Rhizidium, Rhizoclosmatium*. •••••Cladochytriales Mozl.‐Standr 2009 Thallus epibiotic or endobiotic; eucarpic, monocentric or polycentric with intercalary swellings; sporangium operculate or inoperculate; rhizoidal axis apophysate or nonapophysate, and rhizoids can be catenulate, isodiametric or tapering; zoospores with ribosomal aggregation; MLC with fenestrated cisterna; non‐ciliated centriole parallel to ciliated kinetosome and joined by fibrillar bridge, ciliary plug at base of cilium, up to 25 cross‐linked microtubules in a cord‐like microtubular root situated between the kinetosome and the fenestrated cisterna; lacking paracrystalline inclusions and kinetosome‐associated structure. *Allochytridium, Catenochytridium, Cladochytrium, Cylindrochytridium, Endochytrium, Nephrochytrium, Nowakowskiella, Septochytrium*. •••••Gromochytriales Karpov & Aleoshin 2014 Thallus monocentric, eucarpic with endogenous development and inoperculate sporangium; parasite of green algae; penetrates host with a single, weakly branched rhizoid. Zoospore spherical to oval; ribosomes clustered, posterior to the nucleus, covering anterior ends of kinetosome and centriole, and lacking associated endoplasmic reticulum; MLC anterior with fenestrated cisterna and microbody associated with lipid globule and microbody sandwiched between lipid globule and nucleus, mitochondria scattered; non‐ciliated centriole at a 30° angle to kinetosome and connected by a dense band over their anterior ends; kinetosome‐associated structure a short, straight spur. *Gromochytrium*. •••••Lobulomycetales D. R. Simmons 2009, emend. D. R. Simmons 2012 Thallus monocentric, eucarpic with endogenous development; sporangium operculate or inoperculate; rhizoids isodiametric ranging from 0.5 to 1.5 μm wide. Zoospore contains a ribosomal aggregation surrounded by endoplasmic reticulum, an opaque ciliar plug with anterior or posterior plug extensions, non‐ciliated centriole and kinetosome parallel and joined with a dense amorphous bridge, one to three lipid globules in the MLC; MLC cisterna absent, simple or irregularly fenestrated; microtubules, kinetosome‐associated structures and Golgi apparatus absent. *Algomyces, Alogomyces, Cyclopsomyces, Clydaea, Lobulomyces, Maunachytrium*. *Incertae sedis* Lobulomycetales: *Algochytrops*. •••••Mesochytriales Doweld 2013 Thallus monocentric, eucarpic with endogenous development and inoperculate sporangium; parasite of green algae; penetrates host with peg‐like haustorium. Zoospore spherical to oval; ribosomes dispersed; MLC posterior to nucleus with a single lipid globule, large mitochondrion, lobed microbody, and fenestrated cisterna proximal to kinetosome; rough endoplasmic reticulum encircles MLC; non‐ciliated centriole with a veil and at 30° angle to kinetosome and connected along their sides by a broad, dense fibrillar bridge; ciliar plug absent. *Mesochytrium*. •••••Polychytriales Longcore & D.R. Simmons 2012 Thallus monocentric or polycentric lacking intercalary swellings; monocentric species with multiple rhizoidal axes; sporangia operculate or inoperculate. Zoospore with ribosomal aggregation; non‐ciliated centriole parallel or at slight angle (14–24°) to kinetosome and connected by dense material throughout their lengths, length of non‐ciliated centriole equal to or longer than its diameter; one to many lipid globules in MLC; may or may not possess each of the following: a ciliary plug, a kinetosome‐associated structure as a spur, a fenestrated cisterna, and if present 1‐3 microtubular roots. *Arkaya, Karlingiomyces, Lacustromyces, Neokarlingia, Polychytrium*. •••••Polyphagales Doweld 2014 Thallus interbiotic with multiple rhizoids, functions as a prosporangium; Zoospore with posteriorly located MLC with fenestrated cisterna and single lipid globule; ribosomes aggregated with layers of endoplasmic reticulum; disc‐like striated rootlet; resting spores sexually formed. *Polyphagus*. •••••Rhizophydiales Letcher 2006, emend. Letcher 2008 Thallus monocentric, eucarpic, inoperculate or operculate. Zoospore with one or more of the following characters: microtubular root with one or more stacked microtubules extending from one side of the kinetosome to a MLC cisterna on the lipid globule; ribosomes aggregated with endoplasmic reticulum binding or ramifying through; mitochondria; microbodies, and fenestrated or simple cisterna associated with lipid globule (MLC); non‐ciliated centriole either parallel or slightly angled to kinetosome and connected by a fibrillar bridge; fibrillar bridge either perpendicular to or diagonal between kinetosome and non‐ciliated centriole; kinetosome‐associated structure when present a solid spur, laminated spur or shield; no electron‐dense ciliary plug. *Alphamyces, Angulomyces, Aquamyces, Batrachochytrium, Betamyces, Boothiomyces, Collimyces, Coralloidiomyces, Dinomyces, Gammamyces, Globomyces, Gorgonomyces, Halomyces, Kappamyces, Paranamyces, Operculomyces, Paranamyces, Pateramyces, Paludomyces, Protrudomyces, Staurastromyces, Terramyces, Rhizophydium, Uebelmesseromyces, Ulkenomyces, Urceomyces*. *Incertae sedis* Rhizophydiales: *Homolaphlyctis*. •••••Rhizophlyctidales Letcher 2008 Thallus monocentric or polycentric, eucarpic; interbiotic sporangium that is either inoperculate or endo‐operculate with one to several discharge short tubes; multiple rhizoidial axes. Zoospore possesses a non‐ciliated centriole that is at an acute angle (<40°) to the kinetosome and attached by a fibrillar bridge along the length of the non‐ciliated centriole; multiple mitochondria; ribosomes either dispersed or aggregated in the cytoplasm; MLC with one to many lipid globules, simple MLC cisterna when present; without microtubules. *Arizonaphlyctis, Borealophlyctis, Rhizophlyctis, Sonoraphlyctis*. •••••Spizellomycetales D. J. S. Barr 1980, emend. D. J. S. Barr 1983 Thallus monocentric, sporangia epibiotic or interbiotic. Zoospore with nucleus either closely associated with the kinetosome or connected by its root; ribosomes dispersed in the cytoplasm; MLC cisterna simple; non‐ciliated centriole typically at an angle to the ciliated kinetosome; without electron‐opaque material in the kinetosome transition zone. *Barromyces, Brevicalcar, Bulbosomyces, Fimicolochytrium, Gaertneriomyces, Gallinipes, Geranomyces, Kochiomyces, Powellomyces, Spizellomyces, Thoreauomyces, Triparticalcar*. *Incertae sedis* Polyphagales: *Endocoenobium*. •••••Synchytriales Doweld 2014, emend. Longcore, DR Simmons & Letcher 2016 The least inclusive clade of the Chytridiomycetes that includes *Synchytrium taraxaci* and *Synchytrium* species included in James et al. 2006, Smith et al. 2014, Longcore et al. 2016. *Synchytrium*. *Incertae sedis* Synchytriales: *Micromyces*. •••Dikarya Hibbett et al. 2007, emend. Hibbett et al. 2018 Unicellular or filamentous Fungi, lacking cilia, often with a dikaryotic state. The least inclusive clade that contains Ascomycota and Basidiomycota and Entorrhizomycete. Incertae sedis Dikarya: Entorrhizomycetes Begerow et al., 2007 [=Entorrhizaceae R. Bauer and Oberw. 1997; =Entorrhizales R. Bauer and Oberwinkler 1997; =Entorrhizomycetes Begerow et al. 2007; =Entorrhizomycota R. Bauer et al. 2015; =Entorrhizomycotina Tedersoo et al. 2018] (M) Phytoparasitic fungi infecting roots with regularly septate coiled hyphae; septal pores without Woronin bodies or membrane caps. Includes Entorrhizales (*Entorrhiza),* Talbotiomycetales. ••••Ascomycota Cavalier‐Smith 1998 Sexual reproduction within asci (saccate structures); meiosis usually followed by mitosis to produce from one to over 1,000 ascospores, but usually eight; ascospore walls form inside ascus; mating types heterothallic, homothallic (selfing) or both; may reproduce sexually (teleomorph) or asexually (anamorph) only, or both sexually and asexually (holomorph); asci cylindrical, fusiform, clavate or globose, persistent or evanescent, with or without a fruiting structure (ascoma, ‐ata); asci developing directly from ascogenous hyphae, from a crozier or from a single cell; asexual reproduction by conidiospores (mitospores) formed by fragmentation of vegetative hyphae (thallic), blastically from single cells, hyphae or conidiophores; vegetative body of single cells or tubular, septate filaments (hyphae); septa with simple pores, except for those associated with ascogenous hyphae and asci; cell walls lamellate with a thin electron‐dense outer layer and a relatively thick electrontransparent inner layer, consisting of varying proportions of chitin and glucans; saprobes, endophytes, parasites (especially on plants) or lichen forming. •••••Taphrinomycotina O. E. Eriksson and Winka 1997 Mycelium present or absent; asci produced from binucleate cells; do not form croziers or interascal tissue. ••••••*Archaeorhizomyces* Rosling & T. James 2011 [=Archaeorhizomycetes Rosling and T. James 2011; =Archaeorhizomycetales Rosling and T. James 2011] Phylogenetically placed among Taphrinomycotina, differing by mycelial growth on MMN agar together with an association with roots of living plants. Distinctive molecular characters (nuclear large subunit rRNA). Synonymous to “Soil Clone Group 1 (SCG1)”. *Archaeorhizomyces finlayi*. ••••••*Neolecta* Spegazzini 1881 [=Neolectomycetes Eriksson and Winka 1997; =Neolectales Landvik et al. 1997; =Neolectaceae Redhead 1977] (M) Mycelium present, multinucleate; ascomata apothecial, stalked, fleshy; interascal tissue absent; cylindrical asci formed from binucleate cells undergo karyogamy, meiosis, and one mitotic division to produce eight cylindrical ascospores, thin‐walled, walls blueing in iodine; ascus apex truncate, slightly thickened below ascus wall, with wide apical slit, persistent; ascospores ellipsoidal to globose, hyaline, aseptate; anamorph unknown; saprobic; found in wet mixed woodlands. *Neolecta flavovirescens*. ••••••*Pneumocystis* P. Delanoë & Delanoë 1912 [=Pneumocystidales O. E. Eriksson 1994; =Pneumocystidomycetes Eriksson and Winka 1997; =Pneumocystidaceae] (M) Mycelium and ascomata absent; vegetative cells thin‐walled, irregularly shaped, uninucleate, dividing by fission; sexual reproduction initiated by fusion of two vegetative cells followed by karyogamy, cyst wall formation, meiosis, and in some, one mitotic division, to produce 4–8 nuclei that are delimited by the cyst (ascus) vesicle; ascospore walls are deposited between the delimiting membranes; cyst walls rupture to release ascospores; extracellular parasite of mammalian lungs. *Pneumocystis carinii*. ••••••*Schizosaccharomyces* Lindner 1893 [=Schizosaccharomycetaceae Beij. ex Klöcker 1905; Schizosaccharomycetales O. E. Eriksson et al. 1993; =Schizosaccharomycetes O. E. Eriksson and Winka 1997] (M) Mycelium absent or poorly developed; ascomata absent; vegetative cells cylindrical, proliferating by mitosis followed by cell division to produce two daughter cells; cell wall composition differs from that of species of Saccharomycetes; sexual reproduction initiated by fusion of two vegetative cells to form an ascus; karyogamy and meiosis occur within the ascus to produce four nuclei, which may or may not divide once again mitotically; ascospores aseptate, delimited by enveloping membrane system (EMS), wall formed within bilayers of EMS, wall blueing in iodine, hyaline or pigmented; saprophytes in sugary plant exudates; fermentation positive. *Schizosaccharomyces pombe*. ••••••Taphrinales Gäumann and C. W. Dodge 1928 [=Taphrinomycetes O. E. Eriksson and Winka 1997] Vegetative mycelium mostly absent; ascomata absent; interascal tissue absent; dikaryotic mycelium infects host and proliferates through host tissue; dikaryotic cells or mycelium develop directly into asci, often forming a palisade layer on the host; asci globose or ellipsoidal, eight‐spored; ascospores hyaline, aseptate; biotrophic on angiosperms forming galls or lesions; cells bud from ascospores to form a yeast‐like, monokaryotic, saprobic anamorph. *Protomyces, Taphrina*. •••••Saccharomycetales Kudryavtsev 1960 [=Saccharomycetes O.E. Eriksson and Winka 1997; =Saccharomycotina O.E. Eriksson and Winka 1997] Mycelium mostly absent or poorly developed; hyphae, when present, septate, with septa having numerous pores rather than a single septal pore; vegetative cells proliferating by budding or fission; walls usually lacking chitin except around bud scars; ascomata absent; sexual reproduction by fusion of two vegetative haploid cells or fusion of two haploid nuclei in a single cell or within diploid cells, followed by meiosis and, in some cases, one mitotic division to produce either four or eight nuclei; cells undergoing meiosis become asci, ascospores delimited by an enveloping membrane system (EMS); ascospore wall formed within bilayers of EMS; ascospores aseptate, colourless or pigmented, often with wall thickenings of various types; most osmotrophic, some species parasitic on animals. *Ascoidea, Candida, Cephaloascus, Dipodascus, Endomyces, Lipomyces, Metschnikowia, Pichia, Saccharomyces, Scheffersomyces, Trichomonascus, Wickerhamomyces, Yarrowia*. •••••Pezizomycotina O.E. Eriksson and Winka 1997 Mycelium present; hyphae filamentous, septate; septa with simple pores and Woronin bodies; life cycle haploid with a dikaryotic stage immediately prior to sexual reproduction; ascomata discoid, perithecial, cleistothecial or occasionally lacking; antheridium present or absent; ascogonium, ascogenous hyphae, and crosiers present; the penultimate cell of the crozier, in which meiosis and usually one mitotic division occur, becomes the ascus; asci fissitunicate or not fissitunicate, cylindrical, clavate or saccate; asci frequently with ascospore discharge mechanism; usually eight ascospores surrounded by enveloping membrane system; ascospore morphology and pigmentation varied; asexual state present or absent, produced from vegetative hyphae in a thallic or blastic manner; mitospores (conidiospores) varied in morphology and pigmentation. ••••••Arthoniales Henssen & Jahns ex D. Hawksw. and O. E. Eriksson 1986 [=Arthoniomycetes O. E. Eriksson and Winka 1997] Ascomata usually apothecial, occasionally closed with an elongated poroid opening; peridium thin‐ or thick‐walled; interascal tissue of branched paraphysoids in a gel matrix; asci thick‐walled, fissitunicate, blueing in iodine, with or without a large apical dome; ascospores aseptate or septate, sometimes becoming brown and ornamented; anamorphs pycnidial; forming crustose lichens with green algae, lichenicolous or saprobic on plants. *Arthonia, Chrysothrix, Melaspilea, Opegrapha, Roccella, Roccellographa*. ••••••Dothideomycetes O. E. Eriksson & Winka 1997 Ascomata variable (apothecial, perithecial, cleistothecial), formed lysigenously from stromatic tissue (ascolocular); interascal tissue present or absent, of branched paraphysoids or pseudoparaphyses; asci cylindrical to saccate, thick‐walled, fissitunicate, rarely with apical structures; ascospores mostly septate or muriform, colourless to dark brown; anamorphs hyphomycetous or coelomycetous; saprobes, plant parasites, coprophilous or lichen forming. Note that this group partially includes loculoascomycetes. Containing Dothideomycetidae (Capnodiales, Dothideales, Myriangiales)*,* Pleosporomycetidae (Hysteriales, Jahnulales, Mytilinidiales, Pleosporales). *Incertae sedis* Dothideomycetes: Containing Abrothallales (*Abrothallus*), Acrospermales (*Acrospermum, Oomyces*), Asterinales (*Asterina*), Asterotexiales (*Asterotexis*), Botryosphaeriales (*Botryosphaeria, Guignardia, Saccharata*), Eremithallales (*Encephalographa*), Microthyriales (*Microthyrium*), Minutisphaerales (*Minutisphaera*), Monoblastiales (*Monoblastia, Anisomeridium*), Natipusillales (*Natipusilla*), Patellariales (*Baggea, Patellaria*), Phaeotrichales (*Phaeotrichum*), Stigmatodiscales (*Stigmatodiscus*), Strigulales (*Strigulales*), Superstratomycetales (*Superstratomyces*), Trypetheliales (*Laurera, Trypethelium*), Tubeufiales (*Tubeufia*,* Bezerromyces*,* Wiesneriomyces*), Valsariales (*Valsaria*), Venturiales (*Apiosporina, Sympoventuria, Venturia*). ••••••Eurotiomycetes O. E. Eriksson & Winka 1997, emend. Geiser et al. 2006 (R) Morphologically heterogeneous, circumscribed using phylogenetic re‐delimitation to contain Chaetothyriomycetidae, Eurotiomycetidae, Mycocaliciomycetidae and Sclerococcomycetidae. Important industrially and medically; saprobic, pathogenic on animals and rarely on plants, some lineages lichenized. Chaetothyriomycetidae (Chaetothyriales, Pyrenulales, Verrucariales), Coryneliomycetidae (Coryneliales), Eurotiomycetidae (Eurotiales, Onygenales), Mycocaliciomycetidae (Mycocaliciales), Sclerococcomycetidae (Sclerococcales). ••••••Geoglossaceae Corda 1838, emend. Schoch et al. 2009 [=Geoglossales Zheng Wang et al. 2009; Geoglossomycetes Zheng Wang et al. 2009] Ascomata scattered to gregarious, capitate, stipitate; stipe cylindrical, black, smooth to furfuraceous; ascigerous portion capitate, club‐shaped to pileate, indistinguishable from stipe; hymenium surface black, continues with stipe at early development stage; asci clavate, inoperculate, thin‐walled, J+, usually eight‐spored; ascospores elongate, dark‐brown, blackish to hyaline, septate when mature; paraphyses filiform, blackish to hyaline; global distribution, terrestrial, habitat usually boggy and mossy. *Geoglossum, Trichoglossum*. ••••••Laboulbeniomycetes Engler 1898 Mycelium absent except in Pyxidiophorales; cellular thallus hyaline to dark, with basal haustorium present; ascomata perithecial, surrounded by complex appendages, translucent, ovoid, thin‐walled; interascal tissue absent; asci few and basal, not fissitunicate, clavate, thin‐walled, evanescent, maturing sequentially, usually with four ascospores; ascospores two‐celled, hyaline, elongate, one end modified as attachment to host; anamorphs hyphomycetous, spermatial; ectoparasitic on insects, some may be coprophilous. Containing Laboulbeniales, Pyxidiophorales. ••••••Lecanoromycetes O. E. Eriksson & Winka 2001 Ascomata apothecial, discoid, perithecial or elongated, sometimes stalked or immersed, occasionally evanescent; interascal tissue of simple or branched paraphyses swollen at the apices, often with a pigmented or iodine‐staining epithecium; hymenial gel often present; asci not fissitunicate, but thick‐walled, with a thickened, cap‐like apex, often with an internal apical ocular chamber; ascus walls and thickened apex often stains blue with iodine; ascospores one to several septate, occasionally, multiseptate, rarely plurilocular, hyaline or pigmented; anamorphs pycnidial where known; mostly lichen forming with protococcoid algae, with thallus foliose, fructicose, crustose or occasionally absent; some lichenicolous, some saprobic. Containing Acarosporomycetidae (Acarosporales), Lecanoromycetidae (Caliciales, Lecanorales, Lecideales, Leprocaulales, Peltigerales, Rhizocarpales, Teloschistales), Ostropomycetidae (Arctomiales, Baeomycetales, Hymeneliales, Ostropales, Pertusariales, Sarrameanales), Umbilicariomycetidae (Umbilicariales). *Incertae sedis* Lecanoromycetes: Candelariales (*Candelaria, Candelariella*) ••••••Leotiomycetes O. E. Eriksson & Winka 1997 Ascomata apothecial, discoid, cleistothecial, elongated or rarely absent; apothecia stalked or sessile, frequently fleshy, sometimes hairy or with appendages, occasionally stromatic or sclerotioid; interascal tissue of simple paraphyses or absent; peridium thin‐walled; asci typically inoperculate, cylindrical, thin‐walled, not fissitunicate, occasionally with apical pore; apical apparatus variable; ascospores aseptate or transversely septate, hyaline or pigmented and longitudinally slightly asymmetrical; anamorphs occasionally present, hyphomycetous or coelomycetous; saprobes or plant parasites, some lichenized or lichenicolous. Containing Cyttariales (*Cyttaria*), Erysiphales (*Blumeria, Erysiphe, Microsphaera, Oidium, Podosphaera*), Helotiales (*Botryotinia, Bulgaria, Dermea, Hyaloscypha, Lachnum, Leotia, Sclerotinia, Vibrissea*), Rhytismatales (*Ascodichaena, Cudonia, Rhytisma*) and Thelebolales (*Thelebolus, Antarctomyces*). ••••••Lichinales Henssen & Büdel 1986 [=Lichinomycetes Reeb et al. 2004] Ascomata apothecial, discoid, sometimes immersed, occasionally clavate, stalked, setose and fleshy; peridium often not well‐defined; interascal tissue varied; hymenium often stains blue with iodine; asci thin‐walled or apically thickened, not fissitunicate, without well‐defined apical structures, usually with an iodine‐staining outer gelatinized layer; ascospores one‐septate or occasionally multiseptate, ellipsoidal to fusiform, hyaline or pigmented; anamorphs pycnidial; lichenized with cyanobacteria forming crustose, fruticose or foliose often gelatinized thalli. *Gloeoheppia, Heppia*,* Lichina, Peltula*. ••••••Orbiliaceae Nannfeldt 1932 [=Orbiliales Baral et al. 2003; =Orbiliomycetes Eriksson and Baral 2003] Ascomata apothecial, small, waxy, translucent or lightly pigmented; interascal tissue of simple paraphyses, usually with knob‐like apices, united by a matrix; asci minute, not fissitunicate, apex truncate, with J apical rings, often forked at the base; ascospores minute, cylindrical, hyaline, often aseptate; anamorphs hyphomycetous where known; saprobic, often on wet wood. *Halorbilia*,* Orbilia*. ••••••Pezizales J. Schröter 1894 [=Pezizomycetes O. E. Eriksson and Winka 1997] Ascomata apothecial or cleistothecial, usually visible with unaided eye, leathery or fleshy; carotenoids as bright colours to dark, sometimes present; interascal tissue present (paraphyses); asci not fissitunicate, usually elongated, cylindrical but more or less globose in cleistothecial species, thin‐walled, lacking obvious apical wall thickening or apical apparatus, with operculum or vertical slit except in cleistothecial species, forcibly discharging ascospores except in cleistothecial species; ascospores usually ellipsoidal or globose, aseptate, hyaline to darkly pigmented, smooth or ornamented; anamorphs hyphomycetous, where known; saprobes on soil, dead wood or dung; some species hypogeous and mycorrhizal. *Ascobolus, Ascodesmis, Caloscypha, Carbomyces, Chorioactis, Discina, Glaziella Helvella, Karstenella, Morchella, Peziza, Pyronema, Rhizina, Sarcoscypha, Sarcosoma, Tuber*. ••••••Sordariomycetes O. E. Eriksson & Winka 1997 (R) Defined using molecular phylogenetic methods, by a parsimony comparison of small subunit rRNA sequences, containing Boliniales (*Bolinia, Camarops*), Calosphaeriales (*Calosphaeria, Pleurostoma*), Chaetosphaeriales (*Chaetosphaeria, Melanochaeta*), Coniochaetales (*Barrina, Coniochaeta*), Diaporthales (*Cryphonectria, Diaporthe, Gnomonia, Melanconis,* Phyllachorales (*Phaeochora, Phyllachora*), *Pseudovalsa, Schizoparme, Sydowiella, Valsa, Vialaea*), Magnaporthales (*Gaeumannomyces, Magnaporthe, Ophioceras*), Ophiostomatales (*Kathistes, Ophiostoma*), Sordariales (*Annulatascus, Cephalotheca, Chaetomium, Lasiosphaeria, Neurospora, Sordaria*). *Incertae sedis* Sordariomycetes: Koralionastetales (*Koralionastes, Pontogeneia*), Lulworthiales (*Lindra, Lulworthia, Spathulospora*), Meliolales (*Armatella, Meliola*), Pisorisporiales (*Pisorisporium*), Trichosphaeriales (*Trichosphaeria*). ••••••Xanthopyreniaceae Zahlbr 1926 [=Collemopsidiales Pérez‐Ortega et al. 2016; Collemopsidiomycetes Tedersoo et al. 2018] Thallus comprised of fine hyphae loosely associated with Cyanobacteria and developing ascomata; ascomata perithecioid, solitary, unilocular, with a carbonized to hyaline exciple; branched and anastomosing, often irregularly thick, net‐like physes; asci bitunicate, fissitunicate, with ocular chamber, ovoid to subcylindrical, usually stalked; ascospores hyaline (rarely brownish in mature specimens), oblong to ovoid‐fusiform, one‐septate, with gelatinous perispore usually present; conidiomata pycnidial; conidiogenous cells cylindrical; conidiogenesis phialidic; conidia bacilliform to ellipsoid; lichenized and lichenicolous fungi with crustose, epilithic or endolithic, or lichenicolous forms and Cyanobacteria as photobionts. *Collemopsidium, Xanthopyrenia* ••••••Xylonomycetes R. Gazis & P. Chaverri 2012 Strongly supported as a separate class within the Pezizomycotina (BS: 100%; PP: 1) and contained by the superclass ‘Leotiomyceta’ (BS: 100%; PP: 1) sensu Schoch et al. (2009); based on six loci phylogeny (nucSSU, nucLSU, mitSSU, 5.8S, RBP1 and RPB2). *Xylona*. Contains Symbiotaphrinales, Xylonales. ••••Basidiomycota R. T. Moore 1980 Mycelium present, but some with a yeast state primarily in the Tremellomycetes; basidia produced in a fertile layer with or without fleshy sporocarp; basidia whole or divided longitudinally, typically with four spores per basidium but ranging from one to eight; fusion of compatible mycelia of opposite mating types results in a dikaryotic mycelium in which nuclei of the parent mycelia remain paired but not fused; karyogamy quickly followed by meiosis, one or more mitotic divisions and migration of the nuclei into the developing basidiospores; asexual reproduction may occur through production of conidiospores or via spores produced on basidia from nuclei that have not undergone karyogamy and meiosis (secondary homothallism); cell wall with xylose; septa with swelling near pore; septal pore caps (parenthesomes‐multilayered endoplasmic reticulum) usually present, elaborate in Tremellomycetes; clamp connections present in hyphae or at base of basidia in some groups; mycelial or yeast states; karyogamy typically in probasidium or teliospore, followed by meiosis in a separate compartment (metabasidia), but in some it occurs in the same compartment (holobasidia); holobasidia remain whole or fragment at septation after meiosis (phragmobasidia); metabasidia typically transversely septate with basidiospore borne laterally; cell wall with xylose; parenthesome pore caps absent but with microbodies at septal pores; septal pores occluded by a plug; centrosome multilayered; many are plant pathogens (rusts), animal pathogens, non‐pathogenic endophytes and rhizosphere species. •••••Agaricomycotina Doweld 2001 With a type B secondary structure of the 5S RNA; a cell wall carbohydrate composition with dominance of glucose and presence of xylose. ••••••Agaricomycetes Doweld 2001 Fruiting bodies hymenomycetous or gasteroid; basidia two‐ to eight‐spored; parenthesomes perforate or imperforate. The least inclusive clade containing Agaricomycetidae (Agaricales, Amylocorticiales, Atheliales, Boletales, Jaapiales, Lepidostromatales), Phallomycetidae (Geastrales, Gomphales, Hysterangiales, Phallales). *Incertae sedis* Agaricomycetes: Auriculariales *(Auricularia, Exidia, Hyaloria),* Cantharellales *(Botryobasidium, Cantharellus, Ceratobasidium, Clavulina, Hydnum, Tulasnella),* Corticiales *(Corticium, Punctularia),* Gloeophyllales *(Gloeophyllum),* Hymenochaetales *(Hymenochaete),* Polyporales *(Antrodia, Coriolopsis, Donkiopora, Ganoderma, Lentinus, Phanerochaete, Phlebia, Polyporus, Sparassis, Trametes),* Russulales *(Heterobasidion, Lactarius, Peniophora, Russula),* Sebacinales *(Piriformospora, Sebacina),* Thelephorales *(Hydnellum, Sarcodon),* Trechisporales *(Trechispora)* and Tremellodendropsidales (*Tremellodendropsis*). ••••••Dacrymycetales Hennings 1898 [=Dacrymycetes Doweld 2001] Fruiting bodies gelatinous; basidia furcate, rarely unisporous; parenthesomes imperforate. *Cerinomyces, Dacrymyces*. ••••••Tremellomycetes Doweld 2001 Dimorphic fungi; fruiting bodies gelatinous or absent; basidia septate or nonseptate; parenthesomes sacculate or absent. Containing Cystofilobasidiales (*Cystofilobasidium, Mrakia*), Filobasidiales (*Filobasidium*), Holtermanniales (*Holtermannia*) and Tremellales (*Sirobasidium*,* Syzygospora, Tremella*). •••••Pucciniomycotina R. Bauer et al. 2006 [=Urediniomycetes Swann and Taylor 1995] Mycelial or yeast states; karyogamy typically in probasidium or teliospore, followed by meiosis in a separate compartment (metabasidia), but in some it occurs in the same compartment (holobasidia); holobasidia remain whole or fragment at septation after meiosis (phragmobasidia); metabasidia typically transversely septate with basidiospore borne laterally; cell wall with xylose; parenthesome pore caps absent but with microbodies at septal pores; septal pores occluded by a plug; centrosome multilayered; many are plant pathogens (rusts), animal pathogens, non‐pathogenic endophytes and rhizosphere species. ••••••Agaricostilbomycetes R. Bauer et al. 2006 Dimorphic, nonphytoparasitic, with fucose as cell wall carbohydrate component, septal pores without associated microbodies, aseptate basidiospores during germination and no colacosomes, teliospores, curved holobasidia and radiate conidia; septal pores without microbodies, nucleoplasmic spindle pole body (SPB) separation, metaphasic SPB intranuclear. Containing Agaricostilbales (*Agaricostilbum, Chionosphaera*) and Spiculogloeales (*Mycogloea, Spiculogloea*). ••••••Atractiellales Oberwinkler & Bandoni 1982 [=Atractiellomycetes R. Bauer et al. 2006] With symplechosomes. *Atractiella, Phleogena, Saccoblastia*. ••••••Classiculales R. Bauer et al. 2003 [=Classiculomycetes R. Bauer et al. 2006] With septal pores associated with microbodies and tremelloid haustorial cells. *Classicula, Jaculispora*. ••••••*Cryptomycocolax* Oberwinkler & R. Bauer 1990 [=Cryptomycocolacaceae Oberw. and R. Bauer 1990; =Cryptomycocolacales Oberwinkler and R. Bauer 1990; =Cryptomycocolacomycetes R. Bauer et al. 2006] (M) With microbodies. *Cryptomycocolax abnormis*. ••••••Cystobasidiomycetes R. Bauer et al. 2006 With cell wall carbohydrate composition without fucose; cytoplasmic SPB separation; metaphasic SPBs intranuclear. Containing Cystobasidiales (*Cystobasidium*), Erythrobasidiales (*Bannoa, Erythrobasidium*) and Naohideales (*Naohidea*). ••••••Microbotryomycetes R. Bauer et al. 2006 With colacosomes and septal pores without microbodies; with colacosomes and taxa derived from colacosome fungi; metaphasic SPBs intranuclear. Containing Heterogastridiales (*Heterogastridium*), Leucosporidiales (*Leucosporidiella*,* Mastigobasidium*), Microbotryales (*Microbotryum, Ustilentyloma*) and Sporidiobolales (*Rhodosporidium, Sporidiobolus*). ••••••*Mixia* Kramer 1959 [=Mixiaceae C.L. Kramer 1987; =Mixiales R. Bauer et al. 2006; Mixiomycetes R. Bauer et al. 2006] (M) With multinucleate hyphae and multiple spores produced simultaneously on sporogenous cells. *Mixia osmundae*. ••••••Pucciniomycetes R. Bauer et al. 2006 With a metaphasic intermeiotic SPB duplication. Containing Helicobasidiales (*Helicobasidium*), Pachnocybales (*Pachnocybe*), Platygloeales (*Eocronartium, Platygloea*), Pucciniales (*Coleosporium, Cronartium, Dasyspora, Diorchidium, Melampsora, Mikronegeria, Nyssopsora, Ochropsora, Phakopsora, Phragmidium, Pileolaria, Puccinia, Pucciniastrum, Pucciniosira*) and Septobasidiales (*Auriculoscypha, Septobasidium*). ••••••Spiculogloeaceae Denchev 2009 [=Spiculogloeales R. Bauer et al. 2006; =Spiculogloeomycetes Q.M. Wang et al. 2015] Characterized by teleomorphic members that may form tremelloid haustorial cells (nanometre‐fusion mycoparasitism). *Spiculogloea*. ••••••*Tritirachium* Limber 1940 [=Tritirachiaceae Aime and Schell 2011; =Tritirachiales Aime and Schell 2011; =Tritirachiomycetes Aime and Schell 2011] (M) With multinucleate hyphae, simple pore septa, conidiophores that are subhyaline to dematiaceous and subhyaline to dematiaceous sympodial conidiogenous cells that occur in whorls and bear conidia on an elongated rachis; teleomorph not known. *Tritirachium dependens* •••••Ustilaginomycotina R. Bauer et al. 2006 Mycelial in the parasitic phase, and many with saprobic yeast or ballisticonidial states; plant parasites causing rusts and smuts; meiospores produced on septate or aseptate basidia; cell wall carbohydrates dominated by glucose; xylose absent; parenthesomes absent at septal pores; swellings absent at septal pores except in *Tilletia*; centrosomes globose, unlayered. ••••••Exobasidiomycetes Begerow et al. 2007 With local interaction zones and no intracellular hyphal coils. Containing Ceraceosorales (*Ceraceosorus*), Doassansiales (Do*assansia, Melaniella, Rhamphospora*), Entylomatales (*Entyloma*), Exobasidiales (*Kordyana, Laurobasidium, Exobasidium, Graphiola*), Georgefischereriales (*Eballistra, Georgefischereria, Gjaerumia, Tilletiaria*), Golubeviales *(Golubevia*), Microstromatales (*Microstroma, Quambalaria, Volvocisporium*) Robbauerales (*Robbauera*), Tilletiales (*Tilletia*). ••••••*Malassezia* Baill 1889 [=Malasseziales R.T. Moore 1980; =Malasseziaceae Denchev and R.T. Moore 2009; =Malasseziomycetes Boekhout et al. 2014] (M) Cells globose, ovoid or cylindrical; budding typically monopolar on a more or less broad base, enteroblastic and percurrent; cell wall multilamellate, and the inner layer of the cell wall corrugated with a groove spiralling from the bud site; lipid dependent or lipophilic; sugars are not fermented, urease and diazonium blue B (DBB) reactions are positive; coenzyme Q‐9 is formed; xylose absent in whole‐cell hydrolysates; sexual morph unknown. *Malassezia furfur*. ••••••*Moniliella* Stolk & Dakin 1966 [=Moniliellaceae Q.M. Wang et al. 2014; =Moniliellales Q.M. Wang et al. 2014; =Monilielliomycetes Q.M. Wang et al. 2014] (M) Colonies are smooth or velvety, greyish to olivaceous black; budding cells are ellipsoidal and form terminally on true hyphae that disarticulate with artroconidia; pseudohyphae and chlamydospores may be present; cell walls are multilamellar; hyphal septa typically possess dolipores with an arch of endoplasmic reticulum, but ‘micropore’‐like structures may also be present; sugars are fermented by most species; nitrate is assimilated; urease and diazonium blue B (DBB) reactions are positive; coenzyme Q‐9 is present; xylose and fucose absent from whole‐cell hydrolysates; sexual morph unknown. *Moniliella acetoabutans*. ••••••Ustilaginomycetes R. Bauer et al. 1997 With enlarged interaction zones. Containing Urocystales (*Doassansiopsis, Floromyces, Thecaphora, Melanotaenium, Urocystis*) and Ustilaginales (*Anthracoidea, Ustanciosporium, Sporisorium, Ustilago*). •••••Wallemiomycotina Doweld 2014 Conidia arthrospore‐like, verruculose, short cylindrical, becoming spherical; conidiophores unbranched or sympodially proliferating, continuous with conidiogenous cells, smooth; conidiogenesis basauxic; hyphal septa with a single pore, flaring out near the periphery of the pore, barrel‐shaped, dolipore‐like. ••••••*Wallemia* Johan‐Olsen 1887 [=Wallemiaceae R.T. Moore 1996; =Wallemiales Zalar et al. 2005; =Wallemiomycetes Zalar et al. 2005] (M) Xerophilic; produce basauxic anamorphs and do not produce basidiomata in culture; with dolipore septa with adseptal tubular extensions that arise from sheets of endoplasmic reticulum that form the septal pore cap; septal pore cap sometimes absent; septal pore has an electron‐dense non‐membranous septal pore occlusion and striations that are oriented vertically. *Wallemia ichthyophaga*. ••••••Geminibasidiaceae H.D.T. Nguyen et al. 2013 [=Geminibasidiales H.D.T. Nguyen et al. 2013; =Geminibasidiomycetes H.D.T. Nguyen and Seifert 2015] Xerotolerant; basidiomata not produced in culture; basidia arising from somatic hyphae or from swollen basidium‐bearing cells (primary cells) with a basal lateral projection occurring either on the basidium or the swollen primary cell; basidiospores symmetrical on sterigma, not forcibly discharged, and brown at maturity; arthroconidial and/or yeast‐like asexual morphs sometimes produced; species have a dolipore septum that is electron‐dense at the pore swelling with an electron‐dense membranous septal pore occlusion; some species are heat resistant. *Geminibasidium*. •••Monoblepharidomycetes J.H. Schaffn. 1909 Thallus epibiotic, filamentous (hyphal or rhizomycelial), either extensive or a simple unbranched thallus, often with a basal holdfast. Zoospores oval possessing a non‐ciliated centriole parallel to the ciliated kinetosome with a striated disc partially extending around the kinetosome; microtubules radiating anteriorly from the striated disc; ribosomal aggregation around the nucleus; fenestrated cisterna (=rumposome) adjacent to the microbody in the MLC; Golgi apparatus with stacked cisternae; nuclear envelope fenestrated at poles during mitosis; aerobic and anaerobic; asexual reproduction occurs via production of posteriorly uniciliated cells or autospores while sexual reproduction is oogamous via fusion of uniciliated antherozoids produced in antheridia and non‐ciliated female gametes produced within oogonia. *Gonapodya, Harpochytrium, Hyaloraphidium, Monoblepharella, Monoblepharis, Oedogoniomyces, Telasphaerula*. •••Mucoromycota Doweld 2001, emend. Spatafora and Stajich 2016 [Zygomycota F. Moreau 1954, pro parte] (R) The least inclusive clade containing Mucoromycotina, Mortierellomycotina, and Glomeromycotina. Characters associated with sexual reproductive states, where known, include zygospore production by gametangial conjugation. Asexual reproductive states can involve chlamydospores and spores produced in sporangia and sporangioles. ••••Glomeromycotina C. Walker & A. Schüßler 2016 Filamentous; primarily endomycorrhizal, forming arbuscules in roots, sometimes with vesicles; without cilium; presumed asexual spores outside or within roots of host; some complex spores with multiple wall groupings, others simple (blastic chlamydospores); without centrioles, conidia, and airborne spores. •••••Archaeosporales C. Walker & A. Schüßler 2001 [=Archaeosporomycetes Sieverding et al. 2011] Known to form symbiosis with plant roots or thalli, or with cyanobacteria; if symbiosis occurs between plants and fungi, fungal spores may have two morphs, but often only one is known; species form vesicular‐arbuscular or arbuscular mycorrhiza. *Archaeospora, Ambispora, Geosiphon*. •••••Glomeromycetes Cavalier‐Smith 1998, emend. Oehl et al. 2011 Glomoid chlamydospores formed terminally, subterminally or intercalarily in hyphae, either in or on the surface of soils or sometimes in roots, either singly, in spore clusters or multiple‐spored loose to compact sporocarps, on subtending hyphae: complex multiwalled spores on sporogenous structures, or laterally or centrally within a sporiferous saccule or intrahyphally in the stalk of sporiferous saccules, forming arbuscular or vesicular‐arbuscular mycorrhiza. ••••••Diversisporales C. Walker & A. Schüßler 2001 Spore formation by blastic expansion of hypha (chlamydosporic), or sometimes with complex spores with up to three walls or wall groups: multiple layered outer wall, and hyaline middle and inner walls that may be of several components or layers; spores with subtending hyphae, sometimes with a conspicuous colour change distant to the septum most proximal to the spore base; spores with 1–3 wall layers; pore rarely open. *Acaulospora, Diversispora, Gigaspora, Pacispora, Racocetra, Scutellospora*. ••••••Glomerales J. B. Morton & Benny 1990 Spores by blastic expansion of the hyphal tip or intercalarily formed in hyphae, either in soils or occasionally in roots, or other subterranean structures such as rhizomes, either singly, in spore clusters or multiple‐spored; sporocarps loose to compact, with a mono‐ to multiple‐layered spore wall; wall of subtending hyphae continuous with the spore wall and coloured the same as or slightly lighter than it or hyaline to subhyaline; subtending hyphae funnel‐shaped, cylindrical or constricted; forming arbuscular mycorrhiza. *Claroideoglomus, Funneliformis, Glomus, Rhizophagus, Sclerocystis, Septoglomus*. ••••••*Paraglomus* J.B. Morton & D. Redecker 2001 [=Paraglomeraceae J. B. Morton and D. Redecker 2001; =Paraglomerales C. Walker and A. Schüßler 2001; =Paraglomeromycetes Oehl et al. 2011] (M) Endomycorrhizal, forming arbuscular mycorrhiza; asexual spores (chlamydospores) usually formed in soil, sometimes within roots or other host tissue, sometimes with vesicles; without cilium; without centrioles, conidia and aerial spores. *Paraglomus occultum*. ••••Mortierellaceae A. Fischer 1892 [Mortierellales Cavalier‐Smith 1998; =Mortierellomycotina Kerst et al. 2011; =Mortierellomycetes Doweld 2014; =Mortierellomycota Tedersoo et al. 2018] Mycelium with anastomosing hyphae, dichotomously branching, bearing stylospores; hyphae sporangiferous, sporangiophores basally inflated and elongating towards the sporangiophore apex, erect, coenocytic initially, but irregularily septated at maturity; asexual reproduction via sporangia and sporangiola; sporangia spherical, multispored; columella absent; ramifications gracilous, primarily horizontally expanding, erecting hyphae sometimes terminate with sporangiola; spores globose to ellipsoid or irregular, smooth or ornamented; rhizoids only occasional; giant cells absent; zygospores naked. *Mortierella*. ••••Mucoromycotina Benny 2007 Saprobes, or rarely gall‐forming, non‐haustorial, facultative mycoparasites or forming ectomycorrhiza; mycelium branched, coenocytic when young, sometimes producing septa that contain micropores at maturity; asexual reproduction by sporangia, sporangiola or merosporangia, or rarely by chlamydospores, arthrospores or blastospores; sexual reproduction by more or less globose zygospores formed on opposed or apposed suspensors. •••••Endogonales Moreau ex R. K. Benjamin 1979 [=Endogonomycetes Doweld 2014] Filamentous, hyphae coenocytic; saprobic and ectomycorrhizal; zygospores with apposed suspensors produced in a subterranean sporocarp. *Endogone, Sphaerocreas*. •••••Mucorales Fritz 1832, emend. Schröter 1897 [=Mucoromycetes Doweld 2014] (P) Filamentous, generally saprotrophic, with exceptions; septa absent except in older hyphae; with plasmodesmata at septal pores; asexual reproduction with one to many spores in merosporangia, sporangiola or sporangium; reproduction by zygospore, typically with opposed suspensors. Traditional subdivisions artificial. *Backusella*,* Chaetocladium*,* Choanephora*,* Cunninghamella, Lentamyces*,* Lichtheimia*,* Mucor*,* Mycotypha*,* Phycomyces*,* Pilobolus*,* Radiomyces*,* Saksenaea*,* Syncephalestrum*. •••••Umbelopsidales Spatafora, Stajich and Bonito 2016 [=Umbelopsidomycetes Tedersoo et al. 2018] The least inclusive clade containing the genus *Umbelopsis*. Asexual reproduction by sporangia and chlamydospores. Sporangiophores may be branched in a cymose or verticillate fashion. Sporangia are typically pigmented red or ochre, multi‐ or single‐spored and with or without conspicuous columella. Sporangiospores are globose, ellipsoidal or polyhedral and pigmented like sporangia. Chlamydospores are filled with oil globules and often abundant in culture. Sexual reproduction is unknown. *Umbelopsis*. •••Neocallimastigaceae Heath 1983, emend. Barr 1989 [;=Neocallimastigales J. L. Li et al. 1993, =Neocallimastigomycetes M. J. Powell 2007, = Neocallimastigomycota M. J. Powell 2007] Thallus monocentric or polycentric; anaerobic fermentative, found in digestive system of larger herbivorous mammals and possibly in other terrestrial and aquatic anaerobic environments; asexual reproduction; mitochondria absent; hydrogenosomes of mitochondrial origin; uni‐ and multiciliated zoospores with a kinetosome‐associated complex that includes a skirt, strut, spur and circumary ring, microtubules stretching from the spur and radiating around the nucleus, forming a posterior fan; unikont kinetid and without props; nuclear envelope is retained during mitosis. *Anaeromyces, Buwchfawromyces, Caecomyces, Cyllamyces, Feramyces, Neocallimastix, Oontomyces, Orpinomyces, Pecoramyces, Piromyces*. •••*Olpidium* (A. Braun) Rabenh. 1868 [=Olpidiaceae J. Schröt 1889; =Olpidiales Cavalier‐Smith 2013; Olpidiomycota Doweld 2013; =Olpidiomycotina Doweld 2013; =Olpidiomyceta Tedersoo et al. 2018] (M) Thallus monocentric, holocarpic or eucarpic, with no hyphae; zoospores posterior, uniciliate, generally with a single globule, cone‐shaped striated rhizoplast fused to both the functional and vestigial kinetosomes, gamma‐like particles and rough endoplasmic reticulum; sporangium single, endobiotic; nucleus associated with the basal body, no nuclear cap; two parallel centrioles linked to nucleus by shared, tapering, striated rhizoplast; no root microtubules or dictyosome; side‐body complex lacking; pathogens of terrestrial plants. *Olpidium brassicae*. •••Zoopagomycota Gryganskyi, M.E. Smith, Spatafora & Stajich 2016 [Zygomycota F. Moreau 1954, pro parte] The least inclusive clade containing Entomophthoromycotina, Kickxellomycotina and Zoopagales. Sexual reproduction, where known, involves the production of zygospores by gametangial conjugation. Morphologies associated with asexual reproductive states include sporangia, merosporangia, conidia and chlamydospores. ••••Entomophthoromycotina Humber 2007 Obligate pathogens of animals (primarily arthropods), cryptogamic plants, or saprobes; occasionally facultative parasites of vertebrates. Somatic state consisting of a well‐defined mycelium, coenocytic or septate, walled or protoplastic, which may fragment to form multinucleate hyphal bodies; protoplasts either hyphoid or amoeboid and changeable in shape; cystidia or rhizoids formed by some taxa. Conidiophores branched or unbranched. Primary spores true conidia, uni‐, pluri‐ or multinucleate, forcibly discharged by diverse possible means or passively dispersed; secondary conidia often produced. Resting spores with thick bilayered walls form as zygospores after conjugations of undifferentiated gametangia from different or the same hyphal bodies or hypha or as azygospores arising without prior gametangial conjugations. •••••*Basidiobolus* Eidam 1886 [=Basidiobolomycetes Doweld 2001 emend. Humber 2012] (M) Differs from Entomophthoromycetes and Neozygitomycetes by unusually large nuclei (often ≥10 μm long) with a large central nucleolus that is the major feature of uninucleate cells. Mitoses involve barrel‐shaped spindles, mitotic organelles incorporating microtubules (but not centrioles) but not always located at the spindle poles, and the nuclear content isolated from the cytoplasm by a layer of nuclear and cytoplasmic membrane fragments. *Basidiobolus ranarum*. •••••Entomophthorales G. Winter 1880 [=Entomophthoromycetes Humber 2012] Filamentous, primarily without septa; mostly parasites of insects, mites, and spiders; sexual reproduction by thick‐walled zygospore, strictly homothallic, where known; asexual reproduction by conidia formed by blastosporogenesis; conidia forcibly discharged and often form secondary conidia. *Ancylistes, Completoria, Entomophthora, Meristacrum*. •••••*Neozygitaceae* Ben‐Ze'ev, R.G. Kenneth and Uziel 1987 [=Neozygitomycetes Humber 2012;= Neozygitales Humber 2012] Differs from Basidiobolomycetes and Entomophthoromycetes by vermiform, moderately sized chromosomes that condense during mitosis on a central metaphase plate but uncoil during interphase. Nuclear numbers in vegetative cells and conidia are low and apparently controlled at (3)‐4‐(5). *Neozygites*. ••••Zoopagales Bessey ex R.K. Benjamin 1979 [=Zoopagomycotina Benny 2007] Filamentous, hyphae coenocytic or septate; parasites of soil fungi, invertebrates, and amoebae; asexual reproduction by conidia or merosporangia; sexual reproduction by globose zygospores with apposed suspensors. *Amoebophilus, Piptocephalis, Rhopalomyces, Sigmoideomyces, Stylopage*. ••••Kickxellomycotina Benny 2007 Saprobes, mycoparasites or obligate symbionts; thallus arising from a holdfast on other fungi as a haustorial parasite, or branched, septate, subaerial hyphae; mycelium branched or unbranched, regularly septate; septa with median, disciform cavities containing plugs; asexual production by one‐ or two‐spored merosporangia, trichospores or arthrospores; sexual reproduction by zygospores that are globose, biconical or allantoid and coiled. •••••Asellariales Manier ex Manier & Lichtwardt 1978 [=Asellariaceae Manier ex Manier and Lichtw 1968] Kickxellomycotina with filamentous, branched thalli; asexual reproduction by arthrospore‐like cells that disarticulate from the corresponding thallus; in the digestive tracts of terrestrial, aquatic and marine isopods, as well as springtails. *Asellaria, Baltomyces, Orchesellaria*. •••••Dimargaritaceae R.K. Benjamin 1959 [=Dimargaritales R. K. Benjamin 1979] Hyphae regularly septate; septa containing a lenticular cavity; asexual reproduction by bisporous merosporangia; sexual reproduction by a zygospore, often ornamented; obligate haustorial parasites of fungi, especially Mucorales. *Dimargaris, Dispira, Spinalia, Tieghemiomyces*. •••••Harpellales Lichtwardt & Manier 1978 Endosymbionts of freshwater arthropods with basal cell attached to the host, from which a filamentous thallus develops; hyphae septate, with or without branching; septa contain a lenticular cavity; asexual reproduction occurs by lateral elongate monosporous trichospores; sexual reproduction by conical or biconical zygospores. Note that this group includes taxa previously referred to as trichomycetes. *Harpella, Orphella, Smittium, Zygopolaris*. •••••Kickxellaceae Linder 1943 [=Kickxellales Kreisel ex R. K. Benjamin 1979] Filamentous; hyphae possessing septa with a lenticular cavity; asexual reproduction by unispored sporangiola (merosporangia) produced on a sporocladium; saprobic or mycoparasitic, isolated from soil and dung. *Coemansia, Dipsacomyes, Kickxella, Linderina, Martensella, Martensiomyces, Spirodactylon, Spiromyces*. **DIAPHORETICKES Adl et al.** **2012** **;** *Incertae sedis* Diaphoretickes: *Microheliella* Cavalier‐Smith 2012 (M) Aciliated, central centrosome with two concentric shells of dense material and a dense central core; axopodia supported by triads of microtubules, bearing ellipsoid extrusomes; single nucleus eccentric, with internal channels where microtubular triads pass; mitochondrial cristae tubular; cortical filogranular network. *Microheliella maris*. *Ancoracysta* Janouskovec et al. 2017 (M) Small ovoid marine cytotrophic predators on small protists; cell covered by a theca and dense glycocalyx; one pair of heterodynamic cilia; anterior cilium with mastigonemes at its anterior; posterior cilium with short vane, and traverses cell along a groove; mitochondria with lamellar cristae; characteristic extrusome (ancoracyst). *Ancoracysta twista*. Rappemonads Kim et al. 2011 Marine and fresh water 5–7 µm cells with 2–4 plastids containing chlorophyll *a*. Poorly characterized, rare low abundance organisms, known only from environmental samples, and no species or genera described. Telonemia Shalchian‐Tabrizi 2006 Biciliated cells with a proboscis‐like structure located at the ciliary pole and a complex cytoskeleton composed of layers of microtubules and microfilaments; tripartite tubular hairs on the long cilium; mitochondria with tubular cristae; peripheral vacuoles located just beneath the cell membrane; chloroplasts not observed. *Telonema* Griessmann 1913, may consist of several genera. Picozoa Seenivasan et al. 2013 [Picobiliphytes Not et al. 2007] (M) Cell oblong separated in two by a cleft; of picoplanktonic size; marine; without plastid; cytotrophic; two cilia inserted laterally; mitochondrion with tubular cristate; heterotrophic, possibly feeding on small viruses or colloidal particles. *Picomonas judraskeda*. **Archaeplastida Adl et al.** **2005** Photosynthetic plastid with chlorophyll type‐*a* from an ancestral primary endosymbiosis with a cyanobacterium; plastid with two membranes without periplastid endoplasmic reticulum; plastid reduced in some; usually with cell wall or other extracellular covering; flat mitochondrial cristae; starch storage product.
•**Glaucophyta** Skuja 1954 [Glaucocystaceae West 1904 Glaucocystophyta Kies and Kremer 1986] Unicellular or colonial algae; plastid in the form of a cyanelle, which is distinct from the chloroplasts of other organisms in that, like cyanobacteria, it has a conspicuous peptidoglycan wall between its two membranes; chlorophyll type‐*a* only, with phycobiliproteins and other pigments; ciliated and non‐ciliated species or life cycle stages; without cellulosic cell wall except *Glaucocystis*. Reported only in freshwater. *Cyanophora*,* Cyanoptyche*,* Glaucocystis*,* Gloeochaete*. •**Rhodophyceae** Thuret 1855, emend. Rabenhorst 1863 [Rhodophyta Wettstein 1901, Rhodoplantae Saunders and Hommersand 2004] emend. Adl et al. 2005 Red algae without ciliated stages, and without centrioles, or basal bodies, or other 9 + 2 microtubular structures—presence of polar rings instead; two‐membraned simple chloroplasts, unstacked thylakoids with phycobilisomes, and chlorophyll *a* only, lacking external endoplasmic reticulum; cytoplasmic carbohydrate reserve floridean starch; chromosomal and interzonal microtubules not converging towards polar rings, so spindle poles very broad; telophase spindle and nuclear envelope persisting with closed mitosis surrounded by perinuclear endoplasmic reticulum; cell wall of cellulose; cells in filamentous forms linked by pit plugs, formed between cells after incomplete cell division; sexual reproduction typically oogamous; triphasic life history common. ••Cyanidiales T. Christensen 1962 [Cyanidiophyceae Merola et al. 1981, Cyanidiophyta Moehn ex Doweld 2001] Unicellular, spherical or elliptical in shape; thick cell wall or lack of cell wall; facultative heterotrophs or obligate photoautotrophs; cell division or endospore formation; inhabiting acidic and high temperature environments. *Cyanidioschyzon*,* Cyanidium*,* Galdieria*. ••Proteorhodophytina Muñoz‐Gómez et al. 2017 (R) Clade consisting of Compsopogonales, Porphyridiophyceae, Rhodellophyceae, Stylonematales, based on phylogenetic analysis. •••Compsopogonales Skuja 1939 [Compsopogonophyceae G. W. Saunders and Hommersand 2004] Pluricellular with monosporangia and spermatangia usually cut out by curved walls from ordinary vegetative cells; Golgi–ER association; encircling thylakoids in the plastid; life history biphasic if known; inhabiting freshwater or marine environment. *Boldia, Compsopogon*,* Erythrotrichia*,* Rhodochaete*. •••Porphyridiophyceae H. S. Yoon et al. 2006 Unicellular with a single branched or stellate plastid, with or without pyrenoid; Golgi association with mitochondria and ER; cells with floridoside as a low molecular‐weight carbohydrate; reproduction by cell division; inhabiting marine, freshwater and even moist terrestrial areas. *Erythrolobus*,* Flintiella*,* Porphyridium*. •••Rhodellophyceae Cavalier‐Smith 1998 [Rhodellophytina Cavalier‐Smith 1998] Unicellular; single highly lobed plastid with eccentric or centric pyrenoid; Golgi association with nucleus and ER; contains mannitol; reproduction by cell division; inhabiting marine and freshwater habitats. *Dixoniella*,* Glaucosphaera*,* Rhodella*. •••Stylonematales K. Drew 1956 [Stylonematophyceae H.S. Yoon et al. 2006] Unicellular or pseudofilamentous or filamentous; various plastid morphologies with or without pyrenoid; Golgi association with mitochondria and ER; reproduction by cell division or monospores; inhabiting freshwater, brackish and marine environment. *Bangiopsis*,* Chroodactylon*,* Chroothece*,* Purpureofilum*,* Rhodosorus*,* Rhodospora*,* Rufusia*,* Stylonema*. ••Eurhodophytina G.W Saunders & Hommersand 2004 (R) Clade containing Bangiales and Florideophycidae, based on phylogenetic analysis. •••Bangiales Nägeli 1847 [Bangiophyceae A. Wettstein 1901] Pluricellular with Golgi–ER/mitochondrion association; life history biphasic, heteromorphic, gametophyte macroscopic, initially uniseriate, becoming pluriseriate or foliose by diffuse growth; carposporangia and spermatangia produced in packets by successive perpendicular divisions; sporophyte filamentous, with pit plugs with a single cap layer, but lacking membranes; typically forming conchospores in fertile cell rows; inhabiting mostly marine environment. *Bangia*,* Bangiomorpha*,* Boreophyllum*,* Dione*,* Minerva*,* Porphyra*,* Pyropia*,* Pseudobangia*. •••Florideophycidae Cronquist 1960 Pluricellular with Golgi–ER/mitochondrion; growth by means of apical cells and lateral initials forming branched filaments in which the cells are linked throughout by pit connections; life history fundamentally triphasic consisting of gametophytic, carposporophytic and tetrasporophytic phases; reproductive cells (monosporangia, spermatangia, carposporangia, tetrasporangia) generally terminal or lateral on the filaments; carpogonia terminal or lateral, bearing an apical extension, the trichogyne, to which the spermatangia attach; carposporophyte developing directly from the carpogonium or its derivative; inhabiting mostly marine environment. ••••Hildenbrandiaceae Rabenhorst 1868 [Hildenbrandiophycidae G. W. Saunders and Hommersand 2004] Pluricellular that are crustose and smooth to tubercular or with erect branches; composed of a basal layer of laterally adhering branched filaments and laterally adhering simple or branched erect filaments; pit plugs with a single cap layer and membrane; tetrasporangia zonately or irregularly divided, apomeiotic, borne in ostiolate conceptacles; sexual reproduction unknown. *Apophloea*,* Hildenbrandia*. ••••Nemaliophycidae Christensen 1978 Pluricellular; pit plugs characterized by two cap layers. *Acrochaetium*,* Balbiania*,* Ballia*,* Batrachospermum*,* Colaconema*,* Entwisleia*,* Nemalion*,* Palmaria*,* Rhodychlya*,* Thorea*. ••••Corallinophycidae L. Le Gall & G. W. Saunders 2007 Pluricellular; carpogonial branches two‐celled; tetrasporangia zonate or cruciate in division; pit plug with two cap layers at cytoplasmic faces, outer dome shaped, membrane absent; calcification in the form of calcite. *Corallina*,* Harveylithon*,* Hydrolithon*,* Lithophyllum*,* Mastophora*,* Melobesia*,* Metagoniolithon*,* Neogoniolithon*,* Porolithon*,* Rhodogorgon*,* Sporolithon*. ••••Ahnfeltiophycidae G. W. Saunders & Hommersand 2004 Pluricellular; carpogonia terminal and sessile; carposporophyte developing outward; pit plugs naked, lacking caps and membranes. *Ahnfeltia*,* Pihiella*. ••••Rhodymeniophycidae G. W. Saunders & Hommersand 2004 Pluricellular with sexual life histories generally triphasic; carposporophyte developing directly from the carpogonium or carpogonial fusion cell, or indirectly from an auxiliary cell that has received the postfertilization diploid nucleus; pit plugs with membranes only (single inner cap in Gelidiales). *Acrosymphytum*,* Bonnemaisonia*,* Ceramium*,* Gelidium*,* Gigartina*,* Gracilaria*,* Halymenia*,* Nemastoma*,* Peyssonnelia*,* Plocamium*,* Rhodymenia*,* Sebdenia*. •**Chloroplastida** Adl et al. 2005 [Viridiplantae Cavalier‐Smith 1981; Chlorobionta Jeffrey 1982, emend. Bremer 1985, emend. Lewis and McCourt 2004; Chlorobiota Kendrick and Crane 1997] Plastid with two membranes without periplastid endoplasmic reticulum; plastid with chlorophyll *a* and *b*; starch inside plastid; cell wall often with cellulose, or scaly extracellular covering; swimming cells with cilia in multiples of two, or rarely single cilium, with stellate structure linking nine pairs of microtubules at basal body transition zone; with centrioles; Rubisco small subunits encoded in the nuclear genome. ••Chlorophyta Pascher 1914, emend. Lewis & McCourt 2004 Plastid thylakoids single or stacked; glycolate dehydrogenase present; cell division without phragmoplast. •••Ulvophyceae Mattox & Stewart 1984 (P) Swimming cells with one or two pairs of cilia, without mastigonemes; basal bodies with four microtubular rootlets in cruciate arrangement, and smaller roots of two sizes, alternating between two and more microtubules; cilia with scales and rhizoplasts; cell wall more or less calcified; cell division by furrowing with mitotic spindle closed, centric and persistent; phycoplast absent; thallus can be branched or unbranched, mono‐ or distromatic sheet (phyllose), or cushiony forms of compacted tubes; thallus often multinucleate and siphonous; free‐living diplobiontic life cycle, iso‐ or heteromorphic. *Acetabularia, Caulerpa, Chladophora, Codium, Pithophora, Pseudonochloris, Rhizoclonium*. Note: The inclusion of *Oltmannsiellopsis* in Ulvophyceae causes instability in phylogenies, and the monophyly is questioned. •••Trebouxiophyceae Friedl 1995 [Pleurastrophyceae Mattox et al. 1984, Microthamniales Melkonian 1990] (P?) Swimming cells with one or two pairs of cilia, without mastigonemes; basal bodies with four microtubular rootlets in cruciate arrangement, including a multilayered structure, and asmaller root, alternating between two and more microtubules; basal bodies with prominentrhizoplast, cruciate, displaced counterclockwise and counterclockwise basal body orientation; closed mitosis with metacentric spindle, semi‐closed mitosis; cytokinesis with phycoplast; asexual reproduction by autospores or zoospores; sexual reproduction reported but not observed; lichenose and free‐living forms; most with cell walls; osmotrophy and autotrophy. *Botryococcus, Chlorella, Choricystis, Coccomyxa, Microthamnion, Nannochloris, Oocystis, Pabia, Prasiola, Prototheca, Trebouxia* (P). •••Chlorophyceae Christensen 1994 Swimming cells with one to hundreds of cilia, without mastigonemes; when two or four cilia, basal bodies with four microtubular rootlets in cruciate arrangement, alternating between two and more microtubules; basal bodies displaced clockwise or directly opposed; rhizoplast connects basal bodies and extends to nucleus; in colonial forms, basal bodies reoriented to face outside of colony; closed mitosis; cytokinesis has phycoplast with microtubules, sometimes with furrowing, with formation of plasmodesmata cell‐cell connections; haplobiontic life cycle; sexual reproduction by isogamy, anisogamy, or oogamy; asexual reproduction by aplanospores, akinetes, or autosporic; osmotrophy and autotrophy. *Bracteacoccus, Chlamydomonas (P), Desmodesmus, Floydiella, Hydrodictyon, Oedegonium, Pediastrum, Scenedesmus, Volvox*. Incertae sedis Chlorophyceae: *Carteria, Cylindrocapsa, Hafniomonas, Mychanastes, Treubaria, Trochiscia*. •••Chlorodendrophyceae Fritsch 1917 Cells with a pair of cilia, inserted in a ciliary pit; cilia beat in breast‐stroke pattern; basal body rootlets structure in X2X2 configuration; with organic extracellular scales, outer layer of scales fused to form a theca; metacentric spindle collapses at telophase; nutrition by autotrophy and osmotrophy. Treated as prasinophyte clade IV (Nakayamaet al. 1998). *Scherffelia, Tetraselmis*. •••Pedinophyceae Moestrup 1991, emend. Fawley et al. in Adl et al. 2012 Unicellular, with single cilium; closed mitosis with persistent spindle; phycoplast absent; counterclockwise basal body orientation; cilia covered with rigid or thin, hair‐like appendages; single parietal chloroplast. *Marsupiomonas, Pedinomonas*. •••Chloropicophyceae Lopes dos Santos & Eikrem 2017 Small coccoid cells with a diameter of 1.5–4 μm; pyrenoid absent; without cilium; with layered cell wall; marine. Treated as prasinophyte clade VII A & B (Lopes dos Santos et al. 2017). *Chloroparvula, Chloropicon*. •••Picocystophyceae Lopes dos Santos & Eikrem 2017 Coccoid, but ovoid or tri‐lobed forms observed in old cultures; layered cell wall containing polyarabinose, mannose, galactose and glucose. Treated as prasinophyte clade VII C (Lopes dos Santos et al. 2017). *Picocystis*. •••Pyramimonadales Chadefaud 1950 Swimming cells with 4–16 cillia; helical structure in the cilliary transitional region; trailing cells bearing a single cillum observed in some, which may represent gametes; chloroplast cup‐shaped; pyrenoid present; some with eyespot(s); several layers of body and cilliary scales; some producing conspicuous cyst (called phycoma); with one or more large vacuoles and associated duct system; phagotrophy in some. Treated as prasinophyte clade I (Nakayama et al. 1998). *Cymbomonas, Halosphaera, Pterosperma, Pyramimonas*. •••Mamiellophyceae Marin & Melkonian 2010 Cells typically solitary, with single chloroplast, sometimes two; prasinoxanthin commonly present; two cilia, single or no cilium present; cilia equal or unequal in length; eye spot posterior if present; cells and/or cilia with 1–2 layers of flattened, rounded or elliptical scales, or scales absent; scales ornamented with spider web‐like or uniformly reticulate pattern; mostly marine, some freshwater. Treated as prasinophyte clade II (Nakayamaet al. 1998). *Bathycoccus, Crustomastix, Dolichomastix, Mamiella, Monomastix, Mantoniella*. •••*Nephroselmis* [Nephroselmidophyceae Cavalier‐Smith 1993, emend.Yamaguchi 2011] Cells laterally compressed; two cilia inserted laterally; square‐ or diamond‐shaped scales cover cell body and cilia, except in at least one species—*Nephroselmis pyriformis*; single, cup‐shaped chloroplast with pyrenoid and eyespot; contractile vacuole near ciliary bases in freshwater species; sexual reproduction by hologamy; mostly marine, some freshwater. Treated as prasinophyte clade III (Nakayama et al. 1998). *Nephroselmis*. •••Pycnococcaceae Guillard 1991, emend. Fawley 1999 [Pseudoscourfieldiales and Pseudoscourfieldiaceae Melkonian 1990] Plastid with prasinoxanthin; with or without scaly covering; vegetative cells with two cilia or no cilia; trailing cells bearing a single cilium rarely observed in *Pycnococcus* culture; pyrenoid in the plastid; intrusion of mitochondrial membranes into the pyrenoid. Treated as prasinophyte clade V (Fawley et al. 2000). *Pycnococcus, Pseudoscourfieldia*. •••Palmophyllophyceae Leliaert et al. 2016 Marine; solitary, in loose colonies, or cells grouped in gelatinous matrix; strongly supported in plastid multigene and nuclear ribosomal DNA phylogenies. ••••Palmophyllales Zechman et al. 2010 Thallus macroscopic, crustose or erect; subspherical cells in gelatinous matrix make up thallus; cell diameter 6–10 μm; each cell with single cup‐shaped chloroplast lacking pyrenoids; benthic marine. *Palmophyllum*,* Palmoclathrus, Verdigellas*. ••••Prasinococcales Guillou et al. 2004, as in Leliaert et al. 2016 Marine; planktonic; solitary or forming loose colonies; no cilium; without scaly covering; with cell wall; chloroplast cup‐shaped and with pyrenoid; cell division by unequal binary fission in which one of the daughter cells retains the parent wall, while the other is released with a newly produced cell wall. Treated as prasinophyte clade VI (Fawley et al. 2000). *Prasinococcus, Prasinoderma*. ••Streptophyta Bremer & Wanntorp 1981 [Charophyta Migula 1897, emend. Karol et al. 2009; Charophyceae Smith 1938; Jeffrey 1967; Streptophyta, Mattox and Stewart 1984] Asymmetric motile cells, when present, with pair of cilia without mastigonemes; basal bodies with distinctive multilayered structure of microtubular rootlet and cytoskeletal anchor; thylakoids stacked; open mitosis; usually with phycoplast,but some with phragmoplast and cell plate; with primary plasmodesmata between adjacent cells in filamentous forms; filaments branching or nonbranching; with nonmotile vegetative phase; some with multinucleate cells; with or without sexual reproduction; sexual species with haplobiontic life cycle; with desiccation‐resistant cysts (zygospores); glycolate oxidase in peroxisomes; Cu/Zn superoxide dismutase; ciliary peroxisome. •••*Chlorokybus* Geitler 1942 [Chlorokybophyceae Lewis and McCourt 2004] (M) Sarcinoid packets of cells; subaerial; biciliated zoospores; cilia with hairs; multilayered structure (MLS) at ciliary root. *Chlorokybus atmophyticus*. •••*Mesostigma* Lauterborn 1894 [Mesostigmatophyceae Marin and Melkonian 1999, emend. Lewis and McCourt 2004; Mesostigmata Turmel et al. 2002] (M) Asymmetrical cell with pair of lateral cilia without mastigonemes, emerging from a pit; presence of multilayered structures adjacent to the ciliary basal bodies; with glycolateoxidase; ciliary peroxisome present; cell wall of cellulose; organic scales cover cell wall and cilia. *Mesostigma viride*. •••Klebsormidiophyceae van den Hoek et al. 1995 Coccoid or unbranched filaments; one or two chloroplasts with one pyrenoid; most chloroplasts parietal; cleavage furrow during cell division but no cell plate or phragmoplast; sexual reproduction unknown. *Entransia, Interfilum, Klebsormidium*. •••Phragmoplastophyta Lecointre & Guyander 2006 Cell division by way of some form of phragmoplast; some oogamous, others anisogamous with nonmotile female gamete and motile male gamete. ••••Zygnematophyceae van den Hoek et al. 1995, emend. Hall et al. 2009 Without ciliated stages; sexual reproduction via conjugation; thalli unicellular or filamentous; no centrioles. *Spirogyra, Staurastrum*. ••••Coleochaetophyceae Jeffrey 1982 Thalli discs of cells or branched filaments; sheathed hairs as extensions of the cell wall. *Coleochaete, Chaetosphaeridium*. ••••Charophyceae Smith 1938, emend. Karol et al. 2009 [Charales Lindley 1836; Charophytae Engler 1887] Thallus attached to substrate with rhizoids; thallus a central axis of multinucleate internodal cells, with whorls of branchlets radiating from mononucleate cells at nodes; calcium carbonate accumulates in cell wall of many species; haplobiontic life cycle; sexual reproduction oogamous with sperm cells; differentiated sperm and egg producing organs; antheridium with several shield cells and a manubrium that gives rise to spermatogenous filaments; primarily in fresh water. *Chara, Nitella, Tolypella*. ••••Embryophyta Engler 1886, emend. Lewis and McCourt 2004 [Cormophyta Endlicher 1836; Plantae Haeckel 1866] Ciliated basal bodies, when present, with distinctive multilayered structure of microtubules and cytoskeletal anchor; open mitosis with phragmoplast at cytokinesis; plasmodesmata and other characteristic cell–cell junctions; diplobiontic life cycle, with vegetative propagation possible in many; alternation of generations with fertilization of egg by sperm inside protective test; embryology with tissue differentiation coordinated by hormones; differentiated sperm and egg cells, may be on different sexual individuals, on different organs of the same individual or in the same organ. Subdivisions not shown.**Sar** Burki et al. 2008, emend. Adl et al. 2012 The least inclusive clade containing *Bigelowiella natans* Moestrup and Sengco 2001 (Rhizaria), *Tetrahymena thermophila* Nanney and McCoy 1976 (Alveolata), and *Thalassiosira pseudonana* Cleve 1873 (Stramenopiles). This is a node‐based definition in which all of the specifiers are extant; it is intended to apply to a crown clade; qualifying clause—the name does not apply if any of the following fall within the specified clade—*Homo sapiens* Linnaeus 1758 (Opisthokonta), *Dictyostelium discoideum* Raper 1935 (Amoebozoa), *Arabidopsis thaliana* (Linnaeus) Heynhold 1842 (Archaeplastida), *Euglena gracilis* Klebs 1883 (Excavata), *Emiliania huxleyi* (Lohmann) Hay and Mohler in Hay et al. 1967 (Haptophyta). The name is derived from the acronym of the three groups united in this clade. The apparent composition of Sar is: Alveolata, Rhizaria and Stramenopiles, as defined in Adl et al. 2012. The primary reference phylogeny is Burki et al. (2008, Fig. 1). •**Stramenopiles** Patterson 1989, emend. Adl et al. 2005 Motile cells typically biciliate, typically with heterokont ciliation—anterior cilium with tripartite mastigonemes in two opposite rows and a posterior usually smooth cilium; tubular mitochondrial cristae; typically, four microtubular kinetosomal roots. *Incertae sedis* Stramenopiles: Environmental lineages MAST‐21, MAST‐25 Massana et al. 2014 Uncultured groups detected in molecular marine surveys amplifying directly 18S rDNA genes. These clades are mostly detected in the analysis of small size fractions (pico‐ and nanoeukaryotes). *Incertae sedis* Stramenopiles: *Platysulcus*. Marine bacterivorous heterokont cell with short anterior cilium and long posterior cilium; cell with wide, shallow ventral furrow. ••Bigyra Cavalier‐Smith 1998, emend. 2006 (R) Heterotrophs, mostly phagotrophs, without vegetative cell walls. •••Opalozoa Cavalier Smith 1991, emend. 2006 Cilia without tubular hairs or absent; without plastids, typically without vegetative cell walls; most of them phagotrophic but often osmotrophic saprotrophs in vertebrate guts. *Incertae sedis* Opalozoa: Spherical or D‐shaped cells with two heterokont cilia; microaerophilic, in marine sediments. *Cantina, Rictus*. *Incerta sedis* Opalozoa: Environmental lineages MAST‐12, MAST‐16, MAST‐22 and MAST‐24. Massana et al. 2014 (R) Uncultured groups detected in molecular marine surveys amplifying directly 18S rDNA genes, detected mostly in the analysis of small size fractions (pico and nanoeukaryotes). ••••Nanomonadea Cavalier‐Smith 2012 (clade MAST‐3) Phagotrophic and non‐photosynthetic free‐living ciliated cells. Includes the uniciliated *Solenicola* and *Incisomonas,* and their related environmental 18S rDNA sequences. *Incisomonas*,* Solenicola*. ••••Opalinata Wenyon 1926, emend. Cavalier‐Smith 1997 [Slopalinida Patterson 1985] Pluriciliated with double‐stranded transitional helix at the transitional region between kinetosome and cilium; cilia without tubular hairs evenly spaced cortical ridges underlain by microtubules, ranging from singlets to ribbons; cyst‐forming; many are osmotrophic in vertebrate guts. •••••Proteromonadea Grasse 1952 (P?) One or two anterior pairs of anisokont cilia; uninucleate; endobionts in intestinal tract of amphibians, reptiles and mammals. *Karotomorpha, Proteromonas*. •••••Opalinea Wenyon 1926 Multiciliated with cilia originating from an anterior morphogenetic centre, the falx, and forming oblique longitudinal rows or files; microtubular ribbons supporting longitudinal pellicular ridges between ciliary rows; two to many monomorphic nuclei; endobionts in amphibians and some fish; life cycle complex, with sexual processes induced by hormones of host and linked to the host's life cycle. *Cepedea, Opalina, Protoopalina, Protozelleriella, Zelleriella*. •••••*Blastocystis* Alexeieff 1911 Rounded aciliated yeast‐like cells, anaerobic commensals/parasites of intestinal tracts; cilia secondarily lost. Environmental samples and from mammalian, avian, and reptile hosts, many without specific host. *Blastocystis*. ••••Placidida Moriya et al. 2002 Biciliate cells without plastids; described species have mastigonemes on anterior cilium, attach to substrates by posterior cilium during feeding; double‐stranded transitional helix. *Placidia, Suigetsumonas, Wobblia*. ••••Bicosoecida Grasse 1926, emend. Karpov 1998 Biciliate with or without tripartite mastigonemes, typically lacking transitional helix; without plastids; phagotrophic with cytostome, supported by broad microtubular rootlet No. 2 of posterior cilium; predominantly sedentary, often attach to substrate with posterior cilium; with or without lorica; solitary and colonial. *Adriamonas, Anoeca, Bicosoeca, Caecitellus, Cafeteria, Cyathobodo*,* Filos, Halocafeteria, Nanum, Paramonas, Pseudobodo, Pseudodendromonas*. •••Sagenista Cavalier‐Smith 1995 Heterotrophic phagotrophs and in some cases osmotrophs, biciliated cells present in some stages of their life cycle in most species. *Incertae sedis* Sagenista environmental lineages MAST‐4, MAST‐7, MAST‐8, MAST‐9, MAST‐10, MAST‐11, MAST‐20 Massana et al. 2014 (R). Uncultured groups detected in molecular marine surveys amplifying directly 18S rDNA genes. These clades are mostly detected in the analysis of small size fractions (pico and nanoeukaryotes). The majority of these clades form a sister group to Labyrinthulomycetes. ••••Labyrinthulomycetes Dick 2001 Producing an ectoplasmic network of anastomosing branched wall‐less filaments with an organelle called bothrosome; Golgi‐derived scales; biciliate zoospores with lateral insertion in many species. •••••Amphitremida Poche 1913, emend. Gomaa et al. 2003 Phagotrophs or mixotrophs; planktonic or benthic; aerobic, freshwater and marine, and anaerobic/microaerophilic environments. *Amphitrema, Archerella, Diplophrys, Paramphitrema*. •••••Amphifilida Cavalier‐Smith 2012 Pseudostomes instead of true bothrosomes, ectoplasmic elements in the form of pseudopodia. *Amphifila,* Fibrophrys, *Sorodiplophrys*. •••••Oblongichytrida Bennett et al. 2017 Slender oblong zoospores. *Oblongichytrium* •••••Labyrinthulida Doffein 1901 Spindle‐shaped vegetative cells distributed in an extensive ectoplasmic net; zoospores with eyespots; sexual reproduction. *Aplanochytrium, Labyrinthula Stellarchytrium*. •••••Thraustochytrida Sparrow 1943 Cells producing a small ectoplasmic net; presence of interphase centrioles in vegetative cells; no eyespots; no sexual reproduction. *Althornia, Aurantiochytrium, Botryochytrium, Japanochytrium, Monorhizochytrium, Parietichytrium, Schizochytrium, Sicyoidochytrium Thraustochytrium, Ulkenia*. ••••Pseudophyllomitidae Shiratori et al. 2016 (MAST‐6) Free‐living phagotrophic biciliates, anterior cilium with tubular mastigonemes; mitochondria with tubular cristae; ciliary transitional region with transitional helix. *Pseudophyllomitus* (P). ••Gyrista Cavalier‐Smith 1998 Cells with helical or double helix/ring system ciliary transition zone. *Incertae sedis* Gyrista: Environmental lineages MAST‐1, MAST‐2, MAST‐23 Massana et al. 2014 Uncultured groups detected in molecular marine surveys amplifying directly 18S rDNA genes. These clades are mostly detected in the analysis of small size fractions (pico and nanoeukaryotes). •••Developea Karpov et Aleoshin 2016 Free‐swimming, naked, heterotrophic, bearing two cilia, anterior cilium with mastigonemes. *Developayella, Develorapax*. •••Hyphochytriales Sparrow 1960 Single anteriorly directed cilium. ••••Anisolpidiaceae Karling 1943, emend. Dick 2001 Thallus holocarpic. *Anisolpidium, Canteriomyces*. ••••Hyphochytrium Karling 1939 [Hyphochytridiomycetaceae Fischer 1892, emend. Karling 1939] Thallus eucarpic and polycentric. *Hyphochytrium*. ••••Rhizidiomycetaceae Karling 1943 Thallus eucarpic and monocentric. *Latrostium*,* Rhizidiomyces*,* Rhizidiomycopsis*. •••Peronosporomycetes Dick 2001 [Oomycetes Winter 1897, emend. Dick 1976] Thallus mainly aseptate; cell wall of glucan‐cellulose, may have minor amount of chitin; haplomictic‐B nuclear cycle; lysine synthesized via the diaminopimelate (DAP) pathway; lanosterol directly from squalene oxide; zoospores biciliate and heterokont but rarely uniciliate; cilia anteriorly inserted; anteriorly directed cilium shorter; transitional plate of kinetosome sitting above the plasma membrane with a central bead; kinetid base structure with six parts, including four roots; oogamous reproduction that results in the formation of thick‐walled sexual spores known as oospores, due to contact between male and female gametangia in the most derived groups. *Incertae sedis* Peronosporomycetes: *Atkinsiella, Ciliomyces, Crypticola, Ectrogella, Eurychasma, Halodaphnea, Haliphthoros, Haptoglossa, Lagena, Lagenisma, Olpidiopsis, Pontisma, Pythiella, Rozellopsis, Sirolpidium*. ••••Saprolegnialean lineage Lara in Adl et al. 2019 Obligate biotrophs and facultative parasites. Oogamous sexual reproduction. Two, often morphologically different generations of zoospores, which may have lost cilia secondarily. Complex kinetosome‐associated “K‐bodies”. *Achlya, Aphanomyces, Aplanopsis, Apodachlya, Aquastella, Geolegnia, Leptomitus, Newbya, Pythiopsis, Protoachlya, Salisapilia, Saprolegnia,Thraustotheca*. ••••Peronosporalean lineage Lara in Adl et al. 2019 Obligate biotrophs and facultative parasites. Oogamous sexual reproduction. Unable to synethize sterols required for sexual reproduction. Characteristic periplasmic pattern of oogenesis. *Albugo, Bremia, Chlamydomyzium, Halophytopthora, Hyaloperonospora, Lagenidium, Myzocytiopsis, Peronospora, Plasmopara, Pythium, Pseudoperonospora, Phytophthora, Phytopythium, Pustula*. •••Pirsoniales Cavalier‐Smith 1998, emend. 2006 Biciliate parasites of diatoms that differentiate into an intracellular feeding part (trophosome) and external generative part (auxosome). *Pirsonia*. •••Actinophryidae Claus 1874, emend. Hartmann 1926 Axopodia emerging from amorphous centrosome near nuclei; axonemal microtubules in double interlocking coils; single central nucleus or several peripheral nuclei; tubular mitochondrial cristae; two types of extrusomes for prey‐capture along axopodia; cysts covered with siliceous elements; autogamy reported within cysts; cell division with semi‐open orthomitosis; cilia never formed; freshwater and marine. *Actinophrys*,* Actinosphaerium*. •••Ochrophyta Cavalier‐Smith 1986, emend. Cavalier‐Smith & Chao 1996 Commonly with chloroplasts, endoplasmic reticulum surround plastids like a membrane; plastids commonly containing chlorophyll *c1*, and often *c2*. ••••Chrysista Cavalier‐Smith 1986 Unicellular, or filamentous or parentchymatous; ciliary supra‐tz helix ancestrally; eyespot present or absent; transitional helix present or absent; chloroplast usually few in number and with girdle lamella; chloroplasts endoplasmic reticulum usually attached to the nuclear envelope. *Incertae sedis* Chrysista: Environmental lineages MOCH‐3, MOCH‐5 Massana et al. 2014 Uncultured groups detected in molecular marine surveys amplifying directly 18S rDNA genes. These clades are mostly detected in the analysis of small size fractions (pico and nanoeukaryotes). *Incertae sedis* Chrysista: Environmental lineages Synchromophyceae, Chrysomerophyceae, *Picophagus*,* Chrysowaernella*,* Aurearena*. •••••Chrysophyceae Pascher 1914 Predominately ciliated cells, but also capsoid, coccoid, filamentous and parenchymatous forms; swimming cells biciliated—one anteriorly directed and one laterally directed; tripartite mastigonemes with short and long lateral hairs on the shaft; kinetosome usually with four microtubular kinetosomal roots and one large striated root or rhizoplast; ciliary transitional helix with 4–6 gyres located above the major transitional plate; no paraciliary rod; cell coverings, when present, include organic scales, silica scales, organic lorica and cellulose cell wall; chloroplast with girdle lamella; outer chloroplast endoplasmic reticulum membrane with direct membrane connection to the outer nuclear envelope membrane; plastid DNA with ring‐type genophore; eyespots present or absent; plastid pigments include chlorophylls *a* and *c*1 & *c*2, fucoxanthin, violaxanthin, anthaxanthin and neoxanthin; plastid secondarily lost in some, as noted below. *Incertae sedis* Chrysophyceae: *Chryososaccus, Chrysosphera, Cyclonexis, Lygynion, Phaeoplaca*. ••••••Chromulinales Pascher 1910 Swimming cells with only one cilium visible by light microscopy; photosynthetic; four microtubular kinetosomal roots. *Chromulina*,* Chrysomonas*. ••••••Hibberdiales Andersen 1989 Swimming cells with only one cilium visible by light microscopy; photosynthetic; three microtubular kinetosomal roots. *Hibberdia*. ••••••Ochromonadales Pascher 1910 Swimming cells with two cilia visible by light microscopy; some secondarily lost photosynthetic ability, and are colourless; heterotrophic species can be phagotrophic. *Spumella, Pedospumella, Ochromonas*. ••••••Paraphysomonadida Scoble & Cavalier‐Smith 2014 Phagotrophic colourless cells with two visible cilia; scales composed of unperforated base plate, usually circular, with slender simple spines. *Paraphysomonas*. ••••••Synurales Andersen 1987 Predominately ciliated photosynthetic cells, some form benthic palmelloid colonies; swimming cells usually with two anteriorly directed cilia—one bearing tripartite tubular mastigonemes with short and long lateral hairs on their shafts, two microtubular kinetosomal roots and one large striated kinetosomal root or rhizoplast. *Chrysodidymus, Mallomonas, Synura, Tesselaria*. •••••Eustigmatales Hibberd 1981 Coccoid organisms, single cells or colonies; swimming cells biciliate—one anteriorly directed and one posteriorly directed; four microtubular kinetosomal roots and one large striated kinetosomal root or rhizoplast; ciliary transitional helix with six gyres located above the major transitional plate; no paraciliary rod; cell walls present; chloroplast without girdle lamella; outer chloroplast endoplasmic reticulum membrane with direct membrane connection to the outer nuclear envelope membrane; plastid DNA with ring‐type genophore; eyespot present but located outside of the chloroplast; plastid pigments include chlorophylls a, violaxanthin, and vaucherioxanthin. *Botryochloropsis, Eustigmatos, Monodopsis, Nannochloropsis, Pseudocharaciopsis, Vischeria*. •••••Phaeophyceae Hansgirg 1886 [Kjellman 1891; Pfitzer 1894] Filamentous, syntagmatic, parenchymatous or ciliated; swimming cells with two cilia usually inserted laterally—one anteriorly directed and one posteriorly directed; usually four microtubular kinetosomal roots but no striated kinetosomal root or rhizoplast; ciliary transitional helix typically with six gyres located above the major transitional plate; no paraciliary rod; little to no substantial tissue differentiation occurring in parenchymatous forms; cell wall present, containing alginate compounds and cellulose; plasmodesmata or pores between cells in parenchymatous forms; chloroplasts with girdle lamella; outer chloroplast endoplasmic reticulum membrane with direct membrane connection to the outer nuclear envelope membrane; plastid DNA with ring‐type genophore; eyespots present or absent; plastid pigments include chlorophylls *a* and *c*1 & *c*2, fucoxanthin, and violaxanthin. Note that several subdivisions are separated on the basis of life history and gene sequence information, but taxonomic classification is still in flux. ••••••Ascoseirales Petrov 1964 Sporophyte parenchymatous, with intercalary growth; several scattered discoid plastids without pyrenoid; heteromorphic life cycle but gametophyte not free‐living; isogamous sexual reproduction. *Ascoseira*. ••••••Desmarestiales Setchell & Gardner 1925 Gametophyte small and filamentous; sporophyte larger and pseudoparenchymatous; several scattered discoid plastids with no pyrenoid; trichothallic growth; heteromorphic life cycle; oogamous sexual reproduction. *Arthrocladia, Desmarestia* (P), *Himantothallus, Phaeurus*. ••••••Dictyotales Bory de Saint‐Vincent 1828 Gametophyte and sporophyte parenchymatous, with apical or marginal growth; several scattered discoid plastids without pyrenoid; isomorphic life cycle; oogamous sexual reproduction. *Dictyota, Dilophus, Lobophora, Padina, Stypopodium, Taonia, Zonaria*. ••••••Discosporangiales Kawai et al. 2007 Simple branched fillaments with apical growth; plastids multiple, discoid, without pyrenoids; species lack heterotrichy and phaeophycean hairs. Note that the early divergence of this group from other brown algae is reflected in their continuous division and elongation of vegetative cells. *Choristocarpus, Discosporangium*. ••••••Ectocarpales Bessey 1907, emend. Silva & Reviers 2000 Gametophyte and sporophyte uniseriate filaments, either branched or unbranched, with diffuse growth; one or more ribbon‐shaped plastids with pyrenoid; isomorphic life cycle; isogamous, anisogamous or oogamous sexual reproduction*. Adenocystis, Acinetospora, Asterocladon, Asteronema, Chordaria, Ectocarpus, Scytosiphon*. ••••••Fucales Bory de Saint‐Vincent 1927 Sporophyte parenchymatous, with apical cell growth; several scattered discoid plastids without pyrenoid; diploid life stage only with meiosis producing gametes; (mostly) oogamous sexual reproduction. *Ascophyllum, Bifurcaria, Cystoseira, Druvillaea, Fucus, Hormosira, Sargassum, Turbinaria*. ••••••Ishige Yendo 1907 [Ishigeacea Okamura 1935, Ishigeales Cho et al. 2004] Isomorphic alternation of generations, with apical cell growth; scattered discoid plastids without pyreniods; terminal unilocular sporangia or uniseriate plurilocular sporangia; cortex pseudoparenchymatous with assimilatory filaments; phaeophycean hairs in cryptostigmata. *Ishige*. ••••••Laminariales Migula 1908 Gametophyte small and filamentous with apical growth; sporophyte large and parenchymatous, with intercalary growth; several scattered discoid plastids without pyrenoid; heteromorphic life cycle; oogamous sexual reproduction with eggs sometimes ciliated. *Akkesiophycus, Alaria, Chorda, Costaria, Laminaria, Lessonia, Pseudochoda*. ••••••*Nemoderma* Schousboe ex Bonnet 1892 [Nemodermatales Parente et al. 2008] (M) Encrusting heterotrichous thalli; numerous discoid plastids per cell without pyrenoids; isomorphic life cycle; anisogamous gametes; plurilocular reproductive structures are lateral, whereas unilocular sporangia are intercalary. *Nemoderma*. ••••••Onslowiales Draisma & Prud'homme van Reine 2008 An irregularly branched oligostichous thallus, both branches and reproductive structures are the result of lateral divisions from thallus cells; prominent apical cell lacking transverse division of subapical cells; multiple discoid plastids without pyrenoid; isomorphic life cycle; sexual reproduction can occur by plurilocular or unilocular sporangia, or via vegetative propagules lacking a central apical cell. *Onslowia*. ••••••Ralfsiales Nakamura ex Lim & Kawai 2007 Crustose in at least one phase of the life history or via a disc‐type germination; plastids without pyrenoids, from one to many per cell; plurilocular sporangium intercalary and having one or more terminal sterile cells. *Lithoderma, Neoralfsia, Pseudolithoderma, Ralfsia*. ••••••Scytothamnales Peters & Clayton 1998 Gametophyte large and parenchymatous, with intercalary growth; sporophyte small and filamentous, with apical growth; one or more stellate or axial plastids with pyrenoid; heteromorphic alternation of generations; anisogamous sexual reproduction. *Scytothamnus, Splachnidium, Stereocladon*. ••••••Sphacelariales Migula 1908 Gametophyte and sporophyte branched multiseriate filaments, with apical growth; several scattered discoid plastids without pyrenoid; usually an isomorphic alternation of generations; isogamous, anisogamous or oogamous sexual reproduction. *Chaetopteris, Halopteris, Stypocaulon, Sphacelaria, Verosphacella*. ••••••Sporochnales Sauvageau 1926 Gametophyte and larger sporophyte pseudoparenchymatous, with trichothallic growth; several scattered discoid plastids without pyrenoid; heteromorphic alternation of generations; oogamous sexual reproduction. *Bellotia, Carpomitra, Nereia, Sporochonus, Tomaculopsis*. ••••••Syringodermatales Henry 1984 Gametophyte 2–4 cells; sporophyte parenchymatous with apical and marginal growth; several scattered discoid plastids without pyrenoid; heteromorphic alternation of generations but gametophyte not free‐living; isogamous sexual reproduction. *Syringoderma*. ••••••Tilopteridales Bessey 1907 Polystichous construction of the thallus, which grows by a trichothallic meristem; several scattered plastids without pyrenoids; isomorphic alternation of generations; oogamous sexual reproduction. *Cutleria, Halosiphon, Haplospora, Phaeosiphoniella, Phyllaria, Tilopteris*. •••••Phaeothamniophyceae Andersen & Bailey in Bailey et al. 1998 Filamentous, capsoid, palmelloid, ciliated, or coccoid; swimming cells biciliated—anteriorly directed cilium bearing tripartite tubular mastigonemes and posteriorly directed cilium without tripartite hairs; four microtubular kinetosomal roots but no striated kinetosomal root or rhizoplast; ciliary transitional helix with 4–6 gyres located above the major transitional plate; no paraciliary rod; cells covered with an entire or two‐pieced cell wall; chloroplast with girdle lamella; chloroplast endoplasmic reticulum membrane with direct membrane connection to the outer nuclear envelope membrane; plastid DNA with ring‐type genophore; eyespots present; plastid pigments include chlorophylls *a* and *c*, fucoxanthin, heteroxanthin, diatoxanthin and diadinoxanthin. ••••••Phaeothamniales Bourrelly 1954, emend. Andersen & Bailey in Bailey et al. 1998 (R) Distinguished from the Pleurochloridales based upon molecular phylogenetic analyses. *Phaeothamnion*. ••••••Pleurochloridales Ettl 1956 (R) Distinguished from the Phaeothamniales based upon molecular phylogenetic analyses. *Pleurochloridella*. •••••Raphidophyceae Chadefaud 1950, emend. Silva 1980 Naked swimming biciliates with one anteriorly directed cilium bearing tripartite tubular mastigonemes and one posteriorly directed cilium lacking tripartite mastigonemes; microtubular kinetosomal roots present but poorly characterized; one large striated kinetosomal root or rhizoplast present; ciliary transitional helix absent; no paraciliary rod; chloroplast with or without girdle lamella; outer chloroplast endoplasmic reticulum membrane with no or very weak direct membrane connection to the outer nuclear envelope membrane; plastid DNA with scattered granule‐type genophore; eyespots absent; plastid pigments include chlorophylls *a* and *c*1 & *c*2; carotenoid composition distinctly different between marine (m) and freshwater (fw) species—fucoxanthin (m), violaxanthin (m), heteroxanthin (fw), vaucherioxanthin (fw). *Chattonella, Fibrocapsa, Goniostomum, Haramonas, Heterosigma, Merotricha, Olisthodiscus, Vacuolaria*. •••••Schizocladia Henry et al. in Kawai et al. 2003 [Schizocladales Kawai et al. 2003] (M) Branched filamentous algae with biciliated zoospores—an immature cilium bearing tubular tripartite hairs; ciliary transitional helix with ˜5 gyres located above the transitional plate; ciliary apparatus and kinetosomal roots, if present undescribed; chloroplasts parietal with girdle lamella; outer chloroplast endoplasmic reticulum membrane with direct membrane connection to the outer nuclear envelope membrane; plastid DNA with ring‐type genophore; plastids with chlorophylls *a* and *c* as well as carotenoids (unverified); cell wall containing alginates but lacking cellulose and plasmodesmata; eyespot present; major storage product undescribed. *Schizocladia*. •••••Xanthophyceae Allorge 1930, emend. Fritsch 1935 [Heterokontae Luther 1899; Heteromonadea Leedale 1983; Xanthophyta Hibberd 1990] Predominately coccoid or filamentous, rarely amoeboid, ciliated or capsoid; swimming cells with two cilia—one anteriorly directed and bearing tripartite tubular hairs and one posteriorly directed and lacking tripartite hairs; four microtubular kinetosomal roots and one large striated kinetosomeal root or rhizoplast; ciliary transitional helix with six apparently double gyres located above the major transitional plate; no paraciliary rod; cell walls typical, probably of cellulose and either entire or H‐shaped bisectional walls; chloroplast with girdle lamella; outer chloroplast endoplasmic reticulum membrane with direct membrane connection to the outer nuclear envelope membrane; plastid DNA with ring‐type genophore; eyespots present or absent; plastid pigments include chlorophylls *a* and *c*1 & *c*2, violaxanthin, heteroxanthin, and vaucherioxanthin. ••••••Tribonematales Pascher 1939 Filamentous, coccoid, and capsoid forms, sometimes becoming parenchymatous or multinucleate with age; cell walls, when present, either with H‐shaped overlapping cell wall pieces or with complete or entire cell walls; elaborate reproductive structures lacking. *Botrydium, Bumilleriopsis, Characiopsis, Chloromeson, Heterococcus, Monadus, Ophiocytium, Sphaerosorus, Tribonema, Xanthonema*. ••••••Vaucheriales Bohlin 1901 Siphonous filaments; elaborate sexual reproductive structures as antheridia and oogonia. *Vaucheria*. ••••Diatomista Derelle et al. 2016, emend. Cavalier‐Smith 2017 (R) Unicellular or aggregative; no cell wall; naked or with silica frustules or scales; without supra‐tz helix *Incertae* sedis Diatomista: Environmental lineages MOCH‐1, MOCH‐2, MOCH‐4 Massana et al. 2014 Uncultured groups detected in molecular marine surveys amplifying directly 18S rDNA genes. These clades are mostly detected in the analysis of small size fractions (pico and nanoeukaryotes). •••••Bolidophyceae Guillou et al. 1999 [Parmales Booth and Marchant 1987] Two types of cells known: motile and nonmotile. Swimming cells with two cilia, one anteriorly directed (with mastigonemes) and one posteriorly directed; no microtubular or fibrillar kinetosomal roots; ciliary transitional helix absent; no paraciliary rod; chloroplast with girdle lamella; outer chloroplast endoplasmic reticulum membrane with direct membrane connection to the outer nuclear envelope membrane; plastid DNA with ring‐type genophore; no eyespot; plastid pigments include chlorophylls *a* and *c* 1‐3, fucoxanthin, 19′‐butanoyloxyfucoxanthin, diatoxanthin and diadinoxanthin. Nonmotile cells possess chloroplasts like those of swimming cells but are surrounded by complete multipartite walls comprised of abutting silica plates (circular shield and ventral plates, elongate or triradiate girdle plates, and triradiate dorsal plates), often bearing ridges and spines and radiating lines of pores; exclsuively marine. *Bolidomonas*,* Triparma*,* Tetraparma*,* Pentalamina*. •••••Diatomeae^34^ Dumortier 1821 [= Bacillariophyta Haeckel 1878] Vegetative cells cylindrical with a circular, elongate or multipolar cross‐section, lacking any trace of cilia except in the sperm of centric lineages; cell wall complete, composed of tightly integrated silicified elements and comprised of two valves, one at each end of the cell, with several girdle bands (as hoops or segments) covering the cylindrical ‘girdle’ lying between the valves; valves penetrated by simple or chambered pores arranged in rows (striae); other openings often present in the valves (so‐called ‘fields’ and ‘processes’, slits), involved in secretion and motility; chloroplasts usually present, bounded by four membranes, and with lamellae of three thylakoids and a ring nucleoid (rarely multiple nucleoids); ciliated sperm cells bearing a single anterior cilium with a 9 + 0 axoneme and mastigonemes; life cycle diplontic and of unique pattern—slow size reduction over several years during the vegetative phase, caused by an unusual internal wall morphogenesis, alternating with rapid size restitution via a growth phase (auxospore) over several days. ••••••Leptocylindrophytina D.G. Mann, in Adl et al. 2019 Chain‐forming or solitary; valves circular, valve pattern radiating from a central circular annulus; pores simple; girdle bands segmental to strap‐like; sexual reproduction oogamous or apparently absent; chloroplasts several, small; exclusively marine. •••••••Leptocylindrophyceae D.G. Mann, in Adl et al. 2019 Chain‐forming (linked by short spines or secretions), delicate; valves circular, flat‐topped or domed; a single simple process often present near the annulus; girdle bands segmental; auxospore forming a dormant resting stage (rare in other diatom clades). *Leptocylindrus*,* Tenuicylindrus*. •••••••Corethrophyceae D.G. Mann, in Adl et al. 2019 Solitary; valves circular, domed; elaborate articulating silica spines secreted from around the valve margin; rimoportulae and other processes absent; girdle bands segmental. *Corethron*. ••••••Ellerbeckiophytina D.G. Mann, in Adl et al. 2019 Chain‐forming (linked by valve spines), heavily silicified; valves circular, with radially symmetrical valve pattern; rimoportulae or tube processes small, restricted to the mantle; girdle bands hoop‐like; marine and freshwater; sexual reproduction as yet unknown. *Ellerbeckia*. ••••••Probosciophytina D.G. Mann, in Adl et al. 2019 Usually solitary, with a long pervalvar axis; valves circular, extended into an eccentric beak (proboscis); rimoportulae and other processes absent; girdle bands segmental; exclusively marine; sexual reproduction oogamous. *Proboscia*. ••••••Melosirophytina D.G. Mann, in Adl et al. 2019 Usually chain‐forming (linked by valve spines or by central secretions); valves circular, radially symmetrical, pattern radiating from a central annulus (which may occupy the whole of the valve face in *Aulacoseira* and some *Melosira*); rimoportulae small, scattered on the valve face or marginal; girdle bands hoop‐like or segmental; marine and freshwater; sexual reproduction oogamous. *Aulacoseira, Melosira, Hyalodiscus*,* Stephanopyxis*,* Paralia*,* Endictya*. ••••••Coscinodiscophytina Medlin & Kaczmarska 2004, emend. Solitary, robust; valves generally circular, pattern radiating from a central, subcentral or submarginal circular annulus; valve structure chambered; rimoportulae central, scattered on the valve face, or in a submarginal ring, sometimes with slit‐like apertures, tubes or cap‐like structures externally; girdle bands hoop‐like; sexual reproduction oogamous; mostly marine. *Actinoptychus, Coscinodiscus*,* Actinocyclus*,* Asteromphalus*,* Aulacodiscus*,* Stellarima*. ••••••Rhizosoleniophytina D.G. Mann, in Adl et al. 2019 Chain‐forming, rarely solitary; valves circular, radially symmetrical or with the annulus displaced towards one side; valve structure simple; rimoportula single, associated closely with the annulus, sometimes developed externally into a spine; girdle bands segmental; sexual reproduction oogamous; marine. *Guinardia, Rhizosolenia*,* Pseudosolenia*. ••••••Arachnoidiscophytina D.G. Mann, in Adl et al. 2019 Solitary, heterovalvar; valves circular, radially symmetrical; valve structure chambered, complex; valve centre with radial slits (apparently modified rimoportulae); girdle bands hoop‐like; sexual reproduction incompletely known, marine. *Arachnoidiscus*. ••••••Bacillariophytina Medlin & Kaczmarska 2004, emend. Chain‐forming, colonial or solitary; valve outline bipolar or multipolar, rarely circular; valve pattern radiating from a central circular or elongate annulus or from a longitudinal rib (sternum); valve structure simple or chambered; areas of special pores or slits often present, involved in mucilage secretion; rimoportulae present or absent; girdle bands usually hoop‐like; sexual reproduction oogamous with nonmotile eggs and uniciliate sperm, or isogamous with amoeboid or non‐ciliate ‘spinning’ gametes; auxospore usually with band‐like elements (the ‘perizonium’) that facilitate anisometric expansion; chloroplasts many, few or one, very rarely apochlorotic. •••••••Mediophyceae Jouse & Proshkina‐Lavrenko in Medlin & Kaczmarska 2004 Chain‐forming or solitary; valve outline bipolar or multipolar, sometimes (perhaps secondarily) circular; valve pattern radiating from a circular or elongate annulus; rimoportulae central or marginal; sexual reproduction oogamous; auxospore with scales and/or perizonium; chloroplasts usually many, small. ••••••••Chaetocerotophycidae Round & R.M. Crawford in Round et al. 1990, emend. Mostly chain‐forming (attached by the valve poles and setae, where present, or by pads) delicate planktonic species; valve outline usually bipolar, usually with projections or horns at the poles, rarely multipolar or circular; pore fields present at the poles, often also long hollow setae; annulus central or submarginal; rimoportulae central or submarginal; mostly marine, but with a few large robustly silicified freshwater forms. *Chaetoceros*,* Bacteriastrum*,* Dactyliosolen*,* Cerataulina*,* Hemiaulus*,* Eucampia*,* Acanthoceras*,* Urosolenia*,* Terpsinoë*,* Hydrosera*. ••••••••Lithodesmiophycidae Round & R.M. Crawford in Round et al. 1990, emend. Chain‐forming (linked by the valve poles and/or central region) or solitary; valve outline bi‐, tri‐ or to multipolar; valve structure simple, with valve pattern radiating from a central annulus or a scatter of pores; valve poles lacking well‐defined pore fields; rimoportula present, central (within the annulus), sometimes extended externally into a long tube. *Lithodesmium*,* Lithodesmioides*,* Helicotheca*,* Bellerochea*,* Ditylum*. ••••••••Thalassiosirophycidae Round & R.M. Crawford in Round et al. 1990 Cells connected into chains by chitin threads, colonial (forming sheets), or solitary; valve outline almost always circular; valve pattern generally organized radially about a central annulus; valve structure simple or chambered; generally with one or two submarginal rimoportulae; almost all members characterized by ‘fultoportulae’ (special processes involved in chitin thread secretion); auxospores lacking perizonia; marine and freshwater. *Thalassiosira*,* Lindavia*,* Cyclotella, Stephanodiscus, Cyclostephanos*,* Discostella*,* Bacteriosira*,* Skeletonema*,* Detonula*. ••••••••Cymatosirophycidae Round & R.M. Crawford in Round et al. 1990 Generally chain‐forming (linked by spines on the valves), very small‐celled, delicate forms; often heterovalvar; valve outline bipolar; valve pattern sometimes organized about a central annulus but more often with scattered pores; small well‐defined pore fields (‘ocelluli’) present at the apices and some valves often bearing long spines (‘pili’); a single rimoportula near the centre of some valves; exclusively marine. *Cymatosira*,* Minutocellus*,* Papiliocellulus*,* Leyanella*,* Extubocellulus*,* Plagiogrammopsis*,* Campylosira*,* Brockmanniella*,* Pierrecomperia*. ••••••••Odontellophycidae, D. G. Mann, in Adl et al. 2019 Chain‐forming (linked by mucilage pads) or solitary, generally large‐celled and sometimes very robust; valve outline bi‐ or multipolar, rarely circular; valve pattern organized radially about a central circular or elongate annulus; valve structure simple or chambered; valve poles with well‐defined pore fields (‘ocelli’) often surrounded by a thick rim; rimoportulae present (sometimes with long external tubes) or absent; exclusively marine. *Odontella*,* Triceratium*,* Cerataulus*,* Pleurosira*,* Pseudauliscus*,* Amphitetras*,* Trieres*,* Mastodiscus*. ••••••••Chrysanthemodiscophycidae, D.G. Mann, in Adl et al. 2019 Chain‐forming (linked by mucilage pads) or solitary; valve outline circular, bipolar (and then sometimes extremely elongate, either isopolar or heteropolar) or multipolar; valve pattern organized radially about a central annulus, which can be circular or extremely elongate (and then with striae extending inwards from the annulus, which is therefore ‘bifacial’); valve structure simple or chambered (alveolate); rimoportulae present or absent; pore fields present at the poles (and then poorly differentiated, i.e. ‘pseudocelli’) or absent; exclusively marine. *Chrysanthemodiscus*,* Biddulphiopsis*,* Trigonium*,* Isthmia*,* Lampriscus*,* Stictocyclus*,* Ardissonea*,* Climacosphenia*,* Toxarium*. •••••••Biddulphiophyceae, D. G. Mann, in Adl et al. 2019 Chain‐forming or solitary; valve outline bipolar, with elevated projections at the poles; valve pattern radiating from a circular or elongate annulus; valve structure simple; rimoportulae present; exclusively marine. ••••••••Biddulphiophycidae Round and R.M. Crawford in Round et al. 1990, emend. Chain‐forming (linked by mucilage pads), generally large‐celled and robust; valve outline sometimes undulate; polar projections small or large and valve often with additional doming centrally and sometimes between centre and poles; pattern organized radially about a central circular annulus; pore fields (‘pseudocelli’) present at the poles; rimoportulae central. *Biddulphia*. ••••••••*Attheya* T. West 1860c Delicate, solitary; valve outline bipolar, the ends developed into narrow setae; valve pattern radiating from an elongate annulus; pore fields absent, though the setae may end in specialized regions with spines; a single off‐central rimoportula; sexual reproduction oogamous; exclusively marine. *Attheya*. •••••••Bacillariophyceae Haeckel 1878, emend. Chain‐forming, colonial or solitary; valve outline almost always bipolar; valve pattern organized bilaterally about an elongate axial rib (sternum), as in a feather; valve structure simple or chambered; rimoportulae generally only one or two per valve or none, sometimes accompanied (or replaced?) by special slits (the ‘raphe’) involved in motility; sexual reproduction involving gametangiogamy and almost always with gametes of equal size (although sometimes with behavioural differentiation and/or morphological differences); perizonium generally differentiated into two distinct series, transverse and longitudinal; chloroplasts usually only one, two or a few and large, less often many and small. ••••••••Striatellaceae Kützing 1844 Solitary; valve outline bipolar; valve structure simple; a rimmed pore field present at each pole; rimoportulae present (one at each pole or scattered along the sides of the valve); raphe absent; sexual reproduction not confirmed; exclusively marine. *Striatella*,* Pseudostriatella*. ••••••••Urneidophycidae Medlin 2016 (P?) Chain‐forming (linked by spines on the valves), colonial (linked by pads) or solitary; valve outline bi‐ or multipolar, rarely circular; valve structure simple; pore fields sometimes present at the poles, rimoportulae present or absent; raphe absent; ‘male’ and ‘female’ gametes of equal size but ‘male’ gametes with non‐cilium projections that generate movement; exclusively marine. *Plagiogramma*,* Dimeregramma*,* Rhaphoneis*,* Delphineis*,* Psammoneis*,* Bleakeleya*,* Asterionellopsis*. ••••••••Fragilariophycidae Round in Round, Crawford & Mann 1990, emend. Chain‐forming or colonial (linked by pads or stalks, less often by valve spines) or solitary; valve outline bi‐ or multipolar, very rarely circular (with reduction of the sternum and secondary evolution of radial symmetry); valve structure usually simple; pore fields (rimmed or not) often present at the poles; rimoportulae present or absent, very variable in location but often polar; raphe absent; ‘male’ and ‘female’ gametes of equal or unequal size, amoeboid, or with ‘male’ gametes with non‐cilium projections that generate movement; marine and freshwater. *Fragilaria*,* Synedra*,* Tabellaria*,* Asterionella*,* Diatoma*,* Tabularia*,* Cyclophora*,* Astrosyne*,* Licmophora*,* Rhabdonema*,* Grammatophora*,* Staurosira*,* Thalassionema*. ••••••••Bacillariophycidae D.G. Mann in Round, Crawford & Mann 1990, emend. Usually solitary and motile, less often chain‐forming or colonial (linked by stalks or living in mucilage tubes); valve outline almost always bipolar, frustules sometimes heterovalvar; rimoportulae usually absent (exception: Eunotiales); valve structure simple or chambered; pore fields sometimes present at one or both poles; raphe present (rarely infilled during valve formation); usually one or two chloroplasts per cell, positioned to avoid the area beneath the raphe; gametes morphologically similar, amoeboid or fusing through expansion; marine and freshwater, with many examples of genera or families transgressing the marine–freshwater interface. The vast majority of diatom species belong here, classified into many tens of genera. *Eunotia*,* Achnanthes*,* Bacillaria*,* Nitzschia*,* Pseudo‐nitzschia*,* Cylindrotheca*,* Navicula*,* Seminavis*,* Haslea*,* Stauroneis*,* Pleurosigma*,* Gyrosigma*,* Achnanthidium*,* Cocconeis*,* Frustulia*,* Diploneis*,* Sellaphora*,* Pinnularia*,* Gomphonema*,* Cymbella*,* Didymosphenia, Phaeodactylum*,* Amphora*,* Entomoneis*,* Epithemia*,* Surirella*. •••••Dictyochophyceae Silva 1980 Single cells, colonial ciliated cells or amoebae; swimming cells usually with one cilium, anteriorly directed and bearing tripartite tubular hairs; kinetosomes adpressed to nucleus; no microtubular or fibrillar kinetosomal roots; ciliary transitional helix, when present, with 0–2 gyres located below the major transitional plate; paraciliary rod present; cells naked, with organic scales or with siliceous skeleton; chloroplasts, when present, with girdle lamella; plastid DNA with scattered granule‐type genophore; no eyespot; plastid pigments include chlorophylls *a* and *c* 1 & 2, fucoxanthin, diatoxanthin and diadinoxanthin. ••••••Dictyochales Haeckel 1894 Silica skeleton present on at least one life stage; with chloroplasts. *Dictyocha*. ••••••Pedinellales Zimmermann et al. 1984 Naked, organically scaled or loricate ciliated cells; with or without chloroplasts. *Actinomonas, Apedinella, Ciliophrys, Mesopedinella, Palatinella, Pedinella, Pseudopedinella, Pteridomonas*. ••••••Rhizochromulinales O'Kelly & Wujek 1994 Vegetative cells amoeboid; zoospore ciliated; with chloroplasts. *Rhizochromulina*. •••••Pelagophyceae Andersen & Saunders 1993 Ciliated, capsoid, coccoid, sarcinoid or filamentous; swimming cells with one or two cilia—anteriorly directed cilium bearing bipartite or tripartite tubular hairs and second cilium, when present, directed posteriorly; kinetosome(s) adpressed to nucleus; no microtubular or fibrillar kinetosomal roots on uniciliated cells; four microtubular roots on biciliated cells; ciliary transitional helix, when present, with two gyres located below the major transitional plate; paraciliary rod present or absent; cells naked or with organic thecae or cell walls; chloroplasts with girdle lamella; plastid DNA with scattered granule‐type genophore; no eyespot; plastid pigments include chlorophylls *a & c 1, 2*, fucoxanthin, 19‐hexanoyloxyfucoxanthin, 19‐butanoyloxyfucoxanthin, diatoxanthin and diadinoxanthin. ••••••Pelagomonadales Andersen & Saunders 1993 Ciliated or coccoid organisms; when ciliated, a single cilium without a second kinetosome; no kinetosomal roots. *Aureococcus, Aureoumbra, Pelagococcus, Pelagomonas*. ••••••Sarcinochrysidales Gayral & Billard 1977 Sarcinoid, capsoid, ciliated or filamentous; cells typically with organic cell wall; ciliated cells biciliated with four microtubular kinetosomal roots. *Ankylochrisis, Nematochrysopsis, Pulvinaria, Sarcinochrysis*. •••••Pinguiophyceae Kawachi et al. 2003 Ciliated or coccoid organisms; swimming cells with one or two cilia; tripartite hairs present or absent on immature cilium; 3–4 microtubular kinetosomal roots and one large striated kinetosomal root or rhizoplast; ciliary transitional helix with two gyres located below the major transitional plate; no paraciliary rod; cells naked or enclosed in mineralized lorica; chloroplast with girdle lamella; outer chloroplast endoplasmic reticulum membrane with direct membrane connection to the outer nuclear envelope membrane; plastid DNA with scattered granule‐type genophore; eyespots absent; plastid pigments include chlorophylls *a* and *c*1 & *c*2, fucoxanthin, and violaxanthin. *Glossomastix, Phaeomonas, Pinguiochrysis, Pinguiococcus, Polypodochrysis*.
•**Alveolata** Cavalier‐Smith 1991 Cortical alveolae, sometimes secondarily lost; with ciliary pit or micropore; mitochondrial cristae tubular or ampulliform. ••Colpodellida Cavalier‐Smith 1993, emend. Adl et al. 2005, 2019 Photosynthetic, or non‐photosynthetic and predatory; complex plastids, when present bound by four membranes; mitochondrion with tubular cristae; highly flattened cortical alveoli; microtubules beneath alveolae; conoid‐like structure with apical complex and rostrum; biciliate; micropore present; cysts at least in some species. •••Vitrellaceae Oborník & Lukeš 2012 Immotile vegetative cells with laminated cell walls; autosporangia and zoosporangia contain dozens of immotile autospores and motile biciliate zoospores, respectively; prominent pyrenoid; sporangia carry an operculum; plastid pigmented by chlorophyll *a*, violaxanthin, vaucheria xanthin and β‐carotene; chlorophyll *c* absent. *Vitrella*. •••Colpodellaceae Adl et al. 2019 Non‐photosynthetic ciliated cell predatory on other protists, (found once in human blood). *Colpodella, Chilovora, Voromonas*. •••Chromeraceae Oborník & Lukeš 2012 Immotile coccoid cells reproduce by binary division; zoospores with heterodynamic cilia; pseudoconoid, coccoid wedge and chromerosome present; cells surrounded by thin sporangium wall; plastid pigmented by chlorophyll *a*, violaxanthin, isofucoxanthin and β‐carotene; chlorophyll *c* absent. *Chromera*. •••Alphamonaceae Adl et al. 2019 Non‐photosynthetic predatory ciliated cells on other protists; carbohydrate reserve granules absent. *Alphamonas*. ••Perkinsidae Levine 1978, emend. Adl et al. 2005 [Perkinsozoa Moestrup & Rehnstam‐Holm 1999; Perkinsozoa Norén & Moestrup 1999] Trophozoites parasitic, dividing by successive binary fissions; released trophozoites (termed hypnospores) developing outside host to form zoospores via the formation of zoosporangia or morphologically undifferentiated mononucleate cells via a hypha‐like tube; zoospores biciliate; apical organelles including an incomplete conoid (open along one side), rhoptries, micronemes and micropores, and a microtubular cytoskeleton with both an anterior and posterior polar ring. *Dinovorax, Snorkelia, Parvilucifera, Perkinsus, Rastrimonas,* X‐cell parasites. *Incertae sedis* Perkinsidae: *Psammosa* Okamoto et al. 2012—morphologically close to *Colpodella* and *Voromonas*, but phylogenetically more related to Perkinsidae.*Incertae sedis* Perkinsidae*: Phagodinium*. ••Colponemida Cavalier‐Smith 1993 emend. Adl et al. 2019 (P?) Biciliate alveolates, typically cytotrophic predators, found in soil, freshwater and marine environments. These described genera are most probably multiple genera as DNA sequences obtained are divergent and many remain to be described. Poorly sampled due to cytotrophy, we expect better taxon sampling to improve the resolution of this node. •••Colponemidia Tikhonenkov et al. 2014 Biciliate; three‐membraned alveolar pellicle; two microtubule bands armour longitudinal groove; micropore absent; nontubular mastigonemes present; cytotrophic predators and sometimes on microinvertebrates. *Colponema*. •••Acavomonidia Tikhonenkov et al. 2014 Biciliate; rigid cells; lack longitudinal grooves and apical complex structures; cytotrophic predators. *Acavomonas*. •*••Palustrimonas* Patterson & Simpson 1996 Deep branching colponemid alveolate; cytotrophic predator; longitudinal feeding groove. *Palustrimonas*. •••*Oxyrrhis* Dujardin 1841 [Oxyrrhinaceae Sournia 1984] (M) Without true cingulum and sulcus; intranuclear mitotic spindle; with amphiesmal vesicles and trichocysts; cilia inserted laterally; cytotrophic predator. (There may be multiple species). *Oxyrrhis marina*. ••Dinoflagellata Bütschli 1885, emend. Fensome et al. 1993, emend. Adl et al. 2005 Cells with two cilia in the motile stage—typically, one transverse cilium ribbon‐like with multiple waves beating to the cell's left and longitudinal cilium beating posteriorly with only one or few waves; nucleus typically a dinokaryon with chromosomes remaining condensed during interphase and lacking typical eukaryotic histones and centrioles; dinoflagellate/viral nucleoproteins package chromatin; closed dinomitosis with extranuclear spindle. *Incertae sedis* Dinoflagellata: Naked and thecate genera with uncertain affiliation, e.g. *Adenoides, Akashiwo, Amphidiniella, Ankistrodinium, Apicoporus, Archaeosphaerodiniopsis, Bispinodinium, Bysmatrum, Cabra, Cladopyxis, Crypthecodinium, Cucumeridinium, Dactylodinium, Dicroerisma, Gloeodinium, Grammatodinium, Gynogonadinium, Gyrodiniellum, Halostylodinium, Heterodinium, Moestrupia, Paragymnodinium, Phytodinium, Plagiodinium, Planodinium, Pileidinium, Pseudadenoides, Pseudothecadinium, Pyramidodinium, Rhinodinium, Roscoffia, Sabulodinium, Sphaerodinium, Spiniferodinium, Testudodinium, Thecadinium,Thecadiniopsis, Togula.* Incertae sedis Dinoflagellata: Parasitic dinoflagellates with dinokaryon during part of the life cycle only; not highly vacuolated. Fossils unknown. [Blastodiniales Chatton 1906, no longer valid]. *Amyloodinium, Apodinium, Cachonella, Caryotoma, Crepidoodinium, Haplozoon, Oodinium, Myxodinium, Piscinoodinium, Protoodinium, Rhinodinium, Schyzochitriodinium, Stylodinium*. •••Syndiniales Loeblich III 1976 Aplastidic intracellular parasites of marine protists and metazoan, generally surrounded by an external parasitophorous membrane inside the host; sporogenesis by palintomic (schizogonous) divisions; motile cells (i.e. dinospores or gametes) with a dinokont‐like arrangement of cilia; lacking well‐defined cingulum and sulcus; transverse cilium wrapping loosely around cell. V‐shaped chromosomes, attach to the nucleus membrane by the apex and remain condensed during interphase. Although there is a strong morphological argument for the common origin of the Syndiniales (all MALVs together) based on synapomorphies, their monophyly is not always supported by SSU analysis and more phylogenetic analysis is required. *Amoebophrya, Duboscquella, Merodinium, Syndinium*. *Incertae sedis* Syndiniales: *Ichthyodinium* (environmental Marine Alveolate (MALV) Group I). Parasites of the early developmental stages (eggs and larvae) of some species of finfish; sporogenesis occurs in the yolk sac of embryos. *Incertae sedis* Syndiniales: Ellobipsidae: the clade is closely related to MALV‐I and MALV‐II. *Ellobiopsis*,* Thalassomyces*. ••••Euduboscquellidae Coats & Bachvaroff 2012 Parasites of Ciliophora and Radiolaria, with trophont episome bordered by a perinematic ring and a lamina pharyngea extending into trophont cytoplasm; sporogenesis occurs outside the host (or outside the central capsule). *Euduboscquella* (environmental Marine Alveolate Group I), *Dogelodinium, Keppenodinium*. *Incertae sedis*: RP‐parasite, parasite of copepods (environmental Marine Alveolate Group I). ••••Amoebophryidae Cachon 1964 (environmental Marine Alveolate Group II) Parasites of Dinophyceae, Radiolaria and Ciliophora; feeding through a cytopharynx inside the host; palintomic schizogonous divisions occur inside the host and produce a swimming multicellular temporary structure (termed vermiform) after an evagination to leave its host, then each cell of this structure individualize into dinospore(s). *Amoebophrya*. ••••Syndinidae Chatton 1910 (environmental Marine Alveolate Group IV) Parasites of crustacean and Radiolaria. Sporogenesis occurs inside the host, and start by active nucleic divisions into a plasmodium that generally fill the whole body of the host; sporogenesis occurs inside the host by fragmentation of the plasmodium. *Hematodinium, Syndinium, Solenodinium*. ••••Sphaeriparaceae Loeblich III 1970 Parasites of Appendicularia and Radiolaria; feeding through a cytopharynx inside the host; sporogenesis occurs outside the host. *Atlanticellodinium, Sphaeripara*. •••Noctilucales Haeckel 1894 [Noctiluciphyceae Fensome et al. 1993] Principal life cycle stage comprising a large free‐living motile cell inflated by vacuoles; dinokaryon during part of life cycle only. Fossils unknown. *Abedinium, Cachonodinium, Craspedotella, Cymbodinium, Kofoidinium, Leptodiscus, Noctiluca, Petalodinium, Pomatodinium, Scaphodinium, Spatulodinium*. •••Dinophyceae Pascher 1914 Cell cortex (amphiesma) containing alveolae (amphiesmal vesicles) that may or may not contain cellulosic thecal plates, the pattern (tabulation) thus formed being a crucial morphological criterion in recognizing affinities among dinophyceans; with a dinokaryon through the entire life cycle. ••••Gymnodiniphycidae Fensome et al. 1993 With numerous amphiesmal vesicles, arranged nonserially or in more than six latitudinal series or with the pellicle as the principal amphiesmal element or the amphiesmal structure uncertain but not comprising a theca divisible into six or fewer latitudinal plates. Few known fossil representatives. •••••*Gymnodinium* F. Stein 1878, emend. G. Hansen & Moestrup in Daugbjerg et al. 2000 sensu stricto With loop‐shaped, anticlockwise apical structure complex. *Barrufeta, Chytriodinium*,* Dissodinium, Erythropsidinium, Greuetodinium, Gymnodinium, Lepidodinium, Nematodinium, Nusuttodinium, Pellucidodinium, Polykrikos, Proterythropsis, Warnowia*. •••••*Amphidinium* Claparède & Lachmann 1859, emend. Flø Jørgensen et al. 2004 sensu *stricto* Minute irregular triangular‐ or crescent‐shaped episome deflected to the left; no apical structure complex. *Amphidinium*. •••••*Gyrodinium* Kofoid & Swezy 1921, emend. G. Hansen and Moestrup in Daugbjerg et al. 2000 sensu *stricto* Elliptical apical structure complex, bisected by a ridge; with surface striation/ridges. *Gyrodinium*. •••••Kareniaceae Bergholtz et al. 2005 Furrow‐like straight or sigmoid apical structure complex; haptophyte‐derived chloroplasts. *Brachidinium, Karenia, Karlodinium, Takayama*. •••••Ceratoperidiniaceae Loeblich III 1980, emend. Reñé and de Salas 2013 Closed, circular loop‐shaped apical structure complex. *Ceratoperidinium*,* Kirithra*. •••••Torodiniales Boutrup, Moestrup & Daugbjerg 2016 Hat‐like apical projection; counterclockwise spiralling apical structure complex; with longitudinal surface striation; very large episome. *Kapelodinium, Torodinium* •••••*Levanderina* Moestrup et al. 2014 U‐shaped apical structure complex. *Levanderina*. •••••*Margalefidinium* F. Gómez, Richlen & D.M. Anderson 2017 U‐shaped apical structure complex connected to the sulcal extension; sulcus encircles the cell about once; cingulum encircles the cell about twice; smooth cell surface; eyespot in the episome. *Margalefidinium* •••••*Cochlodinium* F. Schütt emend. F. Gómez, Richlen & D.M. Anderson 2017 sensu *stricto* circular apical structure complex connected to the sulcal extension; sulcus invades the episome as a wide loop; descending cingulum encircles the cell about 1.5–2 times; cell surface with fine striations. *Cochlodinium strangulatum*. •••••Ptychodiscales Fensome et al. 1993 With wall of the motile cell dominated by a thick pellicle; alveolae, where discernible, usually devoid of thecal plates, forming a tabulation that is nonserially arranged or is organized into numerous latitudinal series. Few known fossil representatives. *Achradina, Amphilothus, Balechina, Ptychodiscus, Sclerodinium*. •••••Borghiellaceae Moestrup, Lindberg, & Daugbjerg 2009 With or without apical pair of elongate vesicles (PEV) with furrow; eyespot type B with globules inside the chloroplast associated with external brick‐like material in vesicles. *Baldinia, Borghiella*. •••••Tovelliaceae Moestrup et al. 2005 With alveolae containing light thecal plates with apical line of narrow plates (ALP); eyespot type C with extraplastidal, nonmembrane bound pigment globules. *Bernardinium, Esoptrodinium, Jadwigia, Tovellia*. •••••Suessiaceae Fensome et al. 1993, emend. Moestrup et al. 2009 With alveolae containing light thecal plates and forming a tabulation involving 7–15 latitudinal series, with or without elongate apical vesicle (EAV); eyespot type E with cisternae containing brick‐like material. *Ansanella, Asulcocephalium, Biecheleria, Biecheleriopsis, Leiocephalium, Pelagodinium, Polarella, Prosoaulax, Protodinium, Symbiodinium, Yihiella*. ••••Peridiniphycidae Fensome et al. 1993 With a tabulation that accords with, or derives from, a pattern in which there are five or six latitudinal plate series; sagittal suture lacking. •••••Gonyaulacales Taylor 1980 Alveolae usually containing thecal plates, forming a tabulation of 5–6 latitudinal series; distinguished by particular tabulation features that can be recognized generally by a strong degree of asymmetry in the anterior (apical) and posterior (antapical) areas. This taxon has fossils extending from the late Triassic period (˜210 Ma) to the present day. *Alexandrium, Amylax, Ceratium, Ceratocorys, Coolia, Fukuyoa, Fragilidium, Gambierdiscus, Goniodoma, Gonyaulax, Lingulodinium, Ostreopsis, Pentaplacodinium, Peridiniella, Protoceratium, Pyrocystis, Pyrodinium, Pyrophacus, Tripos*. •••••Peridiniales Haeckel 1894 Alveolae containing thecal plates, forming a tabulation of 6 latitudinal series; distinguished by particular tabulation features that can be recognized generally by a strong degree of symmetry in the anterior (apical) and posterior (antapical) areas. This taxon has fossils extending from the early Jurassic Period (˜190 Ma) to the present day. Amphidiniopsis, Archaeperidinium, Blastodinium*, Diplopelta, Diplopsalis, Diplopsalopsis, Herdmania, Niea, Oblea, Palatinus, Parvodinium, Peridinium, Peridiniopsis, Preperidinium, Protoperidinium, Qia, Vulcanodinium. •••••Thoracosphaeraceae Schiller 1930 Calcareous species and non‐calcareous relatives. *Aduncodinium, Amyloodinium, Apocalathium, Blastodinium, Chimonodinium, Cryptoperidiniopsis, Duboscquodinium, Ensiculifera, Leonella, Luciella, Naiadinium, Paulsenella, Pentapharsodinium, Pfiesteria, Scrippsiella, Stoeckeria, Theleodinium, Thoracosphaera, Tintinnophagus, Tyrannodinium*. •••••Podolampadaceae Lindemann 1928 No compressed cingulum but homologous plate series. *Blepharocysta, Gaarderiella, Heterobractum, Lissodinium, Mysticella, Podolampas*. •••••Kryptoperidiniaceae Lindemann 1925 (= “dinotoms”) Genera with diatom‐derived chloroplasts and reduced peridinin‐chloroplast as eyespot; thecate and athecate taxa, e.g. *Blixaea, Durinskia, Galeidinium, Kryptoperidinium, Unruhdinium*. •••••*Heterocapsaceae* Fensome et al. 1993 Alveolae containing thecal plates, forming a tabulation of six latitudinal series; scales. *Heterocapsa*. •••••Amphidomataceae Sournia 1984 Alveolae containing thecal plates, forming a tabulation of 5‐6 latitudinal series. *Amphidoma, Azadinium*. •••••Oxytoxaceae Lindemann 1928 Alveolae containing thecal plates, forming a tabulation of 5‐6 latitudinal series; one antapical plate. *Corythodinium, Oxytoxum*. •••••Centrodiniaceae Hernández‐Becerril 2010 Alveolae containing thecal plates, forming a tabulation of 7 latitudinal series; one antapical plate. *Centrodinium*. ••••Dinophysales Kofoid 1926 With a cingulum, a sulcus, and a sagittal suture extending the entire length of the cell, one ciliary pore. Apart from one possible fossil representative, only known from present day forms. *Amphisolenia, Citharistes, Dinofurcula, Dinophysis, Histioneis, Latifascia, Metadinophysis, Metaphalacroma, Ornithocercus, Oxyphysis, Parahistioneis, Phalacroma, Pseudophalacroma, Sinophysis, Triposolenia*. ••••Prorocentrales Lemmermann 1910 Without cingulum or sulcus; one ciliary pore; cilia apical—one wavy cilium clearly homologous with transverse cilium of other dinoflagellates and one not wavy; thecal plates. Fossils unknown. *Mesoporus, Prorocentrum*. ••Apicomplexa Levine 1980, emend. Adl et al. 2005 At least one stage of the life cycle with flattened subpellicular vesicles and an apical complex consisting of one or more polar rings, rhoptries, micronemes, conoid and subpellicular microtubules; sexuality, where known, by syngamy followed by immediate meiosis to produce haploid progeny; asexual reproduction of haploid stages occurring by binary fission, endodyogeny, endopolyogeny and/or schizogony/merogony; locomotion by gliding, body flexion, longitudinal ridges and/or cilia; mostly parasitic. *Incertae sedis* Apicomplexa: Agamococcidiorida Levine 1979 Merogony and gametogony both absent; several families described but position within Apicomplexa unclear. Gemmocystis*, Rhytidocystis. *Incertae sedis* Apicomplexa: Protococcidiorida Kheisin 1956 Merogony absent; extracellular gamogony and sporogony; in some species, gamogony and fertilization in the host, with oocysts released with sporogony in aqueous environment; sporozoites exist inside intestinal epithelium briefly, on their way to coelom or vascular tissues, where development occurs, followed by sporozoite release in the faeces. Subdivisions uncertain. Angeiocystis*, Coelotropha*, Grellia*, Eleutheroschizon*, Mackinnonia*, Myriosporides*, Myriospora*, Sawayella*. Incertae sedis Apicomplexa: *Aggregata* Frenzel 1885—highly divergent 18S rRNA. *Christalloidophora* Dehorne, 1934—Merozoites with 2–4 fuchsinophile crystalloids. Dobellia* Ikeda, 1914—Life cycle still confused, sporozoites invade intestinal epithelial cells, two forms of meronts: macromeront with period of intranuclear development, micromeront epicellular, syzygy of macro‐ and micromerozoites producing gametocysts, oocyst with 1000 naked sporozoites. Echinococcidium* Porchet 1978—with single spines and spines arranged in bundles forming a pyramid on body surface, amylopectin granules, in large parasitoporous vacuole in intestinal epithelium or free in intestinal lumen. Globidiellum* Brumpt 1913—intracellular in mononuclear leucocytes, endothelial cells and erythrocytes, formation of large number of merozoites. Joyeuxella* Brasil 1902—Merogony in host epithelial cells. Rhabdospora* Laguesse 1906—Meronts in host epithelial cells, merozoites with rodlets (rhoptries?). Spermatobium* Eisen 1895—Young stages in sperm sac cells, later stages free in sperm sac, sporogonia (oocysts?), sporoblasts (sporocysts?) and residuum described. Spiriopsis* Arvy and Peters 1972—with micronemes and paraglycon. Spirogregarina* Wood and Herman 1943—Elongate, spindle‐shaped, cylindrical, with spiral bands (grooves, epicytic folds?) on surface, bulbous, knob‐like terminus at each end. Toxocystis* Léger and Duboscq 1910—elongate, falciform with one broader end, nucleus with two paranuclear bodies opposite each other, not motile. Trophosphaera* Le Calvez 1939—Spherical cysts with spherical cytomeres, cytomeres form spherical sporocytes (schizogony), sporocytes form eight naked spores. •••Aconoidasida Mehlhorn et al. 1980 [= Hematozoa Vivier 1982] (P) Apical complex lacking conoid in asexual motile stages; some diploid motile zygotes (ookinetes) with conoid; macrogametes and microgametes forming independently; heteroxenous. ••••Haemospororida Danilewsky 1885 Zygote motile as ookinete with conoid; ciliated microgametes produced by schizogonous process; oocyst formed in which sporozoites develop. Dionisia*, Haemocystidium, Haemoproteus, Hepatocystis, Leucocytozoon, Mesnilium*, Nycteria, Parahaemoproteus, Plasmodium, Polychromophilus, Rayella*, Saurocytozoon*. ••••Piroplasmorida Wenyon 1926 Piriform, round, rod‐shaped or amoeboid; conoid and cilia absent in all stages; polar ring present; without oocyst; sexuality probably associated with the formation of large axopodium‐like “Strahlen”. Anthemosoma*, Babesia, Cytauxzoon, Echinozoon*, Haemohormidium*, Sauroplasma*, Serpentoplasma*, Theileria. ••••Nephromycida Cavalier‐Smith 1993, emend. Adl et al. 2019 Aciliate, motile infective stage (resembling sporozoites); spore‐stage with rhoptry‐like inclusions, biciliated stages exist; most of life cycle extracellular; symbionts/parasites of marine invertebrates (at least tunicates). *Nephromyces, Cardiosporidium*. •••Conoidasida Levine 1988 (P) Complete apical complex, including a closed conoid in all or most asexual motile stages; cilia, where present, found exclusively in microgametes (male gametes); with the exception of microgametes, motility generally via gliding with possibility of body flexion and undulation of longitudinal pellicular ridges; heteroxenous or homoxenous. This group is not monophyletic with subdivisions artificial and unclear at this time. ••••Coccidia Leuckart 1879 (P) Mature gametes develop intracellularly; microgamont typically produces numerous microgametes; syzygy absent; zygote rarely motile; sporocysts usually formed within oocysts. •••••Adeleorina Léger 1911 Microgamonts produce one to four microgametes, which associate with macrogamete in syzygy; endodyogony is absent. Adelea*, Adelina, Babesiosima*, Bartazoon*, Chagasella*, Cyrilia*, Dactylosoma, Desseria*, Ganapatiella*, Gibbsia*, Haemogregarina, Haemolivia, Hepatozoon, Ithania*, Karyolysus, Klossia, Klossiella*, Legerella*, Orcheobius*, Rasajeyna*. •••••Eimeriorina Léger 1911 Microgametes and macrogametes develop independently; syzygy is absent; microgamonts produce large number of cilated microgametes; zygote is nonmotile; sporozoites always enclosed in sporocyst within oocyst. Atoxoplasma, Barrouxia*, Besnoitia, Caryospora, Caryotropha, Choleoeimeria, Cyclospora, Cystoisospora, Defretinella*, Diaspora*, Dorisa*, Dorisiella*, Eimeria, Elleipsisoma*, Goussia, Hammondia, Hyaloklossia, Isospora, Lankesterella, Mantonella*, Merocystis, Neospora, Nephroisospora, Ovivora*, Pfeifferinella, Pseudoklossia, Sarcocystis, Schellackia, Selenococcidium*, Selysina*, Spirocystis*, Toxoplasma, Tyzzeria*, Wenyonella*. ••••Gregarinasina Dufour 1828 (P) Mature gamonts usually develop extracellularly; syzygy of gamonts generally occurring with production of gametocyst; similar numbers of macrogametes and microgametes maturing from paired gamonts in syzygy within the gametocyst; syngamy of mature gametes leading to gametocyst that contains few to many oocysts, which each contain sporozoites; sporocysts absent; asexual reproduction (merogony) absent in some species. *Incertae sedis* Gregarinasina: *Digyalum* Koura et al. 1990—only transverse epicytic folds, two pouches at anterior end; *Exoschizon* Hukui 1939—epicytic folds, schizogony at anterior end of trophozoite, cytoplasmic buds, 16 merozoites differentiated at anterior end of trophozoite. •••••Archigregarinorida Grassé 1953 (P) Trophozoite aseptate; with syzygy; encystment of gamonts; oocysts contain 4–8 or even more sporozoites. *Filipodium, Meroselenidium, Merogregarina, Platyproteum, Selenidium, Selenocystis, Veloxidium*. •••••Eugregarinorida Léger 1900 (P) Trophozoite with epimerite in gregarines with septum or mucron in gregarines without septum; syzygy followed by encystment of gamonts; oocysts with 8 sporozoites. Apolocystis*, Amoebogregarina, Ancora, Ascogregarina, Asterophora, Blabericola, Caliculium, Cephaloidophora, Colepismatophila, Cystobia*, Cystocephalus*, Difficilina, Diplauxis*, Enterocystis, Ganymedes, Geneiorhynchus, Gonospora, Gregarina, Heliospora, Hentschelia*, Hirmocystis*, Hoplorhynchus, Lankesteria, Lecudina, Leidyana, Lithocystis, Monocystella*, Monocystis, Nematopsis, Nematocystis*, Neoasterophora*, Paralecudina, Paraschneideria, Phyllochaetopterus, Pileocephalus*, Polyplicarium, Polyrhabdina, Porospora*, Prismatospora, Protomagalhaensia, Psychodiella, Pterospora, Pyxinia, Pyxinioides, Rhabdocystis*, Sphaerorhynchus*, Steinina, Stenophora, Stylocephalus, Sycia*, Syncystis, Thalicola*, Thiriotia, Trichotokara, Uradiophora*, Urospora, Xiphocephalus, Zygosoma* (examples). •••••Neogregarinorida Grassé 1953 Trophozoite with epimerite or mucron; multiple rounds of schizogony/merogony; pairing of gamonts; oocysts contain eight sporozoites. Apicystis, Aranciocystis, Caulleryella*, Coelogregarina*, Farinocystis*, Gigaductus*, Lipocystis*, Lipotropha*, Lymphotropha*, Machadoella*, Mattesia, Menzbieria, Ophryocystis, Schizocystis*, Syncystis, Tipulocystis*. •••••Cryptogregarinorida Cavalier‐Smith 2014, emend. Adl et al. 2019 Oocysts and meronts with attachment “feeder” organelle; anisogamous, microgametes aciliate; oocysts without sporocysts containing four naked sporozoites; epicellular localization in host cell. *Cryptosporidium*. ••••Blastogregarinea Chatton and Villeneuve 1936, emend. Simdyanov et al. 2018 Permanent multinuclearity and gametogenesis: nuclear division of merogony and gamogony proceeds within the same individual (merogamont) throughout its life span; merogamonts motile and sexually differentiated. Female oogamy: continuous budding of macrogametes from the posterior part of female merogamonts. Male gamogony: budding of multinuclear microgametocytes or microgametoblasts apparently followed by their dissociation into small putatively biciliated male gametes. Oocysts with many (10–16) free sporozoites (no sporocysts). *Chattonaria*,* Siedleckia*. ••Ciliophora Doflein 1901 [Ciliata Perty 1852; Infusoria Bütschli 1887] Cells with nuclear dimorphism, including a typically polygenomic macronucleus and at least one diploid micronucleus; somatic kinetids having a postciliary microtubular ribbon arising from triplet 9, a kinetodesmal fibril or striated rootlet homologue arising near triplets 5–8, and a transverse microtubular ribbon arising in the region of triplets 4–6; sexual reproduction, when present, by conjugation typically with mutual exchange of haploid gametic nuclei that fuse to form the synkaryon or zygotic nucleus.^35^ •••Postciliodesmatophora Gerassimova & Seravin 1976 Somatic dikinetids with postciliodesmata (overlapping postciliary microtubular ribbons separated by one or two microtubules). ••••Karyorelictea Corliss 1974 Two to many macronuclei containing approximately but sometimes slightly more than the diploid amount of DNA; macronuclei not dividing but replaced by division of micronuclei; major postciliary ribbons separated by two groups of microtubules. •••••Kentrophoridae Jankowski 1980 (*Kentrophoros)* •••••Loxodida Jankowski 1980 Non‐contractile; somatic cilia as files of dikinetids mainly on the right surface with the left surface non‐ciliated, except for single marginal (‘bristle’?) kinety; extrusomes as somatic cnidocyst‐like organelles in some genera; oral kinetids as two dikinetidal perioral kineties and one intraoral (intrabuccal) kinety; stomatogenesis monoparakinetal or buccokinetal; nuclei in clusters, typically two macronuclei and one micronucleus; typically in anoxic sediments and anoxic waters. ••••••Cryptopharyngidae Jankowski 1980 (*Cryptopharynx)* ••••••Loxodidae Bütschli 1889 (*Loxodes)* •••••Geleiidae Kahl 1933 (*Geleia)* ••••Heterotrichea Stein 1859 Polygenomic macronucleus dividing by extra‐macronuclear microtubules; major postciliary ribbons separated by one microtubule. •••••Blepharismidae Jankowski in Small & Lynn 1985 (*Blepharisma)* •••••Climacostomidae Repak 1972 (*Climacostomum)* •••••Condylostomatidae Kahl in Doflein & Reichenow 1929 (*Chattonidium*,* Condylostoma)* •••••Fabreidae Shazib et al. 2014 (*Fabrea)* •••••Gruberiidae Shazib et al. 2014 (*Gruberia)* •••••Coliphorina Jankowski 1967 With a node‐based definition: the clade stemming from the most recent common ancestor of the Maristentoridae and Folliculinidae. ••••••Folliculinidae Dons 1914 (*Folliculina)* ••••••Maristentoridae Miao et al. 2005 (*Maristentor)* •••••Peritromidae Stein 1867 (*Peritromus)* •••••Spirostomidae Stein 1867 (*Anigsteinia*,* Spirostomum)* •••••Stentoridae Carus 1863 (*Stentor)* •••Intramacronucleata Lynn 1996 Polygenomic macronucleus dividing by intramacronuclear microtubules. *Incertae sedis* Intramacronucleata: *Protocruzia* Faria da Cunha and Pinto 1922 [Protocruziidae Jankowski, 1980; Protocruziidia de Puytorac et al., 1987] Nuclear apparatus a cluster of similar‐sized nuclei with paradiploid macronuclei surrounding one or more micronuclei; each macronucleus possibly organized as a single composite chromosome. *Protocruzia*. ••••SAL Gentekaki et al. 2014 Group identified by phylogenomics. With a node‐based definition: the clade stemming from the most recent common ancestor of the Spirotrichea (S) and Lamellicorticata (i.e. Armophorea (A) and Litostomatea (L)). *Incertae sedis* SAL: *Cariacothrix* Orsi et al. 2012 [Cariacotrichea Orsi et al. 2011] With archway‐shaped kinety around oral opening and extending to posterior body end; with unique molecular signature ‘GAAACAGUCGGGGGUAUCAGUA’ (spanning nucleotide positions 283‐305 in GenBank accession number GU819615); confirmed only from deep waters of anoxic Cariaco Basin, Venezuela. At least two adoral organelles; longitudinal ciliary rows; caudal cilia. *Cariacothrix caudata*. *Incertae sedis* SAL: Mesodiniidae Jankowski, 1980 Somatic cilia bristle‐like, of at least two types, arranged in girdles around the body; brosse kineties absent; extrusomes as oral toxicysts; oral region apical, domed, circular, and delimited by circumoral dikinetids, but apparently without nematodesmata and bulge microtubules of rhabdos. *Mesodinium*. *Incertae sedis* SAL: *Phacodinium* Prowazek 1900 [Phacodiniidia Small and Lynn 1985] Somatic kineties as linear polykinetids; each kinetosome bearing a kinetodesmal fibril, and sometimes accompanied by a partner kinetosome in some regions of the body, thus resembling a cirrus. *Phacodinium*. •••••Spirotrichea Bütschli 1889 Conspicuous right and left oral and/or preoral ciliature; left serial oral polykinetids leading (usually clockwise) into the oral cavity, either around a broad anterior end or along anterior and left margins of the body; DNA replication in the macronucleus accomplished by a complicated migrating structure called replication band; extensive chromosomal fragmentation. ••••••Euplotia Jankowski 1979 Adoral zone usually with numerous polykinetids (paramembranelles); body dorsoventrally flattened; right preoral ciliature as paroral and/or endoral with diplo‐ or polystichomonad structure; somatic ciliature usually in dikinetidal rows on dorsal side and forming cirri on ventral side; somatic dikinetids with cilia at anterior basal bodies and retention of kinetodesmal fibrils; stomatogenesis generally apokinetal, sometimes hypoapokinetal or parakinetal; turn‐over or replacement of only ventral somatic infraciliature; typically no resorption of all kinetosomes in cysts. •••••••Euplotida Small & Lynn 1985 Hypoapokinetal stomatogenesis in subsurface tube. ••••••••Aspidiscidae Ehrenberg 1830 (*Aspidisca)* ••••••••Certesiidae Borror & Hill 1995 (*Certesia)* ••••••••Euplotidae Ehrenberg 1838 P (*Euplotes)* ••••••••Gastrocirrhidae Fauré‐Fremiet 1961 (*Gastrocirrhus)* ••••••••Uronychidae Jankowski 1975 (*Diophrys*,* Uronychia)* •••••••Discocephalida Wicklow 1982 Left and right marginal rows form intrakinetally; epiapokinetal stomatogenesis; left most frontal cirrus originates from anterior end of undulating membrane‐anlage; many frontoventral‐transverse cirral anlagen; dorsal kinety anlagen formed in secondary mode; caudal cirri originate from rightmost dorsal kineties anlagen by multisegmentation mode; development of frontoventral‐transverse cirral anlagen of primary type; migrating cirri are not formed, which are always derived from the right most cirral anlage in all traditional hypotrichs; anlage of undulating membranes splits transversely to form endoral and paroral membranes. ••••••••Discocephalidae Jankowski 1979 (*Discocephalus*,* Prodiscocephalus*,* Paradiscocephalus)* ••••••••Pseudoamphisiellidae Song et al. 1997 (*Leptoamphisiella*,* Pseudoamphisiella)* ••••••Perilemmaphora Berger 2008 Perilemma present. Possibly, comprises also *Diophrys* and the discocephalids. •••••••Hypotrichia Stein 1859 Adoral zone usually with numerous polykinetids (paramembranelles) along anterior and left margins of dorsoventrally flattened body, rarely around broad apical end; right preoral ciliature as paroral and/or endoral, paroral diplo‐ or polystichomonad, endoral mono‐, rarely diplostichomonad; somatic ciliature usually in dikinetidal rows on dorsal side and forming cirri on ventral side, rarely with jumping bristles instead of kineties and cirri; somatic dikinetids with cilia at anterior basal bodies and loss of kinetodesmal fibril; stomatogenesis epiapokinetal or parakinetal; complete turn‐over or replacement of ventral and dorsal somatic ciliature; kinetosome‐resorbing cysts. ••••••••Stichotrichida Fauré‐Fremiet 1961 (P) Ventral cirri as one or more longitudinal and linear (not zig‐zag as in Urostylida) files or as frontoventral cirri, typically conspicuous, arranged in specific, localized frontal and ventral groups (i.e. sporadotrichs); dorsal ciliature typically regularly distributed in longitudinal files; stomatogenesis parakinetal or apokinetal, if apokinetal, may occur with five or six anlagen streaks in two groups for differentiation of ventral somatic ciliature (i.e. sporadotrichs). Note that this taxon is artificial. Many of the families listed here alphabetically are not monophyletic and have limited support from molecular phylogenetics of small subunit rRNA. •••••••••Amphisiellidae Jankowski 1979 (*Amphisiella*,* Bistichella)* •••••••••Atractosidae Bourland 2015 (*Atractos)* •••••••••Epiclintidae Wicklow & Borror 1990 (*Epiclintes)* •••••••••Gonostomatidae Small & Lynn 1985 (*Cotterillia, Gonostomum)* •••••••••Halteriidae Claparède and Lachmann 1858 (*Halteria*,* Meseres)* •••••••••Holostichidae Fauré‐Fremiet 1961 (*Holosticha, Uncinata)* •••••••••Kahliellidae Tuffrau 1979 (*Deviata*,* Kahliella*) •••••••••Keronidae Dujardin 1840 (*Kerona*) •••••••••Oxytrichidae Ehrenberg 1830 (P) (*Cyrtohymena*,* Gastrostyla*,* Oxytricha*,* Stylonychia)* •••••••••Parabirojimidae Dai & Xu 2011 (*Parabirojimia*,* Tunicothrix)* •••••••••Plagiotomidae Bütschli 1887 (*Plagiotoma)* •••••••••Psammomitridae Jankowski 1979 (*Psammomitra)* •••••••••Pseudoamphisiellidae Song et al. 1996 (*Pseudoamphisiella)* •••••••••Psilotrichidae Bütschli 1889 (*Psilotricha, Urospinula)* •••••••••Schmidingerotrichidae Foissner 2012 (*Schmidingerothrix)* •••••••••Spirofilidae von Gelei 1929 (*Spirofilopsis*,* Strongylidium)* •••••••••Trachelostylidae Small & Lynn 1985 (*Trachelostyla)* •••••••••Uroleptidae Foissner & Stoeck 2008 (*Paruroleptus*,* Uroleptus)* ••••••••Urostylida Jankowski 1979 (P) Somatic ventral ciliature as frontoventral cirri in zig‐zag files, running almost the full length of ventral surface between right and left files of marginal cirri, and ranging from a “single” file of zig‐zag or offset cirri to multiple and short files of cirri whose anterior and sometimes posterior ends are offset (= developed zig‐zag) (e.g. *Eschaneustyla*); transverse cirri may be present; caudal cirri may be present; during division morphogenesis, zig‐zag cirri differentiating from anlagen of many short oblique kinetofragments. •••••••••Bergeriellidae Liu et al. 2010 (*Bergeriella, Neourostylopsis)* •••••••••Hemicycliostylidae Lyu et al. 2018 (*Australothrix, Hemicycliostyla)* •••••••••Pseudokeronopsidae Borror & Wicklow, 1983 (*Apoholosticha*,* Pseudokeronopsis)* •••••••••Pseudourostylidae Jankowski 1979 (*Pseudourostyla)* •••••••••Urostylidae Bütschli 1889 (*Bakuella, Diaxonella*,* Urostyla)* •••••••Oligotrichea Bütschli 1887 Adoral zone around broad apical cell end, usually composed of large collar and small buccal membranelles, C‐shaped, circular or with secondary ventral gap; endoral monostichomonad; naked or with lorica; somatic ciliature in one to many rows arranged around body; hypoapokinetal stomatogenesis; enantiotropic cell division. ••••••••Oligotrichida Bütschli 1887 Adoral zone C‐shaped, polykinetids become smaller towards cytostome; naked; rarely with contractile tail that might be lost secondarily; usually two somatic kineties that might be fragmented; somatic dikinetids with a cilium only at the anterior or left basal body; needle‐shaped extrusomes (trichites); usually with polysaccharidic cortical platelets; hypoapokinetal stomatogenesis in a subsurface tube. •••••••••Cyrtostrombidiidae Agatha 2004 (*Cyrtostrombidium)* •••••••••Pelagostrombidiidae Agatha 2004 (*Pelagostrombidium)* •••••••••Strombidiidae Fauré‐Fremiet 1970 (*Strombidium)* (P) •••••••••Tontoniidae Agatha 2004 (*Laboea*,* Tontonia)* ••••••••Choreotrichida Small & Lynn 1985 Adoral zone circular, rarely with secondary ventral gap; collar polykinetids extend across peristomial rim, proximalmost ones elongated, extending into buccal cavity; naked or with lorica; structure of somatic kinetids highly diverse even within single specimens (monokinetids, biciliated dikinetids, dikinetids with single cilium at anterior or posterior basal body); somatic ciliature in one to many longitudinal or curved rows arranged around entire body; hypoapokinetal stomatogenesis in a subsurface pouch; enantiotropic cell division less pronounced (oral primordium parallel to lateral cell surface). *Incertae sedis* Choreotrichida: *Lynnella* Liu et al. 2011 [Lynnellidae Liu et al. 2011; Lynnellida Liu et al. 2015] (M) Sistergroup relationship of this monotypic genus and order changes depending on the algorithms used in molecular approaches. Morphological and ontogenetic data suggest an affiliation with the aloricate choreotrichids: proximalmost collar membranelles elongated, extending into buccal cavity; two longitudinal somatic kineties both of which with distinctly derived kinetid structures: one is monokinetidal, the other composed of dikinetids with cilia only at the posterior basal bodies; endoral membrane extends across peristomial field in middle dividers; stomatogenesis in subsurface pouch; oral primordium parallel to cell surface; two macronuclear nodules. Adoral zone with (probably secondary) ventral gap. *Lynnella semiglobulosa*. •••••••••Strobilidiina Jankowski 1980 (P) Adoral zone circular, rarely with secondary ventral gap; naked. ••••••••••Leegaardiellidae Lynn & Montagnes 1988 (*Leegaardiella)* ••••••••••Lohmanniellidae Montagnes & Lynn 1991 (*Lohmanniella)* ••••••••••Strobilidiidae Kahl in Doflein & Reichenow 1929 (*Strobilidium)* ••••••••••Strombidinopsidae Small & Lynn 1985 (*Strombidinopsis)* (P) •••••••••Tintinnina Kofoid & Campbell 1929 Adoral zone circular; with hyaline, entirely or partially agglutinated lorica; somatic ciliature arranged in specialized fields and rows; frequently with extrusive capsules; with contractile peduncle. Molecular phylogenies contribute to far‐reaching revision, but taxon coverage is insufficient, and tree topology is unsettled. *Incertae sedis* Tintinnina: *Helicostomella*,* Tintinnopsis* (P), plus several further genera. ••••••••••Ascampbelliellidae Corliss 1960 (*Ascampbelliella)* ••••••••••Cyttarocylididae Kofoid and Campbell 1929 (*Cyttarocylis)* (Probably, synonymous with Petalotrichidae) ••••••••••Dictyocystidae Haeckel 1873 (*Dictyocysta)* ••••••••••Epiplocylididae Kofoid & Campbell 1939 (*Epiplocylis)* ••••••••••Eutintinnidae Bachy et al. 2012 (*Dartintinnus, Eutintinnus)* ••••••••••Favellidae Kofoid & Campbell 1929 (*Favella)* ••••••••••Nolaclusiliidae Sniezek et al. 1991 (*Nolaclusilis)* ••••••••••Petalotrichidae Kofoid & Campbell 1929 (*Petalotricha)* (Probably, synonymous with Cyttarocylididae) ••••••••••Ptychocylididae Kofoid & Campbell 1929 (*Cymatocylis*,* Ptychocylis)* ••••••••••Rhabdonellidae Kofoid & Campbell 1929 (*Metacylis*,* Rhabdonella*,* Schmidingerella* ••••••••••Stenosemellidae Campbell 1954 (P) (*Stenosemella)* ••••••••••Tintinnidae Claparède & Lachmann 1858 (*Amphorellopsis*,* Salpingacantha*,* Salpingella*,* Tintinnus)* ••••••••••Tintinnidiidae Kofoid & Campbell 1929 (*Tintinnidium)* ••••••••••Undellidae Kofoid & Campbell 1929 (*Undella)* ••••••••••Xystonellidae Kofoid & Campbell 1929 (*Dadayiella*,* Parafavella*,* Xystonella)* ••••••Licnophoridae Bütschli 1887 Body hour‐glass shaped, both ends discoidal; posterior disc adhesive, with peripheral rings of cilia anterior disc with serial oral polykinetids around oral region; ectosymbionts, temporarily attached to substrate or host by ciliated mobile, posterior adhesive disc. *Licnophora*,* Prolicnophora*. ••••••Kiitrichidae Nozawa 1941 With weakly differentiated and non‐grouped somatic ature, i.e. cirri on ventral side generally uniform, no clearly defined marginal cirral rows, cirri mixed with dikinetids on dorsal side, i.e. no clearly differentiated dorsal kineties. *Caryotricha*,* Kiitricha*. •••••Lamellicorticata Vd'ačný et al. 2010 Postciliary microtubular ribbons arranged in a single layer right of and between kineties; oral apparatus composed of a dikinetidal paroral membrane and several multirowed adoral membranelles; somatic dikinetids typically very narrowly spaced in anterior body portion; stomatogenesis telokinetal, commencing in dorsal or dorsolateral somatic kineties, and with migrating oral kinetofragments. ••••••Armophorea Lynn 2004 (R) Somatic dikinetids; energy‐generating organelles as anaerobic mitochondria or hydrogenosomes; contain cytoplasmic endosymbiotic methanogenic bacteria; extensive chromosomal fragmentation; stomatogenesis typically pleurokinetal; in oxygen‐depleted habitats. *Incertae sedis* Armophorea: Mylestomatidae Kahl in Doflein & Reichenow, 1929 (*Mylestoma)* •••••••Metopida Jankowski 1980 (P) Free‐living or endocommensal Armophorea with five or fewer perizonal stripe kineties. ••••••••Metopidae Kahl 1927 (P) Anterior part of body uniquely twisted to the left; with series of five perizonal somatic kineties. *Bothrostoma*,* Brachonella*,* Eometopus*,* Metopus*,* Parametopidium,Tesnospira*. ••••••••Apometopidae Foissner 2016 Obpyriform to clavate Metopida with perizonal ciliary stripe composed of four kineties. *Cirranter*,* Urostomides*. ••••••••Tropidoatractidae Rotterová et al. 2018 Preoral dome flattened, without torsion; cortex with interkinetal ridges; sparse, widely spaced somatic ciliature; short peristomium with deep, cup‐like buccal cavity; adoral zone reduced, more or less straight; perizonal stripe with five rows arranged in false kineties. *Palmarella*,* Tropidoatractus* •••••••Clevelandellida de Puytorac & Grain 1976 Body typically flattened; somatic dikinetids with non‐microtubular retrodesmal and cathetodesmal fibrils; oral polykinetids with a fourth row of kinetosomes directly opposite those of the third (heteromembranelles); paroral as diplostichomonad; macronucleus anchored in a karyophore in many species; conjugation often synchronized with reproductive life cycle of the host; endocommensals in digestive tracts of invertebrates and some vertebrates. ••••••••Clevelandellidae Kidder 1938 (*Clevelandella)* ••••••••Inferostomatidae Ky 1971 (*Inferostoma)* ••••••••Neonyctotheridae Affa'a 1987 (*Neonyctotherus)* ••••••••Nyctotheridae Amaro 1972 (P) (*Nyctotherus)* ••••••••Sicuophoridae Amaro 1972 (*Sicuophora)* •••••••Caenomorphidae Poche 1913 Body globular or conical, rigid, twisted to the left; somatic cilia as small kineties or cirrus‐like tufts; several oral polykinetids in small oral cavity in posterior cell half; paroral possibly absent; free‐swimming. Complex perizonal ciliary stripe comprising more than five kineties. Small subunit ribosomal DNA analysis suggests a sister group relationship with the Litostomatea. *Caenomorpha*,* Ludio*,* Sulfonecta*. •••••••Odontostomatida Sawaya 1940 Discoid, laterally compressed, wedge‐ or helmet‐shaped, typically nearly as wide as long, with armour‐like cuirass and often short posterior spines; somatic cilia arising from cortical pits; oral polykinetids inconspicuous, typically less than ten in number. ••••••••Discomorphellidae Corliss 1960 (*Discomorphella)* ••••••••Epalxellidae Corliss 1960 *(Epalxella, Saprodinium)* Note that *Epalxella* has been genetically characterized with Plagiopylea but *Saprodinium* with Armophorea. ••••••Litostomatea Small & Lynn 1981 Somatic monokinetids with two transverse ribbons, a slightly convergent postciliary ribbon, and a laterally directed kinetodesmal fibril that does not overlap those of adjacent kineties; one tangential transverse extending anteriorly into the somatic ridge to the left of the kinetid, one radial transverse ribbon extending transversely into the adjacent somatic ridge; one to several dorsal somatic kineties differentiated as brosse or brush kineties with specialized dikinetids bearing clavate *cilia lamina corticalis* or ecto‐endoplasmic fibrillar layer often present and well‐developed; oral ciliature as simple kinetids from which nematodesmata arise, supporting the cytopharynx, but nematodesmata may also arise from “oralized” somatic kinetids adjacent to the oral region; stomatogenesis telokinetal. •••••••Rhynchostomatia Jankowski 1980 Elongate to flask‐shaped body with dorsal proboscis of varying relative length; oral region circular or elliptical, possibly with permanent cytostome, distant from extreme anterior end of body, i.e. at base of the proboscis, but with right branch of circumoral kinety accompanied by at least one perioral kinety, extending along one border of the ventral surface of the proboscis, and with left branch of circumoral kinety accompanied by many oblique preoral kineties along the other border; toxicysts in the ventral band of the proboscis or distributed around the cytostome. ••••••••Dileptida Jankowski 1978 With a hybrid circumoral kinety; a staggered dorsal brush; and a stripe without cilia on the left side of the proboscis. •••••••••Dileptidae Jankowski 1980 (*Apodileptus*,* Dileptus*,* Pseudomonilicaryon)* •••••••••Dimacrocaryonidae Jankowski 1978 (*Dimacrocaryon, Monomacrocaryon*,* Rimaleptus)* ••••••••Tracheliidae Ehrenberg 1838 Body broad ovoid; proboscis immobile or only slightly mobile, with dorsal side distinctly shorter than ventral one; distinct groove (fossa) on right side containing and surrounded by condensed somatic ciliature; dorsal brush three‐ to four‐rowed and isoarchistichad; circumoral kinety dikinetidal throughout; internal oral basket clavate. *Trachelius*. •••••••Haptoria Corliss 1974 (P) Oral region typically circular or elliptical, with circumoral dikinetids whose microtubules support the cytostome‐cytopharynx; where circumoral dikinetids are absent, oralized somatic monokinetids bear nematodesmata forming the rhabdos; anterior condensation of dikinetidal cilia forming a dorsal brush, partially reduced; somatic kinetids otherwise monokinetidal after loss of anterior basal body; toxicysts; predatory life style. Questionable support for its monophyly as several members group with Trichostomatia, and its genera *Helicoprorodon* and/or *Trachelotractus* branch basally in the class Litostomatea. *Incertae sedis* Haptoria: *Chaenea*. ••••••••Lacrymariidae de Fromentel 1876 Anterior region of the body (=head) bulb‐like, covered by short oblique kineties with densely packed kinetids that abut the circumoral dikinetids. *Lacrymaria*. ••••••••Haptorida Corliss 1974 Oral region typically circular or elliptical, surrounded by circumoral dikinetids whose microtubules extend to support the cytostome‐cytopharynx; where circumoral dikinetids are absent, oralized somatic monokinetids bear nematodesmata forming the rhabdos. •••••••••Enchelyodonidae Foissner et al. 2002 (*Enchelyodon, Fuscheria)* •••••••••Homalozoonidae Jankowski 1980 (*Homalozoon)* •••••••••Pleuroplitidae Foissner 1996 (*Pleuroplites)* ••••••••Didiniidae Poche 1913 Somatic cilia as series of apparently short kinetofragments in one or more girdles around body, but in non‐ciliated regions, non‐ciliated kinetosomes are arranged in meridional kineties; brosse typically a field of clavate cilia or “sensory bristles” usually clearly detectable in 3–5 kineties. *Didinium, Monodinium*. ••••••••Pleurostomatida Schewiakoff 1896 Body leaf‐like or laterally compressed; free‐swimming, typically gliding on the substrate; somatic ciliation on both body sides, typically more dense on the right side; brosse dorsal, integrated in one or two dorsolateral kineties; oral region ventral and elongated, with oral kinetids as left and right components extending along the ventral edge of the laterally flattened body, bordering a slit‐like cytostome, surrounded by toxicysts; micronucleus lying between two macronuclear nodules; voracious cytotrophs. •••••••••Amphileptidae Bütschli 1889 (*Amphileptus)* •••••••••Litonotidae Kent 1882 (*Litonotus)* •••••••••Kentrophyllidae Wu et al. 2015 (*Epiphyllum, Kentrophyllum)* ••••••••Spathidiida Foissner & Foissner 1988 Somatic kineties curved anteriorly; three‐rowed dorsal brush. •••••••••Acropisthiidae Foissner & Foissner 1988 (*Acropisthium*,* Chaenea)* •••••••••Actinobolinidae Kahl 1930 (*Actinobolina)* •••••••••Apertospathulidae Foissner et al. 2005 (*Apertospathula)* •••••••••Enchelyidae Ehrenberg 1838 (*Enchelys)* •••••••••Pseudoholophryidae Berger et al. 1984 (*Pseudoholophrya)* •••••••••Spathidiidae Kahl in Doflein & Reichenow 1929 (*Spathidium)* •••••••••Trachelophyllidae Kent 1882 (*Trachelophyllum)* •••••••Helicoprorodontidae Small & Lynn 1985 Body elongate, vermiform, contractile; somatic ciliation holotrichous; 2–5 brosse kineties; toxicysts distributed along perioral ridge that makes from one to several turns around anterior end; oral region apical; marine psammobiont. *Helicoprorodon*. •••••••Trichostomatia Bütschli 1889 Oral region or oral cavity densely ciliated, sometimes organized as “polykinetids”; oralized somatic monokinetids; narrowly spaced somatic dikinetids form a clavate field; toxicysts absent; alveoli often filled with “skeletal” material; hydrogenosomes developed from mitochondria; stomatogenesis telokinetal, cryptotelokinetal in entodiniomorphids; typically anaerobic endosymbionts in vertebrates. ••••••••Vestibuliferida de Puytorac et al. 1974 Cortex often with thick microfilamentous layer between ecto‐ and endoplasm; oral region a depression or vestibulum, densely ciliated by extensions of somatic kineties whose cilia do not appear organized as “polykinetids”; endocommensals in fish and herbivorous placental mammals, except for marsupials. •••••••••Balantidiidae Reichenow in Doflein and Reichenow 1929 (*Balantidium, Neobalantidium)* (P) •••••••••Buetschliidae Poche 1913 (*Buetschlia)* •••••••••Paraisotrichidae da Cunha 1917 (*Paraisotrichia)* •••••••••Protocaviellidae Grain in Corliss 1979 (*Protocaviella)* •••••••••Protohalliidae da Cunha & Muniz 1927 (*Protohallia)* •••••••••Pycnotrichidae Poche 1913 (*Pycnothrix*, perhaps *Buxtonella*) •••••••Isoendo (R) Group identified by SSU rRNA phylogenies. With a node‐based definition: the clade stemming from the most recent common ancestor of the Isotrichidae (Iso), Entodiniomorphida (endo), and Macropodiniida. ••••••••Isotrichidae Bütschli 1889 Endoplasmic polysaccharide reserves; somatic mucocysts; oral cavity at or near posterior pole, lined by extensions of somatic kineties, with parental vestibulum migrating anteriorly during stomatogenesis to become the proter's vestibulum; macronucleus may be anchored by a karyophore; often endocommensals in ungulate ruminants. *Dasytricha*,* Isotricha*. ••••••••Entodiniomorphida Reichenow in Doflein & Reichenow 1929 Pellicle firm and thickened, often drawn out into posterior spines; cortex with thick microfilamentous layer between ecto‐ and endoplasm; somatic ciliature typically markedly reduced, appearing only in bands, zones or tufts, often as polybrachykineties, and functioning as syncilia oral area as only a slight depression to a deep one, often with well‐differentiated “polykinetids”; often commensals in mammalian host. •••••••••Blepharocorythina Wolska 1971 Somatic ciliation markedly reduced, as tufts and bands; presumed remnant of concrement vacuole complex present only as its overlying somatic kinetids. ••••••••••Blepharocorythidae Hsiung 1929 (*Blepharocorys)* ••••••••••Parentodiniidae Ito, Miyazaki & Imai 2002 (*Parentodinium)* ••••••••••Pseudoentodiniidae Wolska, 1986 (*Pseudoentodinium)* •••••••••Entodiniomorphina Reichenow in Doflein & Reichenow 1929 Somatic ciliature markedly reduced, appearing in tufts, sometimes elongated as spiralled bands, often arranged as polybrachykineties and functioning as syncilia; pellicle firm and thickened, often drawn out into spines; prominent skeletal plates in many species, composed of polysaccharide reserves (e.g. amylopectin granules or plaques); oral cilia often functioning as syncilia of two parts: a pre‐vestibular band in the peristomial region and a vestibular part(s) sensu stricto. ••••••••••Cycloposthiidae Poche 1913 (*Cycloposthium)* ••••••••••Gilchristidae Ito, Ishihara & Imai 2014 (*Gilchristia)* ••••••••••Ophryoscolecidae Stein 1859 (*Entodinium*,* Ophryoscolex*,* Polyplastron)* ••••••••••Polydiniellidae Corliss 1960 (*Polydiniella)* ••••••••••Rhinozetidae Van Hoven, Gilchrist and Hamilton‐Attwell 1988 (*Rhinozeta)* ••••••••••Spirodiniidae Strelkow 1939 (*Spirodinium)* ••••••••••Telamodiniidae Latteur & Dufey 1967 (*Telamodinium)* ••••••••••Troglodytellidae Corliss 1979 (*Troglodytella)* ••••••••Macropodiniida Lynn 2008 (R) Oral cavity as an anterior vestibulum lined by extensions of somatic kineties, supported by nematodesmata arising from these kinetids; somatic mucocysts; stomatogenesis telokinetal or cryptotelokinetal, possibly apokinetal; in terrestrial habitats as endocommensals in the forestomach of macropodid and vombatid marsupials. ••••••••••Amylovoracidae Cameron & O'Donoghue 2002 (*Amylovorax)* ••••••••••Macropodiniidae Dehority 1996 (*Macropodinium)* ••••••••••Polycostidae Cameron & O'Donoghue 2003 (*Polycosta)* ••••CONTHREEP Lynn in Adl et al. 2012 [Ventrata Cavalier‐Smith 2004] (R) Group identified by SSU rRNA phylogenies. With a node‐based definition: the clade stemming from the most recent common ancestor of the Colpodea (C), Oligohymenophorea (O), Nassophorea (N), Phyllopharyngea (P), Prostomatea (P), and Plagiopylea (P), hence CON‐threeP. “Ventrata” suggests a ventral morphological synapomorphy for the group, but this character does not exist. *Incertae sedis* CON‐threeP: *Askenasia* Blochmann 1895 Groups near Plagiopylea in molecular phylogenies. *Incertae sedis* CON‐threeP: Cyclotrichiidae Jankowski 1980 Groups near Plagiopylea in molecular phylogenies. *Cyclotrichium*. *Incertae sedis* CON‐threeP: *Paraspathidium* Noland 1937 Groups within Plagiopylea in molecular phylogenies. *Incertae sedis* CON‐threeP: Pseudotrachelocercidae Song 1990 Groups near Plagiopylea in molecular phylogenies. *Pseudotrachelocerca*. *Incertae sedis* CON‐threeP: Discotrichidae Jankowski 1967 [Discotrichida Fan et al. 2014] Conspicuous cortical papillae on both dorsal and ventral faces; mucocysts rod‐shaped; cytopharyngeal basket asymmetric. *Discotricha*,* Lopezoterenia*. •••••Phyllopharyngea de Puytorac et al. 1974 The ciliated stage with somatic kineties mostly as monokinetids that each have a lateral kinetodesmal fibril, a reduced or absent transverse microtubular ribbon that is usually accompanied by a left‐directed transverse fibre, and a somewhat convergent postciliary ribbon extending posteriorly to accompany ribbons of more anterior monokinetids; oral region with radially arranged microtubular ribbons, called phyllae. ••••••Synhymeniida de Puytorac et al. in Deroux 1978 Ribbon‐like subkinetal nematodesmata arising from somatic monokinetids and extending beneath kineties as nematodesmata; cyrtos conspicuous. •••••••Nassulopsidae Deroux in Corliss 1979 (*Nassulopsis)* •••••••Orthodonellidae Jankowski 1968 (*Orthodonella*,* Zosterodasys)* •••••••Scaphidiodontidae Deroux in Corliss 1979 (*Chilodontopsis*,* Scaphidiodon)* •••••••Synhymeniidae Jankowski in Small & Lynn 1985 (*Synhymenia)* ••••••Subkinetalia Gong et al. 2009 Ribbon‐like subkinetal nematodesmata arising from somatic monokinetids and extending, either anteriorly in Cyrtophoria/Chonotrichia or posteriorly, beneath kineties as subkinetal ribbons in Rhynchodia/Suctoria. •••••••Cyrtophoria Fauré‐Fremiet in Corliss 1956 Oral ciliature typically composed of one preoral kinety and two circumoral kineties; true cytostome and cytopharynx surrounded by phyllae and rod‐shaped nematodesmata; macronucleus heteromerous. ••••••••Chlamydodontida Deroux 1976 (P) Body typically dorsoventrally flattened, broad; free‐swimming, but may attach to substrate by thigmotactic ventral somatic cilia somatic kineties typically ventrally arranged in two roughly equal fields, which may be separated midventrally (except in Family Kryoprorodontidae); without non‐ciliated adhesive region or flexible podite. •••••••••Chilodonellidae Deroux 1970 (*Chilodonella)* •••••••••Chitonellidae Small & Lynn 1985 (*Chitonella)* •••••••••Chlamydodontidae Stein 1859 (*Chlamydodon)* •••••••••Gastronautidae Deroux 1994 (*Gastronauta)* •••••••••Kryoprorodontidae Alekperov and Mamajeva, 1992 (*Gymnozoum)* •••••••••Lynchellidae Jankowski 1968 (*Chlamydonella*,* Lynchella)* ••••••••Dysteriida Deroux 1976 Body typically laterally compressed with dorsal surface rounded, in extreme; free‐swimming, but often temporarily attached; ventral cilia not thigmotactic, but cell attached to substrate by non‐ciliated adhesive region or by flexible podite (except *Atelepithites*); macronucleus juxtaposed heteromerous. •••••••••Dysteriidae Claparède & Lachmann 1858 (*Dysteria* (P), *Trochilia)* •••••••••Hartmannulidae Poche 1913 (P) *Hartmannula*) •••••••••Kyaroikeidae Sniezek & Coats 1996 (*Kyaroikeus*) •••••••••Plesiotrichopidae Deroux 1976 (*Plesiotrichopus*) •••••••••Chonotrichia Wallengren 1895 Sedentary and sessile forms with somatic cilia only on walls of perioral funnel or cone‐shaped region, which may be flared or compressed; oral cilia absent or only as several inverted kineties next to cytostome; cytopharyngeal apparatus with phyllae, but no nematodesmata; macronucleus heteromerous; unequal cell division typical, producing “bud” for dispersal; most species are ectosymbionts on crustacean appendages. Small subunit rDNA phylogenies place this group as sister to Hartmannulidae. ••••••••••Exogemmida Jankowski 1972 Body typically long and cylindrical with a well‐developed collar (except Family Chilodochonidae); spines absent or poorly developed; usual attachment by undistinguished peduncle (rather than a “true” stalk, except in Family Chilodochonidae); a few to several tomites or buds produced by external budding; macronucleus heteromerous, with orthomere directed apically towards funnel. •••••••••••Chilodochonidae Wallengren 1895 (*Chilodochona*) •••••••••••Filichonidae Jankowski 1973 (*Filichona*) •••••••••••Helichonidae Jankowski 1972 (*Heliochona*) •••••••••••Lobochonidae Jankowski 1967 (*Lobochona*) •••••••••••Phyllochonidae Jankowski 1972 (*Phyllochona*) •••••••••••Spirochonidae Stein 1854 (*Spirochona*) ••••••••••Cryptogemmida Jankowski 1975 Body often flattened, leaf‐like, and angular; spines common and of several types; collar reduced; stalk typically present; internal budding with up to eight tomites produced in a crypt or marsupium; macronucleus heteromerous, with orthomere directed antapically away from funnel. •••••••••••Actinichonidae Jankowski 1973 (*Actinichona*) •••••••••••Echinichonidae Jankowski 1973 (*Echinichona*) •••••••••••Inversochonidae Jankowski 1973 (*Inversochona*) •••••••••••Isochonidae Jankowski 1973 (*Isochona*) •••••••••••Isochonopsidae Batisse & Crumeyrolle 1988 (*Isochonopsis*) •••••••••••Stylochonidae Mohr 1948 (*Stylochona*) •••••••Rhynchodia Chatton & Lwoff 1939 Oral apparatus a suctorial tube supported by radially arranged microtubular ribbons (= phyllae) enclosing toxic (?) extrusomes (acmocysts or haptotrichocysts); cytotrophic or endosymbiotic parasites of bivalve molluscs and other marine invertebrates. ••••••••Hypocomidae Bütschli 1889 Body dorsoventrally flattened; somatic kineties essentially restricted to ventral surface with a short anterio‐lateral left kinety, a presumed homologue of the dorsal right kinetofragment of cyrtophorines; posterior adhesive region bounded by somatic kineties in right‐ventral pit or fosette; oral ciliature absent or reduced to a few pericytostomial kinetosomes; macronucleus homomerous. *Hypocoma*. ••••••••Rhynchodida Chatton & Lwoff 1939 Free‐swimming, but typically attached to the host by the oral region; somatic kineties sometimes with non‐ciliated kinetosomes, typically organized in a thigmotactic field, which may extend to cover the entire body or which may be divided in two, leaving a large part of the cell surface bare; no posterior adhesive region. •••••••••Ancistrocomidae Chatton & Lwoff 1939 (*Ancistrocoma)* •••••••••Sphenophryidae Chatton & Lwoff 1921 (*Sphenophrya)* •••••••Suctoria Claparède & Lachmann 1858 Mature sessile trophonts usually non‐ciliated with one to many tentacles that ingest prey; extrusomes at tentacle tips as haptocysts; tentacles supported by an outer ring of microtubules and an inner set of microtubular ribbons (= phyllae); unequal cell division typical, with ciliated migratory dispersal “larvae” or swarmers usually bearing neither tentacles nor a stalk. ••••••••Exogenida Collin 1912 Often stalked and loricate; tentacles borne on actinophores in some species, and others with prehensile as well as suctorial tentacles; exogenous budding, most often monogemmic, but polygemmic in some species, or by binary fission with no appreciable invagination of parental cortex; migratory larval form typically large or long, the former with complex ventral ciliature derived from the parental kinetosomal field, but some of the longer larvae practically devoid of cilia vermiform, and incapable of swimming; some endocommensals. •••••••••Allantosomatidae Jankowski 1967 (*Allantosoma*) •••••••••Dentacinetidae Batisse 1992 (*Dentacineta*) •••••••••Dendrosomididae Jankowski, 1978 (*Dendrosomides*) •••••••••Ephelotidae Kent 1882 (*Ephelota*) •••••••••Lecanophryidae Jankowski, 1973 (*Lecanophrya*) •••••••••Manuelophryidae Dovgal 2002 (*Manuelophrya*) •••••••••Metacinetidae Bütschli 1889 (*Metacineta)* •••••••••Ophryodendridae Stein 1867 (*Ophryodendron*) •••••••••Paracinetidae Jankowski 1978 (*Paracineta*) •••••••••Phalacrocleptidae Kozloff 1966 (*Phalacrocleptes*) •••••••••Podophryidae Haeckel 1866 (*Podophrya*) •••••••••Praethecacinetidae Dovgal 1996 (*Praethecacineta*) •••••••••Rhabdophryidae Jankowski 1970 (*Rhabdophrya*) •••••••••Severonidae Jankowski 1981 (*Severonis*) •••••••••Spelaeophryidae Jankowski in Batisse 1975 (*Spelaeophrya*) •••••••••Tachyblastonidae Grell 1950 (*Tachyblaston*) •••••••••Thecacinetidae Matthes 1956 (*Thecacineta*) ••••••••Endogenida Collin 1912 Often loricate; tentacles frequently in fascicles; endogenous budding occurring in a pouch, monogemmic or polygemmic, with swarmers produced completely internally and becoming free‐swimming in brood pouch *before* emergence through birth pore; swarmer ciliated; ectosymbiotic forms common, some endocommensals. •••••••••Acinetidae Stein 1859 (*Acineta*) •••••••••Acinetopsidae Jankowski 1978 (*Acinetopsis*) •••••••••Choanophryidae Dovgal 2002 (*Choanophrya*) •••••••••Corynophryidae Jankowski 1981 (*Corynophrya*) •••••••••Dactylostomatidae Jankowski 1978 (*Dactylostoma*) •••••••••Dendrosomatidae Fraipont 1878 (*Dendrosoma*) •••••••••Endosphaeridae Jankowski in Corliss 1979 (*Endosphaera*) •••••••••Erastophryidae Jankowski 1978 (*Erastophrya*) •••••••••Pseudogemmidae Jankowski 1978 (*Pseudogemma*) •••••••••Rhynchetidae Jankowski 1978 (*Rhyncheta*) •••••••••Solenophryidae Jankowski 1981 (*Solenophrya*) •••••••••Tokophryidae Jankowski in Small & Lynn 1985 (*Tokophrya*) •••••••••Trichophryidae Fraipont 1878 (*Trichophrya*) ••••••••Evaginogenida Jankowski in Corliss 1979 Trophonts sessile; with or without stalk, occasionally in lorica; tentacles either scattered singly or in fascicles at the ends of sometimes massive arms or trunks; kinetosomes of larval kineties first develop on “parental” surface of a brood pouch, but cytokinesis of a single swarmer completed exogenously *after* full emergence of the “everted” bud (i.e. evaginative budding); often symphorionts, with species of one endosymbiotic genus showing a strikingly aberrant life cycle. •••••••••Cometodendridae Jankowski 1978 (*Cometodendron*) •••••••••Cyathodiniidae da Cuhna 1914 (*Cyathodinium*) •••••••••Dendrocometidae Haeckel 1866 (*Dendrocometes*) •••••••••Discophryidae Collin 1912 (*Discophrya*) •••••••••Enchelyomorphidae Augustin & Foissner 1992 (*Enchelyomorpha*) •••••••••Heliophryidae Corliss 1979 (*Heliophrya*) •••••••••Periacinetidae Jankowski 1978 (*Periacineta*) •••••••••Prodiscophryidae Jankowski 1978 (*Prodiscophrya*) •••••••••Rhynchophryidae Jankowski 1978 (*Rhynchophrya*) •••••••••Stylocometidae Jankowski 1978 (*Stylocometes*) •••••••••Trypanococcidae Dovgal 2002 (*Trypanococcus*) •••••Colpodea Small & Lynn 1981 Ciliated somatic dikinetids with one transverse ribbon and at least one postciliary microtubule associated with the anterior kinetosome and one transverse ribbon, one postciliary ribbon, and one kinetodesmal fibril associated with the posterior kinetosome; posteriorly directed transverse ribbons overlap one another, forming the so‐called transversodesmata. Oral structures as a dikinetidal row in the right field and brick‐ or ribbon‐shaped polykinetids in the left field; micronucleus may be in perinuclear space of macronucleus; division in freely motile condition or in reproductive cysts; stomatogenesis pleurotelokinetal or merotelokinetal, parental ciliature maintained or reorganized; mucocysts; mostly terrestrial. ••••••Bursariomorphida Fernández‐Galiano 1978 Oral structures in oral cavity, often very deep or trough‐like; right oral field composed of one or many dikinetidal rows; left oral field composed of few to many polykinetids forming a conspicuous ribbon resembling an adoral zone of membranelles. •••••••Bryometopidae Jankowski 1980 (*Bryometopus* (P), *Thylakidium*) •••••••Bursaridiidae Foissner 1993 (*Bursaridium*,* Paracondylostoma*) •••••••Bursariidae Bory de St. Vincent 1826 (*Bursaria*) ••••••Colpodida de Puytorac et al. 1974 Oral structures in oral cavity; right oral field as single row of monokinetids, dikinetids or a complex organelle including roof kineties and/or monokinetidal ciliary fields; left oral field composed of one to several brick‐shaped polykinetids and/or a comparatively large polykinetid comprising few to many monokinetidal rows; silverline pattern colpodid or platyophryid; usually divide in reproductive cysts; stomatogenesis merotelokinetal or pleuromerotelokinetal, parental ciliature reorganized; mostly terrestrial. *Incertae sedis* Colpodida: Bardeliellidae Foissner 1984 (*Bardeliella*) Hausmanniellidae Foissner 1987 (*Avestina*,* Hausmanniella*) Ilsiellidae Bourland et al. 2011 (*Ilsiella*) Pseudochlamydonellidae Buitkamp et al. 1989 (*Hackenbergia*,* Pseudochlamydonella*) Marynidae Poche 1913 (*Maryna*) •••••••Bryophryina de Puytorac et al. 1979 Oral cavity almost flat; right oral field a single row of dikinetids or a complex organelle including roof kineties; left field as described for order; silverline pattern platyophryid or colpodid; division in reproductive cysts; terrestrial. ••••••••Bryophryidae de Puytorac et al. 1979 (*Bryophrya*) ••••••••Sandmanniellidae Foissner & Stoeck 2009 (*Sandmanniella*) •••••••Colpodina Foissner et al. 2011 Oral cavity small to very large, in the latter case densely ciliated by roof kineties having supra‐epiplasmic microtubules; right oral field composed of a dikinetidal row and a crescentic accumulation of slightly disordered monokinetids; left oral field as a crescentic polykinetid composed of many monokinetidal rows; postorally, a more or less pronounced (diagonal) groove extending obliquely onto left body side; extrusomes globular or oblong; silverline pattern colpodid; division usually in reproductive cysts; stomatogenesis pleuromerotelokinetal; terrestrial and limnetic. ••••••••Colpodidae Bory de St. Vincent 1826 (P) (*Colpoda)* ••••••••Grandoriidae Corliss 1960 (*Grandoria*) ••••••••Tillinidae Foissner et al. 2011 (*Tillina*) •••••••Grossglockneriidae Foissner 1980 [Grossglockneriina Foissner 1980] Oral apparatus on cell surface; with unique feeding tube used for puncturing cell walls of fungi and yeasts; right oral field as single monokinetidal row; left oral field composed of one to several brick‐shaped polykinetids; silverline pattern colpodid; division in reproductive cysts; stomatogenesis merotelokinetal; terrestrial. *Grossglockneria*,* Pseudoplatyophrya*. ••••••Cyrtolophosidida Foissner 1978 Oral cavity shallow; right oral field as a single dikinetidal row, forms an elliptical figure with left oral field comprising up to 10 brick‐shaped polykinetids; occasionally micronucleus in perinuclear space of macronucleus; silverline pattern colpodid or kreyellid; division in freely motile condition; stomatogenesis pleurotelokinetal, parental oral ciliature partially reorganized; limnetic and terrestrial, some marine. •••••••Cyrtolophosididae Stokes 1888 (*Cyrtolophosis)* •••••••Kreyellidae Foissner 1979 (*Kreyella*) ••••••Platyophryida de Puytorac et al. 1979 Oral structures on cell surface; right oral field as a single dikinetidal row, usually forms an elliptical figure with the left oral field composed of few to many brick‐shaped polykinetids; occasionally micronucleus in perinuclear space of macronucleus; some with postoral pseudomembrane consisting of short kineties with two dikinetids each along left slope of oral aperture; silverline pattern platyophryid, rarely colpodid or kreyellid; division in reproductive cysts or in freely motile condition; stomatogenesis pleurotelokinetal, parental oral ciliature not reorganized; with or without the ability to form aerial sorocarps; mostly terrestrial or semiterrestrial, few limnetic. •••••••Ottowphryidae Foissner et al. 2011 (*Ottowphrya*,* Platyophryides*) •••••••Platyophryidae de Puytorac et al. 1979 (P) (*Platyophrya*) •••••••Sagittariidae Grandori & Grandori 1935 (*Sagittaria*) •••••••Sorogenidae Bradbury & Olive 1980 (*Sorogena*) •••••••Woodruffiidae Gelei 1954 (*Etoschophrya*,* Rostrophrya*,* Woodruffia*) •••••Nassophorea Small & Lynn 1981 (P) Somatic cilia as monokinetids and dikinetids; monokinetid with an anterior, tangential transverse ribbon, a divergent postciliary ribbon, and an anteriorly directed kinetodesmal fibril; somatic alveoli well‐developed with paired alveolocysts sometimes present; oral nematodesmata well‐developed as cyrtos in several groups. ••••••Colpodidiidae Foissner 1995 Oral region in middle third of cell, with a paroral and three oral polykinetids that can be reduced in size to only one or two kinetosomes; cytostome‐cytopharynx supported by a delicate cyrtos (?), which extends anteriorly, then dorsally and posteriorly. *Colpodidium*. ••••••Nassulida Jankowski 1967 Alveolocysts present; somatic ciliature with distinct preoral suture; somatic basal bodies with a proximal and distal cartwheel; somatic extrusomes rod‐shaped when present; synhymenium or hypostomial frange begins in postoral region, always to the right of the stomatogenic kinety, and extending to lateral left onto dorsal surface, but sometimes reduced to three or four polykinetids restricted to a shallow oral cavity; cyrtos typically large, with complete palisade of nematodesmata. •••••••Furgasoniidae Corliss 1979 (*Furgasonia*,* Wolfkosia*) •••••••Nassulidae de Fromentel 1874 (*Nassula*,* Obertrumia*) ••••••Microthoracida Jankowski 1967 Body frequently broadly ellipsoidal with right side more rounded, occasionally crescentic, and often laterally flattened; alveolocysts present; pellicle, firm and rigid, with thickened epiplasm in some forms; typically with a few somatic kineties, separated by wide interkinetal spaces, composed of monokinetids and/or dikinetids; somatic extrusomes as fibrous trichocysts with anchor‐shaped tip (fibrocysts); usually three left oral polykinetids; right paroral field of dikineties variably developed, but its vestige always appears during stomatogenesis; cyrtos small, with complete palisade of nematodesmata. •••••••Microthoracidae Wrzesniowski 1870 (*Drepanomonas, Microthorax*) •••••••Leptopharyngidae Kahl 1926 (*Leptopharynx, Pseudomicrothorax*) •••••Prostomatea Schewiakoff 1896 (P) Oral dikinetids radial to tangential to perimeter of oral area with postciliary microtubular ribbons extending laterally from each dikinetid, overlapping one another, and, in some species, forming a circular microtubular band that supports the wall of a shallow precytostomial cavity; associated oral ciliature as two or more assemblages of dikinetids, often called a “brush”. ••••••Apsiktratidae Foissner et al. 1994 [Prostomatida Schewiakoff, 1896] Somatic ciliature “radially symmetrical”; paratenes typically conspicuous; oral region apical, surrounded by circumoral dikinetids; brosse absent; toxicysts absent. *Apsiktrata*. ••••••Prorodontida Corliss 1974 Alveoli well‐developed, including calcium carbonate concretions as skeletal plates in the Family Colepidae; somatic ciliation may be reduced in posterior half of cell, which typically bears one to many caudal cilia somatic extrusomes as mucocysts; oral extrusomes as toxicysts, may be in oral palps or extra‐oral, near kinetids of “brosse”; oral region apical to subapical, surrounded by circumoral dikinetids; brosse typically of three or more dikinetidal rows bearing clavate cilia varying from parallel to perpendicular to body axis, and developing on parental ventral surface; cytostome sometimes in shallow atrium, which is lined by oral ridges supported by two unequal rows of microtubules; most species cytotrophic or scavengers on detritus. •••••••Balanionidae Small & Lynn 1985 (*Balanion*) •••••••Cryptocaryonidae Wright & Colorni 2002 (*Cryptocaryon*) •••••••Colepidae Ehrenberg 1838 (*Coleps*,* Plagiopogon*) •••••••Holophryidae Perty 1852 (*Holophrya*) •••••••Lagynidae Sola et al. 1990 (*Lagynus*) •••••••Metacystidae Kahl 1926 (*Metacystis*,* Vasicola*) •••••••Placidae Small & Lynn 1985 (*Placus*) •••••••Plagiocampidae Kahl 1926 (*Plagiocampa*) •••••••Prorodontidae Kent 1881 (*Prorodon*) •••••••Urotrichidae Small & Lynn 1985 (*Urotricha*) •••••Plagiopylea Small & Lynn 1985 (R) Alveoli well‐developed, often filled with dense material; somatic monokinetid with divergent postciliary ribbon, well‐developed anteriorly directed kinetodesmal fibril, and a transverse ribbon arising from dense material near triplets 2 and 3, but if *Epalxella* is correctly placed here, they typically have dikinetids; somatic extrusomes as mucocysts; cytostome partially encircled by one or two files of dikinetids, but if *Epalxella* is correctly placed here, oral ciliature can include polykinetids; stomatogenesis holotelokinetal, but may be apokinetal in *Epalxella*; mitochondria may be replaced by hydrogenosomes, which in many species are associated with endosymbiotic methanogens. ••••••Plagiopylida Small & Lynn 1985 Typically with sandwich‐like arrangement of the hydrogenosome‐methanogen assemblages. •••••••Epalxellidae Corliss 1960 Morphology suggests an affiliation with the Odontostomatida, while SSU rRNA phylogenies indicate a relationship of *Epalxella* with Plagiopylea. *Saprodinium* groups with *Discomorphella* (Odontostomatida) in molecular genealogies. *Epalxella*. •••••••Plagiopylidae Schewiakoff 1896 (*Plagiopyla*) •••••••Sonderiidae Small & Lynn 1985 (*Sonderia*) •••••••Trimyemidae Kahl 1933 (*Trimyema*) •••••Oligohymenophorea de Puytorac et al. 1974 Oral apparatus with a distinct right paroral kinety and typically three left oral polykinetids, residing in a ventral oral cavity or deeper infundibulum (maybe secondarily lost (?) in Astomatia and some astomatous Hymenostomatia); somatic monokinetids with anteriorly directed overlapping kinetodesmal fibrils, divergent postciliary ribbons, and radial transverse ribbons (except in Peniculia). ••••••Apostomatia Chatton & Lwoff 1928 Cells with a polymorphic life cycle; usually as epibionts of marine Crustacea; in some forms, novel cortical structures including a “rosette” organelle and the *x*,* y* and *z* kineties. •••••••Apostomatida Chatton & Lwoff 1928 Somatic ciliature with *x*,* y* and *z* kineties that can be associated with an *a* kinety or *a*,* b* and *c* kineties; oral apparatus may have a rosette tomites formed by multiple fission, either by palintomy in a cyst or by catenulation; trophonts, sanguicolous or exuviotrophic. ••••••••Colliniidae Cépède 1910 (*Collinia*,* Metacollinia*) ••••••••Cyrtocaryidae Corliss 1979 (*Cyrtocaryum*) ••••••••Foettingeriidae Chatton 1911 (*Foettingeria*) ••••••••Pseudocolliniidae Chantangsi et al. 2013 (*Fusiforma, Pseudocollinia*) •••••••Astomatophorida Jankowski 1966 Trophont vermiform; free‐swimming, but attached to host tissue; kineties distinctly spiralled and somatic ciliature markedly thigmotactic; no cytostome (in stages of life cycles known to date), but with remnants of oral ciliature; fission of tomont‐trophont by sequential formation of tomites (catenulation) or by multiple transverse fission with tomites remaining connected. ••••••••Opalinopsidae Hartog 1906 *Opalinopsis*. •••••••Pilisuctorida Jankowski 1966 Body ovoid to elongate; free‐swimming, but attached to host in the feeding state; with ventral adhesive organelle; species of most genera permanently in so‐called “neotenic” tomite stage; somatic kineties of tomite arched, following rim of flattened ventral surface; mature trophonts non‐ciliated, immobile, characteristically attached to seta or cuticle of host; migrating tomite ciliated but lacks a cytostome; tomites produced by synchrony or strobilation; feeding on tissue fluids of marine amphipods, isopods, decapods and cirripeds. ••••••••Conidophryidae Kirby 1941 (*Conidophrys*). ••••••Astomatia Schewiakoff 1896 Without cytostome; symbionts typical in digestive tracts of annelids, especially oligochaetes; cortical cytoskeleton in the anterior region may be conspicuously developed as attachment structure(s). Small subunit rDNA phylogenies indicate that many families may not be monophyletic. •••••••Anoplophryidae Cépède 1910 (*Anoplophrya*) •••••••Buetschliellidae de Puytorac in Corliss 1979 (*Buetschliella*) •••••••Clausilocolidae de Puytorac in Corliss 1979 (*Clausilocola*) •••••••Contophryidae de Puytorac 1972 (*Contophyra*) •••••••Haptophryidae Cépède 1923 (*Haptophrya*) •••••••Hoplitophryidae Cheissin 1930 (*Hoplitophrya*) •••••••Intoshellinidae Cépède 1910 (*Intoshellina*) •••••••Maupasellidae Cépède 1910 (*Maupasella*) •••••••Radiophryidae de Puytorac 1972 (*Radiophrya*) ••••••Hymenostomatia Delage & Hérouard 1896 Stomatogenesis by proliferation of kinetosomes typically in mid‐ventral region of the cell, posterior to and somewhat apart from parental oral apparatus. •••••••Tetrahymenida Fauré‐Fremiet in Corliss 1956 Somatic kineties with anteriormost kinetid as a dikinetid; oral region, inconspicuous, except in species that undergo microstome‐to‐macrostome transformation; oral structures with right oral *b* segment of paroral (haplokinety, undulating membrane) and three left oral polykinetids (membranelles) in oral cavity; stomatogenesis monoparakinetal, typically involving the rightmost postoral somatic kinety; microphagous forms primarily bacterivorous, but some histophagous and several polymorphic forms with cytotrophic macrostome stage; complex life cycle in histophagous and parasitic species; in freshwater habitats, sometimes terrestrial, and others as facultative or obligate parasites associated mainly with invertebrate hosts. *Incertae sedis* Tetrahymenida: Trichospiridae Kahl, 1926 (*Trichospira)* ••••••••Curimostomatidae Jankowski 1968 (*Curimostoma*) ••••••••Glaucomidae Corliss 1971 (*Glaucoma*) ••••••••Spirozonidae Kahl 1926 (*Spirozona*) ••••••••Tetrahymenidae Corliss 1952 (*Tetrahymena*) ••••••••Turaniellidae Didier 1971 (*Colpidium*,* Dexiostoma*,* Turaniella*) •••••••Ophryoglenida Canella 1964 Somatic ciliature very dense, with preoral suture; oral region inconspicuous, with paroral and three oral polykinetids, its wall “supported” by the organelle of Lieberkühn in at least one stage of the life cycle; stomatogenesis teloparakinetal, with dedifferentiation and replacement of parental oral structures, accompanied by marked regression of the paroral in the differentiated oral apparatus; division free‐swimming or by palintomy in a cyst; histophagous forms generally feeding on moribund or wounded invertebrates, though several species attack healthy fishes; in freshwater habitats; polymorphic life cycle, including resting cysts. ••••••••Ichthyophthiriidae Kent 1881 (*Ichthyophthirius*) ••••••••Ophryoglenidae Kent 1881 (*Ophryoglena*) ••••••Peniculia Fauré‐Fremiet in Corliss 1956 (P) Somatic kinetids with tangential transverse ribbons and prominently overlapping kinetodesmal fibrils; cortical alveoli lie between kinetosomal rows of oral polykinetids; extrusomes as typical fibrous trichocysts. •••••••Peniculida Fauré‐Fremiet in Corliss 1956 Somatic kinetids predominantly dikinetids; somatic extrusomes as trichocysts. ••••••••Clathrostomatidae Kahl 1926 (*Clathrostoma*) ••••••••Frontoniidae Kahl 1926 (*Disematostoma*,* Frontonia* (P)) ••••••••Lembadionidae Jankowski in Corliss 1979 (*Lembadion*) ••••••••Maritujidae Jankowski in Small & Lynn 1985 (*Marituja*) ••••••••Neobursaridiidae Dragesco & Tuffrau 1967 (*Neobursaridium*) ••••••••Parameciidae Dujardin 1840 (*Paramecium*) ••••••••Paranassulidae Fauré‐Fremiet 1962 (*Paranassula*) ••••••••Stokesiidae Roque 1961 (*Stokesia*) •••••••Urocentridae Claparède & Lachmann, 1858 [Urocentrida de Puytorac, Grain & Mignot 1987] Body broadly cylindrical with larger, rounded anterior half; free‐swimming, but may be temporarily attached to the substratum by a mucous thread; somatic ciliation as a distinct equatorial girdle; caudal cilia form a conspicuous tuft that is used for temporary attachment to substrates by a mucous thread; somatic kinetids only as monokinetids with broad, tangential transverse ribbon; somatic extrusomes as mucocysts; oral structures as a paroral along the right margin of the oral opening and three oral polykinetids of three rows each along the dorsal‐left wall. *Urocentrum*. ••••••Peritrichia Stein 1859 Body divided into three major areas: (1) oral, with a prominent peristome bordered by a dikinetid file (haplokinety) and an oral polykinetid that both originate in an oral cavity (infundibulum) at the base of which is the cytostome; (2) aboral, including kinetosomes as part of the scopula, which secretes the stalk of sessile species; and (3) telotroch, permanently ciliated on mobile species. •••••••Sessilida Kahl 1933 Body inverted bell‐ or goblet‐shaped or conical‐cylindrical; zooids dimorphic, with mature zooids or trophonts, sedentary or sessile, commonly stalked or with inconspicuous adhesive disc, attached to substrate by scopula, but a few species presumed to be secondarily mobile; dispersal stage as migratory telotroch; division isotomic or anisotomic, followed in many species by development into arboroid colonies; resting cysts; free‐living or ectosymbionts, rarely endosymbionts. ••••••••Astylozoidae Kahl 1935 (*Astylozoon*,* Hastatella*) ••••••••Ellobiophryidae Chatton and Lwoff 1929 (*Ellobiophrya*) ••••••••Epistylididae Kahl 1933 (*Epistylis*) ••••••••Lagenophryidae Bütschli 1889 (*Lagenophrys*) ••••••••Operculariidae Fauré‐Fremiet in Corliss 1979 (*Opercularia*) ••••••••Rovinjellidae Matthes 1972 (*Rovinjella*) ••••••••Scyphidiidae Kahl 1933 (P) (*Scyphidia*) ••••••••Termitophryidae Lom in Corliss 1979 (*Termitophrya*) ••••••••Usconophryidae Clamp 1991 (*Usconophrys*) ••••••••Vaginicolidae de Fromentel 1874 (*Cothurnia*,* Pyxicola*,* Thuricola*,* Vaginicola*) ••••••••Vorticellidae Ehrenberg 1838 (*Carchesium*,* Epicarchesium* (P), *Ophrydium*,* Pelagovorticella*,* Pseudovorticella* (P), *Vorticella* (P)) ••••••••Zoothamniidae Sommer 1951 (*Haplocaulus*,* Zoothamnium*) •••••••Mobilida Kahl 1933 Body conical, cylindrical, or goblet‐shaped, sometimes discoidal and orally aborally flattened; zooid mobile, with permanently ciliated trochal band, typically composed of three rings of cilia; adhesive disc on aboral pole, slightly contractile; with a ring‐like, complex skeletal armature of denticles and fibres surrounding a vestigial scopula; oral region not contractile; oral structures with infundibular portions of oral polykinetids 1 and 2 always running together in a “ribbon” and oral polykinetid 3 short, perpendicular to the other two oral polykinetids; bacterivorous, obtaining prey from water or from detritus adhering to the host, and microphagous on cellular debris from host; ectosymbionts, often on the integument or gills of invertebrates, rarely endosymbionts. ••••••••Polycyclidae Poljansky 1951 (*Polycycla*) ••••••••Trichodinidae Claus 1874 (*Trichodina*) ••••••••Trichodinopsidae Kent 1881 (*Trichodinopsis*) ••••••••Urceolariidae Dujardin 1840 (*Leiotrocha*,* Urceolaria*) ••••••Scuticociliatia Small 1967 (P) Paroral file of dikinetids with *a*,* b*, and *c* segments; stomatogenesis by proliferation of kinetosomes from the *c* segment or a “scutico”‐vestige posterior to *a* and *b* segments, with varying involvement of kinetosomes in the *a* and *b* segments; typically three oral polykinetids, often as membranoids; somatic dikinetids usually with both basal bodies ciliated; mucocysts; mitochondria cortically located, often‐fused to chondriome; stomatogenesis scuticobuccokinetal. •••••••Philasterida Small 1967 Paroral file of dikinetid shorter than other oral structures, typically by reduction of paroral *a* and *c* segments; scutica typically present. ••••••••Cohnilembidae Kahl 1933 (*Cohnilembus*) ••••••••Cryptochilidae Berger in Corliss 1979 (*Cryptochilum*.) ••••••••Entodiscidae Jankowski 1973 (*Entodiscus*) ••••••••Entorhipidiidae Madsen 1931 (*Entorhipidium*) ••••••••Orchitophryidae Cépède 1910 (P) (*Orchitophrya*) ••••••••Paralembidae Corliss & de Puytorac in Small & Lynn 1985 (*Anophrys*,* Paralembus*) ••••••••Parauronematidae Small & Lynn 1985 (*Parauronema* (P)) (P) ••••••••Philasteridae Kahl 1931 (*Kahlilembus*,* Philaster*) ••••••••Pseudocohnilembidae Evans & Thompson 1964 (*Pseudocohnilembus*) ••••••••Schizocaryidae Jankowski 1979 (*Schizocaryum*) ••••••••Thigmophryidae Chatton & Lwoff 1926 (*Thigmophrya*) ••••••••Thyrophylacidae Berger in Corliss 1961 (*Thyrophylax*) ••••••••Uronematidae Thompson 1964 (*Uronema*) ••••••••Urozonidae Grolière 1975 (*Urozona*) •••••••Pleuronematida Fauré‐Fremiet in Corliss 1956 Paroral often prominent forming a velum, with short *a* and elongate *b* segment and with *c* segment as a permanent scutica or scuticovestige; mucocysts; stomatogenesis from proter's paroral and scutica. ••••••••Calyptotrichidae Small & Lynn 1985 (*Calyptotricha*) ••••••••Conchophthiridae Kahl in Doflein and Reichenow 1929 (*Conchophthirus*) ••••••••Ctedoctematidae Small & Lynn 1985 (*Ctedoctema*) ••••••••Cyclidiidae Ehrenberg 1838 (P) (*Cristigera*,* Cyclidium*) ••••••••Dragescoidae Jankowski 1980 (*Dragescoa*) ••••••••Eurystomatellidae Miao et al. 2010 (*Eurystomatella*) ••••••••Histiobalantiidae de Puytorac & Corliss in Corliss 1979 (*Histiobalantium*) ••••••••Peniculistomatidae Fenchel 1965 (*Peniculistoma*) ••••••••Pleuronematidae Kent 1881 (P) (*Pleuronema*) ••••••••Thigmocomidae Kazubski 1958 (*Thigmocoma*) •••••••Thigmotrichida Chatton & Lwoff 1922 Thigmotactic cilia as anterior differentiations of somatic kineties, attach to host tissues; somatic kineties often spiralled around posterior cell pole, where cytostome is located; paroral not velum‐like; oral polykinetid 3 reduced or absent; stomatogenesis from proter's paroral and scutica. In molecular analyses, often nested within the Pleuronematida close to the cyclidiids. ••••••••Ancistridae Issel 1903 (*Ancistrum*) ••••••••Hemispeiridae König 1894 (*Hemispeira*) ••••••••Hysterocinetidae Diesing 1866 (*Hysterocineta*) ••••••••Paraptychostomidae Ngassam et al. 1994 (*Paraptychostomum*) •••••••Loxocephalida Jankowski 1964 (P) Non‐monophyletic group that is most closely related to Astomatia and Apostomatia; further studies are needed in order to clarify the systematics of the loxocephalids. ••••••••Cinetochilidae Perty 1852 (P) (*Cinetochilum*,* Sathrophilus*) ••••••••Loxocephalidae Jankowski 1964 (*Cardiostomatella*,* Dexiotricha*,* Loxocephalus*)
•**Rhizaria** Cavalier‐Smith 2002 With fine pseudopodia varying as simple, branching, or anastomosing patterns, often supported by microtubules in those groups examined by electron microscopy. *Incertae sedis* Rhizaria: Gymnosphaerida Poche 1913, emend. Mikrjukov 2000 Axopodial microtubules in irregular hexagonal prism; kinetocyst and other types of extrusomes along axopodia; tubular mitochondrial cristae; in some genera, cells attached to substrate with cytoplasmic stalk; free‐swimming as amoeboid or motile biciliated cells; one or more nuclei, often located in the amoeboid base of stalk when present; complex life cycle unresolved. Actinocoryne*, Cienkowskya*, Gymnosphaera*, Hedraiophrys* (possible junior synonym of Cienkowskya), Wagnerella*. Note: This group is placed here based solely on morphology, as there is no DNA sequence information. ••Cercozoa Cavalier‐Smith 1998, emend. Adl et al. 2005; emend. Cavalier‐Smith 2018 Diverse clade lacking distinctive morphological or behavioural characters; biciliated and/or amoeboid, usually with filopodia; most with tubular mitochondrial cristae; cysts common; kinetosomes connecting to nucleus with cytoskeleton; usually with microbodies and extrusomes. Incertae sedis Cercozoa: Discocelia Cavalier‐Smith 2013 [*Discocelis* Vørs 1988]. *Incertae sedis* Cercozoa: Psammonobiotidae* Golemansky 1974, emend Meisterfeld 2002 This clade is considered as most likely belonging to Euglyphida. However, this position remains to be confirmed as they do not secrete scales. Test resembling a Greek vase with terminal collar either straight or angled, test circular in cross‐section with aboral end spherical, flattened or pointed; mostly in marine interstitial sand, but also in freshwater and soils. Including *Alepiella, Chardezia, Edaphonobiotus, Feuerbornia, Frenzelina, Lesquerella, Micramphora, Micropsammella, Nadinella, Ogdeniella, Psammonobiotus*,* Propsammonobiotus* and *Rhumbleriella*. *Incertae sedis* Cercozoa: Volutellidae Sudzuki 1979 Test half‐coiled, either totally organic or with some attached particles; marine. *Pseudovolutella, Volutella*. *Incertae sedis* Cercozoa: *Kraken* Dumack, Schuster, Bass et Bonkowski 2016 Very slow moving filose amoeboid cell, roundish in shape; usually a single highly branched filopodium originating between the cell body and the substrate through a ring‐like structure sometimes visible by light microscopy; filopodium branching and anastomoses forming a network; cell division longitudinal; phagocytosis of bacteria, prey transported through the filopodium to the cell body; with one, rarely two, nuclei with a round nucleolus, one contractile vacuole, and usually one food vacuole. *Incertae sedis* Cercozoa: *Dictiomyxa, Katabia, Myxodictyium, Pontomyxa, Protomyxa, Protogenes, Pseudospora, Rhizoplasma*. •••Cercomonadida Poche 1913, emend. Vickerman 1983, emend. Mylnikov 1986, emend. Karpov et al. 2006; emend Howe et al. 2009; emend Cavalier‐Smith 2012; [=Cercomonadidae Kent 1880, emend. Mylnikov and Karpov 2004; Cercobodonidae Hollande 1942]. Phagotrophic and heterotrophic biciliate Cercozoa from soil and freshwater; usually do not swim, but glide on surfaces by means of the long posterior cilium to which the ventral surface of their typically soft bodies adheres (except *Cavernomonas*). Posterior centriole (C1) attached to side of younger anterior centriole (C2) at usually strongly obtuse angle (orthogonal only in clade A1a2) by fibrillar connections, the distal one weakly striated; both typically attached to anteriorly tapering nucleus. C2 points forwards, somewhat downwards and/or to left; C1 directed backwards, pointing downwards towards the substratum or to the left. Anterior cilium beats asymmetrically to the left; posterior typically does not undulate during smooth forward gliding. All but *Cavernomonas* have spindle‐shaped body when gliding; cytoplasm adherent to posterior cilium drawn out into a posterior tail, often pointed; high propensity for making extensive non‐locomotory feeding pseudopodia of seven types (depending on species): lamellipodia, filopodia (unbranching or branching), bulbous, finger‐like, reticulopodia or axopodia (*Filomonas radiata* only). Most have contractile vacuoles and resting cysts; some have multinucleate and multiciliate syncytial or plasmodial stages in older cultures. Ciliar transition zone very short; with proximal hub‐lattice structure; unlike glissomonads, cryothecomonads or *Katabia*, have a slender distal hub‐spoke structure instead of a conspicuous dense distal transverse plate. Anterior centriole has two microtubular roots (vp2 without an associated dense plate, unlike glissomonads and thaumatomonads, and da of two microtubules), sometimes a third (dp2; *Eocercomonas* only); posterior centriole has nucleated at its basal half ventral root vp1 and sometimes (*Paracercomonas* only) also dorsal dp1 of two or three microtubules. A root of one or two microtubules (lr) nucleates near the junction of amorphous fibrillar material projecting from both centriole bases. Ventral posterior (vp1) and ventral anterior (but posterior‐pointing) roots (vp2) typically mutually closely attached by dense material at least in their anterior region, where one crosses over the other. Microtubule nucleating centre (MNC) that nucleates a small cone of singlet microtubules associated with either or both centrioles: medial and ventral centrosome‐like fibrillar root (fr) attached directly or indirectly to posterior centriole close to centriolar junction and base of dp1 if present, often connecting to nucleus; another fibrillar root (fs) usually present on dorsal side of C2; either or both of fr and fs may nucleate a small cone of microtubules. *Cercomonas, Eoercomonas, Filomonas, Neocercomonas, Cavernomonas*. •••Paracercomonadida Cavalier‐Smith 2018 Cercomonads with a medial posterior microtubular root of two or three microtubules (dp1) attached to dorsal side of posterior centriole (unlike Cercomonadidae, whose only C1 root is vp1). Fibrillar MNC (fr) linked to and partially surrounding base of dp1, attached to nuclear‐facing side of both centrioles, but mainly C1. Sheet‐like MNC (fs) on dorsal side of C2 nucleates diverging single microtubules including two parallel conspicuous ones pointing backwards. Ventral roots vp1 and vp2 consistently to the right of plane passing longitudinally through posterior cilium (if viewed from its tip to base) and orthogonally to cell surface. Ciliar transition zone proximal hub‐lattice with broad obvious hub; peripheral lattice not partially obscured by dense diaphragm‐like material. Conspicuous dense ciliar axosome at base of central pair, sometimes with noticeable denser central hub structure; spokes radiating from it more obvious than nonagonal fibre. Centrioles in one plane, posterior centriole not offset. Left root, if present, points left; does not nucleate secondary microtubules. Pseudopodia most often finger‐like or long thick, often branched filopodia; more rarely purely lamellipodial. Gliding cells typically smaller than Cercomonadida (3‐18 μm). *Brevimastigomonas, Metabolomonas, Nucleocercomonas, Paracercomonas, Phytocercomonas*. •••Glissomonadida Howe & Cavalier‐Smith 2009 [Heteromitidae Kent 1880, emend. Mylnikov 1990, emend. Mylnikov & Karpov 2004; Bodomorphidae Hollande 1952] Heterotrophic biciliates that are covered by a plasma membrane only but are not strongly amoeboid; ancestrally biciliates that glide upon substrata on the longer posterior cilium, but includes also one derived non‐gliding genus (*Proleptomonas*); if exceptionally the anterior cilium is longer the posterior one is adherent to the cell and not used for gliding (*Proleptomonas* only); in gliding species the cell posterior is usually rounded and the cell most often oval or ovoid, not highly elongated; although some species may extend a protoplasmic tail temporarily, it is not drawn out along the posterior cilium as in most cercomonads; no cytopharynx or deep ciliar groove or pocket is evident and most species are singularly lacking in obvious morphological specializations; apart from *Proleptomonas*, which is exceptionally elongated and has a modified cytoskeleton, the nucleus is anterior and attached to the kinetid by well‐developed fibrous roots; typically at least two posterior and one anterior microtubular centriolar roots; contractile vacuole usually in cell posterior; cilia of equal thickness, simple without paraxonemal rod, hairs, or scales, sometimes acronematic; unlike in cercomonads, the ciliary transition region has a single dense transverse plate at its distal end; anterior cilium beats in ciliary fashion towards the left (as in cercomonads), and is sometimes reduced to a very short stub without an axoneme; only aerobic species currently known; mitochondria with tubular cristae; microbody attached to nuclear posterior except in *Proleptomonas*; almost exclusively inhabit soil or freshwater; Golgi dictyosomes typically associated with the nucleus, as is not the case in all Cercozoa; smooth walled cysts commonly present; sex unknown. ••••Sandonidae Howe et al. 2009 Jerky gliders; anterior‐pointing anterior cilium: *Sandona*,* Flectomonas*,* Neoheteromita, Mollimonas*. ••••Dujardinidae Howe & Cavalier‐Smith 2011 Jerky gliders; anterolateral pointing anterior cilium: *Dujardina*. ••••Bodomorphidae Hollande 1952, emend. Cavalier‐Smith in Howe et al. 2009 Smoothly gliding biciliate cells with a shorter anterior cilium that points mainly sideways or posteriorly in parallel with the posterior one; in one genus with a prominent anterior rostrum; posterior centriolar base asymmetric, sharply angled with basally extended triplets on side facing nucleus. *Bodomorpha*. ••••Proleptomonadidae Howe et al. 2009 Swimmers. *Proleptomonas*. ••••Allapsidae Howe & Cavalier‐Smith 2009 Smooth gliders; anterior cilium points sideways and somewhat backwards; centrioles symmetric. *Allapsa, Teretomonas*,* Allantion*. •••Viridiraptoridae Hess & Melkonian 2013 Biciliate naked cells, mostly occurring as rigid, variously shaped cells without rostrum or bulge. In ciliated state markedly exceeding 10 μm (contrasting most known glissomonad families). Capable of transforming into surface‐attached amoeboid state, retaining cilia and showing bridge‐like morphology with several distinct adhesion sites. Cell containing single ‘vesicular’ nucleus, close to ciliary apparatus, thus apical in ciliate state. Nucleolus spherical, roughly central, occasionally exhibiting lacuna/ae. Golgi bodies in close proximity to nuclear envelope. Cytoplasm colourless, often opaque due to diverse globules, granules and crystals. Digestive stages containing several globules of medium refractivity. Crystal‐like structures restricted to starving cells, showing various shapes: 1) Small, isodiametric or slightly elongate, glistening particles, ∼ 0.5–1 μm. 2) Slender, fusiform or needle‐like rods, often 2–3 μm in length, rarely up to ∼ 6 μm. Several mitochondria scattered throughout cell, slightly elongate to botuliform. Isodiametric extrusomes, ∼ 0.5 μm as seen with light microscope, directly beneath plasma membrane, but not in pseudopodia. Several contractile vacuoles at non‐defined positions in cell periphery, ≤ 2 μm in diameter (late diastole), non‐synchronous. Cilia naked and heterodynamic, arising very close to each other in a slightly acute or right angle. Cells gliding on posterior cilium (mostly longer than anterior cilium), cell body not attaching to gliding cilium or to substrate. Flapping motion of anterior cilium often causing motions of cell body during gliding (rotation, jiggling, vibrating). Cells capable of fluttering swimming locomotion to different extent, involving both cilia. Heterotrophic nutrition, feeding by phagocytosis on dead or live eukaryotic cells, capable of local cell wall lysis to feed exclusively on protoplast material (e.g. in certain green algae), not bacterivorous. Propagation by binary fission, plasmodia not observed. Inhabiting freshwater‐fed ecosystems. Phylogenetically defined as a well‐supported, clade including the genera *Orciraptor* and *Viridiraptor*, but excluding the genera *Agitata*,* Allantion*,* Allapsa*,* Aurigamonas*,* Bodomorpha*,* Dujardina*,* Flectomonas*,* Mollimonas*,* Neoheteromita*,* Proleptomonas*,* Sandona* and *Teretomonas*. *Viridiraptor, Orciraptor* •••Pansomonadidae Vickerman in Vickerman et al. 2005 Heterotrophic cell with two heterodynamic cilia, both cilia free from body; motile ciliate phase alternates with sedentary amoeboid phase; one amino acid insertion only between ubiquitin monomers. Clade includes last common ancestor of *Aurigamonas* and *Agitata*. *Agitata, Aurigamonas*. •••Sainouroidea^33^ Schuler et al. 2018 (R) Ancestrally amoeboid biciliates, typically without scales or theca; cells often gliding on posteror cilium; tubular cristae or flat cristae; microbody attached to nucleus. ••••Sainouridae Cavalier‐Smith 2008 Biciliate or tetraciliate phagotrophic, with one or two long, motile and highly acronematic posterior cilia possessing transition region hub‐lattice and nonagonal fibre; posterior centrioles attached basally to a dense spiral fibre, and laterally to a microtubular root; one or two anterior stubby nipple‐like cilia without these structures or roots, but with a ninefold submembrane skeleton; nucleus attached to cell surface and centrioles by striated rhizoplasts; centriole shorter (1—1.5X width) than in most Cercozoa (2—2.5 X width); fine tubular invaginations of inner membrane of nuclear envelope; peroxisome attached to the nuclear envelope; mitochondrial cristae flat or with peripheral vesicles, not elongated tubules as in most Cercozoa. *Acantholus*,* Cholamonas, Homocognata, Sainouron*. ••••Helkesimastigidae Cavalier‐Smith 2008 Biciliate semi‐rigid cells with two parallel, longitudinally offset centrioles, one bearing a trailing cilium for gliding, the other bearing a ciliary stump or rarely a short motile laterally beating cilium; non‐amoeboid phagotrophs with resting cysts; apical centrosomal plate with dorsal cape of single microtubules; dense forked fibre attaches the centriole bearing the long cilium to the centrosomal plate and nucleates the single ventral microtubule; both centrioles attached to nucleus by striated rhizoplasts. *Helkesimastix*. ••••Guttulinopsidae Olive 1970 (R) *Guttulinopsis*,* Olivorum*,* Puppisaman*,* Rosculus*. •••Thecofilosea^36^ Cavalier‐Smith 2003, emend. Cavalier‐Smith 2011 Ancestrally with robust organic extracellular theca, unlike most other Cercozoa, which are usually naked or with scales; ventral filose pseudopodia emerge from ventral groove; two cilia with diver gent kinetosomes, secondarily lost in Rhizaspidae and the euglyphid amoebae, and restricted to zoospores in Phaeodarea; ancestrally benthic gliding on posterior cilium only, but some secondarily planktonic swimmers among which Ebriacea have lost pseudopodia; theca with perforations for cilia and for pseudopodia, and three perforations in Phaeodarea (thus Tripylea Hertwig 1879), which have surrounded it by a pseudopodial net containing a pigmented phaeodium, thus converting it into a ‘central capsule’, but not homologous with that of Polycystinea of Radiolaria; silica scales absent, unlike many Imbricatea (see below), but hollow silica endoskeleton in all ebriids and most phaeodarians. Incertae sedis Thecofilosea: *Mataza*. ••••Phaeodarea Haeckel 1879 [Tripylea Hertwig 1879] Central capsule with thickened, double‐layered, capsular wall containing two kinds of pores or openings; large opening known as an “astropylum” or oral pore with a protruding mass of cytoplasm, and smaller, typically lateral openings, as “parapylae”, with thinner protruding strands of cytoplasm; dense mass of darkly pigmented granular cytoplasm, the “phaeodium,” containing undigested debris, suspended in the extracapsulum; mineral skeletons, when present, composed of scattered spicules or hollow silica bars, joined by organic material; a wide variety of forms, including geodesic frameworks, spherical to polyhedral shells, or more solid, porous clam‐shaped, bivalve shells; tubular mitochondrial cristae. •••••Phaeoconchia Haeckel 1879 Central capsule enclosed within bivalve lattice shell composed of dorsal and ventral boat‐shaped valves, which are completely separated and rarely connected by a ligament on the aboral pole. *Coelodendrum, Coelographis, Conchellium, Conchopsis*. •••••Phaeocystina Haeckel 1879 Central capsule suspended in the centre of the extra‐capsular cytoplasmic network; skeleton absent or incomplete, composed of numerous solitary, scattered pieces or spicules without organic connections. *Aulacantha, Aulographis, Cannoraphis*. •••••Phaeogromia Haeckel 1879 Central capsule located eccentrically, aborally, in simple lattice shell typically provided with large shell opening placed on the oral pole of the main axis; capsule opening surrounded by “teeth” or by peculiar elongate extensions known as “feet”, sometimes with elaborate branches. *Castanella, Challengeron, Haeckeliana, Medusetta, Tuscarora*. •••••Phaeosphaeria Haeckel 1879 Central capsule located in the centre of a simple or double spherical lattice shell, not bivalve, with a simple shell opening, lacking “feet” or “teeth”. *Aulosphaera, Cannosphaera, Sagosphaera*. ••••Cryomonadida Cavalier‐Smith 1993 (R) rDNA trees show *Rhogostoma* (Rhizaspididae) within the cryomonads, so they evolved after the hypothesized ciliated common ancestor of Ebriacea and Cryomonadida by loss of cilia and are unrelated to *Pseudodifflugia*. Includes *Rhogostoma* (previously misidentified as *Lecythium*), *Cryothecomonas, Protaspis* (renamed *Protaspa* in Howe et al. 2011 as the name *Protaspis* was preoccupied). •••••Rhogostomidae Dumack et al. 2017 Thecate amoebae with ventral slit‐like and not flexible cleft that emits filopodia; theca thin, flexible, in active cells adherent throughout to cell surface, consisting of single smooth dense layer outside and scarcely thicker than the plasma membrane; thus with bilateral symmetry; theca with exosomes (*Capsellina*) or without (*Rhogostoma*,* Sacciforma*); phagotrophic (mainly bacteria, also yeasts, algae); division longitudinal, binary; sexual reproduction unknown. Electron microscopy of *Capsellina* and *Rhogostoma* by Simitzis and Le Goff (1981). *Rhogostoma, Sacciforma, Capsellina*. •••••Protaspidae Cavalier‐Smith 1993 (R) Heterodynamic biciliated cells with cilia subapical separated by a protrusion; ciliary pit with funnel; dorsoventrally flattened and oval‐shaped with parallel lateral sides; ventral longitudinal furrow in anterior half of cell; nucleus posterior with permanently condensed chromosomes; thickened cell wall with seven layers with pores for extrusome discharge; pseudopodia emerge from slits. *Cryothecomonas, Protaspa* (P) ••••Ventricleftida Cavalier‐Smith 2011 Strongly flattened oval cell with rigid theca without scales; two unequal cilia emerging subapically, often from apical notch—posterior cilium used for gliding on surfaces; ventral cleft from which branched filose pseudopods emerge for feeding separate from and posterior to ciliary groove unlike Thaumatomonadida and *Auranticordis*; with extrusomes. *Ventrifissura, Verrucomonas*. ••••Tectofilosida Cavalier‐Smith 2003 Uninucleate cells (some may form fused multicellular aggregates) surrounded by an organic flexible tectum with one basal aperture for filopodia, sometimes including foreign mineral particles (agglutinated); cilia or silica scales absent; tubular mitochondrial cristae, cytotrophic. *Pseudodifflugia, Rhizaspis, Fisculla*. •••••Chlamydophryidae de Saedeleer 1934 (R) *Chlamydophrys*,* Lecythium, Trachyrhizium, Diaphoropodon, Clypeolina, Leptochlamydophrys*. ••••Ebriacea Lemmermann 1901 [Ebriidae Poche 1913; Ebriida Deflandre 1936] Cells with two subapically inserting cilia; open internal skeleton of silica; phagotrophic without plastids. *Ebria, Hermesinum, Botuliforma*. •••Imbricatea Cavalier‐Smith 2011 [Cavalier‐Smith 2003] Secreted surface silica scales or secondarily lost, except in basal lineages where ancestrally absent; tubular mitochondrial cristae; ciliary transition region longer than in cercomonads and sainouroids, and unlike them with dense distal plate but without the internal dense aggregates and elaborate extra structures opposite the thecal contact zone in cryomonads; groove and cilia secondarily lost by euglyphids; centrioles multiplied and reoriented to make four posteriorly directed gliding cilia in *Auranticordis*, which also lost pseudopodia; centrioles independently made parallel in the thaumatomonad/spongomonad subclade.^37^ *Incertae sedis* Imbricatea: *Discomonas* Chantangsi and Leader, 2010. ••••Spongomonadida Hibberd 1983 [Spongomonadidae Karpov 1990] Biciliated cells with asymmetrical cell projection at anterior. *Rhipidodendron, Spongomonas*. ••••Marimonadida Cavalier‐Smith & Bass 2011 Without scales and without theca; marine heterotrophic biciliate swimming cells (*Pseudopirsonia*: diatom parasites) or interstitial gliding cells with somewhat deformable, semi‐rigid pellicle underlain by muciferous bodies and four posterior cilia associated with ventral cleft (*Auranticordis*), or ciliated amoebaes with two gliding posterior cilia and a non‐ciliate feeding stage with broad lobose fan‐like pseudopods (*Rhabdamoeba*). Differ from Euglyphida by the absence of silica scales and presence of cilia, and from thaumatomonads, the only other gliding imbricates, by absence of scales. *Auranticordis, Pseudopirsonia, Rhabdamoeba, Abollifer*,* Cyranomonas* ••••Variglissida Cavalier‐Smith 2014 (R) *Clautriavia*,* Nudifila, Quadricilia*. ••••Silicofilosea Adl et al. 2005, emend. Adl et al. 2012 Secreted surface silica scales or secondarily lost; tubular mitochondrial cristae. •••••Thaumatomonadida Shirkina 1987 [Thaumatomastigidae Patterson and Zoelfell 1991] Heterotrophic, cells usually gliding that may swim also; body flattened and with two heterodynamic cilia inserting subapically and/or ventrally; some unikont; with extrusomes; filopodia produced subapically or from ventral groove; cysts; multinucleate and multiciliate stages known. ••••••Thaumatomonadidae Hollande 1952 Biciliated cells with ventral pseudopodia, long ventral posterior‐pointing cilium used for gliding on surfaces unlike Peregriniidae (see below), and a much shorter anterior cilium, which is naked (*Thaumatomonas*,* Allas*) or with small scales (*Reckertia*,* Thaumatomastix*); siliceous scales formed in vesicles attached to mitochondria cover the rigid cell surface except for a ventral zone that emits pseudopodia for feeding; unlike Peregriniidae, do not transform completely into an amoeba; all have oval or triangular two‐tiered body scales, with an upper plate bearing species‐specific perforations supported at the oval ends or triangle corners by discrete struts, unlike *Gyromitus*; upper plate lacks central cleft with inrolled sides, unlike *Peregrinia*;* Thaumatomastix* only additionally has long spine scales with near‐circular or rounded triangular bases. *Allas, Hyaloselene, Reckertia, Thaumatomonas, Thaumatomastix, Ovaloplaca, Scutellomonas, Thaumatospina, Penardeugenia*. ••••••*Esquamula* Shiratori, Yabuki & Ishida 2012 Unicellular heterotrophic, with a short anterior cilium and a long posterior cilium; cells gliding with a posterior cilium; both cilia emerge from the same ciliar pit; cells with a rigid surface and without thecae or scales; filose or lobose pseudopodia sometimes emerging; slender extrusomes consist of shaft with a horizontal‐stripe pattern and cap structure on the tip. ••••••Peregriniidae Cavalier‐Smith 2011 With only oval two‐tiered body scales and without scales on cilia or spine scales; scales either symmetric ovals with heavily out‐turned upper and lower rims (as in *Gyromitus*) or asymmetric ovals with concave to flat lower surface and convex upper surface with rims more strongly laterally inrolled than at the ends (as in *Peregrinia*); in contrast to *Thaumatomonas*, ciliary pit apical not subapical and ventral, or cells so amoeboid as to lack defined shape; cilia not clearly differentiated into anterior and posterior; unlike Thaumatomonadidae no evidence for ciliary gliding; locomotion by swimming or slow amoeboid creeping. *Gyromitus, Peregrinia*. •••••Euglyphida Copeland 1956, emend. Cavalier‐Smith 1997 Testate amoebae, with a shell built of organic material; most taxa with secreted silica scales held together by organic cement; tubular mitochondrial cristae; filamentous pseudopodia; large and conspicuous nucleus easy to see in active cells using light microscopy, surrounded by vesicles. *Incertae sedis* Euglyphida: Ampullataria*, Euglyphidion*, Heteroglypha*, Matsakision*, Pareuglypha*, Pileolus*. ••••••Euglyphina Kosakyan et al. 2016 Test covered with elliptical, discoid or denticulated siliceous plates. The least inclusive clade containing *Assulina*,* Euglypha*,* Sphenoderia* and *Trinema*. •••••••Assulinidae Lara et al. 2007 Test with a terminal aperture composed of elliptic plates disposed in a regular, alternate pattern; test strongly compressed; no specialized type of scales around the aperture; shape flattened in cross‐section; in litter and mosses. Assulina, Placocista, Valkanovia*. •••••••Sphenoderiidae Chatelain et al. 2013 Circular to elliptical silica scales that can be of different sizes and shapes, but without indentations; aperture surrounded with small round or oval scales; slightly subterminal aperture; shape flattened or circular in cross‐section in litter and mosses, and freshwater plants; *Sphenoderia*,* Trachelocorythion*, Deharvengia*. •••••••Trinematidae Hoogenraad & De Groot 1940, emend Adl et al. 2012 Test with bilateral symmetry; scales oval or round, sometimes of both types; specialized tooth‐shaped scales around the aperture, which is located on the side of the shell; aperture invaginated in some taxa; shape flattened in cross‐section; in litter and mosses; Corythion, Playfairina*, Puytoracia*, Trinema. •••••••Euglyphidae Wallich 1864, emend Lara et al. 2007 Test with a terminal aperture; thin elliptical scales; presence of specialized scales around the aperture with typical indentation; shape flattened or circular in cross‐section; in litter and mosses, freshwater sediments and plants. *Euglypha*,* Scutiglypha*. •••••••*Tracheleuglypha* Deflandre 1928 Test reinforced with relatively large, discoid plates; cross‐section circular; without specialized scales around the aperture. (There is a single sequence from this genus, which typically causes long branches in SSU rRNA gene phylogenies (Lara et al., 2007) and the node is unresolved). ••••••Cyphoderiidae de Saedeleer 1934 Scales circular, oval or kidney‐shaped, juxtaposed or imbricated; test aperture angled, some with collar; shape circular in cross‐section; in freshwater sediments and plants, supralittoral sands. Campascus*, Corythionella, Cyphoderia, Messemvriella*, Pseudocorythion, Schaudinnula*. ••••••Paulinellidae de Saedeller 1934, emend. Adl et al. 2012 Test with self‐secreted siliceous reinforcements, or proteinaceous; when present, scales are elongated, with length perpendicular to aperture. At least two genera (*Micropyxidiella* and Ovulinata) with totally organic test without silica scales; in litter and mosses, freshwater plants, marine sediments and plankton. *Micropyxidiella, Ovulinata, Paulinella*. •••Metromonadea Cavalier‐Smith 2007, emend. Cavalier‐Smith 2011 Nonpseudopodial marine gliding biciliated cells; cytotrophic predator; nonthecate but with a dense single or double‐layered surface coat that may extend up cilium; extrusomes highly elongated. *Metromonas, Metopion, Micrometopion, Kiitoksia*. •••Granofilosea Cavalier‐Smith & Bass 2009 With very fine branching or unbranched granuloreticulopodia bearing obvious extrusomes as the granules at frequent rather regular intervals, or with radiating, sometimes branched, axopodia with similar granules; pseudopodia supported by internal microtubules and typically appressed to the sub stratum during feeding, in a semi‐immobile state; in most species, pseudopodia do not anastomose; some with biciliated swimming or gliding stage. *Incertae sedis* Granofilosea*: *Apogromia*,* Kibisidytes*,* Leucodictyon, Limnofila, Mesofila, Microcometes, Microgromia, Nanofila, Reticulamoeba* and probably *Belaria, Ditrema, Heliomorpha (=Dimorpha*) and *Paralieberkuehnia*. ••••Massisteridae Cavalier‐Smith 1993 (R) *Massisteria, Minimassisteria*. ••••Clathrulinidae Claus 1874 [Desmothoracida Hertwig and Lesser 1874] Extracellular capsule or lorica attached to substrate, or cell free‐floating; sometimes mucous sheath aroud cell; pseudopodia branching capable of anastomosis, and with microtubules, but not organized in any recognizable pattern; kinetocyst extrusomes on pseudopodia; tubular mitochondrial cristae; biciliated and amoeboid stages; can be colonial; cysts. Actinosphaeridium, Cienkowskya*, *Clathrulina*,* Hedriocystis, Penardiophrys*. *Incertae sedis* Clathrulinidae*: Servetia*. •••Chlorarachnea Hibberd & Norris 1984 Amoeboid with plastids of secondary origin; plastid containing chlorophylls *a* and *b*, associated with a nucleomorph and surrounded by four membranes in total; usually reticulate pseudopodia with extrusomes; cell bodies often anastomosing; with a biciliated dispersal stage. Also includes minute marine picoplanktonic bacterivorous cell with single long acronematic smooth cilium (*Minorisa* Del Campo, 2013). *Amorphochlora, Bigelowiella, Chlorarachnion, Cryptochlora; Gymnochlora, Lotharella, Minorisa, Partenskyella, Viridiuvalis*. ••Endomyxa Cavalier‐Smith 2002, emend. Bass & Berney in Adl et al. 2019 (R) This clade has varied somewhat in definition and composition but retains a core group of Rhizaria robustly distinct from Cercozoa and Retaria. Retaria as defined here is either a sister clade to Endomyxa, or branches within it. Numerous environmental lineages exist without morphological data. It is defined here as the least inclusive clade containing the last common ancestor of Vampyrellida, Phytomyxea, *Filoreta*,* Gromia*, Ascetosporea, and all its descendants. •••Vampyrellida West 1901, emend. Hess et al. 2012 Exclusively heterotrophic, naked, phagotrophic amoeboid organisms; life cycle includes amoeboid, free‐moving trophozoites alternating with an obligatory digestive cyst, in which cell division usually take place; several taxa can fuse to form plasmodia and reach considerable sizes; sexual processes unknown; cytoplasm often differentiated into a finely granular, sometimes highly vacuolated part and structure‐less hyaloplasm, the latter often surrounding the main cell body, but at least constituting the pseudopodia; free‐living in freshwater, soil, or marine environments. *Arachnula, Gobiella, Hyalodiscus, Lateromyxa, Leptophrys, Platyreta, Thalassomyxa, Theratromyxa, Vampyrella, Vernalophrys, Penardia*. •••Phytomyxea Engler & Prantl 1897 Amoeboid or plasmodial feeding cells producing biciliate or tetraciliate cells; some with specialized solid extrusome—“satchel”—for penetrating host cells; with distinctive cruciform mitotic profile due to elongate persistent nucleolus lying orthogonal to metaphase plate; parasites or parasitoids of plants or stramenopiles. ••••Plasmodiophorida Cook 1928 *Plasmodiophora*,* Polymyxa*,* Woronina, Ligniera*,* Sorosphaerula, Spongospora*. ••••Phagomyxida Cavalier‐Smith 1993 (R) *Phagomyxa, Maullinia*. •••*Filoreta* Bass & Cavalier‐Smith 2009 Aciliate, naked, free‐living, mainly bacterivorous reticulose amoebae that form extensive multinucleate open‐mesh nets. *Filoreta*. •••*Gromia* Dujardin 1835 Test of organic material, brown and opaque, with single aperture; filopodia branched, with non‐granular cytoplasm; filopodia anastomose but not into a reticulum; multinucleate; tubular mitochondrial cristae; ciliated dispersal cells or gametes. *Gromia*. •••Ascetosporea Sprague 1979, emend. Cavalier‐Smith 2009 Complex spore structure—one or more cells, with one or more sporoplasms, without polar capsules or filaments; parasites of invertebrates. ••••Haplosporida Caullery & Mesnil 1899 Distinctive lidded spores; during spore development, spore wall produced inside of outer membrane of invaginated area; without polar capsules or polar filaments; spore anterior opening covered by hinged operculum; intranuclear spindle, a rudiment of which persists in interphase nuclei (“kernstab”); tubular mitochondrial cristae; plasmodial endoparasites of marine and sometimes freshwater animals. *Bonamia, Haplosporidium, Minchinia, Urosporidium*. ••••Mikrocytida Hartikainen et al. 2013 Microcell parasites within the genera *Mikrocytos* Farley et al., 1988 and *Paramikrocytos* Hartikainen et al. 2013 infecting aquatic invertebrates. Plasmodial (*Paramikrocytos*) and unicellular (*Mikrocytos* and *Paramikrocytos*) stages. Phylogenetically highly distinct, and defined as all lineages branching in a clade including *Mikrocytos mackini* and *Paramikrocytos canceri*. Spores unknown, in contrast to most haplosporids. *Mikrocytos, Paramikrocytos*. ••••Paramyxida Chatton 1911 Spore bicellular consisting of a parietal cell and one sporoplasm; without orifice. *Marteilia, Paramyxa, Paramarteilia, Marteilioides, Eomarteilia*. ••••*Claustrosporidium* Larsson 1987 Uninucleate sporoplasm with haplosporosomes; spore wall with no orifice and formed on sporoplasm surface, not intracellular; spores without operculum and lingula. ••••Paradiniida Schiller 1935 Unlike other ascestosporans, have a biciliated dispersal phase with two unequal cilia; marine parasites of Crustacea with multinucleate plasmodial trophic phase. *Paradinium*, “spot prawn parasite”. ••Retaria Cavalier‐Smith 2002 (R) Mainly marine heterotrophs, with reticulopodia or axopodia, and usually having various types of skeleton. •••Foraminifera d'Orbigny 1826 Filopodia with granular cytoplasm, forming branching and anastomosing network (reticulopodia); bidirectional rapid (10 mm/s) transport of intracellular granules and plasma membrane domains; tubular mitochondrial cristae; fuzzy‐coated organelle of unknown function in reticulopodia; polymorphic assemblies of tubulin as (i) conventional microtubules singly or in loosely organized bundles, (ii) single helical filaments and (iii) helical filaments packed into paracrystalline arrays; majority of forms possess a test, which can be organic walled, agglutinated or calcareous; unusual characteristic beta‐tubulin; wall structure in naked and single‐chambered forms quite variable for “naked” athalamids, such as Reticulomyxa, thicker veins vested with an amorphous, mucoid material; for thecate (soft‐walled) species, such as members of the genus *Allogromia*, proteinaceous with little or no foreign material; for agglutinated species, foreign materials bound with an amorphous or fibrous organic matrix; for multichambered (polythalamous) forms, walls containing agglutinated material or mineralized with calcite, aragonite, or silica; life cycle often comprising an alternation of asexually reproducing agamont and sexually reproducing gamont. *Incertae sedis* Foraminifera: *Lagenida* Delage and Hérouard 1896 Incertae sedis Foraminifera: *Heterogromia,* Komokiacea*. ••••Monothalamea Pawlowski et al. 2013 Single chamber (monothalamous) test with an organic or agglutinated wall; the group comprises all genera traditionally included into the Allogromiida, Astrorhizida, and the Xenophyophorea; although considered marine there are 4‐5 clades of fresh water species, the diversity of this mainly unfossilized group is poorly known and has been largely overlooked in micropaleontologically oriented foraminiferal research. *Allogromia, Astrammina, Crithionina, Notodendrodes, Psammophaga, Bathysiphon Reticulomyxa*. ••••Tubothalamea Pawlowski et al. 2012 Bi‐ or multichambered test with tubular chambers at least in the juvenile stage; wall agglutinated or calcareous; in ancestral forms the test is composed of a spherical proloculus followed by a planispirally enrolled tubular chamber in *Ammodiscus, Spirillina* and *Cornuspira*; more derived forms have multichambered tests; the highly diverse group of extinct large *Fusulinida* probably also belong to this clade. •••••Miliolida Delage & Hérouard 1896 Test bi‐ or multichambered, wall agglutinated or calcareous of high‐magnesium calcite with randomly oriented crystals refracting light in all directions and resulting in a porcelaneous appearance of the test; generally imperforate walls; chambers tubular or elongate, often planispirally coiled; some with complex internal structures adapted to host algal endosymbionts. *Alveolina, Cornuspira, Miliammina, Pyrgo, Quinqueloculina, Sorites*. •••••Spirillinida Hohenegger & Piller 1975 Test composed of proloculus followed by an enrolled tubular chamber, undivided or with few chambers per whorl; wall of low‐magnesium calcite, optically a single crystal. *Patellina, Spirillina*. •••••Ammodiscidae Reuss 1862 Test composed of globular proloculus followed by a coiled undivided tubular chamber with terminal aperture; wall agglutinated. *Ammodiscus, Glomospira*. ••••Globothalamea Pawlowski et al. 2012 Test multichambered, typically trochospirally enrolled but may be triserial, biserial or uniserial; chambers globular or crescent‐shaped in early stage; wall agglutinated or calcareous. •••••Rotaliida Delage & Hérouard 1896 Wall of low‐magnesium calcite, optically radial, bilamellar, perforate; some with internal canal system. Subdivisions proposed below. ••••••Planorbulinidae Schwager 1877 (*Planorbulinella, Hyalinea)* ••••••Discorboidea Ehrenberg 1838 •••••••Discorbidae Ehrenberg 1838 (*Discorbis)* •••••••Rosalinidae Reiss 1963) (*Rupertina, Discanomalina, “Rosalina”, Gavelinopsis, Planorbulina)* ••••••Rotalioidea Ehrenberg 1839, emend. Pawlowski 2013 •••••••Elphidiidae Galloway 1933 (*Elphidium)* •••••••Ammoniidae Saidova, 1981 (*Ammonia)* •••••••Elphidiellidae Holzmann & Pawlowski 2017 (*Elphidiella*) •••••••Haynesinidae Mikhalevich 2013 (*Haynesina, Aubignyna*) •••••••*Incertae sedis Cribroelphidium, “Elphidium”, Protelphidium* ••••••Glabratelloidea (Loeblich & Tappan 1964) •••••••Rotaliellidae Loeblich & Tappan 1964 (*Rotaliella, Rossyatella*) •••••••Buliminoididae Seiglie 1970 (*Buliminoides*) •••••••Glabratellidae Loeblich & Tappan 1964 (*Glabratella, Glabratellina, Angulodiscorbis, Planoglabratella*) ••••••Calcarinoidea (Schwager 1876) •••••••Calcarinidae Schwager 1876 (*Neorotalia, Baculogypsina, Baculogypsinoides, Schlumbergerella, Pararotalia*) ••••••Nummulitoidea de Blainville 1827 •••••••Nummulitidae de Blainville 1827 (*Nummulites, Operculinella, Cycloclypeus, Heterostegina, Operculina, Planoperculina, Planostegina*) ••••••Serioidea (Holzmann & Pawlowski 2017) •••••••Uvigerinidae Haeckel 1894 (*Uvigerina, Rectuvigerina, Trifarina*) •••••••Bolivinitidae Glaessner 1937 (*Bolivina, Brizalina, Saidovina*) •••••••Cassidulinidae d'Orbigny 1839 (*Globocassidulina, Cassidulinoides, Evolvocassidulina, Islandiella, Ehrenbergina*) •••••••Sphaeroidinidae Cushman 1927 (*Sphaeroidina*) •••••••Globobuliminidae Cushman 1927 (*Globobulimina*) *Incertae sedis* Rotaliida: •••••••Nonionidae Schultze 1854 (*Nonion, Nonionella, Nonionellina, Nonionoides*) •••••••Virgulinellidae Loeblich & Tappan 1984 (*Virgulinella*) •••••••Buliminidae Jones 1875 (*Bulimina*) •••••••Epistominellidae Holzmann & Pawlowski 2017 (*Epistominella*) •••••••Stainforthiidae Reiss 1963 (*Stainforthia, Gallitellia*) •••••••Cibicididae Cushman 1927 (*Cibicides, Cibicidoides, Heterolepa*) •••••••Chilostomellidae Brady 1881 (*Chilostomella*) •••••••Pullenidae Schwager 1877 (*Pullenia*) •••••••Nuttalidae Saidova 1981 (*Nuttalides*) •••••••Discorbinellidae Sigal 1952 (*Discorbinella, Hanzawaia*) •••••••Astrononionidae Cushman & Edwards 1937 (*Astrononion*) •••••••Oridorsalidae Loeblich & Tappan 1984 (*Oridorsalis*) •••••••Melonidae Holzmann & Pawlowski 2017 (*Melonis)* •••••••Cymbaloporidae Cushman 1927 (*Cymbaloporella*) •••••••Rubratelliidae Holzmann & Pawlowski 2017 (*Rubratella)* •••••••Murrayinelliidae Holzmann & Pawlowski 2017 (*Murrayinella*) •••••Globigerinida Delage & Hérouard 1896 (P) Wall of low‐magnesium calcite, bilamellar, perforate; surface may be covered with fine, elongate spines; planktonic mode of life. *Globigerina, Globigerinoides, Globorotalia, Orbulina*. •••••Robertinida Loeblich & Tappan 1984 Wall of hyaline, perforate, optical radial aragonite; chambers with internal partition. *Hoeglundina, Robertina, Robertinoides*. •••••Textulariida Delage & Hérouard 1896 (P) Wall agglutinated, with foreign particles attached to organic lining or cemented by low‐magnesium calcite. *Cyclammina, Eggerella, Reophax, Textularia, Trochammina*. •••••Carterina Brady 1884 [Carterinida Loeblich & Tappan 1981] Wall composed of rod‐like spicules of low‐magnesium calcite held in organic lining; chambers numerous, trochospirally coiled. *Carterina*. •••Radiolaria Müller 1858, sensu Adl et al. 2005 Cells with distinctive organic, nonliving, porous capsular wall surrounding the intra‐capsulum, which contains the nucleus or nuclei and cytoplasmic organelles; tubularcristae; axopodia supported by internal microtubules, extending distally through the capsular wall pores and connecting to a frothy external layer, the extracapsulum; extracapsulum containing digestive vacuoles and in some cases algal and/or cyanobacterial symbionts; skeletons, when present, of amorphous silica (opal) or strontiumsulphate (in Acantharia) and varying in shape from simple scattered spicules to highly ornate geometric‐shaped shells, within and/or surrounding the central capsule; the siliceous skeleton is secreted within a specialized cytoplasmic envelope (cytokalymma) that dynamically determines the shape of the skeletal matter. ••••Acantharea Haeckel 1881, emend. Mikrjukov 2000 Cell surrounded by fibrillar capsule outside of cell membrane; axopodia, spicules and amoeboid anastomosing dynamic network of irregular pseudopodia extending from the capsule; this outer network (ectoplasm) surrounded by fibrillar periplasmic cortex; inner cell region inside capsule (endoplasm) holding the organelles; axopodia, supported by microtubular arrays, with kinetocyst extrusomes and with a centroplast‐type centrosome at base of each spicule; 20 radial spicules of strontium sulphate merged at cell centre; spicule tips attached to contractile myonemes at periplasm; tubular mitochondrial cristae; often with algal symbionts in endoplasm, and captured prey in ectoplasm network; asexual reproduction unknown; sexual reproduction involving consecutive mitotic and meiotic divisions that ultimately release biciliated isogametic cells; only marine isolates known. •••••Chaunocanthida Schewiakoff 1926 Pigmented endoplasm, clears towards periphery; many small nuclei in endoplasm; clear ectoplasm with periplasmic cortex; sexual reproduction in gamontocyst; small plaques as lithosomes synthesized in Golgi and forming the gamontocyst wall; litholophus stage prior to reproduction; hexagonal microtubular arrays in axopodia; contractile matrix at base of spicules. *Amphiacon, Conacon, Gigartacon, Heteracon, Stauracon*. •••••Holocanthida Schewiakoff 1926 Pigmented endoplasm, clears towards periphery; many small nuclei in endoplasm; sexual reproduction in gamontocyst; with lithosomes forming the gamontocyst wall; dodecagonal microtubular arrays in axopodia. *Acanthochiasma, Acanthocolla, Acanthoplegma*. •••••Symphyacanthida Schewiakoff 1926 Pigmented endoplasm, clears towards periphery; ectoplasm clear; single large central nucleus; outer endoplasm with anastomosing pseudopodia; capsule and periplasmic cortex visible with light microscopy; sexual reproduction in gamontocyst with lithosomes forming the gamontocyst wall. *Amphilithium, Astrolonche, Pseudolithium*. •••••Arthracanthida Schewiakoff 1926 Thick capsule clearly demarcates pigmented endoplasm from ectoplasm; axopodia with hexagonal microtubular arrays; many nuclei in endoplasm; algal symbionts in all known species, except at reproduction; sexual reproduction without gamontocyst. *Acanthometra, Daurataspis, Dictyacantha, Diploconus, Phractopelta, Phyllostaurus, Pleuraspis, Stauracantha*. ••••Taxopodida Fol 1883 Axopodial pseudopods without kinetocysts (extrusomes), used for motility as oars; axopodial microtubules originate from depressions in nuclear envelope; microtubules in axoneme arranged in irregular hexagons; siliceous tangential spicules, with external radial spicules. *Sticholonche*, several environmental clades. ••••Polycystinea Ehrenberg 1838, emend. Haeckel 1887 Central capsule spherical to ovate with round pores in the capsular wall either distributed uniformly on the surface of a spherical capsular wall or localized at one pole of an ovate capsular wall; skeleton either absent or when present, composed of spicules or forming elaborate geometric‐shaped, porous or latticed shells. •••••Spumellaria Ehrenberg 1875, Haeckel 1887, emend. Riedel 1967 Central capsule typically spherical with uniformly distributed round pores in the capsular wall; skeleton either absent or when present, composed of spicules or forming latticed shells (spicules single, or multiple and concentrically arranged). Subdivisions not fully resolved. *Actinomma, Didymocyrtis, Euchitonia, Hexacontium, Hexalonche, Hexastylus, Octodendron, Plegmosphaera, Saturnalis, Spongaster, Spongosphaera*. •••••Nassellaria Ehrenberg 1875, emend. Haeckel 1887 Central capsule ovate with pores localized at one pole; skeleton, when present, composed of a simple tripod, a sagittal ring without tripod or porous helmet‐shaped “cephalis” enclosing the central capsule. *Artostrobus, Eucyrtidium, Lithomelissa, Pterocanium, Pterocorys*. •••••Collodaria Haeckel 1887 Skeleton either absent or when present, composed of scattered spicules within the extracapsulum; solitary or colonial forms. *Acrosphaera, Collosphaera, Collozoum, Sphaerozoum, Rhaphidozoum, Siphonsphaera, Thalassicolla*. ••Aquavolonida Bass & Berney 2018 Defined as the least inclusive clade containing the last common ancestor of the rhizarian lineages sharing a unique combination of 18S rRNA sequence signatures consisting of two complementary substitutions from U‐A to G‐C and from G‐C to C(U)‐G(A) in helix 11, one complementary substitution from A‐T to C(T)‐G(A) in helix 48, and a specific motif of two adjacent substitutions (AU instead of YG) in a nonbinding part of the 3° stem of helix 25 and all their descendants. •••*Aquavolon* Tikhonenkov, Mylnikov, & Bass 2018 With two smooth subapical heterodynamic cilia; kinetosomes approximately at right angle to each other and connected by at least one fibril; posterior kinetosome is very long (> 1 µm); shape slightly flexible, not flattened, with remarkable lateral depression in the middle lateral point of the cell body; single contractile vacuole and nucleus located anteriorly; several mitochondria with tubular cristae; rapidly swimming and rarely gliding protist; cytotrophic. ••*Tremula* Howe et al. 2011 [=Tremulida Howe et al. 2011] M Heterotrophic and phagotrophic biciliates with long anterior and posterior cilia; glide on surfaces by means of both cilia, one pointing forwards and one backwards; without light microscopically visible theca, scales or cytostome. Molecular phylogenies show it is a sister clade to Aquavolonida. *Tremula longifila*.
•**Haptista** Cavalier‐Smith 2003 Thin microtubule‐based appendages (haptonema or axopodia) used for feeding; complex mineralized (siliceous or calcareous) scales. **••**Haptophyta Hibberd 1976, ex. Edvardsen & Eikrem 2000 Autotrophic, mixotrophic or heterotrophic single cells; some in colonies, or a few filamentous; motile cells mostly possessing a haptonema, a filiform appendage situated between one pair of cilia; characteristic cell covering of unmineralized and/or mineralized scales; motile cells with two cilia generally without appendages, inserted apically or subapically; usually one or two chloroplasts with thylakoids in groups of three and with no girdle lamella; chloroplasts with immersed or bulging pyrenoid; nucleus usually posterior or central; outer membrane of nuclear envelope continuous with outer chloroplast membrane; major pigments chlorophylls *a, c1,* and *c2* with *c3* in prymnesiophyceans, fucoxanthin (e.g. 19′ hexanoyloxyfucoxanthin, 19′butanoyloxyfucoxnthin), beta‐carotene, diadinoxanthin and diatoxanthin; chrysolaminarin often the main storage product; eyespots recorded in a few genera (*Pavlova, Diacronema*); haplo‐diploid life cycles including heteromorphic alternating stages; motile, ciliated stage may alternate with nonmotile palmelloid (colonial), single‐celled or filamentous stages, or with motile, ciliated stages; sexual reproduction may be common in prymnesiophyceans; some species ichthyotoxic. **•••**Pavlovales Green 1976 Biciliated with unequal cilia inserted subapically or laterally; body scales absent; shorter cilium may have a swelling with densely staining projections on the side adjacent to the cell, the longer cilium may have thin hairs or scales; haptonema short, tapered and noncoiling; single chloroplast, sometimes with an eyespot beneath the short cilium. *Diacronema, Exanthemachrysis, Pavlova, Rebecca*. **•••**Prymnesiophyceae Hibberd 1976 Unicellular or colonial, mostly ciliated cells with mineralized and/or unmineralized scales covering the cells; some species exhibit two stages in the life cycle, with a diploid nonmotile or motile stage alternating with a ciliated haploid stage; haptonema may be long and coiling to short and noncoiling; cilia of equal or subequal lengths inserted apically or subapically. **••••**Prymnesiales Papenfuss 1955, emend. Edvardsen & Eikrem 2000 Motile or nonmotile cells, usually with two cilia and a coiling or flexible haptonema; covering of organic, sometimes spiny scales, sometimes absent; some alternate life cycle stages reported. *Chrysochromulina, Chrysocampanula, Dicrateria, Haptolina, Prymnesium, Pseudohaptolina*. **••••**Phaeocystales Medlin 2000 Motile cells with two cilia and short noncoiling haptonema; one to four chloroplasts per cell; the cell covered with scales of two sizes; life cycle may consist of nonmotile and motile stages; nonmotile cells colonial and embedded in gelatinous material. *Phaeocystis*. **••••**Isochrysidales Pascher 1910, emend. Edvardsen & Eikrem 2000 Motile or nonmotile cells; haptonema rudimentary or absent; motile cells covered with small organic scales; nonmotile cells usually covered with coccoliths. *Emiliania, Gephyrocapsa, Isochrysis, Ruttnera, Tisochrysis*. **••••**Coccolithales Schwarz 1932, emend. Edvardsen & Eikrem 2000 Cells with calcified organic scales during some stage of the life cycle; single or alternating stages in the life cycle; haptonema short or highly reduced; some species lack chloroplasts. *Balaniger, Calciosolenia, Coccolithus, Hymenomonas, Chrysotila, Wigwamma*. ••Centroplasthelida Febvre‐Chevalier & Febvre 1984^38^ [Centrohelea Kühn 1926 sensu Cavalier‐Smith in Yabuki et al. 2012; Centroheliozoa Dürrschmidt & Patterson 1987] Without cilium; naked or covered with mucous, usually with organic or mineralized elements (scales) embedded in it; axopodia supported by microtubules forming hexagon‐related pattern; ball‐and‐cone structure containing kinetocyst extrusomes along axopodia; centrosome as trilaminar disc with fibrous electron‐dense cortex, flat mitochondrial cristae. *Incertae sedis* Centroplasthelida: Spiculophryidae Shishkin & Zlatogursky 2018 (M) Centrohelids typically lacking silica scales but with numerous thin, pointed organic spicules tapering towards acute apices; expansions (but sometimes noticeably short) in panacanthocystid increase regions (PINs) 2, 6, 7, 10, 12 of SSU rRNA gene present. *Spiculophrys*. *Incertae sedis* Centroplasthelida: *Parasphaerastrum*.^39^ **•••**Pterocystida Cavalier‐Smith and von der Heyden 2007, emend. Shishkin and Zlatogursky 2018 (R) The least inclusive clade, containing *Pterocystis devonica*,* Raphidiophrys heterophryoidea* and *Choanocystis curvata* but not *Acanthocystis nichollsi*. **••••**Raphidista Shishkin & Zlatogursky 2018 Typically with unconsolidated layer of flattened siliceous plate scales, which consist of upper and lower plates, connected by internal septae or with two types of scales: plate scales of inner layer are flattened, simple in structure, usually ornamented with axial rib, outer spine scales with cylindrical shaft attached on the heart‐shaped base plate, which provides bilateral symmetry. **•••••**Choanocystidae Cavalier‐Smith & von der Heyden 2007 (M) Two contrasting types of siliceous scales, oval or bilobed tangential plate scales (margin not hollow and inrolled) forming the inner layer and outer bipartite spine scales consisting of a vertical stalk, sometimes curved or branched but lacking lateral wings, emanating from near a strong indentation on one side of a flat horizontal base. *Choanocystis*. **•••••**Raphidiophryidae Febvre‐Chevalier & Febvre 1984, emend. Shishkin & Zlatogursky 2018 (M) Typically with unconsolidated layer of flattened siliceous plate scales, which consist of upper and lower plates, connected by internal septae; scales usually surrounded by a hollow margin; organic spicules may occur. *Raphidiophrys*. **••••**Pterista Shishkin & Zlatogursky 2018 Two contrasting types of siliceous scales: tangential inner plate scales usually oval (margin not hollow and inrolled but sometimes thickened) sometimes slightly (rarely markedly) narrower at one end, sometimes slightly indented on one side (rarely slightly on both) but never distinctly bilobed; outer bipartite spine scales typically consisting of leaf‐like blade with an axial rib, usually in various degree extended apically as a projecting needle‐like point (blunt or pointed, but unbranched) and with lateral wings; spine scale base may be rounded or truncated and may be in the same plane as the blade or somewhat bent away from it (*Pterocystis*) or markedly extended as a distinct shelf or basal disc (sometimes called basal wing) orthogonal to the main blade (*Raineriophrys*); species with a pronounced basal wing may have lateral wings that are greatly reduced or absent; siliceous scales may be secondarily missing with or without replacing it with organic spicules. **•••••**Oxnerellidae Cavalier‐Smith & Chao 2012 (M) Naked, without scales, spicules or mucus coat; axopodia either radiating from the centrosome in all directions or appressed to substratum; extrusomes conspicuous. *Oxnerella*. **•••••**Pterocystidae Cavalier‐Smith & von der Heyden 2007 With two contrasting layers of siliceous scales: inner plate scales and outer bipartite leaf‐like spine scales; if siliceous scales secondarily missing (*Chlamydaster*) cell has a distinct mucous coat. *Pterocystis*,* Raineriophrys, Chlamydaster; Pseudoraphidiophrys; Pseudoraphidocystis*.^40^ **•••••**Heterophryidae Poche 1913, emend. Cavalier‐Smith & von der Heyden 2007 Lacking siliceous scales but with numerous thin pointed organic spicules tapering towards acute apices; SSU rRNA gene short, lacking expansions in panacanthocystid increase regions^41^ (PINs) 1, 2, 6, 7, 9‐12, 14 found in Marophryidae. *Heterophrys, Sphaerastrum*.^42^ **•••**Panacanthocystida Shishkin & Zlatogursky 2018 Usually with siliceous scales or with organic spicules: scale‐bearing species with both inner plate scales and outer scales of different morphology or only plate scales with a hollow inrolled margin; outer scales may be of different types based on the plate scale morphology or radially symmetrical spine scales, present in one of two forms: funnel‐shaped, or needle‐like with a radially symmetrical base. Gene of SSU rRNA usually has at least five expansions in panacanthocystid increase regions (PINs). The most inclusive clade containing *Acanthocystis nichollsi*,* Marophrys marina* and *Yogsothoth knorrus* but not a *Pterocystis devonica*. **••••**Yogsothothidae Shishkin & Zlatogursky 2018 (M) Typically with two types of siliceous scales: scales of inner layer are flattened, simple in structure, usually ornamented with axial rib, outer scales may be of different types but more similar to plate, than to spine scales. Gene of SSU rRNA usually having expansions only in panacanthocystid increase regions (PINs) 2, 6, 7, 10, 12. *Yogsothoth*. **••••**Acanthocystida Cavalier‐Smith & von der Heyden 2007 emend. Shishkin & Zlatogursky 2018 Usually with siliceous scales or with organic spicules: scale‐bearing species with both plate and spine scales or only plate scales with a hollow inrolled margin; outer spine scales radially symmetrical, present in one of two forms: funnel‐shaped, or needle‐like with a radially symmetrical base. Gene of SSU rRNA usually has expansions at least in panacanthocystid increase regions (PINs) 1, 2, 6, 7, 9–12, 14. **•••••**Marophryidae Cavalier‐Smith & von der Heyden 2007 emend. Shishkin & Zlatogursky 2018 (M) Without silica scales but covered with numerous thin, pointed organic scales tapering towards acute apices; gene of SSU rRNA usually having expansions only in panacanthocystid increase regions (PINs) 1, 2, 6, 7, 9–12, 14, not found in Heterophryidae. *Marophrys*. **•••••**Chalarothoracina Hertwig & Lesser 1874 *sensu* Cavalier‐Smith in Yabuki et al. 2012 emend. Shishkin & Zlatogursky 2018) Usually with siliceous scales or with organic spicules; scale‐bearing species with both plate and spine scales or only plate scales with a hollow inrolled margin; outer scales radially symmetrical, present in one of two forms: funnel‐shaped, or needle‐like with a radially symmetrical base; gene of SSU rRNA usually with expansions at least in panacanthocystid increase regions (PINs) 1–7, 9–12, 14. ••••••Acanthocystidae Claus 1874 emend. Shishkin & Zlatogursky 2018 (M) With two contrasting types of siliceous scales, an inner layer of typically oval plate scales (usually without an inrolled hollow edge) and an outer layer of needle‐like spine scales sometimes apically branched and/or with a peltate radially symmetrical basal disc lacking a lateral indentation (unlike *Choanocystis* spine scales). *Acanthocystis*. ••••••Raphidocystidae Zlatogursky 2018 (M) With monolayered plate scales, covering cell surface; plate scales with a hollow inflected margin; sometimes trumpet‐shaped or tubular spine scales present, forming an outer layer of coverings; at some life cycle stages the siliceous scales are completely substituted with organic spicules with some intermediate stages having a combination of the siliceous scales and organic spicules. *Raphidocystis*.
•**Cryptista** 43 Adl et al. 2019 [Cavalier‐Smith 1989, 2018] (R) This is a node‐based definition for the clade stemming from the most recent common ancestor of *Cryptomonas, Goniomonas, Kathablepharis,* and *Palpitomonas*. The name does not apply if any of the following fall within the specified clade: *Glaucocystis nostochinearum, Chlamydomonas reinhardtii, Telonema subtilis, Emiliana huxleyi*. ••*Palpitomonas* Yabuki & Ishida 2010 (M) Marine isolate, free‐living heterotrophic, heterokont biciliate, with unilateral bipartite mastigonemes on anterior cilium; cilia emerge on left side with anterior cilium vigorous and trailing posterior cilium; when swimming, in slow gyromotion; one cilium can adhere to substratum; double‐layered MLS‐like structure on one ciliary root; vacuolated cytoplasm; phagotrophic on bacteria; without ejectisomes; flat mitochondrial cristae. *Palpitomonas bilix*. ••Cryptophyceae Pascher 1913, emend. Schoenichen 1925, emend. Adl et al. 2012 [Cryptophyta Silva 1962; Cryptophyta Cavalier‐Smith 1986] Autotrophic, mixotrophic or heterotrophic with ejectisomes (trichocysts); mitochondrial cristae flat tubules; two cilia emerging subapically or dorsally from right side of an anterior depression (vestibulum); longitudinal grooves (furrows) and/or tubular channels (gullets) or a combination of both, extending posteriorly from the vestibublum on the ventral side; gullet/furrow complexes lined with large ejectisomes; with or without plastid nucleomorph complex; chloroplasts when present contain chlorophylls *a* and *c*2 and phycobiliproteins, located in thylakoid lumen; chloroplast covering comprised of inner and superficial periplast components (IPC and SPC respectively); includes heterotrophic species formerly known as *Chilomonas* and some genera diplomorphic such as *Cryptomonas* and *Proteomonas*. *Incertae sedis* Cryptophyceae: *Bjornbergiella*. **•••**Cryptomonadales Pascher 1913 Chloroplasts or leucoplasts present. *Chroomonas, Cryptomonas, Falcomonas, Geminigera, Guillardia, Hanusia, Hemiselmis, Plagioselmis, Proteomonas, Rhinomonas, Rhodomonas, Storeatula, Teleaulax*. •••Cyathomonadacea Pringsheim 1944 Chloroplasts absent. *Goniomonas* (previously *Cyathomonas*), *Hemiarma*. •••Kathablepharidacea Skuja 1939 [Kathablepharidae Vørs 1992] Free‐swimming cells with two heterodynamic cilia inserting subapically/medially; cell membrane thickened by lamellar sheath; ingest eukaryotic prey through an apical cytostome supported by bands of longitudinal microtubules; extrusomes are large coiled‐ribbons arrayed near kinetosomes; tubular mitochondrial cristae; plastids not observed. *Hatena*,* Kathablepharis, Leucocryptos, Platychilomonas, Roombia*. *Incertae sedis* **EUKARYA** **EXCAVATES** [Excavata Cavalier‐Smith 2002, emend. Simpson 2003] (P) Typically with suspension‐feeding groove of the “excavate” type, secondarily lost in many taxa; feeding groove used for capture and ingestion of small particles from feeding current generated by a posteriorly directed cilium (F1); right margin and floor of groove are supported by parts of the R2 microtubular root, usually also supported by microtubular fibres (B fibre, composite fibre), and the left margin by the R1 microtubular root and C fibre. Grouping of Metamonada and Discoba and Malawimonads is somewhat controversial, although recent multigene phylogenies have markedly increased support for monophyly of Metamonada, and of Discoba, separately. Apomorphy: Suspension‐feeding groove, homologous to that in *Jakoba libera*. Recent phylogenies indicate Metamonada and Dicoba probably do not share the same node. •**Metamonada** Grassé 1952, emend. Cavalier‐Smith 1987 Anaerobic/microaerophilic, either with modified mitochondria that lack cristae, are nonrespiratory, and lack a genome (e.g. hydrogenosomes or mitosomes), or without mitochondria; mostly ciliated cells, ancestrally with four kinetosomes per kinetid, though a great variation exists; some free‐living, many endobiotic, some parasitic. Apomorphies: mitochondrial organelles anaerobic and nonrespiratory (secondarily lost in oxymonads); four kinetosomes per kinetid (secondarily modified in a number of lineages). Incertae sedis Metamonada: *Barthelona* ••Fornicata Simpson 2003 With a single kinetid and nucleus, or a pair of kinetids and nuclei; 2‐4 kinetosomes and 1‐4 cilia per kinetid; usually with a feeding groove or cytopharyngeal tube associated with each kinetid. Nonrespiratory mitochondria without cristae. Apomorphy: “B fibre” originates against R2 microtubular root (secondarily lost in Diplomonadida and Caviomonadidae). •••“*Carpediemonas*‐like organisms” (Kolisko et al. 2010) (P) Free‐living, marine, anaerobic/microaerophilic ciliated cells with a broad ventral suspension‐feeding groove; biciliated, but with 2–4 kinetosomes; posterior cilium with 1–3 vanes and beating within the groove; with relatively large cristae‐lacking mitochondria; paraphyletic assemblage within Fornicata in molecular phylogenies. *Aduncisulcus*,* Carpediemonas*,* Dysnectes*,* Ergobibamus*,* Hicanonectes*,* Kipferlia*. •••Diplomonadida Wenyon 1926 Usually with ‘diplomonad’ cell organization, namely a pair of kinetids and two nuclei; some taxa (‘enteromonads’) have a single kinetid and nucleus, probably secondarily; each kinetid usually with four ciliated kinetosomes but sometimes only two or three ciliated; at least one cilium per kinetid directed posteriorly, associated with a cytopharyngeal tube or groove, or stretching as free axoneme axially within the cell; various nonmicrotubular fibres supporting the nucleus and cytopharyngeal apparatus; free‐living or endobiotic, often parasitic. Apomorphy: diplomonad cell organization. ••••Hexamitinae Kent 1880 With cytopharyngeal tube or groove; with an alternate genetic code—TAR codons for glutamine; several have a single kinetid and nucleus; endobiotic or secondarily free‐living. *Enteromonas*, Gyromonas*,* Hexamita*,* Spironucleus*,* Trepomonas*, Trigonomonas*, *Trimitus*. ••••Giardiinae Kulda & Nohýnková 1978 Without distinct feeding apparatus; one posteriorly directed cilium from each kinetid (F1) runs through the length of the cell axially and is intracytoplasmic; with standard genetic code; all endobiotic and with ‘diplomonad’ cell organization. Brugerolleia*, *Giardia*,* Octomitus*. •••Retortamonadida Grassé 1952 (P) Single ciliary apparatus with four kinetosomes and either two (*Retortamonas*) or four (*Chilomastix*) emergent cilia; posterior cilium has 2–3 vanes and is associated with a ventral feeding groove with posterior cytostome; cell surface often underlain by a corset of microtubules; all endobiotic, except for one free‐living species. Apomorphy: “lapel” structure as an electron‐dense sheet supporting the anterior origin of the peripheral microtubules. *Chilomastix*,* Retortamonas*. Note that molecular phylogenetic studies currently do not support monophyly, perhaps due to misidentification/polyphyly of *Retortamonas* spp. •••Caviomonadidae Cavalier‐Smith 2013 Single ciliary apparatus with two or four kinetosomes and a single cilium without vanes; ventral groove rudimentary of lost; microtubular cytoskelet simple, consisting from nuclear fibre and dorsal fan; endobiotic or free‐living. Caviomonas*, *Iotanema*. **•**•Parabasalia Honigberg 1973 Cells with a parabasal apparatus—two or more striated parabasal fibres connecting the Golgi apparatus to the ciliary apparatus; kinetid ancestrally with four cilia/kinetosomes, but frequently with additional cilia (one to thousands); one kinetosome bears sigmoid fibres that connect to a pelta–axostyle complex; reduction or loss of the ciliary apparatus in some taxa, multiplication of complete or parts of the ciliary apparatus in other taxa; closed mitosis with an external spindle, including a conspicuous microtubular bundle; mitochondria transformed to acristate hydrogenosomes; mostly endobiotic, some parasitic, some free‐living, presumably secondarily. Apomorphy: parabasal apparatus. *Incertae sedis* Parabasalia: *Tricercomitus*. •••Trichomonadida Kirby 1947 Four to six (four ancestrally) cilia with one ciliary axoneme supporting a lamelliform undulating membrane; B‐type costa, sometimes absent; comb‐like structure and infrakinetosomal body absent; axostyle usually of “*Trichomonas* type”; mostly endobiotic, some parasitic, exceptionally free‐living. *Cochlosoma*,* Dientamoeba, Lacusteria*,* Pentatrichomonas*,* Pentatrichomonoides*,* Pseudotrichomonas*,* Pseudotrypanosoma, Tetratrichomonas*,* Trichomonas*,* Trichomonoides*,* Trichomitopsis*. •••Honigbergiellida Čepička et al. 2010 (P?) Two and more than 20 (four ancestrally) cilia with one ciliary axoneme sometimes supporting a lamelliform undulating membrane; costa, comb‐like structure, and infrakinetosomal body absent; axostyle usually of “*Trichomonas* type”, sometimes of “*Tritrichomonas* type”; usually free‐living, some endobiotic. *Ditrichomonas*,* Cthulhu*,* Cthylla*,* Hexamastix*,* Honigbergiella*,* Monotrichomonas*. •••Hypotrichomonadida Čepička et al. 2010 Four cilia with one ciliary axoneme supporting a lamelliform undulating membrane; A‐type costa, sometimes absent; comb‐like structure present, but no infrakinetosomal body; biramous parabasal body; axostyle usually of “*Trichomonas* type”; endobiotic. *Hypotrichomonas, Trichomitus*. •••Tritrichomonadida Čepička et al. 2010 (P?) Uninucleate or binucleate; 0–5 (four ancestrally) cilia; ancestrally with comb‐like structure, suprakinetosomal and infrakinetosomal body; if present, undulating membrane typically of rail type, sometimes lamelliform; A‐type costa, often absent; axostyle of “*Tritrichomonas* type” or “*Trichomonas* type”; endobiotic, some parasitic. *Dientamoeba*,* Histomonas*,* Monocercomonas*,* Parahistomonas*,* Simplicimonas*,* Tritrichomonas*. •••Cristamonadida Brugerolle & Patterson 2001 Uninucleate to multinucleate; akaryomastigonts in addition to karyomastigonts in some multinucleate genera; four‐to‐thousands of cilia per mastigont; kinetosomes, except for ‘privileged kinetosomes’, often discarded during cell division in highly ciliated taxa; some with cresta and paraxonemal rod associated with the recurrent cilium; axostyle ancestrally of “*Tritrichomonas* type”, secondarily thin or reduced in some; multiple axostyles in multinuclear forms; parabasal body single or multiple, ellipsoid or rod‐shaped, often spiralled or ramified; endobiotic. *Caduceia*,* Calonympha*,* Coronympha*,* Deltotrichonympha*,* Devescovina*,* Foaina*,* Gigantomonas*,* Joenia*,* Joenina*,* Joenoides*, Joenopsis*, *Kofoidia*,* Koruga*,* Macrotrichomonas*,* Macrotrichomonoides*,* Metadevescovina*,* Mixotricha*, Pachyjoenia*, Projoenia*, *Pseudodevescovina*, Rhizonympha*, *Snyderella*,* Stephanonympha*. •••Spirotrichonymphida Grassé 1952 Multiple kinetosomes in counterclockwise spiral rows; cilia retained during cell division with the ciliary rows dividing between daughter cells; axostyle single of “*Tritrichomonas* type”, or multiple in thin bands, or reduced; endobiotic. Holomastigotes*, Holomastigotoides, Microjoenia*, Micromastigotes*, Rostronympha*, Spiromastigotes*, Spironympha*, Spirotrichonympha*, Spirotrichonymphella, Uteronympha*. •••Lophomonadida Light 1927 Multiple kinetosomes in a single kinetid arranged in an ear‐shaped row partially encircling the nucleus; single thin axostyle; endobiotic. *Lophomonas*. •••Trichonymphida Poche 1913 Bilaterally or tetraradially symmetrical, with anterior rostrum divided into two hemirostra; each hemirostrum bears one or two ciliary areas with hundreds to thousands of cilia; cilia usually retained during cell division; one hemirostrum goes to each daughter cell; numerous parabasal fibres originate from two or four parabasal plates that form a rostral tube in some; numerous thin axostyles do not protrude outside the cell; endobiotic. *Barbulanympha*,* Eucomonympha, Heliconympha, Hoplonympha, Leptospironympha*, Macrospironympha*, Pseudotrichonympha, Rhynchonympha*, Spirotrichosoma*, Staurojoenina, Teranympha, Trichonympha, Urinympha. ••Preaxostyla Simpson 2003 Heterotrophic cell with four cilia and kinetosomes per kinetid; nonrespiratory mitochondria without cristae or absent. Apomorphy: “I fibre” associated with R2 root has ‘preaxostylar’ substructure—latticework paracrystalline layer of ‘double‐cross’ thickness with a single, fine outer layer. •••Oxymonadida Grassé 1952 Single kinetid (occasionally multiple kinetids) consisting of two pairs of ciliated kinetosomes distantly separated by a preaxostyle (microtubular root R2 and paracrystalline I fibre), from which arises a microtubular axostyle; axostyle consists of parallel rows of microtubules and is contractile in some taxa; microtubular pelta present in some genera; mitochondrion either absent or non‐recognized, gut endosymbionts, mostly in lower termites and *Cryptocercus*, many taxa attach to gut wall using an anterior holdfast; closed mitosis with internal spindle. Apomorphy: Absence of ventral groove. Kinetosomes grouped in two pairs. Axostyle formed by parallel rows of microtubules*,* (not homologous to that of Parabasalia). Absence of recognizable mitochondrion. *Barroella, Blattamonas*,* Brachymonas*,* Dinenympha, Microrhopalodina*,* Monocercomonoides, Notila*,* Opisthomitus*,* Oxymonas, Paranotila*,* Polymastix, Pyrsonympha, Sauromonas*,* Saccinobaculus, Streblomastix, Tubulimonoides*. **•**•**•**Trimastigidae Saville Kent 1880‐1882 Free‐living excavate cells bearing four cilia stretched roughly in the anterior, right, left, and posterior directions; a broad ventral feeding groove, in which beats the posteriorly directed cilium; posterior cilium with two broad vanes without thickened vane margins, no conspicuous lateral cytopharynx; nonrespiratory mitochondria without cristae. Marine and freshwater. *Trimastix*. **•**•**•**Paratrimastigidae Zhang et al. 2015 Similar to Trimastigidae but with thickened vane margins on posterior cilium; lateral cytopharynx may be present; freshwater species only. *Paratrimastix*.
•**Discoba** Simpson in Hampl et al. 2009 (R) A grouping robustly recovered in multigene phylogenetic analyses, containing Heterolobosea, Euglenozoa, Jakobida, and Tsukubamonadida; ancestrally biciliate. Node‐based definition: the clade stemming from the most recent common ancestor of *Jakoba libera*,* Andalucia godoyi*,* Euglena gracilis*,* Naegleria gruberi* and *Tsukubamonas globosa*. **•**•Jakobida Cavalier‐Smith 1993 With two cilia at the head of a broad ventral feeding groove, in which beats the posterior cilium; posterior cilium with a single dorsal vane that is distinctive among excavates but possibly plesiomorphic; free‐living. •••Andalucina Cavalier‐Smith 2013 Free‐swimming cells, attaching temporarily to surfaces; aerobic with tubular mitochondrial cristae or anaerobic with acristate mitochondria. With a G:C base pair within the base of the stem of “helix 27” of the 18S rRNA molecule (positions 1050 and 1085 in the *Andalucia incarcerata* 18S rRNA gene sequence AY117419). ••••Andaluciidae Cavalier‐Smith 2013 Aerobic, mitochondria cristate. *Andalucia*. ••••Stygiellidae Pánek et al. 2016 Anaerobic, mitochondria acristate. *Stygiella, Velundella*. •••Histionina Cavalier‐Smith 2013 Free‐swimming or sessile cells, some genera with lorica; aerobes with flat mitochondrial cristae. Without the Andalucina‐specific G:C base pair within the base of the stem of “helix 27” of the 18S rRNA molecule. *Histiona, Jakoba*,* Moramonas*,* Reclinomonas*,* Seculamonas* nomen nudum. ••Tsukubamonadida Yabuki et al. 2011 (M) Rounded biciliate cell, with four kinetosomes per kinetid; aerobic; consumes prey through ventral groove; dyctiosomes and ciliary vanes absent. *Tsukubamonas*. **•**•Heterolobosea Page & Blanton 1985 Typically with ciliated and amoeboid phases, though many species lack the ciliated phase, while some others lack the amoeboid phase; rarely amoeboid when ciliated; amoebae often with eruptive pseudopodia; ciliated cells usually with two or four cilia (rarely uni‐ or multiciliate), sometimes nonfeeding; if capable of feeding usually use a groove‐like cytostome; closed mitosis with internal spindle; mitochondrial cristae flattened, often discoidal, mitochondria sometimes acristate; discrete dictyosomes not observed; ciliary vanes usually absent. Apomorphy: complex life cycle containing amoeba, ciliate and cyst. •••Pharyngomonada Cavalier‐Smith 2008 Amoebae usually flabellate or ovoid, eruptive movement rare; with four cilia in side‐by‐side obtuse pairs; feeding using a large groove and cytopharynx; with amoeboid phase; lack helix 17‐1 region in SSU rRNA that is typical of Tetramitia. *Pharyngomonas*. •••Tetramitia Cavalier‐Smith 1993 Amoebae usually with cylindrical, eruptive pseudopodia; swimming form usually with four cilia or two per kinetid. Apomorphy: distinct helix 17‐1 in the SSU rRNA molecule. ••••Selenaionidae Hanousková et al. 2018 Amoeboid or ciliated; when present with two cilia per kinetid, with orthogonally arranged kinetosomes; finger‐like projection on the proximal part of the recurrent cilium; nucleus with parietal nucleoli. *Dactylomonas*,* Selenaion*. ••••Neovahlkampfiidae Hanousková et al. 2018 Amoebae; nucleus with central nucleolus. *Neovahlkampfia*. ••••Eutetramitia Hanousková et al. 2018 Amoebae or ciliated; when present usually with four cilia or two per kinetid, with parallel kinetosomes, although *Stephanopogon* has numerous monokinetids, *Creneis* has up to 14 cilia emerging from two to three places; nucleus with central or parietal nucleoli. •••••Vahlkampfiidae Jollos 1917 (P) Ciliated amoebae or amoeboid without cilium; quadriciliate or biciliate; nucleolus persists through mitosis; single nucleus *Fumarolamoeba*,* Heteramoeba*,* Naegleria*,* Neovahlkampfia*,* Paravahlkampfia*,* Tetramitus*,* Vahlkampfia*,* Willaertia*. •••••Gruberellidae Page & Blanton 1985 Locomotion amoeboid or swimming with cilia; nucleolus fragments during mitosis; uninucleate or multinucleate; ciliated form observed in unidentified species of *Stachyamoeba*. Gruberella*, *Stachyamoeba*. •••••Acrasidae Poche 1913 Amoebae of some species aggregate to form fruiting bodies; nucleus may or may not fragment. Apomorphy: Formation of fruiting bodies. *Acrasis*,* Allovahlkampfia*,* Pocheina*. •••••Percolomonadidae Cavalier‐Smith 2008 Swimming cells with four cilia; without amoeboid from; aerobic. *Percolomonas*. •••••Psalteriomonadidae Cavalier‐Smith 1993 Cell amoeboid with or without cilia, or swimming with one cilium; when amoeboid and ciliated, with 4 groups of 4 cilia; anaerobic. *Harpagon*,* Psalteriomonas*,* Monopylocystis*,* Pseudoharpagon*,* Sawyeria*. •••••Stephanopogonidae Corliss 1961 Always with multiple cilia; aerobic. *Stephanopogon*. •••••Creneidae Pánek et al. 2014 (M) Amoeboid with a single cilium or multiciliate; anaerobic with acristate mitochondria. *Creneis*. •••••Tulamoebidae Kirby et al. 2015 Biciliate, locomotion amoeboid or swimming with cilia; with elongate ingestion apparatus opening slightly posterior to the ciliary insertion; halophilic or very halotolerant. *Pleurostomum*,* Tulamoeba*. ••Euglenozoa Cavalier‐Smith 1981, emend. Simpson 1997 Cells with two cilia, occasionally one, rarely more, inserted into an apical/subapical ciliary pocket; with rare exceptions, emergent cilia with heteromorphic paraxonemal rods; usually with tubular feeding apparatus associated with ciliary apparatus; basic ciliary apparatus pattern consisting of two functional kinetosomes and three asymmetrically arranged microtubular roots; single mitochondrion mostly with discoidal cristae. Apomorphy: heteromorphic paraxonemal rods, tubular/whorled in anterior cilium F2 and a parallel lattice in posterior cilium F1. •••Euglenida Butschli 1884, emend. Simpson 1997 With a pellicle of proteinaceous strips, fused in some taxa; when unfused and with > ˜20 strips capable of active distortion (metaboly); where known, paramylon is the carbohydrate store. Apomorphy: Pellicle of protein strips. ••••Heteronematina Leedale 1967 (P)^44^ With ingestion apparatus capable of phagotrophy; lacking plastids; most glide on surfaces on one or both cilia; a paraphyletic assemblage from which Euglenophyceae and Aphagea are descended. *Anisonema, Atraktomonas, Biundula*,* Calycimonas, Decastava*,* Dolium, Dinema, Dylakosoma, Entosiphon, Heteronema, Jenningsia*,* Keelungia, Lentomonas, Neometanema, Notosolenus, Pentamonas, Peranema, Peranemopsis, Petalomonas, Ploeotia*,* Scytomonas*,* Serpenomonas*,* Sphenomonas*,* Teloprocta*,* Tropidoscyphus*,* Urceolus*. ••••Aphagea Cavalier‐Smith 1993, emend. Busse & Preisfeld 2002 Osmotrophic euglenids lacking photosensory apparatus and plastids; one or two emergent cilia; no ingestion apparatus. *Astasia s.s., Distigma*,* Gyropaigne*,* Menoidium*,* Parmidium*,* Rhabdomonas*. ••••Euglenophyceae Schoenichen 1925, emend. Marin & Melkonian 2003 [Euglenea Butschli 1884, emend. Busse and Preisfeld 2002] Phototrophic, with one to several plastids of secondary origin with three bounding membranes and chlorophylls *a* and *b*; some species secondarily non‐photosynthetic; most with extraplastidic eyespot and photosensory apparatus associated with cilia; most motile. •••••Rapaza Yamaguchi et al. 2012 Cells solitary, cytotrophic on microalgae with two heterodynamic cilia of unequal length; pellicle with helically arranged strips capable of metaboly locomotion; discoidal chloroplast(s) with pyrenoids surrounded by three membranes; marine species. *Rapaza*. •••••Eutreptiales Leedale 1967, emend. Marin & Melkonian 2003 2–4 emergent heterodynamic cilia of equal or unequal length; cells not rigid, usually capable of metaboly; mostly marine or brackish species, rarely freshwater. *Eutreptia, Eutreptiella*. •••••Euglenales Leedale 1967, emend. Marin & Melkonian 2003 Single emergent cilium and second cilium within the reservoir, or both cilia non‐emergent; phototrophic or secondarily heterotrophic; mostly freshwater species. ••••••Phacaceae Kim et al. 2010 Solitary, with one emergent cilium; palmelloid stages, cysts and envelopes unknown; numerous small discoid chloroplasts without pyrenoids, large paramylon grains. *Discoplastis, Lepocinclis, Phacus*. ••••••Euglenaceae Dujardin 1841, emend. Kim et al. 2010. Cells solitary or colonial, with one emergent cilium; some with palmelloid stages, especially in *Euglena*;* Strombomonas* and *Trachelomonas* loricate; one large to several small chloroplasts of various shapes with or without pyrenoid. *Ascoglena, Colacium, Cryptoglena, Euglena, Euglenaformis, Euglenamorpha, Euglenaria, Euglenopsis, Hegneria, Klebsina, Monomorphina, Strombomonas, Trachelomonas*. •••Diplonemea Cavalier‐Smith 1993, emend. Simpson 1997 Heterotrophic cells exhibiting pronounced metaboly; both cilia are short and usually supported with paraciliary rod; apical papilla, feeding apparatus with ‘pseudovanes’; few giant, flattened mitochondrial cristae. ••••Diplonemidae Cavalier‐Smith 1993, emend. Adl et al. 2019 Perform extensive *trans*‐splicing and editing of mitochondrial RNA; trophic stage present; some species contain endosymbiotic bacteria; metaboly always present. *Diplonema*,* Rhynchopus, Lacrimia, Sulcionema, Flectonema*. ••••Hemistasiidae Cavalier‐Smith 2016, emend. Adl et al. 2019 Anterior rostrum; large posterior vacuole; contain numerous extrusomes; *Hemistasia*. ••••Eupelagonemidae Okamoto & Keeling 2018. Previously refered to as the deep‐sea pelagic diplonemids (DSPD1 clade). Possible lack of metaboly, represent ˜97% of all marine diplonemids, globally distributed. *Eupelagonema*. •••Symbiontida Yubuki et al. 2009 Microaerobic or anaerobic cells that possess rod‐shaped epibiotic bacteria; lack euglenid type pellicle strips. Currently treated as a major taxon within Euglenozoa, but are probably derived phagotrophic euglenids. Apomorphy: Rod‐shaped epibiotic bacteria above superficial layer of mitochondrion‐derived organelles with reduced or absent cristae, homologous to the organization in *Calkinisia aureus*. *Bihospites, Calkinsia, Postgaardi*. •••Kinetoplastea Honigberg 1963 Cells with a kinetoplast, which is a large mass(es) of mitochondrial (=kinetoplast; k) DNA; Apomorphy: kinetoplast; mitochondrial RNA editing; *trans*‐splicing of splice leader RNA; polycistronic transcription. *Incertae sedis* Kinetoplastea: *Bordnamonas*,* Cephalothamnium*,* Rhynchoidomonas*. ••••Prokinetoplastina Vickerman in Moreira et al. 2004 (R) Two genera: *Ichthyobodo* are polykinetoplastic and biciliated cells with the cilia originating in a pocket that continues as a furrow; ectoparasitic, freshwater, and marine. *Perkinsela*‐like organisms (PLOs), oval cells with a single large kinetoplast, non‐ciliated, live as endosymbionts (‘parasomes’) of certain amoebae (e.g. *Paramoeba* spp.), but are not enclosed in parasitophorous membrane. *Ichthyobodo*,* Perkinsela*. ••••Metakinetoplastina Vickerman in Moreira et al. 2004 (R) Group identified by SSU rRNA phylogenies. Node‐based definition: clade stemming from the most recent common ancestor of Neobodonida, Parabodonida, Eubodonida, and Trypanosomatida. •••••Neobodonida Vickerman in Moreira et al. 2004 (R) Eu‐ or polykinetoplastic kDNA not in a single network, but in multiple loci throughout the mitochondrion; biciliated, without conspicuous hairs; posterior cilium attached or free; phagotrophic or osmotrophic; preciliary rostrum containing apical cytosome. Node: *Actuariola, Azumiobodo, Cruzella, Cryptaulax*,* Dimastigella, Klosteria, Neobodo, Phanerobia, Rhynchobodo, Rhynchomonas*. •••••Parabodonida Vickerman in Moreira et al. 2004 (R) Pankinetoplastic kDNA not in a single network, but evenly distributed in the mitochondrion; biciliated, without mastigonemes; posterior cilium attached or free; phagotrophic or osmotrophic; cytostome, when present, anterolateral; free‐living or commensal/parasitic. Node: *Cryptobia, Jarrellia, Parabodo, Procryptobia, Trypanoplasma*. •••••Eubodonida Vickerman in Moreira et al. 2004 (R) Eukinetoplast with kDNA not in a single network, but in parakinetosomal position; biciliated with anterior cilium with nontubular mastigonemes; phagotrophic; anterolateral cytostome surrounded by lappets; free‐living *Bodo*. •••••Trypanosomatida Kent 1880, emend. Vickerman in Moreira et al. 2004 Eukinetoplastic with kDNA network associated with ciliary basal body; unciliated, lacking mastigonemes and emerging from anterior pocket, or emerging laterally and attached to body; phagotrophic or osmotrophic; cytostome, when present, simple and close to ciliary pocket; exclusively parasitic. Node: monoxenous (= single host) genera *Angomonas*,* Blechomonas, Leptomonas, Paratrypanosoma, Sergeia, Strigomonas, Wallaceina* and dixenous (= two hosts) genera *Phytomonas, Trypanosoma*. ••••••Leishmaniinae Maslov & Lukeš 2012 (R) Group identified by SSU rRNA and GAPDH phylogenies, with relatively slow evolution of the gene sequences. Includes monoxenous genera *Borovskyia, Crithidia, Leptomonas, Lotmaria, Novymonas, Porcisia, Zelonia,* and dixenous genera *Endotrypanum*,* Leishmania*.
•**Malawimonadidae** O'Kelly & Nerad 1999 Small free‐living biciliated cells, superficially similar to *Carpediemonas* but not closely related, with a typical respiratory mitochondrion with discoidal cristae and genome; two kinetosomes, posterior cilium with a single ventral (*Malawimonas*) or two opposing (*Gefionella*) vanes; typically from freshwater or soil. *Gefionella, Malawimonas*. •“CRuMs” (Brown et al. 2018) [Varisulca Cavalier‐Smith 2012] (R) This probable sister clade in Amorphea, informally referred to as CRuMs, includes at least Collodictyonidae, Rigifilida and *Mantamonas*. All members exhibit some form of cellular plasticity, some with pseudopodia. Incertae sedis CRuMs: *Glissandra*. ••Collodictyonidae Brugerolle et al. 2002, emend. Adl et al. 2019 [Diphylleidae Cavalier‐Smith 1993, Diphylleida Cavalier‐Smith 1993, Diphyllatea Cavalier‐Smith 2003, Sulcomonadidae Cavalier‐Smith 2013] Free‐swimming 10–15 µm long cells with two or four equal apical cilia orthogonal to each other; ciliary transition zone long, with a two‐part axosome; phagocytosis of other eukaryotic cells occurring through use of pseudopodia in a conspicuous longitudinal ventral groove that extends to posterior end, giving a double‐lobed appearance. *Collodictyon*,* Diphylleia* (= *Aulacomonas*), *Sulcomonas*. ••Rigifilida Cavalier‐Smith in Yabuki et al. 2012 Cells rounded, 5–10 µm in diameter, aproximately circular in dorsoventral aspect although somewhat plastic; pellicle underlies cell membrane on dorsal and lateral surfaces; central circular depression on venter of cell, with collar‐like margin of reflected pellicle; branching fine pseudopodia arising from ventral depression, used to capture bacteria; flat mitochondrial cristae. *Micronuclearia*,* Rigifila*. ••*Mantamonas* Cavalier‐Smith and Glücksman in Glücksman et al. 2011 (M) Gliding marine biciliated ˜5‐µm cell; body flattened, characteristically seen wider than long with left side of cell having angled shape or with short (˜1 µm) rounded projection, and right side plastic; however, cell can also be longer than wide, with rounded anterior with posterior end tapering to point at posterior cilium, or any intermediate shape; long posteriorly directed cilium directed straight behind gliding cell; anterior cilium very short and thin, projecting stiffly forward and left. *Mantamonas plastica*. •Ancyromonadida Cavalier‐Smith 1998 [=Planomonadida Cavalier‐Smith 2008] Small (˜5 µm) benthic gliding cells, dorsoventrally compressed, with leftward‐oriented rostrum at anterior; two unequal cilia, each emerging in separate shallow pocket; short apical anterior cilium may be very thin or terminate at cell membrane; long posterior cilium inserts ventrally/left‐laterally; rostrum contains extrusomes in rows; cell membrane supported by a thin single‐layered theca; discoidal/flat mitochondrial cristae; bacterivorous. This clade is the likely sister clade to the Amorphea and CRuMs. *Ancyromonas*,* Fabomonas*,* Nutomonas*,* Planomonas*. •Hemimastigophora Foissner et al. 1988 Ellipsoid to vermiform cells, cilia typically 15–40 µm long, arranged in two lateral rows that may or may not run the whole length of the cell, with up to about a dozen cilia per row; submembranous thecal plates separate the cilia; thecal plates rotationally symmetrical, supported by microtubules; anterior differentiated into a capitulum, which is the site of phagocytosis; tubular and saccular mitochondrial cristae; with bottle‐shaped extrusomes. *Incertae sedis* Hemimastigophora*: Paramastix*. ••Spironemidae Doflein 1916 *Hemimastix*,* Spironema*,* Stereonema*. •*Meteora* Hausmann et al. 2002 (M) Gliding 3–4 µm round cell with long, thin, stiff anterior and posterior protrusions; two (rarely more) short paired lateral ‘arms’ that wave anteriorly and posteriorly as if rowing, generally at same frequency but varying between moving in same or opposite directions; ‘arms’ usually straight, ˜cell diameter long, but may be branched; ‘arms’ often have linearly arranged nodules, nodules occasionally also found in anterior/posterior protrusions. *Meteora sporadica*.

**Table 3:** Genera *incertae sedis* in eukaryotes, with uncertain affiliation within protists

*Acinetactis*
*Actinastrum*
*Actinocoma*
*Actinolophus*
*Adinomonas*
*Aletium*
*Amphimonas*
*Amylophagus*
*Anaeramoeba*
*Aphelidiopsis*
*Asterocaelum*
*Asthmatos*
*Aurospora*
*Barbetia*
*Belaria*
*Belonocystis*
*Bertarellia*
*Bertramia*
*Bodopsis*
*Boekelovia*
*Branchipocola*
*Camptoptyche*
*Chalarodora*
*Cibdelia*
*Cichkovia*
*Cinetidomyxa*
*Cingula*
*Cladomonas*
*Clathrella*
*Codonoeca*
*Coelosporidium* a
*Copromonas*
*Cyanomastix*
*Cyclomonas*
*Cytamoeba*
*Dallingeria*
*Dictyomyxa*
*Dimastigamoeba*
*Dinemula*
*Dinoasteromonas*
*Diplocalium*
*Diplomita*
*Diplophysalis*
*Diploselmis*
*Dobellina*
*Ducelleria*
*Ectobiella*
*Elaeorhanis*
*Embryocola*
*Endamoeba*
*Endemosarca*
*Endobiella*
*Endomonas*
*Endospora*
*Enteromyxa*
*Eperythrocytozoon*
*Errera*
*Fromentella*
*Gymnococcus*
*Gymnophrydium*
*Haematotractidium*
*Hartmannina*
*Heliobodo*
*Heliomonas*
*Hermisenella*
*Heterogromia*
*Hillea*
*Hyalodaktylethra*
*Immanoplasma* b
*Isoselmis*
*Janickina*
*Kamera*
*Lagenidiopsis*
*Liegeosia*
*Luffisphaera* c
*Lymphocytozoon*
*Lymphosporidium*
*Macappella*
*Magosphaera*
*Malpighiella*
*Martineziella*
*Megamoebomyxa*
*Meringosphaera*
*Microcometes*
*Monochrysis*
*Monodus*
*Mononema*
*Myrmicisporidium*
*Naupliicola*
*Nephrodinium*
*Neurosporidium*
*Orbulinella*
*Ovicola*
*Palisporomonas*
*Pansporella*
*Paradinemula*
*Paraluffisphaera*
*Paramonas*
*Paraplasma*
*Parastasia*
*Parastasiella*
*Peliainia*
*Peltomonas*
*Petasaria*
*Phagodinium*
*Phanerobia*
*Phloxamoeba*
*Phyllomitus*
*Phyllomonas*
*Physcosporidium*
*Piridium*
*Pleuophrys*
*Pleuromastix*
*Protenterospora*
*Protomonas*
*Pseudoactiniscus*
*Pseudosporopsis*
*Rhizomonas*
*Rhynchodinium*
*Rigidomastix*
*Schewiakoffia*
*Sergentella*
*Serpentoplasma*
*Sphaerasuctans*
*Spongastericus*
*Spongocyclia*
*Stephanomonas*
*Strobilomonas*
*Tetradimorpha*
*Tetragonidium*
*Thaulirens*
*Topsentella*
*Toshiba*
*Trichonema*
*Urbanella*

a Probably a junior synonym of *Nephridiophaga*, a Zygomycete.

b Immanoplasma Neumann 1909 (see Kar 1990).

c *Belonocystis* (Amoebozoa incertae sedis) *and Luffisphaera* maybe the same genus.

**Table 4:** Recommended primers for environmental samples

Supergroup or highest rank	Clades	Primer pair codes	Sequence length (bp)	Forward primer (5′→3′)	Reverse primer (5′→3′)
Amorphea	Apusomonadida^1^	18S, EK‐42F & APU‐1R	1,500‐2,200	CTCAARGAYTAAGCCATGCA	CTTCCTTTGGTTAAAACAC
Amoebozoa	Tubulinea, Discosea, Variosea	18S, RibA & RibB	Entire SSU molecule, variable	ACCTGGTTGATCCTDCCAGT	TGATCCATCTGCAGGTTCACCTAC
	Tubulinea, Discosea, Variosea	18S, RibA & S20R	~1,800	ACCTGGTTGATCCTDCCAGT	GACGGGCGGTGTGTACAA
	Nearly all clades	Cox‐I, LCO1490 & HCO2198 (modified Folmer primers)	~660	GGTCAACAAATCATAAAGATATTGG	TAAACTTCAGGGTGACCAAAAAATCA
	Arcellinida	1st step, Euk 82F & Euk 1498 R, 2nd step, cloning	Variable	GAAACTGCGAATGGCTC	CYGCAGGTTCACCTA C
Opisthokonta	Choanoflagellata^2^	18S, 42F & 1510R	~1,750	CTCAARGAYTAAGCCATGCA	CCTTCYGCAGGTTCACCTAC
Porifera^3^	Demospongiae, Homoscleromorpha:	Cox‐I (Folmer primers), LCO1490 & HCO2198	658	GGTCAACAAATCATAAAGATATTGG	TAAACTTCAGGGTGACCAAAAAATCA
		28S, C1 & D2, universal primers	800–900	ACCCGCTGAATTTAAGCAT	TCCGTGTTTCAAGACGGG
		28S D1–D2, Por28S‐15F & Por28S‐878R	790–830	GCGAGATCACCYGCTGAAT	CACTCCTTGGTCCGTGTTTC
		28S D3–D5, Por28S‐830F & Por28S‐1520R	650‐660	CATCCGACCCGTCTTGAA	GCTAGTTGATTCGGCAGGTG
		28S D3‐D5, NL4F & NL4R	650–660	GACCCGAAAGATGGTG AACTA	ACCTTGGAGACCTGA TGCG
	Calcarea:	28S C‐region, C2 & D2, or C2'modified & D2	430–470	GAAAAGAACTTTGRARAGAGAGT or GAAA AGCACTTTGAAAAGAGA	TCCGTGTTTCAAGACGGG
	Hexactinellida:	28S D3‐D5, NL4F & NL4R	900–1,000	GACCCGAAAGATGGTG AACTA	ACCTTGGAGACCTGATGCG
		16S partial primers, 16S1fw & 16SH modified	500	TCGACTGTTTACCAAAAACATAGC	YRTAATTCAACATCGAGGTC
Fungi	Chytridiomycota^4^^,^ 5 ^,^ 6 ^,^ 7	18S, NS1 & NS4	~950–1,100		
		PolySSU1 & PolySSU1R	Variable		TGATCCTTCYGCAGGTTCACC
		28S, LROR & LR5	~800‐950		AACTAAGAACGGCCATGCAC
		ITS1‐5.8S‐ITS2, ITS5 & ITS4	~614–987		CCCGTGTTGAGTCAAATTAAGC
		EF‐1a,* 983F & EF1aZ‐1R	~1,150		GAACGGCCATGCACCACCACC
		EF‐1a‐like,** 983F & EFL‐RS2R	−~550–590		GTTCTTGTGTTAATCTCAC
Fungi^8^^,^ 9		ITS (See citation 9, Table S1)	Variable		
		18S, AU2‐F & AU4‐R, & inner AUPH1	TTTCGATGGTAGGATAGDGG	RTCTCACTAAGCCATTC, and inner AGAGCTMTCAATCTGTCAATCCT	ACTTCTGGRTGICCRAARAAYCA
Haptista	Haptophyta^10^^,^ 11	1F & 1528R (or EukA & EukB)	1,800	AACCTGGTTGATCCTGCCAGT	TGATCCTTCTGCAGGTTCACCTAC
			1,795	ACCTGGTTGATCCTGCCAG	TGATCCTTCYGCAGGTTCAC
		EukF & EukR	830	GGGTTCGATTCCGGAGAG	CCGTGTTGAGTCAAATT
		TAReuk454FWD1 & TAReukREV3	Variable	CCAGCA(G⁄C)C(C⁄T)GCGGTAATTCC	ACTTTCGTTCTTGATYRA
		Hapto4 & Euk34r	1,000	ATGGCGAATGAAGCGGGC	GCATCGCCAGTTCTGCTTACC
		LSU1 (Lhapto8 & Lhapto20R_bis)	350‐400	GGTATCGGAGAAGGTGAGAATCCT	TCAGACTCCTTGGTCCGTGTTTCT
		Prym03‐3 & Hapto1R	416	GTAAATTGCCCGAATCCTG	CGAAACCAACAAAATAGCAC
		528FLong & PRYM01 + 7	399	GCGGTAATTCCAGCTCCAA	GATCAGTGAAAACATCCCTGG
		Pavlova‐V4F & 1528R	904	GTGAAATTCTTAGACCCACGGA	TGATCCTTCTGCAGGTTCACCTAC
		1F & Pavlova‐V4F2R	593	See 1F above	GTGAAATTCTTAGACCCACGGA
		Pry421F & Pry1572R	1,070	AGCAGGCGCGTAAATTGCCCG	TCAACGYRCGCTGATGACA
		Hap220F & Pav1702R	1,400	ACCGGTCTCCGGTTGCGTGC	TAGATGATAAGGTTTGGGTG
	Centroplasthelida^12^	Helio1979R	Variable		CACACTTACWAGGAYTTCCTCGTTSAAGACG
Cryptista^13^^,^ 14		18S‐0024F & 18S‐1757R	Variable	CTGGTTGATCCTGCCAGTAGT	CAGGTTCACCTACGGAAACCT
		18S‐33F & 18S‐1768R	1,700‐1,800	CCT GCC AGT AGT CAT AYG CTT	TGA TCC TTC YGC AGG TTC ACC
Stramenopiles	Sar^15^	18S, SAR‐V3‐SSU F & R	150	TCGTCGGCAGCGTCAGATGTGTATAAGAGACA	ATGTGTATAAGAGACAGRACTACGAGCTTTTTAACTGC
	Euglyphid^16^	1st step EuglySSUF & EuglyLSUR	Variable	GCGTACAGCTCATTATATCAGCA	GTTTGGCACCTTAACTCGCG
		2nd step EuglySSUF & EuglySSUR	Variable	GCGTACAGCTCATTATATCAGCA	GCACCACCACCCATAGAATCWAGAAAGATC
	Assulinidae^17^	1st step, COI: Eucox1F & Euglycox1R	Variable	GAYATGGCKTTNCCAAGATTAAA	AGCACCCATTGAHAAAACRTAATG
		2nd step, Assucox 1F & Assucox 1R	Variable	AAYATGAGRGCYAGRGG	5¢‐CGTAATGAAARTGWCCYACC
	Amphitremida	1st step, Euk 82F & Euk 1498 R, 2nd step, cloning	Variable	GAAACTGCGAATGGCTC	CYGCAGGTTCACCTAC
	Diatomea^18^^,^ 19	rbcL, 646F& 998R	379	ATGCGTTGGAGAGARCGTTTC	GATCACCTTCTAATTTACCWACAACTG
		Diat_rbcL_708F (mixture of 3 primers) & two reverse primers R3_1 & R3_2	312 (amplicon 263)	1: AGGTGAAGTAAAAGGTTCWTACTTAAA, and 2: AGGTGAAGTTAAAGGTTCWTAYTTAAA and 3: AGGTGAAACTAAAGGTTCWTACTTAAA	1: CCTTCTAATTTACCWACWACTG, and 2: CCTTCTAATTTACCWACAACAG
Alveolata	Ciliophora^20^	18S V4,	Variable		
	Apicomplexa^21^^,^ 22	18S PF1 & R4	1,800	GCGCTACCTGGTTGATCCTGCC	GATCCTTCTGCAGGTTCACCTAC
		18S V4 TAReuk454FWD1 & TAReukREV3	Variable	ACTTTCGTTCTTGAT(C⁄T)(A⁄G)	ACTTTCGTTCTTGAT(C⁄T)(A⁄G)
		18S V4, 346Fmix & 785R‐mix	Variable	CADCGACGGGTAACGGGGAATTA; CAGYGACGGGTAACGGGGAATTA; CAGYGACGGGTAACGGGGAATTA; CAGYGACGGGTAACGGGGAATTA	IIITATTCCATGCTGIAGTATTCA; IIITATTCCATGCTAAASTATTCA
	Dinoflagellata**** ^,^ 23 ^,^ 24	18S V4, Next. For & Rev	Variable	TCGTCGGCAGCGTCAGATGTGTATAAGAGACAG[CCAGCASCYGCGGTAATTCC]	GTCTCGTGGGCTCGGAGATGTGTATAAGAGACAG[ACTTTCGTTCTTGATYRATGA]
	Syndiniales	18S V4, 528F & UnonMet	Variable		
Rhizaria	Cercozoa	18S V4, 3NDF & 1256R	~500	GGCAAGTCTGGTGCCAG	GCACCACCACCCAYAGAATCAAGAAAGAWCTTC
		18S V4***, 25F & 1256R	1,200	CATATGCTTGTCTCAAAGATTAAGCCA	GCACCACCACCCAYAGAATCAAGAAAGAWCTTC
	Cyphoderiidae^25^	Cox‐I, Eucox1F & Euglycox1R	Variable	GAYATGGCKTTNCCAAGATTAAA	AGCACCCATTGAHAAAACRTAATG
	Foraminifera^26^	18S, 14F1 & s17	300‐400	AAGGGCACCACAAGAACGC	CGGTCACGTTCGTTGC
Excavates	Fornicata^27^	EukA & EukB*	Variable		
	Parabasalia	16SI & 16S RR (or EukA & B)	Variable	TACTTGGTTGATCCTGCC	TCACCTACCGTTACCTTG
		ITS‐F & ITS‐R	Variable	TTCAGTTCAGCGGGTCTTCC	GTAGGTGAACCTGCCGTTGG
	Jakobida	EukA ‐ EukB	Variable		
	Heterolobosea	ITS1‐5.8S & ITS2, JITS‐F & JITS‐R, (or EukA & EukB)	Variable	GTCTTCGTAGGTGAACCTGC	CCGCTTACTGATATGCTTAA
	Preaxostyla: Oxymonadida	Mon‐F & Mon‐R	Variable	GAAGTCATATGCTGTCTCAA,	TCACCTACGGAAACCTT
	Preaxostyla: Paratrimastigida, Trimastigida	EukA & EukB	1,800–3,100	CTGGTTGATCCTGCCAG	TGATCCTTCTGCAGGTTCACCTAC
	Euglenida Heterotrophs^28^	See citation	Varies with the primer pairs		
	Euglenophyceae^28^	See citation	Varies with the primer pair		
Protist, general* ^,^ 27	General Medlin primers	EukA‐F & EukB‐R	Variable	CTGGTTGATCCTGCCAG	TGATCCTTCTGCAGGTTCACCTAC
Protist general^21^	General Stoeck primers	See citation	Variable		

* Selective amplification of species, some clades missed.

** Spizellomycetales.

*** Chytridiomycota except Spizellomycetales.

**** See citations for DINOREF in PR2 v.4.9.0.

1 Torruella, G., Moreira, D. & López‐García, P. 2017. Phylogenetic and ecological diversity of apusomonads, a lineage of deep‐branching eukaryotes *Env. Microbiol. Rep*. 9:113‐119. doi.org/10.1111/1758‐2229.12507.

2 Amaral‐Zettler, L.A., McCliment, E.A., Ducklow, H.W. & Huse, S.M. 2009. A Method for Studying Protistan Diversity Using Massively Parallel Sequencing of V9 Hypervariable Regions of Small‐Subunit Ribosomal RNA Genes. *PLOS one* 4 (12): https://doi.org/10.1371/journal.pone.0006372.

3 Morrow, C.C., Picton, B.E., Erpenbeck, D., Boury‐Esnault, N., Maggs, C.A. & Allcock, A.L. 2012. Congruence between nuclear and mitochondrial genes in Demospongiae: A new hypothesis for relationships within the G4 clade (Porifera: Demospongiae). *Molecular Phylogenetics and Evolution* 62: 174–190.

4 White, M.M., James, T.Y., O'Donnell, K., Cafaro, M.J., Tanabe, Y. & Sugiyama, J. 2006. Phylogeny of the Zygomycota based on nuclear ribosomal sequence data. *Mycologia*, 98(6): 872‐884. https://doi.org/10.1080/15572536.2006.11832617.

5 Simmons, D.R. 2011. Phylogeny of Powellomycetaceae fam. nov. and description of *Geranomyces variabilis* gen. et comb. Nov. *Mycologia* 103(6):1411‐1420.

6 Vilgalys, R. & Hester, M. 1990. Rapid genetic identification and mapping of enzymatically amplified ribosomal DNA from several Cryptococcus species. *J. Bacteriology* 172(8):4238‐4246.

7 James, T.Y., Letcher, P.M., Longcore, J.E., Mozley‐Standridge, S.E., Porter, D., Powell, M.J., Griffith, G.W. & Vilgalys, R. 2006. A molecular phylogeny of the flagellated fungi (Chytridiomycota) and description of a new phylum (Blastocladiomycota). *Mycologia* 98(6):860‐871.

8 Schoch, C.I., Seifert, K.A, Huhndorf, S., Robert, V., Spouge, J.L., Levesque, C.A., Chen, W. & Fungal Barcoding Consortium. 2012. Nuclear ribosomal internal transcribed spacer (ITS) region as a universal DNA barcode marker for Fungi. *PNAS* 109 (16): 6241‐6246; https://doi.org/10.1073/pnas.1117018109.

9 Vandenkoornhuyse, P., Baldauf, S.L., Leyval, C., Straczek, J. &. Young, J.P.W. 2002. Extensive Fungal Diversity in Plant Roots *Science* 295 (5562): 2051 https://doi.org/10.1126/science.295.5562.2051.

10 Egge, E, Bittner, L, Andersen, T, Audic, S, de Vargas, C. 2013. 454 Pyrosequencing to Describe Microbial Eukaryotic Community Composition, Diversity and Relative Abundance: A Test for Marine Haptophytes. *PLoS ONE* 8(9): e74371. https://doi.org/10.1371/journal.pone.0074371.

11 Edvardsen, B., Egge, E.S. & Vaulot, D. 2016. Diversity and distribution of haptophytes revealed by environmental sequencing and metabarcoding – a review. *Perspectives in Phycology* 3 (2): 77–91.

12 Cavalier‐Smith, T. & von der Heyden, S. 2007. Molecular phylogeny, scale evolution and taxonomy of centrohelid heliozoan. *Molecular Phylogenetics and Evolution* 44(3): 1186‐1203. https://doi.org/10.1016/j.ympev.2007.04.019.

13 Kim, E., Simpson, A.G.B. & Graham, L.E. 2006. Evolutionary relationships of apusomonads inferred from taxon‐rich analyses of 6 nuclear encoded genes. *Molec. Biol. Evol*. 23(12):2455‐2466.

14 Kim, E. & Archibald, J. 2013. Ultrastructure and molecular phylogeny of the Cryptomonad *Gonimonas avonlea* sp. Nov. *Protist* 164(2):160‐182.

15 Sisson, C., Gulla‐Devaney, B., Katz, L.A. & Grattepanche, J‐D. 2018. Seed bank and seasonal patterns of eukaryotic SAR (Stramenopila, Alveolata, Rhizaria) clade in a New England vernal pool. *J. Plankton Res*. 00(00): 1–15. https://doi.org/10.1093/plankt/fby020.

16 Lara, E., Roussel‐Delif, L., Fournier, B., Wilkinson, D.M. & Mitchell, E.A.D. 2016. Soil microorganisms behave like macroscopic organisms: Patterns in the global distribution of soil euglyphid testate amoebae. *Journal of Biogeography* 43(3):520‐532.

17 Lara, E., Heger, T.J., Scheihing, R. & Mitchell, E.A.D. 2011. COI gene and ecological data suggest size‐dependent high dispersal and low intra‐specific diversity in free‐living terrestrial protists (Euglyphida: Assulina). *Journal of Biogeography* 38(4): 640‐650.

18 Kelly, M., Boonham, N., Juggins, S., Kille, P., Mann, D., Pass, D., Sapp, M., Sato, S. & Glover, R. 2018. A DNA based diatom metabarcoding approach for Water Framework Directive classification of rivers. *SC140024/R,* Environment Agency, Bristol. ISBN: 978‐1‐84911‐406‐6.

19 Vasselon, V., Rimet, F., Tapolczai, K. & Bouchez, A. 2017. Assessing ecological status with diatoms DNA metabarcoding: scaling up on a WFD monitoring network (Mayotte island, France). *Ecol. Indicators* 82: 1–12.

20 Lara, E., Berney, C., Harms, H. & Chatzinotas, A. 2007. Cultivation‐independent analysis reveals a shift in ciliate 18S rRNA gene diversity in a polycyclic aromatic hydrocarbon‐polluted soil. *FEMS Microbiol. Ecol*. 62:365‐373.

21 Stoeck, T., Bass, D., Nebel, Christen, R., Jones, M.C.M., Breiner, H‐W. & Richards, T. 2010. Multiple marker parallel tag environmental DNA sequencing reveals a highly complex eukaryotic community in marine anoxic water. *Mol. Ecol*. 19(1), 21‐31.

22 Janouškovec, J., Tikhonenkov, D.V., Burki, F., Howe, A.T., Kolisko, M., Mylnikov, A.P., & Keeling, P. 2015. Factors mediating plastid dependency and the origins of parasitism in apicomplexans and their close relatives. *PNAS* 112(33), 10200–10207.

23 Piredda R., Tomasino, M.P., D'Erchia, A.M., Manzari, C., Pesole, G., Montresor, M., Kooistra, W.H.C F., Sarno, D. & Zingone, A. 2017. Diversity and temporal patterns of planktonic protist assemblages at a Mediterranean Long Term Ecological Research site *FEMS Microbiology Ecology*, 93(1), fiw200, doi.org/10.1093/femsec/fiw200.

24 Mordret, S., Piredda, R., Vaulot, D., Montresor, M., Kooistra, W.H.C.F. & Sarno. D. DINOREF: A curated dinoflagellate (Dinophyceae) reference database for the 18S rRNA gene. *Mol Ecol Resour*. 2018;1–14. https://doi.org/10.1111/1755-0998.12781.

25 Heger, T., Pawlowski, J., Lara, E., Leander, B.S., Todorov, M., Golemansky, V. & Mitchell, E.A.D. 2011. Comparing potential COI and SSU rDNA barcodes for assessing the diversity and phylogenetic relationships of cyphoderiid testate amoebae (Rhizaria: Euglyphida). *Protist* 162(1):131‐141.

26 Pawlowski, J. & Lecroq, B. 2010. Short rDNA barcodes for species identification in foraminifera. *J. Eukaryot. Microbiol*. 57(2):197‐205.

27 Medlin, L., Elwood, H.J., Stickel, S. & Sogin, M.L. 1988. The characterization of enzymatically amplified eukaryotic 16S‐like rRNA‐coding regions. *Gene* 71(2): 491‐499. https://doi.org/10.1016/0378-1119(88)90066-2.

28 Lax, G. & Simpson, AGB. 2013. Combining molecular data with classical morphology for uncultured phagotrophic euglenids (Excavata): a single cell approach. *J. Eukaryot. Microbiol.*. 60(6):615‐25. https://doi.org/10.1111/jeu.12068.

## Appendices

**Revisions to the Classification, Nomenclature, and Diversity of Eukaryotes** by Sina M. Adl, David Bass, Christopher E. Lane, Julius Lukeš, Conrad L. Schoch, Alexey Smirnov, Sabine Agatha, Cedric Berney, Matthew W. Brown, Fabien Burki, Paco Cárdenas, Ivan Čepička, Ludmila Chistyakova, Javier del Campo, Micah Dunthorn, Bente Edvardsen, Yana Eglit, Laure Guillou, Vladimír Hampl, Aaron A. Heiss, Mona Hoppenrath, Timothy Y. James, Anna Karnkowska, Sergey Karpov, Eunsoo Kim, Martin Kolisko, Alexander Kudryavtsev, Daniel J. G. Lahr, Enrique Lara, Line Le Gall, Denis H. Lynn, David G. Mann, Ramon Massana i Molera, Edward A. D. Mitchell, Christine Morrow, Jong Soo Park, Jan W. Pawlowski, Martha J. Powell, Daniel J. Richter, Sonja Rueckert, Lora Shadwick, Satoshi Shimano, Frederick W. Spiegel, Guifré Torruella i Cortes, Noha Youssef, Vasily Zlatogursky, Qianqian Zhang.

Appendix S1. Supplementary references.

Appendix S2. Functional group assignments.

Appendix S3. Translation guide for East Asian users.

## Appendix S1. Selected Literature mostly since 2012.

## AMORPHEA

Berney, C., Geisen, S., Van Wichelen, J., Nitsche, F., Vanormelingen, P., Bonkowski, M. & Bass, D. 2015. Expansion of the “reticulosphere”: diversity of novel branching and network‐forming amoebae helps to define Variosea (Amoebozoa). *Protist*,** 166**, 271–295.

Brown, M. W., Sharpe, S. C., Silberman, J. D., Heiss, A. A., Lang, B. F., Simpson, A. G. B. & Roger, A. J. 2013. Phylogenomics demonstrates that breviate flagellates are related to opisthokonts and apusomonads. *Proc. R. Soc. Lond. B*,** 280**:1769.

Cavalier‐Smith, T., Chao, E., E., & Lewis, R. 2016. 187‐Gene phylogeny of protozoan phylum Amoebozoa reveals a new class (Cutosea) of deep‐branching, ultrastructurally unique, enveloped marine Lobosa and clarifies amoeba evolution. *Mol. Phylogenet. Evol*., **99**: 275–296.

Hamann, E., Gruber‐Vodicka, H., Kleiner, M., Tegetmeyer, H. E., Riedel, D., Littmann, S., Chen, J., Milucka, J., Viehweger, B., Becker, K. W., Dong, X., Stairs, C. W., Hinrichs, K.‐U., Brown, M. W., Roger, A. J. & Strous, M. 2016. Environmental Breviatea harbor mutualistic Acrobacter epibionts. *Nature*,** 534**:254–258.

Heiss, A. A., Lee, W. J., Ishida, K. & Simpson, A. G. B. 2015. Cultivation and characterisation of new species of apusomonads (the sister group to opisthokonts), including close relatives of *Thecamonas* (*Chelonemonas* n. gen.). *J. Eukaryot. Microbiol*., **62**:637‐649.

Kang, S., Tice, A.K., Spiegel, F. W., Silberman, J. D., Pánek, T., Čepička, I., Kostka, M., Kosakyan, A., Alcântara, D. M., Roger, A. J., Shadwick, L. L., Smirnov, A., Kudryavstev, A., Lahr, D. J., & Brown, M. W. 2017. Between a pod and a hard test: the deep evolution of amoebae. *Mol Biol Evol* msx162. https://doi.org/10.1093/molbev/msx162.

Pánek, T, Zadrobílková, Walker, G., Brown, M. W., Gentekaki, E., Hroudrová, M., Kang, S. Roger, A. J., Tice, A. K., Vlček, Č., & Čepička, I. 2016. First multigene analysis of Archamoebae (Amoebozoa: Conosa) robustly reveals its phylogeny and shows that Entamoebidae represents a deep lineage of the group. Molec. Phylogen. Evol., **98**:41‐51.

Schaap, P., Winckler, T., Nelson, M., Alvarez‐Curto, E., Elgie, B., Hagiwara, H., Cavender, J., Milano‐Curto, A., Rozen, D. E., Dingermann, T., Mutzel, R. & Baldauf, S. 2006. Molecular phylogeny and evolution of morphology in the social amoebas. Science, 314: 661‐663.

Spiegel, F. W.**,** Shadwick, L. L., Ndiritu, G. G., Brown, M. W., Aguilar, M., & Shadwick, J. D. L. 2017. Protosteloid Amoeboazoa (Protosteliids, Protosporangiida, Cavostellida, Schizoplasmodiida, Fractoviteliida, and sporcarpic members of Vanellida, Centramoebida, and Pellitida). In: Archibald, J. M.,Simpson, A. G. B., and Slamovits, C., eds. Handbook of the Protists (Second Edition of the Handbook of Protoctista by Margulis et al.) Springer Reference Works (e‐book) https://doi.org/10.1007/978-3-319-32669-6_12-1

Sheikh, S., MatsThulin Cavender, J.C., Escalante, R., Kawakami, S.I., Lado, C., Landolt, J.C., Nanjundiah, V., Queller, D.C., Strassmann, J.E., Spiegel, F.W., Stephenson, S.L., Vadell, S.M., & Baldauf, S.L. 2018. A new classification of the dictyostelids. *Protist* **169**: 1‐28.

Wilkinson, D. M. & Mitchell, E. A. D. 2010. Testate amoebae and nutrient cycling with particular reference to soils. Geomicrobiol J., 27(6):520‐533. https://doi.org/10.1080/01490451 003702925.

Walker, G., Zadrobílková, E., Čepička., I. (2017) Archamoebae In: Archibald, J. M.,Simpson, A. G. B., & Slamovits, C., eds. Handbook of the Protists (Second Edition of the Handbook of Protoctista by Margulis et al.) Springer Reference Works (e‐book) https://doi.org/10.1007/978-3-319-28149-0_11

## AMOEBOZOA

Berney, C., Geisen, S., Van Wichelen, J., Nitsche, F., Vanormelingen, P., Bonkowski, M. & Bass, D. 2015. Expansion of the “reticulosphere”: diversity of novel branching and network‐forming amoebae helps to define Variosea (Amoebozoa). *Protist*,** 166**, 271–295.

Cavalier‐Smith, T., Chao, E., E., & Lewis, R. 2016. 187‐Gene phylogeny of protozoan phylum Amoebozoa reveals a new class (Cutosea) of deep‐branching, ultrastructurally unique, enveloped marine Lobosa and clarifies amoeba evolution. *Mol. Phylogenet. Evol*., **99**: 275–296.

Kang, S., Tice, A.K., Spiegel, F. W., Silberman, J. D., Pánek, T., Čepička, I., Kostka, M., Kosakyan, A., Alcântara, D. M., Roger, A. J., Shadwick, L. L., Smirnov, A., Kudryavstev, A., Lahr, D. J. & Brown, M. W. 2017. Between a pod and a hard test: the deep evolution of amoebae. *Mol Biol Evol* msx162. https://doi.org/10.1093/molbev/msx162.

Pánek, T, Zadrobílková, Walker, G., Brown, M. W., Gentekaki, E., Hroudrová, M., Kang, S. Roger, A. J., Tice, A. K., Vlček, Č., & Čepička, I. 2016. First multigene analysis of Archamoebae (Amoebozoa: Conosa) robustly reveals its phylogeny and shows that Entamoebidae represents a deep lineage of the group. *Molec. Phylogenet. Evol.,* **98**:41‐51.

Schaap, P., Winckler, T., Nelson, M., Alvarez‐Curto, E., Elgie, B., Hagiwara, H., Cavender, J., Milano‐Curto, A., Rozen, D. E., Dingermann, T., Mutzel, R. & Baldauf, S. 2006. Molecular phylogeny and evolution of morphology in the social amoebas. Science, **314**: 661‐663.

Spiegel, F. W., Shadwick, L. L., Ndiritu, G. G., Brown, M. W., Aguilar, M. & Shadwick, J. D. L. 2017. Protosteloid Amoeboazoa (Protosteliids, Protosporangiida, Cavostellida, Schizoplasmodiida, Fractoviteliida, and sporcarpic members of Vanellida, Centramoebida, and Pellitida). In: Archibald, J. M.,Simpson, A. G. B., and Slamovits, C., eds. Handbook of the Protists (Second Edition of the Handbook of Protoctista by Margulis et al.) Springer Reference Works (e‐book) https://doi.org/10.1007/978-3-319-32669-6_12-1

Sheikh, S., MatsThulin Cavender, J.C., Escalante, R., Kawakami, S.I., Lado, C., Landolt, J.C., Nanjundiah, V., Queller, D.C., Strassmann, J.E., Spiegel, F.W., Stephenson, S.L., Vadell, S.M. & Baldauf, S.L. 2018. A new classification of the dictyostelids. *Protist* **169**: 1‐28.

Smirnov, A., V., Brown, S., 2004. Guide to the methods of study and identification of soil gymnamoebae. *Protistology* **3**, 148–190.

Wilkinson, D. M. & Mitchell, E. A. D. 2010. Testate amoebae and nutrient cycling with particular reference to soils. Geomicrobiol J., **27**(6):520‐533. https://doi.org/10.1080/01490451003702925.

Walker, G., Zadrobílková, E. & Čepička., I. 2017 Archamoebae In: Archibald, J. M.,Simpson, A. G. B. & Slamovits, C., eds. Handbook of the Protists (Second Edition of the Handbook of Protoctista by Margulis et al.) Springer Reference Works (e‐book) https://doi.org/10.1007/978-3-319-28149-0_11

## OPISTHOKONTA

### Holozoa

Grau‐Bové, X., Torruella, G., Donachie, S., Suga, H., Leonard, G., Richards, T.A. & Ruiz‐Trillo, I. 2017. Dynamics of genomic innovation in the unicellular ancestry of animals. *eLife* 6**,** e26036.

Hehenberger, E., Tikhonenkov, D.V., Kolisko, M., del Campo, J., Esaulov, A.S., Mylnikov, A.P., & Keeling, P.J., 2017. Novel predators reshape holozoan phylogeny and reveal the presence of a two‐ component signaling system in the ancestor of animals. *Curr. Biol.,* **27:** 2043‐2050.

Toruella, G., Mendoza, A., Grau Bauvé, X., Anto, M., Chaplin, M., del Campo, J., Eme, L., Perez Cordon, G., Whipps, C.M., Nichols, K.M., Paley, R., Roger, A.J., Sitja‐Bobadillas, A., Donachie, S., & Ruiz‐ Trillo, I. 2015. Phylogenomics Reveals Convergent Evolution of Lifestyles in Close Relatives of Animals and Fungi. *Curr. Biol., **25***: 2404‐2410. https://doi.org/10.1016/j.cub.2015.07.053

### Choanoflagellata

Brunet, T. & King, N. 2017 The origin of animal multicellularity and cell differentiation. *Developmental Cell,* **43**: 124‐140. https://doi.org/10.1016/j.devcel.2017.09.016

Budd, G.E. & Jensen, S. 2017. The origin of the animals and a “Savannah” hypothesis for early bilaterian evolution *Biol. Rev. Camb Philos. Soc*., **92:** 446‐473.

Carr, M., Richter, D.J., Fozouni, P., Smith, T.J., Jeuck, A., Leadbeater, B.S.C. & Nitsche, F., 2017. A six‐gene phylogeny provides new insights into choanoflagellate evolution. *Mol. Phylogenetics Evol.,* **107**: 166–178.

Nitsche, F., Carr, M., Arndt, H. & Leadbeater, B. S. C. 2011. Higher level taxonomy and molecular phylogenetics of the Choanoflagellatea. *J.Eukaryot. Microbiol.,* **58**: 452‐462. https://doi.org/10.1111/j.1550‐ 7408.2011.00572.x

Richter, D.J. & Nitsche, F., 2017. Choanoflagellatea. In: Archibald, J.M., Simpson, A.G.B. & Slamovits, C.H. (Eds.) Handbook of the Protists. Springer International Publishing, pp. 1479‐1496.

### Porifera

Cárdenas, P., Pérez, T. & Boury‐Esnault, N. 2012. Sponge Systematics facing new challenges. *Adv.Marine Biol.,* **61:** 79‐209.

Dohrmann, M., Kelley, C., Kelly, M., Pisera, A., Hooper, J. N. A. & Reiswig, H. M. 2017. An integrative systematic framework helps to reconstruct skeletal evolution of glass sponges (Porifera, Hexactinellida). *Frontiers Zool.,* **14**: 18.

Morrow, C. & Cárdenas, P. 2015. Proposal for a revised classification of the Demospongiae (Porifera). *Frontiers Zool.,* **12**: 1‐27.

Redmond, N. E., Morrow, C. C., Thacker, R. W., Diaz, M. C., Boury‐Esnault, N., Cárdenas, P., et al. 2013. Phylogeny and Systematics of Demospongiae in Light of New Small Subunit Ribosomal DNA (18S) Sequences. *Integr. Comparat. Biol.,* **53**: 388‐415.

Ruiz, C., Muricy, G., Lage, A., Domingos, C., Chenesseau, S. & Pérez, T. 2017. Descriptions of new sponge species and genus, including aspiculate Plakinidae, overturn the Homoscleromorpha classification. *Zool. J. Linn. Soc*., **179**: 707‐724.

Voigt, O., Wülfing, E. & Wörheide, G. 2012. Molecular Phylogenetic Evaluation of Classification and Scenarios of Character Evolution in Calcareous Sponges (Porifera, Class Calcarea). *PLoS ONE*,** 7**, e33417.

### Metazoa

Cannon, J.T., Vellutini, B.C., Smith, J., Ronquist, F., Jondelius, U. & Hejnol, A. 2016. Xenacoelomorpha is the sister group to Nephrozoa. *Nature,* **530**: 89‐93.

Laumer, C.E., Bekkouche, N., Kerbl, A., Goetz, F., Neves, R.C., Sørenson, M.V., Kristensen, R.M., Hejnol, A., Dunn, C.W., Giribet, G. & Worsaae, K. 2015. Spiralian phylogeny informs the evolution of microscopic lineages. *Curr. Biol.,* **25:** 2000‐2006.

Simion, P., Philippe, H., Baurain, D., Jager, M., Richter, D.J., Di Franco, A., Roure, B., Satoh, N., Queinnec, E., Ereskovsky, A., Lapebie, P., Corre, E., Delsuc, F., King, N., Wörheide, G. & Manuel, M. 2017. A large and consistent phylogenomic dataset supports sponges as the sister group to all other animals. *Curr. Biol*., 27: 958‐967.

Whelan, N.V., Kocot, K.M., Moroz, T.P., Mukherjee, K., Williams, P., Paulay, G., Moroz, L.L. & Halanych, K.M. 2017. Ctenophore relationships and their placement as the sister group to all other animals. *Nat. Ecol. Evol.,* **1**: 1737‐1746.

### Holomycota

Bass, D., Czech, L., Williams, B.A.P., Berney, C., Dunthorn, M., Mahé, F., Torruella, G., Stentiford, G.D., Williams, T.A. 2018. Clarifying the Relationships between Microsporidia and Cryptomycota. *J. Euk. Microbiol.,* **65**: 773–782. https://doi.org/10.1111/jeu.12519

Jones, M. D. M., Richards, T. A., Hawksworth, D. L. & Bass, D. 2011. Validation and justification of the phylum name Cryptomycota. *IMA Fungus*,** 2**:173–175.

Karpov, S. A., Mamkaeva, M. A., Aleoshin, V. V., Nassonova, E., Lilje, O. & Gleason, F. H. 2014.Morphology, phylogeny, and ecology of the aphelids (Aphelidea, Opisthokonta) and proposal for the new superphylum Opisthosporidia.*Front. Microbiol*., **5**:112.

Lara, E., Moriera, D. & Lopez‐Garcia, P. 2010. The environmental clade LKM11 and *Rozella* form the deepest branching clade of fungi. *Protist*,** 161**:116–121.

Liu, Y., Steenkamp, E.T., Brinkmann, H., Forget, L., Philippe, H. & Lang, F.B. 2009. Phylogenomic analyses predict sistergroup relationship of nucleariids and Fungi and paraphyly of zygomycetes with significant support. *BMC Evol. Biol.,* **20099**:272, https://doi.org/10.1186/1471-2148-9-272.

### Fungi

Bauer, R., Garnica, S., Oberwinkler, F., Riess, K., Weiss, M. & Begerow, D. 2015. Entorrhizomycota: A New Fungal Phylum Reveals New Perspectives on the Evolution of Fungi. *PLoS One*,** 10**:e0128183.

Chang, Y., Wang, S. S., Sekimoto, S., Aerts, A. L., Choi, C., Clum, A., LaButti, K. M., Lindquist, E. A., Ngan, C. Y., Ohm, R. A., Salamov, A. A., Grigoriev, I. V., Spatafora, J. W. & Berbee, M. L. 2015. Phylogenomic Analyses Indicate that Early Fungi Evolved Digesting Cell Walls of Algal Ancestors of Land Plants. *Genome Biol. . Evol*., **7**:1590‐1601.

Edlind, T. D., Li, J., Visvesvara, G. S., Vodkin, M. H., McLaughlin, G. L. & Katiyar, S. K. 1996. Phylogenetic analysis of beta‐tubulin sequences from amitochondrial protozoa. *Mol. Phylogenet. Evol*., **5**:359‐67.

Hibbett, D. S., Binder, M., Bischoff, J. F., Blackwell, M., Cannon, P. F., Eriksson, O. E., Huhndorf, S., James, T., Kirk, P. M., Lücking, R., Lumbsch, T., Lutzoni, F., Matheny, P. B., Mclaughlin, D. J., Powell, M. J., Redhead, S., Schoch, C. L., Spatafora, J. W., Stalpers, J. A., Vilgalys, R., Aime, M. C., Aptroot, A., Bauer, R., Begerow, D., Benny, G. L., Castlebury, L. A., Crous, P. W., Dai, Y.‐C., Gams, W., Geiser, D. M., Griffith, G. W., Gueidan, C., Hawksworth, D. L., Hestmark, G., Hosaka, K., Humber, R. A., Hyde, K., Ironside, J. E., Kõljalg, U., Kurtzman, C. P., Larsson, K.‐H., Lichtwardt, R., Longcore, J., Miadlikowska, J., Miller, A., Moncalvo, J.‐M., Mozley‐Standridge, S., Oberwinkler, F., Parmasto, E., Reeb, V., Rogers, J. D., Roux, C., Ryvarden, L., Sampaio, J. P., Schüßler, A., Sugiyama, J., Thorn, R. G., Tibell, L., Untereiner, W. A., Walker, C., Wang, Z., Weir, A., Weiß, M., White, M. M., Winka, K., Yao, Y.‐J. & Zhang, N. 2007. A higher‐level phylogenetic classification of the Fungi. *Mycological Res*., **111**:509‐547.

Hibbett, D. S., Blackwell, M., James, T. Y., Spatafora, J. W., Taylor, J. W. & Vilgalys, R. 2018. Phylogenetic taxon definitions for Fungi, Dikarya, Ascomycota and Basidiomycota. *IMA Fungus*,** 9**: 291–298.

James, T. Y., Kauff, F., Schoch, C. L., Matheny, P. B., Hofstetter, V., Cox, C. J., Celio, G., Gueidan, C., Fraker, E., Miadlikowska, J., Lumbsch, H. T., Rauhut, A., Reeb, V., Arnold, A. E., Amtoft, A., Stajich, J. E., Hosaka, K., Sung, G.‐H., Johnson, D., O'Rourke, B., Crockett, M., Binder, M., Curtis, J. M., Slot, J. C., Wang, Z., Wilson, A. W., Schuszler, A., Longcore, J. E., O'Donnell, K., Mozley‐Standridge, S., Porter, D., Letcher, P. M., Powell, M. J., Taylor, J. W., White, M. M., Griffith, G. W., Davies, D. R., Humber, R. A., Morton, J. B., Sugiyama, J., Rossman, A. Y., Rogers, J. D., Pfister, D. H., Hewitt, D., Hansen, K., Hambleton, S., Shoemaker, R. A., Kohlmeyer, J., Volkmann‐Kohlmeyer, B., Spotts, R. A., Serdani, M., Crous, P. W., Hughes, K. W., Matsuura, K., Langer, E., Langer, G., Untereiner, W. A., Lucking, R., Budel, B., Geiser, D. M., Aptroot, A., Diederich, P., Schmitt, I., Schultz, M., Yahr, R., Hibbett, D. S., Lutzoni, F., McLaughlin, D. J., Spatafora, J. W. & Vilgalys, R. 2006. Reconstructing the early evolution of Fungi using a six‐gene phylogeny. *Nature*,** 443**:818‐822.

Jones, M. D. M., Richards, T. A., Hawksworth, D. L. & Bass, D. 2011. Validation and justification of the phylum name Cryptomycota phyl. nov. *IMA Fungus*,** 2**:173‐175.

Jones, M. D. M., Forn, I., Gadelha, C., Egan, M. J., Bass, D., Massana, R. & Richards, T. A. 2011. Discovery of novel intermediate forms redefines the fungal tree of life. *Nature*,** 474**:200‐203.

Karpov, S. A., Mamkaeva, M. A., Aleoshin, V. V., Nassonova, E., Lilje, O. & Gleason, F. H. 2014. Morphology, phylogeny, and ecology of the aphelids (Aphelidea, Opisthokonta) and proposal for the new superphylum Opisthosporidia. *Front. Microbiol*., **5**:112.

Keeling, P. J. 2003. Congruent evidence from alpha‐tubulin and beta‐tubulin gene phylogenies for a zygomycete origin of microsporidia. Fungal Genet Biol, 38:298‐309.

Kirk, P. M., Cannon, P. F., Minter, D. W. & Stalpers, J. A. 2008. Ainsworth and Bisby's dictionary of the Fungi, 10th ed. CAB International., Wallingford , UK p. 771.

Lara, E., Moreira, D. & Lopez‐Garcia, P. 2010. The Environmental Clade LKM11 and *Rozella* Form the Deepest Branching Clade of Fungi. *Protist*,** 161**:116‐121.

Lee, S. C., Corradi, N., Doan, S., Dietrich, F. S., Keeling, P. J. & Heitman, J. 2010. Evolution of the sex‐related locus and genomic features shared in Microsporidia and Fungi. *Plos ONE*,** 5**:e10539.

Liu, Y., Steenkamp, E. T., Brinkmann, H., Forget, L., Philippe, H. & Lang, B. F. 2009. Phylogenomic analyses predict sistergroup relationship of nucleariids and fungi and paraphyly of zygomycetes with significant support. *BMC Evol. Biol*., **9**:272.

Ren, R., Sun, Y., Zhao, Y., Geiser, D., Ma, H. & Zhou, X. 2016. Phylogenetic Resolution of Deep Eukaryotic and Fungal Relationships Using Highly Conserved Low‐Copy Nuclear Genes. *Genome Biol. Evol*., **8**:2683‐701.

Spatafora, J. W., Chang, Y., Benny, G. L., Lazarus, K., Smith, M. E., Berbee, M. L., Bonito, G., Corradi, N., Grigoriev, I., Gryganskyi, A., James, T. Y., O'Donnell, K., Roberson, R. W., Taylor, T. N., Uehling, J., Vilgalys, R., White, M. M. & Stajich, J. E. 2016. A phylum‐level phylogenetic classification of zygomycete fungi based on genome‐scale data. *Mycologia*,** 108**:1028‐1046.

Tedersoo, L., Sanchez‐Ramirez, S., Koljalg, U., Bahram, M., Doring, M., D., S., May, T., Ryberg, M. & Abarenkov, K. 2018. High‐level classification of the Fungi and a tool for evolutionary ecological analyses. *Fungal Diversity*,** 90**:135‐159.

### Chytridiomycota

Barr, D. J. S. 1980. An outline for the reclassification of the Chytridiales, and for a new order, the Spizellomycetales. *Can. J. Bot.,* **58**:2380–2394.

Karpov, S. A., Kobseva, A. A., Mamkaeva, M. A., Mamkaeva, K. A., Mikhailov, K. V.., Mirzaeva, G. S. & Aleoshin, V. V. 2014. *Gromochytrium mamkaevae* gen. & sp. nov. and two new orders: Gromochytriales and Mesochytriales (Chytridiomycetes). *Personia,* **32**:115–126.

Letcher, P. M., Powell, M. J., Churchill, P. F. & Chambers, J. G. 2018. Ultrastructural and molecular phylogenetic delineation of a new order, the Rhizophydiales (Chytridiomycota). *Mycol. Res*., **110**:898–915.

Letcher P. M., Powell, M. J., Barr, D. J. S., Churchill, P. F., Wakefield, W. S. & Picard, K. T. 2008. Rhizophlyctidales–a new order in Chytridiomycota. *Mycol. Res*., **112**:1031–1048.

Longcore, J. E. & Simmons, D. R. 2012. The Polychytriales ord. nov. contains chitinophilic members of the rhizophlyctoid alliance. *Mycologia*,** 104**: 276–294**.**

Longcore, J. E., Simmons, D. R. & Letcher, P. M. 2016. *Synchytrium microbalum* sp. nov. is a saprobic species in a lineage of parasites. *Fungal Biol*., **120**:1156–1164.

Mozley‐Standridge, S. E., Letcher, P. M., Longcore, J. E., Porter, D. & Simmons, D. R. 2009. Cladochytriales–a new order in the Chytridiomycota. *Mycol. Res*., **113**:498–507.

Simmons, D. R., James, T. Y., Meyer, A. F. & Longcore, J. E. 2009. Lobulomycetales, a new order in the Chytridiomycota. *Mycol. Res.,* **113**:450–460.

Smith, D.S., Rocheleau, H., Chapados, J. T., Abbott, C., Ribero, S., Redhead, S. A., Lévesque, C. A., & De Boer, S. H., 2014. Phylogeny of the genus *Synchytrium* and the development of TaqMan PCR assay for sensitive detection of *Synchytrium endobioticum* in soil. *Phytopath*., **104**:422–432.

Torruella, G., Grau‐Bové, X., Moreira, D., Karpov, S. A., Burns, J. A., Sebé‐Pedrós, A., Völcker E. & López‐García, P. 2019. Global transcriptome analysis of the aphelid Paraphelidium tribonemae supports the phagotrophic origin of fungi. Communications Biology. **1**: 231, https://doi.org/10.1038/s42003-018-0235-z

Vélez, C. G., Letcher, P. M., Schultz, S., Powell, M. J. & Churchill, P. F. 2011. Molecular phylogenetic and zoospore ultrastructural analyses of *Chytridium olla* establish the limits of a monophyletic Chytridiales. *Mycologia,* **103**:118–130.

## DIAPHORETICKES ARCHAEPLASTIDA

### Chloroplastida

Fawley, M.V., Yun, Y., & Qin, M. 2000. Phylogenetic analyses of 18S rDNA sequences reveal a new coccoid lineage of the Prasinophyceae (Chlorophyta). *J. Phycol*., **36**:387‐393.

Fucikova, K., Leliaert, F., Cooper, E.D., Skaloud, P., D'Hondt, S., De Clerk, O., Gurgel, C.F.D., Lewis, L.A., Lewis, P.O., Lopez‐Bautista, J.M., Delwiche, C.F., Verbruggen, H. 2014. New phylogenetic hypotheses for the core Chlorophyta based on chloroplast sequence data. *Frontiers Ecol. Evol.,* **2**:67, https://doi.org/10.3389/fevo.2014.00063

Leliaert. F., Tronholm, A., Lemieux, C., Bhattacharya, D., Karol, K.G., Fredericq, S. 2016. Chloroplast phylogenomic analyses reveal the deepest‐branching lineage of the Chlorophyta, Palmophyllophyceae class. nov. *Sci. Rep*., **6**:25367, https://doi.org/10.1038/srep25367

Lopes Dos Santos, A., Pollina, T., Gourvil, P., Corre, E., Marie, D., Garrido J.L., Rodríguez, F., Noël, M.H. & Vaulot, D., Eikrem W. 2017. Chloropicophyceae, a new class of picophytoplanktonic prasinophytes. *Sci. Rep*., **25**:14019, https://doi.org/10.1038/s41598-017-12412-5

Nakayama, T., Marin, B., Kranz, H.D., Surek, B., Huss, V.A., Inouye, I. & Melkonian, M. 1998. The basal position of scaly green flagellates among the green algae (Chlorophyta) is revealed by analyses of nuclear‐encoded SSU rRNA sequences. *Protist*,** 149**:378‐380.

### Rhodophyceae and Glaucophyta

Muñoz‐Gómez, S.A., Mejía‐Franco, F.G., Durnin, K., Colp, M., Grisdale, C.J., Archibald, J.M. & Slamovits C.H. 2017. The New Red Algal Subphylum Proteorhodophytina Comprises the Largest and Most Divergent Plastid Genomes Known. *Curr. Biol*., **27**: 1677–1684.

Chong, J., Jackson, C., Kim, J. I., Yoon, H. S. & Reyes‐Prieto, A. 2014. Molecular markers from different genomic compartments reveal cryptic diversity within glaucophyte species. *Mol. Phylogenet. Evol*., **76**:181–188.

Jackson, C., Clayden, S. & Reyes‐Prieto, A. 2015. The Glaucophyta: The blue‐green plants in a nutshell. *Acta Soc. Bot. Pol.,* **84**:149–165.

Jackson C.J., Reyes‐Prieto A. 2014. The Mitochondrial Genomes of the Glaucophytes *Gloeochaete wittrockiana* and *Cyanoptyche gloeocystis*: Multilocus Phylogenetics Suggests a Monophyletic Archaeplastida. *Gen. Biol. Evol.,* **6:** 2774–2785.

Price, D.C., Chan, C.X., Yoon, H.S., Yang, E.C., Qiu, H., Weber, A.P.M., Schwacke, R., Gross, J., Blouin, N.A., Lane, C., Reyes‐Prieto, A., Durnford, D.G., Neilson, J.A.D., Lang, B.F., Burger, G., Steiner, J.M., Loffelhardt, W., Meuser, J.E., Posewitz, M.C., Ball, S., Arias, M.C., Henrissat, B., Coutinho, P.M., Rensing, S.A., Symeonidi, A., Doddapaneni, H., Green, B.R., Rajah, V.D., Boore, J. & Bhattacharya, D. 2012. *Cyanophora paradoxa* genome elucidates origin of photosynthesis in algae and plants. *Science,* **335**:843–847.

Takahashi, T., Sato, M., Toyooka, K., Matsuzaki, R., Kawafune, K., Kawamura, M., Okuda, K. & Nozaki, H. 2014. Five *Cyanophora* (Cyanophorales, Glaucophyta) species delineated based on morphological and molecular data. *J. Phycol.,* **50**:1058–1069.

Takahashi, T., Nishida, T., Tuji, A., Saito, C., Matsuzaki, R., Sato, M., Toyooka, K., Yasuda, H. & Nozaki, H. 2016. Delineation of six species of the primitive algal genus *Glaucocystis* based on in situ ultrastructural characteristics. *Sci. Rep.,* **6**:29209.

## Sar

Burki, F., Shalchian‐Tabrizi, K., Minge, M., Skjaeveland, A., Nikolaev, S. I., Jakobsen, K. S., & Pawlowski, J. 2007. Phylogenomics reshuffles the eukaryotic supergroups. *PLoS ONE*,** 2**(8), e790. https://doi.org/10.1371/journal.pone.0000790

Hackett, J. D., Yoon, H. S., Li, S., Reyes‐Prieto, A., Rümmele, S. E., & Bhattacharya, D. 2007. Phylogenomic analysis supports the monophyly of cryptophytes and haptophytes and the association of rhizaria with chromalveolates. *Molec. Biol. Evol*., **24**(8), 1702–1713. https://doi.org/10.1093/molbev/msm089

Rodriguez‐Ezpeleta, N., Brinkmann, H., Burger, G., Roger, A. J., Gray, M. W., Philippe, H., & Lang, B. F. 2007. Toward resolving the eukaryotic tree: the phylogenetic positions of jakobids and cercozoans. *Current Biol.,* **17**(16): 1420–1425. https://doi.org/10.1016/j.cub.2007.07.036

### Stramenopiles

Aleoshin, V. V., A. P. Mylnikov, G. S. Mirzaeva, K. V. Mikhailov, and S. A. Karpov. 2016. Heterokont Predator *Develorapax marinus* gen. et sp. nov. ‐ A Model of the Ochrophyte Ancestor. *Front. Microbiol*., **7**:1194.

Cavalier‐Smith, T. 2018. Kingdom Chromista and its eight phyla: a new synthesis emphasising periplastid protein targeting, cytoskeletal and periplastid evolution, and ancient divergences. *Protoplasma,* **255**:297‐357.

Cavalier‐Smith, T., and J. M. Scoble. 2013. Phylogeny of Heterokonta: *Incisomonas marina*, a uniciliate gliding opalozoan related to *Solenicola* (Nanomonadea), and evidence that Actinophryida evolved from raphidophytes. *Eur. J. Protistol*., **49**:328‐353.

Chang, F. H., J. Sutherland, and J. Bradford‐Grieve. 2017. Taxonomic revision of Dictyochales (Dictyochophyceae) based on morphological, ultrastructural, biochemical and molecular data. *Phycol. Res*. **65**:235‐347.

Derelle, R., P. Lopez‐Garcia, H. Timpano, and D. Moreira. 2016. A Phylogenomic Framework to Study the Diversity and Evolution of Stramenopiles (=Heterokonts). *Mol. Biol. Evol*., **33**:2890‐2898.

Lin, Y. C., T. Campbell, C. C. Chung, G. C. Gong, K. P. Chiang, and A. Z. Worden. 2012. Distribution patterns and phylogeny of marine stramenopiles in the north pacific ocean. *Appl. Environ. Microbiol*. **78**:3387‐3399.

Massana, R., J. del Campo, M. E. Sieracki, S. Audic, and R. Logares. 2014. Exploring the uncultured microeukaryote majority in the oceans: reevaluation of ribogroups within stramenopiles. *ISME J* **., 8**:854‐866.

McCarthy, C. G. P., and D. A. Fitzpatrick. 2017. Phylogenomic Reconstruction of the Oomycete Phylogeny Derived from 37 Genomes. *mSphere,* **2**.

Shiratori, T., T. Nakayama, and K. Ishida. 2015. A New Deep‐branching Stramenopile, *Platysulcus tardus* gen. nov., sp. nov. *Protist,* **166**:337‐348.

Yang, E. C., G. H. Boo, H. J. Kim, S. M. Cho, S. M. Boo, R. A. Andersen, and H. S. Yoon. 2012. Supermatrix data highlight the phylogenetic relationships of photosynthetic stramenopiles. *Protist*,** 163**:217‐231.

Yubuki, N., T. Panek, A. Yabuki, I. Cepicka, K. Takishita, Y. Inagaki, and B. S. Leander. 2015. Morphological Identities of Two Different Marine Stramenopile Environmental Sequence Clades: *Bicosoeca kenaiensis* (Hilliard, 1971) and Cantina marsupialis (Larsen and Patterson, 1990) gen. nov., comb. nov. *J. Eukaryot. Microbiol.,* **62**:532‐542.

### Diatomea

Ashworth, M. P., Nakov, T. & Theriot, E. C. 2013. Revisiting Ross and Sims (1971): toward a molecular phylogeny of the Biddulphiaceae and Eupodiscaceae (Bacillariophyceae). *J*. *Phycol*. **49**:1207–1222.

Ichinomiya, M., Yoshikawa, S., Kamiya, M., Ohki, K., Takaichi, S. & Kuwata, A. 2011. Isolation and characterization of Parmales (Heterokonta/Heterokontophyta/Stramenopiles) from the Oyahio region, western North Pacific. *J. Phycol*. **47**:144–151.

Ichinomiya, M., Lopes dos Santos, A., Gourvil, P., Yoshikawa, S., Kamiya, M., Ohki, K., Audic, S., Vargas, C. de, Noël, M.‐H., Vaulot, D. & Kuwata, A. 2016. Diversity and oceanic distribution of the Parmales (Bolidophyceae), a picoplanktonic group closely related to diatoms. *ISME J*. **10**:2419–2434.

Mann, D.G., Crawford, R.M. & Round, F.E. 2017. Bacillariophyta. In: *Handbook of the Protists* (Archibald, J.M., Simpson, A.G.B. & Slamovits, C.H., eds), 62 pp. Springer, Cham. https://doi.org/10.1007/978-3-319-32669-6_29-1.

Medlin, L. K. 2016. Opinion: can coalescent models explain deep divergences in the diatoms and argue for the acceptance of paraphyletic taxa at all taxonomic hierarchies? *Nova Hedwigia* **102**:107–128.

Medlin, L. K. & Kaczmarska, I. 2004. Evolution of the diatoms: V. Morphological and cytological support for the major clades and a taxonomic revision. *Phycologia* **43**:245–270.

Nakov, T., Beaulieu, J. M. & Alverson, A. J. 2018. Accelerated diversification is related to life history and locomotion in hyperdiverse lineage of microbial eukaryotes (diatoms, Bacillariophta). *New Phytol.,* https://doi.org/10.1111/nph.15137

Parks, M. B., Wickett, N. J. & Alverson, A. J. 2017. Signal, uncertainty, and conflict in phylogenomic data for a diverse lineage of microbial eukaryotes (diatoms, Bacillariophyta). *Mol*. *Biol. Evol*., **35**:80–93.

Theriot, E. C., Ashworth, M. P., Nakov, T., Ruck, E. C. & Jansen, R. K. 2015. Dissecting signal and noise in diatom chloroplast protein encoding genes with phylogenetic information profiling. *Mol. Phylogenet. Evol*. **89**:28–36.

Round, F. E., Crawford, R. M. & Mann, D. G. 1990. The diatoms. Biology and morphology of the genera. Cambridge: Cambridge University Press. 747 pp.

## ALVEOLATA

Cumbo, V. R., Baird, A. H., Moore, R. B., Negri, A. P., Neilan, B. A., Salih, A., et al. 2013. *Chromera velia* is endosymbiotic in larvae of the reef corals *Acropora digitifera* and *A. tenuis*. *Protist* **164:** 237–244.

Freeman, M. A., Fuss, J., Kristmundsson, A., Bjorbaekmo, M. F. M., Mangot, J. F., del Campo, J., Keeling, P. J., Shalchian‐Tabrizi, K. & Bass, D. 2017. X‐cells are globally distributed, genetically divergent fish parasites related to perkinsids and dinoflagellates. *Curr. Biol.,* **27:** 1645‐1651. https://doi.org/10.1016/j.cub.2017.04.045

Gile, G. H. & Slamovits, C. H. 2014 Transcriptomic analysis reveals evidence for a cryptic plastid in the colpodellid *Voromonas pontica*, a close relative of chromerids and apicomplexan parasites. *PLoS ONE*. **9:** e96258. https://doi.org/10.1371/journal.pone.0096258

Mathur, V., del Campo, J., Kolisko, M. & Keeling, P. J. 2018. Global diversity and distribution of close relatives of apicomplexan parasites. *Environ. Microbiol*. **20**: 2824–2833. https://doi.org/10.1111/1462-2920.14134

Oborník, M., Kručinská, J. & Esson, H. 2016. Life cycles of chromerids resemble those of colpodellids and apicomplexan parasites. *Perspect. Phycol*. **3:** 21–27. https://doi.org/10.1127/pip/2016/0038

Oborník M., Lukeš J. 2015. The organellar genomes of *Chromera* and *Vitrella*, the phototrophic relatives of apicomplexan parasites. *Annu. Rev. Microbiol*. **69:** 129‐144. https://doi.org/10.1146/annurev-micro-091014-104449

Okamoto, N., & Keeling, P. J. 2014. A comparative overview of the flagellar apparatus of dinoflagellate, perkinsids and colpodellids. *Microorganisms*,** 2:** 73–91. https://doi.org/10.3390/microorganisms2010073

Reñé, A., Alacid, E., Ferrera, I., & Garcés, E. 2017. Evolutionary trends of Perkinsozoa (Alveolata) characters based on observations of two new genera of parasitoids of dinoflagellates, *Dinovorax* gen. nov. and *Snorkelia* gen. nov. *Front. Microbiol*. **8**. 1594. https://doi.org/10.3389/fmicb.2017.01594.

Tikhonenkov, D. V., Janouškovec, J., Mylnikov, A. P., Mikhailov, K. V., Simdyanov,T. G., Aleoshin, V. V. & Keeling, P. J. 2014. Description of *Colponema vietnamica*sp. n. and *Acavomonas peruviana*n. gen. n. sp., two new Alveolate phyla (Colponemidia nom. nov. and Acavomonidia nom. nov.) and their contributions to reconstructing the ancestral state of alveolates and eukaryotes. *PLoS ONE,* **9:** e95467. https://doi.org/10.1371/journal.pone.0095467

Woo, Y.H., Ansari, H., Otto, T.D., Klinger, C.M., Kolisko, M., Michalek, J., et al. 2015. Chromerid genomes reveal the evolutionary path from photosynthetic algae to obligate intracellular parasites. *eLife*. **4:**1–41.

Yuan, C. L., Keeling, P. J., Krause, P. J., Horak, A., Bent, S., Rollend, L. & Hua, X. G. 2012. *Colpodella* spp.–like Parasite infection in woman, China. *Emerg. Infect. Dis*. **18:** 125‐127. https://doi.org/10.3201/eid1801.110716.

### Apicomplexa

Arisue, N. & Hashimoto, T. 2015. Phylogeny and evolution of apicoplasts and apicomplexan parasites. *Parasitol. Int*. **64:** 254–259.

Cavalier‐Smith, T. 2018. Kingdom Chromista and its eight phyla: A new synthesis emphasising periplastid protein targeting, cytoskeletal and periplastid evolution, and ancient divergences. *Protoplasma*. **1:** 297‐357. https://doi.org/10.1007/s00709-017-1147-3.

Cavalier‐Smith, T. 2014. Gregarine site‐heterogeneous 18S rDNA trees, revision of gregarine higher classification, and the evolutionary diversification of Sporozoa. *Europ. J. Protistol*. **50:** 472‐495. https://doi.org/10.1016/j.ejop.2014.07.002.

Desportes, I. & Schrével, J. 2013. Treatise on Zoology ‐ Anatomy, Taxonomy, Biology. The Gregarines, Vol 1 & 2. Brill, Leiden.

Flegontov, P. Michálek, J., Tomčala, A., Janouškovec, J., Jirků, M., Lai, D. H., Hajdůšková, E., Otto, T. D., Keeling, P. J., Pain, A., Oborník, M. & Lukeš, J. 2015. Divergent mitochondrial respiratory chains in phototrophic relatives of apicomplexan parasites. *Mol. Biol. Evol*. **32:** 1115‐1131. https://doi.org/10.1093/molbev/msv021

Ghazy, A. A., Abdel‐Shafy, S. & Shaapan, R. M. 2015. Cryptosporidiosis in animals and man: 1. taxonomic classification, life cycle, epidemiology and zoonotic importance. *Asian J. Epidemiol*. **8:** 48‐63. https//doi.org/10.3923/aje.2015.48.63

Heintzelman, M. B. 2015. Gliding motility in apicomplexan parasites. *Semin. Cell. Dev. Biol*. **46:**135–142. https://doi.org/10.1016/j.semcdb.2015.09.020

Iritani, D., Wakeman, K. & Leander, B.S. 2018. Molecular phylogenetic positions of two new marine gregarines (Apicomplexa) from the intestines of *Lumbrineris inflata* (Polychaeta) show patterns of co‐ evolution: *Paralecudina anankea* nov. sp. and *Lecudina caspera* nov. sp. *J. Eukaryot. Microbiol*. **65:**211‐219. https://doi.org/10.1111/jeu.12462

Janouškovec, J., Tikhonenkov, D. V., Burki, F., Howe, A. T., Kolisko, M., Mylnikov, A. P., et al. 2015. Factors mediating plastid dependency and the origins of parasitism in apicomplexans and their close relatives. *Proc. Natl. Acad. Sci. USA* **112:** 10200–10207. https://doi.org/10.1111/1462-2920.14134

Janouškovec, J. Horák, A., Barrot, K. L., Rohwer, F. L. & Keeling, P. J. 2012. Global analysis of plastid diversity reveals new apicomplexan‐related lineages associated with coral reefs. *Curr. Biol*. **22:** R518–9. https://doi.org/10.1016/j.cub.2012.04.047

Karadjian, G., Chavatte, J.‐M., & Landau, I. 2015. Systematic revision of the adeleid haemogregarines, with creation of *Bartazoon* n. g., reassignment of *Hepatozoon argantis* Garnham, 1954 to *Hemolivia*, and molecular data on *Hemolivia stellata*. *Parasite*. **22:** 31. https://doi.org/10.1051/parasite/2015031

Megía‐Palma, R., Martínez, J., Nasri, I., Cuervo, J. J., Martín, J., Acevedo, I., Belliure, J., Ortega, J., García‐Roa, R., Selmi, S. & Merino, S. 2016. Phylogenetic relationships of *Isospora*,* Lankesterella*, and *Caryospora* species (Apicomplexa: Eimeriidae) infecting lizards. *Org. Divers. Evol*. **16:** 275‐288. https://doi.org/10.1007/s13127-015-0253-3

Muñoz‐Gómez S. A. & Slamovits C. H. 2018. Chapter Three ‐ Plastid Genomes in the Myzozoa. In: Chaw, S.‐M. & Jansen, R. K. *Adv. Bot. Res*. **85:** 55‐94. https://doi.org/10.1016/bs.abr.2017.11.015.

Ogedengbe, M. E., El‐Sherry, S., Ogedengbe, J. D., Chapman, H. D. & Barta, J. R. 2018. Phylogenies based on combined mitochondrial and nuclear sequences conflict with morphologically defined genera in the eimeriid coccidia (Apicomplexa). *Int. J. Parasitol*. **48:** 59‐69. https://doi.org/10.1016/j.ijpara.2017.07.008.

Ogedengbe, J. D., Ogedengbe, M. E., Hafeez, M. A. & Barta, J. R. 2015. Molecular phylogenetics of eimeriid coccidia (Eimeriidae, Eimeriorina, Apicomplexa, Alveolata): A preliminary multi‐gene and multi‐ genome approach. *Parasitol. Res*. **114:** 4149‐4160. https://doi.org/10.1007/s00436-015-4646-1

Rueckert, S. & Horák, A. 2017. Archigregarines of the English Channel revisited: New molecular data on *Selenidium* species including early described and new species and the uncertainties of phylogenetic relationships. *PLoS ONE* **12:** e0187430. https://doi.org/10.1371/journal.pone.0187430

Rueckert, S., Wakeman, K.C. & Leander, B.S. 2013. Discovery of a diverse clade of gregarine apicomplexans (Apicomplexa: Eugregarinorida) from Pacific eunicid and onuphid polychaetes, including descriptions of *Paralecudina* n. gen., *Trichotokara japonica* n. sp., and *T. eunicae* n. sp. *J. Eukaryot. Microbiol*. **60:**121‐136. https://doi.org/10.1111/jeu.12015

Ryan, U., Fayer, R. & Xiao, L. (2014) *Cryptosporidium* species in humans and animals: Current understanding and research needs. *Parasitol.,* **141:** 1667‐1685. https://doi.org/10.1017/s0031182014001085

Schrével, J., Valigurová, A., Prensier, G., Chambouvet, A., Florent, I. & Guillou, L. 2016. Ultrastructure of *Selenidium pendula*, the type species of archigregarines, and phylogenetic relations to other marine Apicomplexa. *Protist*,** 167:** 339‐368. https://doi.org/10.1016/j.protis.2016.06.001.

Seeber, F. & Steinfelder, S. 2016. Recent advances in understanding apicomplexan parasites. *F1000Research*. **5:**1369. https://doi.org/10.12688/f1000research.7924.1

Simdyanov, T. G., Paskerova, G. G., Valigurová, A., Diakin, A., Kováčiková, M., Schrével, J., Guillou, L., Dobrovolskij, A. A. & Aleoshin, V. V. (2018) First ultrastructural and molecular phylogenetic evidence from the blastogregarines, an early branching lineage of plesiomorphic Apicomplexa. *Protist,* **169**: 697–726. https://doi.org/10.1016/j.protis.2018.04.006.

Votýpka, J., Modrý, D., Oborník, M., Šlapeta, J. & Lukeš, J. 2017. Apicomplexa. In: Archibald, J. M.,Simpson, A. G. B., and Slamovits, C., eds. Handbook of the Protists (Second Edition of the Handbook of Protoctista by Margulis et al.) Springer Reference Works (e‐book) https://doi.org/10.1007/978-3-319-32669-6_12-1.

Wakeman, K.C., Heintzelman, M.B. & Leander, B.S. 2014. Comparative ultrastructure and molecular phylogeny of *Selenidium melongena* n. sp. and *S. terebellae* Ray 1930 demonstrate niche partitioning in marine gregarine parasites (apicomplexa). *Protist*. **165:** 493‐511. https://doi.org/10.1016/j.protis.2014.05.007

Wakeman, K., Reimer, J.D., Jenke‐Kodama, H. & Leander, B.S. 2014. Molecular phylogeny and ultrastructure of *Caliculium glossobalani* n. gen. et sp. (Apicomplexa) from a Pacific *Glossobalanus minutus* (Hemichordata) confounds the relationships between marine and terrestrial gregarines. *J. Eukaryot. Microbiol*. **61**:343‐353.

### Ciliophora

Antipa, G.A., Dolan, J.R., Lynn, D.H., Obolkina, L.A. & Strüder‐Kypke, M.C. 2016. Molecular phylogeny and evolutionary relationships between the ciliate genera *Peniculistoma* and *Mytilophilus* (Peniculistomatidae, Pleuronematida). *J. Eukaryot. Microbiol.,* **63**:642‐650.

Bourland, W., Rotterová, J. & Čepička, I. 2017. Morphologic and molecular characterization of seven species of the remarkably diverse and widely distributed metopid genus *Urostomides* Jankowski, 1964 (Armophorea, Ciliophora). *Eur. J. Protistol.,* **61**:194‐232.

Bourland, W.A., Hampikian, G. & Vďačný, P. 2012. Morphology and phylogeny of a new woodruffiid ciliate, *Etoschophrya inornata* sp. n. (Ciliophora, Colpodea, Platyophryida), with an account on evolution of platyophryids. *Zool. Scr.,* **41**:400‐416.

Chen, X., Ma, H.‐G., Al‐Rasheidm, K. A. S. & Miao, M. 2015. Molecular data suggests the ciliate *Mesodinium* (Protista: Ciliophora) might represent an undescribed taxon at class level. *Zool. Syst*., **40**:31‐40.

Dunthorn, M., Otto, J., Berger, S. A., Stamatakis, A., Mahé, F., Romac, S., de Vargas, C., Audic, S., BioMarKs Consortium, Stock, A., Kauff, F. & Stoeck, T. 2014. Placing environmental next‐generation sequencing amplicons from microbial eukaryotes into a phylogenetic context. *Mol. Biol. Evol*., **31**:993‐1009.

Fan, X., Pan, H., Li, L., Jiang, J., Al‐Rasheid, K.A.S. & Gu, F. 2014. Phylogeny of the poorly known ciliates, Microthoracida, a systematically confused taxon (Ciliophora), with morphological reports of three species. *J. Eukaryot. Microbiol.,* **61**:227‐237.

Feng, J.‐M., Jiang, C.‐Q., Warren, A., Tian, M., Cheng, J., Liu, G.‐L., Xiong, J. & Miao, W. 2015. Phylogenomic analyses reveal subclass Scuticociliatia as the sister group of subclass Hymenostomatia within class Oligohymenophorea. *Mol. Phylogenet. Evol.,* **90**:104‐111.

Fernandes, N.M., Vizzoni, V.F., Borges, B.d.N., Soares, C.A.G., da Silva‐Neto, I.D. & Paiva, T.d.S. 2018. Molecular phylogeny and comparative morphology indicate that odontostomatids (Alveolata, Ciliophora) form a distinct class‐level taxon related to Armophorea. *Mol. Phylogenet. Evol.,* **126**:382‐389.

Foissner, W., Stoeck, T., Agatha, S. & Dunthorn, M. 2011. Intraclass evolution and classification of the Colpodea (Ciliophora). *J. Eukaryot. Microbiol.,* **58**:397‐415.

Foissner, W., Bourland, W.A., Wolf, K.W., Stoeck, T. & Dunthorn, M. 2014. New SSU‐rDNA sequences for eleven colpodeans (Ciliophora, Colpodea) and description of *Apocyrtolophosis* nov. gen. *Eur. J. Protistol.,* **50**:40‐46.

Gao, F., Katz, L.A. & Song, W. 2012. Insights into the phylogenetic and taxonomy of philasterid ciliates (Protozoa, Ciliophora, Scuticociliatia) based on analyses of multiple molecular markers. *Mol. Phylogenet. Evol.,* **64**:308‐317.

Gao, F., Katz, L.A. & Song, W. 2013. Multigene‐based analyses on evolutionary phylogeny of two controversial ciliate orders: Pleuronematida and Loxocephalida (Protista, Ciliophora, Oligohymenophorea). *Mol. Phylogenet. Evol.,* **68**:55‐63.

Gao, F., Warren, A., Zhang, Q., Gong, J., Miao, M., Sun, P., Xu, D., Huang, J., Yi, Z. & Song, W. 2016. The all‐data‐based evolutionary hypothesis of ciliated protists with a revised classification of the Phylum Ciliophora (Eukaryota, Alveolata). *Sci. Rep.,* **6**:24874.

Gentekaki, E., Kolisko, M., Gong, Y. & Lynn, D. 2017. Phylogenomics solves a long‐standing evolutionary puzzle in the ciliate world: the subclass Peritrichia is monophyletic. *Mol. Phylogenet. Evol.,* **106**:1‐5.

Gentekaki, E., Kolisko, M., Boscaro, V., Bright, K. J., Dini, F., Di Giuseppe, G., Gong, Y., Miceli, C., Modeo, L., Molestina, R.E., Petroni, G., Pucciarelli, S., Roger, A.J., Strom, S.L. & Lynn, D.H. 2014. Large‐ scale phylogenomic analysis reveals the phylogenetic position of the problematic taxon *Protocruzia* and unravels the deep phylogenetic affinities of the ciliate lineages. *Mol. Phylogenet. Evol.,* **78**:36‐42.

Gong, J., Stoeck, T., Yi, Z., Miao, M., Zhang, Q., Roberts, D. M., Warren, A. & Song, W. 2009. Small subunit rRNA phylogenies show that the class Nassophorea is not monophyletic (Phylum Ciliophora). *J. Eukaryot. Microbiol.,* **56**:339‐347.

Johnson, M. D., Tengs, T., Oldach, D. W., Delwiche, C. F. & Stoecker, D. K. 2004. Highly divergent SSU rRNA genes found in the marine ciliates *Myrionecta rubra* and *Mesodinium pulex*. *Protist*,** 155**:347‐359.

Kittelmann, S., Devente, S.R., Kirk, M.R., Seedorf, H., Dehority, B.A. & Janssen, P.H. 2015. Phylogeny of intestinal ciliates, including *Charonina ventriculi*, and comparison of microscopy and 18S rRNA gene pyrosequencing for rumen ciliate community structure analysis. *Appl. Environ. Microbiol.,* **81**:2433‐2444.

Liu, A., Yi, Z., Lin, X., Hu, X., Al‐Farraj, S. A. & Al‐Rasheid, K. A. S. 2015. Molecular phylogenetic lineage of *Plagiopogon* and *Askenasia* (Protozoa, Ciliophora) revealed by their gene sequences. *J. Ocean Univ. China,* **14**:724‐730.

Lynn, D. H. 2008. The ciliated protozoa: characterization, classification, and guide to the literature, 3rd edition. Springer, Dordrecht.

Lynn, D.H., Kolisko, M. & Bourland, W. 2018. Phylogenomic analysis of *Nassula variabilis* n. sp., *Furgasonia blochmanni*, and *Pseudomicrothorax dubius* confirms a nassophorean clade. *Protist,* **169**:180‐189.

Orsi, W., Edgcomb, V., Faria, J., Foissner, W., Fowle, W. H., Hohman, T., Suarez, P., Taylor, C., Taylor, G. T., Vd'ačný, P. & Epstein, S. 2012. Class Cariacotrichea, a novel ciliate taxon from the anoxic Cariaco Basin, Venezuela. *Int. J. Syst. Evol. Microbiol.,* **62**:1425‐1433.

Pan, H., Stoeck, T. 2017. Redescription of the halophile ciliate, *Blepharisma halophilum* Ruinen, 1938 (Ciliophora, Heterotrichea, Heterotrichida) shows that the genus *Blepharisma* is non‐monophyletic. *Eur. J. Protistol.,* **61**:20‐28.

Santoferrara, L. F., Alder, V.V. & McManus, G.B. 2017. Phylogeny, classification and diversity of Choreotrichia and Oligotrichia (Ciliophora, Spirotrichea). *Mol. Phylogenet. Evol.,* **112**:12‐22.

Sauvadet, A.‐L., Lynn, D.H., Roussel, E. G., Le Panse, S., Bigeard, E., Schrével, J. &, Guillou, L. 2017. Redescription and phylogenetic analyses of *Durchoniella* spp. (Ciliophora, Astomatida) associated with the polychaete *Cirriformia tentaculata* (Montagu, 1808). *Eur. J. Protistol.,* **61**:265‐277.

Shin, M. K., Hwang, U. W., Kim, W., Wright, A.‐D. G., Krawczyk, C. & Lynn, D. H. 2000. Phylogenetic position of the ciliates *Phacodinium* (Order Phacodiniida) and *Protocruzia* (Subclass Protocruziidia) and systematics of the spirotrich ciliates examined by small subunit ribosomal RNA gene sequences. *Europ. J. Protistol*., **36**:293‐302.

Vďačný, P. & Rataj, M. 2017. Evaluation of systematic position of helicoprorodontids and chaeneids (Ciliophora, Litostomatea): an attempt to break long branches in 18S rRNA gene phylogenies. *J. Eukaryot. Microbiol.,* **64**:608‐621.

Wang, P., Wang, Y., Wang, C., Zhang, T., Al‐Farraj, S.A. & Gao, F. 2017. Further consideration on the phylogeny of the Ciliophora: analyses using both mitochondrial and nuclear data with focus on the extremely confused class Phyllopharyngea. *Mol. Phylogenet. Evol.,* **112**:96‐106.

Xu, Y., Shao, C., Miao, M. & Song, W. 2013. Redescription of *Parasonderia vestita* (Kahl, 1928) comb. nov. (Ciliophora, Plagiopylida), with notes on its phylogeny based on SSU rRNA gene. *Eur. J. Protistol.,* **49**:106‐113.

Yan, Y., Xu, Y., Al‐Farraj, S.A., Al‐Rasheid, K.A.S. & Song, W. 2016. Morphology and phylogeny of three trachelocercids (Protozoa, Ciliophora, Karyorelictea), with description of two new species and insight into the evolution of the family Trachelocercidae. *Zool. J. Linn. Soc.‐Lond.,* **177**:306‐319.

Zhan, Z., Xu, K. & Dunthorn, M. 2013. Evaluating molecular support for and against the monophyly of the Peritrichia and phylogenetic relationships within the Mobilida (Ciliophora, Oligohymenophorea) Zool. Scripta, **42**:213‐226.

Zhan, Z., Xu, K., Warren, A. & Gong, Y. 2009. Reconsideration of phylogenetic elationships of the subclass Peritrichia (Ciliophora, Oligohymenophorea) based on small subunit ribosomal RNA gene sequences, with the establishment of a new subclass Mobilia Kahl, 1933. *J. Eukaryot. Microbiol*., **56**:552‐558.

Zhang, Q., Fan, X., Clamp, J.C., Al‐Rasheid, K.A.S. & Song, W. 2010. Description of *Paratetrahymena parawassi* n. sp. using morphological and molecular evidence and a phylogenetic analysis of *Paratetrahymena* and other taxonomically ambiguous genera in the order Loxocephalida (Ciliophora, Oligohymenophorea). *J. Eukaryot. Microbiol.,* **57**:483‐493.

Zhang, Q., Miao, M., Strüder‐Kypke, M.C., Al‐Rasheid, K.A.S., Al‐Farraj, S.A. & Song, W. 2011. Molecular evolution of *Cinetochilum* and *Sathrophilus* (Protozoa, Ciliophora, Oligohymenophorea), two genera of ciliates with morphological affinities to scuticociliates. *Zool. Scr.,* **40**:317‐325.

Zhang, Q., Yi, Z., Fan, X., Warren, A., Gong, J. & Song, W. 2014. Further insights into the phylogeny of two ciliate classes Nassophorea and Prostomatea (Protista, Ciliophora). *Mol. Phylogenet. Evol.,* **70**:162‐170.

Zhang, X., Ji, D., Zhang, Q. & Li, C. 2015. Description and phylogeny of a new prostomatid, *Metacystis similis* nov. spec. (Protista, Ciliophora) from the East China Sea. *Zootaxa,* **4033**:584‐592.

Zhao, X., Miao, M., Chen, X., Ma, H. & Al‐Rasheid, K.A.S. 2014. A phylogenetic reconsideration of suctorian ciliates (Protista, Ciliophora, Phyllopharyngea) based on small subunit rRNA gene sequences. *Zool. Scr.,* **43**:206‐216.

### Dinoflagellata

Bachvaroff, T. R., Gornik, S. G., Concepcion, G. T., Waller, R. F., Mendez, G. S., Lippmeier, J. C. & Delwiche, C. F. 2014. Dinoflagellate phylogeny revisited: using ribosomal proteins to resolve deep branching dinoflagellate clades. *Mol. Phylogen. Evol*., **70**: 314‐322.

Boutrup, P. V., Moestrup, Ø., Tillmann, U. & Daugbjerg, N. 2016. *Katodinium glaucum* (Dinophyceae) revisited: proposal of new genus, family and order based on ultrastructure and phylogeny. *Phycologia*,** 55**(2): 147‐164.

Hoppenrath, M. 2017. Dinoflagellate taxonomy ‐ a review and proposal of a revised classification. *Mar. Biodiv*., **47**: 381‐403.

Janouskovec, J., Gavelis, G. S., Burki, F., Dinh, D., Bachvaroff, T. R., Gornik, S. G., Bright, K. J., Imanian, B., Strom, S. L., Delwiche, C. F., Waller, R. F., Fensome, R. A., Leander, B. S., Rohwer, F. L. & Saldarriaga, J.F. 2017. Major transitions in dinoflagellate evolution unveiled by phylotranscriptomics. *PNAS, USA*,** 114**: E171‐E180.

Orr, R. J. S., Murray, S. A., Stüken, A., Rhodes, L. & Jakobsen, K. S. 2012. When naked became armored: an eight‐gene phylogeny reveals monophyletic origin of theca in dinoflagellates. *PLoS ONE*,** 7**(11): e50004.

Takano, Y., Yamaguchi, H., Inouye, I., Moestrup, Ø. & Horiguchi, T. 2014. Phylogeny of five species of *Nusuttodinium* gen. nov. (Dinophyceae), a genus of unarmoured kleptoplastidic dinoflagellates. *Protist*,** 165**(6): 759‐778.

## RHIZARIA

Bass, D., Tikhonenkov, D.V., Foster, R., Dyal, P., Janouskovec, J., Keeling, P.J., Gardner, M., Neuhauser, S., Hartikainen, H., Mylnikov, A.P., & Berney, C. 2018. Rhizarian ‘Novel Clade 10’ revealed as abundant and diverse planktonic and terrestrial flagellates, including *Aquavolon* n. gen. *J. Eukaryot. Microbiol*. **65**: 828–842. https://doi.org/10.1111/jeu.12524.

Cavalier‐Smith, T., Chao, E.E., & Lewis, R. 2018. Multigene phylogeny and cell evolution of chromist infrakingdom Rhizaria: contrasting cell organisation of sister phyla Cercozoa and Retaria. *Protoplasma*,** 255**: 1517‐1574. https://doi.org/10.1007/s00709-018-1241-1.

Krabberød, A.K., Orr, R.J., Bråte, J., Kristensen, T., Bjørklund, K.R., & Shalchian‐Tabrizi, K. 2017. Single cell transcriptomics, mega‐phylogeny, and the genetic basis of morphological innovations in Rhizaria. *Mol. Biol. Evol*. **34**:1557‐1573. https://doi.org/10.1093/molbev/msx075.

Sierra, R., Cañas‐Duarte, S.J., Burki, F., Schwelm, A., Fogelqvist, J., Dixelius, C., González‐García, L.N., Gile, G.H., Slamovits, C.H., Klopp, C., Restrepo, S., Arzul, I., & Pawlowski, J. 2016. Evolutionary origins of Rhizarian parasites. *Mol. Biol. Evol*. **33**:980‐983. https://doi.org/10.1093/molbev/msv340.

### Cercozoa

Bass, D., Silberman, J.D., Brown, M.W., Pearce, R.A., Tice, A.T., Jousset, A., Geisen, S., & Hartikainen, H. 2016. Coprophilic amoebae and flagellates, including *Guttulinopsis*,* Rosculus* and *Helkesimastix*, characterise a divergent and diverse rhizarian radiation and contribute to a large diversity of faecal‐associated protists. *Env. Microbiol*. **18**:1604‐1619. https://doi.org/10.1111/1462-2920.13235.

Bass, D., Yabuki, A., Santini, S., Romac, S., & Berney, C. 2012. *Reticulamoeba* is a long‐branched Granofilosean (Cercozoa) that is missing from sequence databases. *PLoS One* **7**:e49090. https://doi.org/10.1371/journal.pone.0049090.

Bugge Harder, C., Rønn, R., Brejnrod, A., Bass, D., Al‐Soud, W.A. & Ekelund, F. 2016. Local diversity of heathland Cercozoa explored by in‐depth sequencing. *ISME J*. **10**: 2488‐97.

Cavalier‐Smith, T. & Oates, B. (2012). Ultrastructure of *Allapsa vibrans* and the Body Plan of Glissomonadida (Cercozoa). *Protist,* **163:** 165–187.

Cavalier‐Smith, T. & Karpov, S.A. 2012. *Paracercomonas* Kinetid Ultrastructure, Origins of the Body Plan of Cercomonadida, and Cytoskeleton Evolution in Cercozoa. *Protist,* **163**, 47–75.

Chatelain, A.P., Meisterfeld, R., Roussel‐Delif, L., & Lara, E. 2013. Sphenoderiidae (fam. nov.), a new clade of euglyphid testate amoebae characterized by small, round scales surrounding the aperture. *Protist* **164**:782‐792. https://doi.org/10.1016/j.protis.2013.08.001.

Dumack, K., Baumann, C. & Bonkowski, M. 2016. A Bowl with Marbles: Revision of the Thecate Amoeba Genus *Lecythium* (Chlamydophryidae, Tectofilosida, Cercozoa, Rhizaria) Including a Description of Four New Species and an Identification Key. *Protist,* **167:** 440–459.

Dumack, K., Schuster, J., Bass, D., & Bonkowski, M. 2016. A novel lineage of ‘naked filose amoebae’; *Kraken carinae* gen. nov. sp. nov. (Cercozoa) with a remarkable locomotion by disassembly of its cell body. *Protist* **167**:268‐278. https://doi.org/10.1016/j.protis.2016.04.002.

Dumack, K., Flues, S., Hermanns, K. & Bonkowski, M. 2017. Rhogostomidae (Cercozoa) from soils, roots and plant leaves (*Arabidopsis thaliana*): Description of *Rhogostoma epiphylla* sp. nov. and *R. cylindrica* sp. nov. *Europ. J. Protistol., **60***, 76–86.

Dumack, K., Mausbach, P., Hegmann, M. & Bonkowski, M. 2017. Polyphyly in the Thecate Amoeba Genus *Lecythium* (Chlamydophryidae, Tectofilosida, Cercozoa), Redescription of its Type Species *L. hyalinum*, Description of *L. jennyae* sp. nov. and the Establishment of *Fisculla* gen. nov. and Fiscullidae fam. nov. *Protist,* **168:** 294–310.

Dumack, K., Mylnikov, A.P. & Bonkowski, M. 2017. Evolutionary Relationship of the Scale‐Bearing *Kraken* (incertae sedis, Monadofilosa, Cercozoa, Rhizaria): Combining Ultrastructure Data and a Two‐ Gene Phylogeny. *Protist,* **168:** 362–373.

Dumack, K., Öztoprak, H., Rüger, J. & Bonkowski, M. 2017. Shedding Light on the Polyphyletic Thecate Amoeba Genus *Plagiophrys*: Transition of Some of its Species to *Rhizaspis* (Tectofilosida, Thecofilosea, Cercozoa) and the Establishment of *Sacciforma* gen. nov. and Rhogostomidae fam. nov. (Cryomonadida, Thecofilosea, Cercozoa). *Protist,* **168**: 92–108.

Dumack, K., Bonkowski, M., Clauss, S. & Völcker, E. 2018. Phylogeny and redescription of the testate amoeba *Diaphoropodon archeri* (Chlamydophryidae, Thecofilosea, Cercozoa), De Saedeleer 1934, and annotations on the polyphyly of testate amoebae with agglutinated tests in the Cercozoa. *J. Eukaryot. Microbiol*. [Epub ahead of print] https://doi.org/10.1111/jeu.12474.

Dumack, K., Siemensma, F. & Bonkowski, M. 2018. Rediscovery of the testate amoeba genus *Penardeugenia* (Thaumatomonadida, Imbricatea). *Protist,* **169**:29‐42. https://doi.org/10.1016/j.protis.2017.12.002.

Hess, S. & Melkonian, M. 2013. The mystery of clade X: *Orciraptor* gen. nov. and *Viridiraptor* gen. nov. are highly specialised, algivorous amoeboflagellates (Glissomonadida, Cercozoa). *Protist,* **164**:706‐747. https://doi.org/10.1016/j.protis.2013.07.003.

Kosakyan, A., Gomaa, F., Lara, E. & Lahr, D.J.G. 2016. Current and future perspectives on the systematics, taxonomy and nomenclature of testate amoebae. *Eur. J. Protistol.,* **55**:105‐117. https://doi.org/10.1016/j.ejop.2016.02.001.

Lee, W.J. & Park, J.S. 2016. Placement of the unclassified *Cyranomonas australis* Lee 2002 within a novel clade of Cercozoa. *Eur. J. Protistol.,* **56**:60‐66. https://doi.org/10.1016/j.ejop.2016.06.004.

Ngo, C.N., Braithwaite, K.S., Bass, D., Young, A.J. & Croft, B.J. 2018. *Phytocercomonas venanatans*, a new species of Cercozoa associated with chlorotic streak of sugarcane. *Phytopathology,* **108**:479‐486. https://doi.org/10.1094/phyto-07-17-0237-r.

Nicholls, K.H. 2012. *Zoelucasa sablensis* n. gen. et n. sp. (Cercozoa, Incertae sedis), a new scale‐ covered flagellate from marine sandy shores. *Acta Protozoologica,* **51**(2): 113‐117.

Scoble, J.M. & Cavalier‐Smith, T. 2014 Scale evolution, sequence phylogeny, and taxonomy of thaumatomonad Cercozoa: 11 new species and new genera *Scutellomonas*,* Cowlomonas*,* Thaumatospina* and *Ovaloplaca*. *Europ. J. Protistol.,* **50**: 270–313.

Schuler, G.A., Tice, A.K., Pearce, R.A., Foreman, E., Stone, J., Gammill, S., Wilson, J.D., Reading, C., Silberman, J.D., Brown, M.W., Phylogeny and Classification of Novel Diversity in Sainouroidea (Cercozoa, Rhizaria) Sheds Light on a Highly Diverse and Divergent Clade. *Protist* https://doi.org/10.1016/j.protis.2018.08.002

Shiratori, T. & Ishida, K‐I. 2016. *Trachyrhizium urniformis* n. g., n. sp., a Novel Marine Filose Thecate Amoeba Related to a Cercozoan Environmental Clade (Novel Clade 4) *J. Euk. Microbiol.,* **63:**722–731.

Shiratori, T., Yabuki, A. & Ishida, K‐I. 2012. *Esquamula lacrimiformis* n. g., n. sp., a New Member of Thaumatomonads that Lacks Siliceous Scales. *J. Eukaryot. Microbiol*., **59**(6): 527–536.

Shiratori, T., Yokoyama, A. & Ishida, K‐I. 2014. Phylogeny, Ultrastructure, and Flagellar Apparatus of a New Marimonad Flagellate *Abollifer globosa* sp. nov. (Imbricatea, Cercozoa). *Protist,* **165:** 808–824.

Shiratori, T., Fujita, S., Shimizu, T., Nakayama, T. & Ishida, K. 2017. *Viridiuvalis adhaerens* gen. et sp. nov., a novel colony‐forming chlorarachniophyte. *J. Plant Res.,* **130**:999‐1012. https://doi.org/10.1007/s10265-017-0961-1.

Yabuki, A. & Ishida, K. 2018. An orphan protist *Quadricilia rotundata* finally finds its phylogenetic home in Cercozoa. *J. Eukaryot. Microbiol*. [Epub ahead of print] https://doi.org/10.1111/jeu.12502.

Yabuki, A. & Ishida, K‐I. 2011. *Mataza hastifera* n. g., n. sp.: A Possible New Lineage in the Thecofilosea (Cercozoa). *J. Eukaryot. Microbiol*., **58**(2): 94–102.

### Endomyxa

Berney, C., Romac, S., Mahé, F., Santini, S., Siano, R. & Bass, D. 2013. Vampires in the oceans: predatory cercozoan amoebae in marine habitats. *ISME J.,* **7**:2387‐2399. https://doi.org/10.1038/ismej.2013.116.

Gong, Y., Patterson, D.J., Li, Y., Hu, Z., Sommerfeld, M., Chen, Y. & Hu, Q. 2015 *Vernalophrys algivore* gen. nov., sp. nov. (Rhizaria: Cercozoa: Vampyrellida), a new algal predator isolated from outdoor mass culture of Scenedesmus dimorphus. *Appl. Environ. Microbiol.,* **81**:3900 –3913. https://doi.org/10.1128/aem.00160-15.

Hartikainen, H., Stentiford, G.D., Bateman, K.S., Berney, C., Feist, S.W., Longshaw, M., Okamura, B., Stone, D., Ward, G., Wood, C. & Bass, D. 2014. Mikrocytids are a broadly distributed and divergent radiation of parasites in aquatic invertebrates. *Curr. Biol.,* **24**:807‐812. https://doi.org/10.1016/j.cub.2014.02.033.

Hartikainen, H., Ashford, O.S., Berney, C., Okamura, B., Feist, S.W., Baker‐Austin, C., Stentiford, G.D. & Bass, D. 2014. Lineage‐specific molecular probing reveals novel diversity and ecological partitioning of haplosporidians. *ISME J.,* **8**: 177‐86.

Hess, S. 2017. Description of *Hyalodiscus flabellus* sp. nov. (Vampyrellida, Rhizaria) and identification of its bacterial endosymbiont, “Candidatus *Megaira polyxenophila*” (Rickettsiales, Alphaproteobacteria). *Protist*,** 168**:109‐133. https://doi.org/10.1016/j.protis.2016.11.003.

Hess, S., Sausen, N. & Melkonian, M. 2012. Shedding Light on Vampires: The Phylogeny of Vampyrellid Amoebae Revisited. *PLoS ONE*,** 7**(2): e31165. https://doi.org/10.1371/journal.pone.0031165

Neuhauser, S., Kirchmair, M., Bulman, S. & Bass, D. 2014. Cross‐kingdom host shifts of phytomyxid parasites. *BMC Evol. Biol.,* **14**:33. https://doi.org/10.1186/1471-2148-14-33.

Ward, G.M., Bennett, M., Bateman, K., Stentiford, G.D., Kerr, R., Feist, S.W., Williams, S.T., Berney, C. & Bass, D. 2016. A new phylogeny and environmental DNA insight into paramyxids: an increasingly important but enigmatic clade of protistan parasites of marine invertebrates. *Int. J. Parasitol.,* **46**:605‐619. https://doi.org/10.1016/j.ijpara.2016.04.010.

Ward, G.M., Neuhauser, S., Groben, R., Ciaghi, S., Berney, C., Romac, S. & Bass, D. 2018. Environmental sequencing fills the gap between parasitic haplosporidians and free‐living giant amoebae. *J. Eukaryot. Microbiol.,* **65**: 574–586. https://doi.org/10.1111/jeu.12501.

### Retaria

Biard, T., Pillet, L., Decelle, J., Poirier, C., Suzuki, N. & Not, F. 2015. Towards an integrative morpho‐molecular classification of the Collodaria (Polycystinea, Radiolaria). *Protist,* **166**:374‐388. https://doi.org/10.1016/j.protis.2015.05.002.

Decelle, J., Martin, P., Paborstava, K., Pond, D.W., Tarling, G., Mahé, F., de Vargas, C., Lampitt, R. & Not, F. 2013. Diversity, ecology and biogeochemistry of cyst‐forming Acantharia (Radiolaria) in the oceans. *PLoS ONE,* **8**:e53598. https://doi.org/10.1371/journal.pone.0053598.

Gooday, A.J., Holzmann, M., Caulle, C., Goineau, A., Kamenskaya, O., Weber, A.A.T. & Pawlowski, J. 2017. Giant protists (xenophyophores, Foraminifera) are exceptionally diverse in parts of the abyssal eastern Pacific licensed for polymetallic nodule exploration. *Biological Conservation,* **207**:106‐116. https://doi.org/10.1016/j.biocon.2017.01.006.

Holzmann, M. & Pawlowski, J. 2017. An updated classification of rotaliid foraminifera based on ribosomal DNA phylogeny. *Mar. Micropal.,* **132**:18‐34. https://doi.org/10.1016/j.marmicro.2017.04.002.

Pawlowski, J., Holzmann, M. & Tyszka, J. 2013. New supraordinal classification of Foraminifera: Molecules meet morphology. *Mar. Micropal.,* **100**:1‐10. https://doi.org/10.1016/j.marmicro.2013.04.002.

Siemensma, F., Apothéloz‐Perret‐Gentil, L., Holzmann, M., Clauss, S., Völcker, E. & Pawlowski, J. 2017. Taxonomic revision of freshwater foraminifera with the description of two new agglutinated species and genera. *Eur. J. Protistol.,* **60**:28‐44. https://doi.org/10.1016/j.ejop.2017.05.006.

### Cryptista

Burki, F., Okamoto, N., Pombert, J.‐F. & Keeling, P. J. 2012. The evolutionary history of haptophytes and cryptophytes: phylogenomic evidence for separate origins. *Proc. Roy. Soc. B: Biol. Sci.,* **279**(1736): 2246–2254. https://doi.org/10.1098/rspb.2011.2301

Yabuki, A., Kamikawa, R., Ishikawa, S. A., Kolisko, M., Kim, E., Tanabe, A. S., et al. 2014. *Palpitomonas bilix* represents a basal cryptist lineage: insight into the character evolution in Cryptista. *Sci. Reports*,** 4**: 4641. https://doi.org/10.1038/srep04641

Cavalier‐Smith, T., Chao, E. E. & Lewis, R. 2015. Multiple origins of Heliozoa from flagellate ancestors: New cryptist subphylum Corbihelia, superclass Corbistoma, and monophyly of Haptista, Cryptista, Hacrobia and Chromista. *Mol. Phylogenet. Evol*., **93:** 331–362. https://doi.org/10.1016/j.ympev.2015.07.004

### Haptista

Edvardsen, B., Egge, E.S. & Vaulot, D. 2016. Diversity and distribution of haptophytes revealed by environmental sequencing and metabarcoding – a review. *Perspectives in Phycology*,** 3**, Issue 2, p. 77–91.

Burki, F., Kaplan, M., Tikhonenkov, D. V., Zlatogursky, V., Minh, B. Q., Radaykina, L. V., et al. 2016. Untangling the early diversification of eukaryotes: a phylogenomic study of the evolutionary origins of Centrohelida, Haptophyta and Cryptista. *Proc. Royal Soc. B: Biol. Sci.,* **283**(1823). https://doi.org/10.1098/rspb.2015.2802 .

Cavalier‐Smith, T., Chao, E. E. & Lewis, R. 2015. Multiple origins of Heliozoa from flagellate ancestors: New cryptist subphylum Corbihelia, superclass Corbistoma, and monophyly of Haptista, Cryptista, Hacrobia and Chromista. *Mol. Phylogenet. Evol*., **93**, 331–362. https://doi.org/10.1016/j.ympev.2015.07.004.

Egge, E.S., Bittner, L., Andersen, T., Audic, S., de Vargas, C. & Edvardsen, B. 2013. 454 Pyrosequencing to Describe Microbial Eukaryotic Community Composition, Diversity and Relative Abundance: A Test for Marine Haptophytes. *PLOS ONE,* **8** e74371.

### Centroplasthelida

Bass, D., Chao, E. E., Nikolaev, S., Yabuki, A., Ishida, K. I., Berney, C., Pakzad, U., Wylezich, C. & Cavalier‐Smith, T. 2009. Phylogeny of novel naked filose and reticulose Cercozoa: Granofilosea cl. n. and Proteomyxidea revised. *Protist*,** 160**: 75‐109.

Cavalier‐Smith, T. & Chao, E. E. 2012. *Oxnerella micra* sp. n.(Oxnerellidae fam. n.), a tiny naked centrohelid, and the diversity and evolution of heliozoa. *Protist*,** 163**: 574‐601.

Cavalier‐Smith, T. & Scoble J. M. 2013. Phylogeny of Heterokonta: *Incisomonas marina,* a uniciliate gliding opalozoan related to *Solenicola* (Nanomonadea), and evidence that Actinophryida evolved from raphidophytes. *Europ. J. Protistol*., **49**: 328‐353.

Febvre‐Chevalier, C., & Febvre, J. 1984. Axonemal microtubule pattern of *Cienkowskya mereschkovskyi* and a revision of heliozoan taxonomy. *Origins of life*,** 13**: 315‐338.

Krabberød, A. K., Orr, R. J., Bråte, J., Kristensen, T., Bjørklund, K. R. & Shalchian‐Tabrizi, K. 2017. Single Cell Transcriptomics, Mega‐Phylogeny, and the Genetic Basis of Morphological Innovations in Rhizaria. *Mol. Biol. Evol*., **34**; 1557‐1573.

Mikrjukov, K. A. 1996. Revision of Genera and Species Composition of Lower Centroheliozoa. II. Family Raphidiophryidae n. fam. *Archiv. Protistenkd*., **147**: 205‐212.

Mikrjukov, K. A. 2000. Taxonomy and phylogeny of heliozoa. I. The order Desmothoracida Hertwig et Lesser, 1874. *Acta Protozool*., **39**; 81‐97.

Mikrjukov, K. A. 2002. Centrohelid heliozoans (Centroheliozoa). KMK Scientific, Moscow. Sekiguchi, H., Kawachi, M., Nakayama, T. & Inouye I. 2003. A taxonomic re‐evaluation of the Pedinellales (Dictyochophyceae), based on morphological, behavioural and molecular data. *Phycologia*,** 42**: 165–182.

Shishkin, Y., Drachko, D., Klimov, V. I. & Zlatogursky, V. V. 2018. *Yogsothoth knorrus* gen. n., sp. n. and *Y. carteri* sp. n. (Yogsothothidae fam. n., Haptista, Centroplasthelida), with notes on Evolution and Systematics of Centrohelids. *Protist*, https://doi.org/10.1016/j.protis.2018.06.003.

Zlatogursky, V. V. 2016. There and back again: parallel evolution of cell coverings in centrohelid heliozoans. *Protist*,** 167**; 51‐66.

Zlatogursky, V. V., Drachko, D., Klimov, V. I. & Shishkin, Y. 2018. On the phylogenetic position of the genus *Raphidocystis* (Haptista: Centroplasthelida) with notes on the dimorphism in centrohelid life cycle. *Europ. J. Protistol.,* **64**: 82‐90.

### Excavates

Bennett, M. S. & Triemer, R. E. 2012 A new method for obtaining nuclear gene sequences from field samples and taxonomic revisions of photosynthetic euglenoids *Lepocinclis* (*Euglena*) *helicoideus* and *Lepocinclis* (*Phacus*) *horridus* (Euglenophyta). *J. Phycol*., **48**: 254‐60.

Bennett, M. S., Triemer R. E. 2014 The genus *Cyclidiopsis*: an obituary. *J. Eukaryot. Microbiol.,* **61**:166‐72.

Bennett, M. S., Wiegert, K. E. & Triemer, R.E. 2014 Characterization of *Euglenaformis* gen. nov. and the chloroplast genome of *Euglenaformis* [*Euglena*] *proxima* (Euglenophyta). *Phycologia,* **53**: 66–73.

Breglia, S. A., Yubuki, N. & Leander, B. S., 2013 Ultrastructure and molecular phylogenetic position of *Heteronema scaphurum*: a eukaryovorous euglenid with a cytoproct. *J. Eukaryot. Microbiol.,* **60**: 107‐20.

Cavalier‐Smith, T. 2013 Early evolution of eukaryote feeding modes, cell structural diversity, and classification of the protozoan phyla Loukozoa, Sulcozoa, and Choanozoa. *Eur. J. Protistol.,* **49**: 115‐178.

Cavalier‐Smith, T. 2016 Higher classification and phylogeny of Euglenozoa. *Eur. J. Protistol.,* **56**: 250‐276.

Cavalier‐Smith, T., Chao, E. E. & Vickerman, K. 2016 New phagotrophic euglenoid species (new genus *Decastava*;* Scytomonas saepesedens*;* Entosiphon oblongum*), Hsp90 introns, and putative euglenoid Hsp90 pre‐mRNA insertional editing. *Eur. J. Protistol.,* **56**: 147‐170.

Čepička, I., Dolan, M. F. & Gile, H. F. 2017. Parabasalia. In: Archibald, J. M.,Simpson, A. G. B., and Slamovits, C., eds. Handbook of the Protists (Second Edition of the Handbook of Protoctista by Margulis et al.) Springer Reference Works (e‐book) https://doi.org/10.1007/978-3-319-32669-6_12-1.

Čepička, I., Hampl, V. & Kulda, J. 2010 Critical taxonomic revision of parabasalids with description of one new genus and three new species. *Protist,* **161**: 400‐433.

Chan, Y.‐F., Moestrup, Ø. & Chang, J. 2013 On *Keelungia pulex* nov. gen. et nov. sp., a heterotrophic euglenoid flagellate that lacks pellicular plates (Euglenophyceae, Euglenida). *Eur. J. Protistol*., **49**: 15‐31.

Gibson, W. 2017 Kinetoplastea. In: Archibald, J. M.,Simpson, A. G. B. & Slamovits, C., eds. Handbook of the Protists (Second Edition of the Handbook of Protoctista by Margulis et al.) Springer Reference Works (e‐book) https://doi.org/10.1007/978-3-319-32669-6_12-1.

Hampl, V. 2017 Preaxostyla. In: Archibald, J. M., Simpson,G. B., and Slamovits, C., eds. Handbook of the Protists (Second Edition of the Handbook of Protoctista by Margulis et al.) Springer Reference Works (e‐book) https://doi.org/10.1007/978-3-319-32669-6_12-1

Hanousková, P., Táborský, P. & Čepička, I. 2018 *Dactylomonas* gen. nov., a novel lineage of heterolobosean flagellates with unique ultrastructure, closely related to the amoeba *Selenaion koniopes* Park, de Jonckheere & Simpson, 2012. *J. Eukaryot. Microbiol*., *in press*.

Heiss, A. A., Kolisko, M., Ekelund, F., Brown, M. W., Roger, A. J. & Simpson, A. G. B. 2018. Combined morphological and phylogenomic re‐examination of malawimonads, a critical taxon for inferring the evolutionary history of eukaryotes. *R. Soc. Open Sci*., **5**:171707. https://doi.org/10.1098/rsos.171707.

Kamikawa, R., Kolisko, M., Nishimura, Y., Yabuki, A., Brown, M. W., Ishikawa, S. A., Ishida, K., Roger, A. J., Hashimoto, T. & Inagaki, Y. 2014 Gene content evolution in discobid mitochondria deduced from the phylogenetic position and complete mitochondrial genome of *Tsukubamonas globosa*. *Genome Biol. Evol.,* **6**: 306‐315.

Karnkowska, A., Bennett, M. S., Watza, D., Kim, J. I., Zakryś, B. & Triemer, R. E. 2015 Phylogenetic relationships and morphological character evolution of photosynthetic euglenids (Excavata) inferred from taxon‐rich analyses of five genes. *J. Eukaryot. Microbiol.,* **62**: 362‐73.

Kaufer, A., Ellis, J., Stark, D. & Barratt, J. 2017 The evolution of trypanosomatid taxonomy. *Parasites & Vectors,* **10**: 287.

Kostygov, A.Y. & Yurchenko, V. 2017 Revised classification of the subfamily Leishmaniinae (Trypanosomatidae). *Folia Parasitol*., **64**: 020.

Kulda, J., Nohýnková, E. & Čepička, I. 2017 Retortamonadida (with notes on *Carpediemonas*‐like organisms and Caviomonadidae). In: Archibald, J. M.,Simpson, A. G. B., and Slamovits, C., eds. Handbook of the Protists (Second Edition of the Handbook of Protoctista by Margulis et al.) Springer Reference Works (e‐book) https://doi.org/10.1007/978-3-319-32669-6_12-1.

Lax, G. & Simpson, A. G. B. 2013 Combining molecular data with classical morphology for uncultured phagotrophic euglenids (Excavata): a single‐cell approach. *J. Eukaryot. Microbiol*., **60**: 615‐25.

Leander, B. S., Lax, G., Karnkowska, A. & Simpson, A. G. B. 2017 Euglenida. In: Archibald, J. M.,Simpson, A. G. B., and Slamovits, C., eds. Handbook of the Protists (Second Edition of the Handbook of Protoctista by Margulis et al.) Springer Reference Works (e‐book) https://doi.org/10.1007/978-3-319-32669-6_12-1

Lee, W. J. & Simpson, A. G. 2014 Ultrastructure and molecular phylogenetic position of *Neometanema parovale* sp. nov. (*Neometanema* gen. nov.), a marine phagotrophic euglenid with skidding motility. *Protist*,** 165**: 452‐72.

Lee, W. J. & Simpson, A. G. 2014 Morphological and molecular characterisation of *Notosolenus urceolatus* Larsen and Patterson 1990, a member of an understudied deep‐branching euglenid group (petalomonads). *J. Eukaryot. Microbiol*., **61**: 463‐79.

Lukeš, J., Butenko, A., Hashimi, H., Maslov, D.A., Votýpka, J. & Yurchenko, V. 2018 Trypanosomatids are much more than just trypanosomes: clues from the expanded family tree. *Trends Parasitol*., **34**: 466‐480.

Pánek, T., Simpson, A. G. B., Brown, M. W. & Dyer, B. D. 2017 Heterolobosea. In: Archibald, J. M.,Simpson, A. G. B., and Slamovits, C., eds. Handbook of the Protists (Second Edition of the Handbook of Protoctista by Margulis et al.) Springer Reference Works (e‐book) https://doi.org/10.1007/978-3-319-32669-6_12-1.

Pánek T., Táborský P., Pachiadaki, M. G., Hroudová, M., Vlček, Č., Edgcomb, V. P. & Čepička, I. 2017 Combined culture‐based and culture‐independent approaches provide insights into diversity of jakobids, an extremely plesiomorphic eukaryotic lineage. *Front. Microbiol.,* **6**: 1288.

Okamoto, N., Gawryluk, R.M.R., del Campo, J., Strassert, J.F.H., Lukeš, J., Richards, T.A., Worden, A.Z., Santoro, A.E. & Keeling, P.J. 2018 A revised taxonomy of diplonemids including the Eupelagonemidae n. fam. and a type species, *Eupelagonema oceanica* n. gen. & sp. *J. Euk. Microbiol*., (in press)

Radek, R., Strassert, J. F., Krüger, J., Meuser, K., Scheffrahn, R. H. & Brune, A. 2014 Phylogeny and ultrastructure of *Oxymonas jouteli*, a rostellum‐free species, and *Opisthomitus longiflagellatus* sp. nov., oxymonadid flagellates from the gut of *Neotermes jouteli*. *Protist,* **165**: 384‐99.

Simpson, A. G. B. 2017. Jakobida. In: Archibald, J. M.,Simpson, A. G. B., & Slamovits, C., eds. Handbook of the Protists (Second Edition of the Handbook of Protoctista by Margulis et al.) Springer Reference Works (e‐book) https://doi.org/10.1007/978-3-319-32669-6_12-1.

Strassert, J. F. H., Tikhonenkov, D. V., Pombert, J. F., Kolisko, M., Tai, V., Mylnikov, A. P. & Keeling, P. J. 2016. *Moramonas marocensis* gen. nov., sp. nov.: a jakobid flagellate isolated from desert soil with a bacteria‐like, but bloated mitochondrial genome. *Open Biol*., **6**: 150239.

Tashyreva, D., Prokopchuk, G., Yabuki, A., Kaur, B., Faktorová, D., Votýpka, J., Kusaka, C., Fujikura, K., Shiratori, T., Ishida, K.‐I., Horák, A. & Lukeš, J. 2018. Phylogeny and morphology of new diplonemids from Japan. *Protist,* **169**: 158‐179.

Treitli, S. C., Kotyk, M., Yubuki, N., Jirounková, E., Vlasáková, J., Smejkalová, P., Šípek, P., Čepička, I. & Hampl, V. 2018. Molecular and morphological diversity of the oxymonad genera *Monocercomonoides* and *Blattamonas* gen. nov. *Protist*. **169**:744–783. https://doi.org/10.1016/j.protis.2018.06.005

Yamaguchi, A., Yubuki, N. & Leander, B. S. 2012. Morphostasis in a novel eukaryote illuminates the evolutionary transition from phagotrophy to phototrophy: description of *Rapaza viridis* n. gen. et sp. (Euglenozoa, Euglenida). *BMC Evol. Biol.,* **12**: 29.

Yubuki, N., Zadrobílková, E. & Čepička, I. 2017. Ultrastructure and molecular phylogeny of *Iotanema spirale* gen. nov. et sp. nov., a new lineage of endobiotic Fornicata with strikingly simplified ultrastructure. *J. Eukaryot. Microbiol*., **64**: 422‐433.

Zhang, Q., Táborský, P., Silberman, J. D., Pánek, T., Čepička, I. & Simpson, A. G. B. 2015. Marine isolates of *Trimastix marina* form a plesiomorphic deep‐branching lineage within Preaxostyla, separate from other known trimastigids (*Paratrimastix* n. gen.). *Protist,* **166**: 468‐91.

### Incertae sedis Eukarya

Foissner, I. & Foissner, W. (1993): Revision of the family Spironemidae Doflein (Protista, Hemimastigophora), with description of two new species, *Spironema terricola* n. sp. and *Stereonema geiseri* n. g., n. sp. *J. Eukaryot. Microbiol.,* **40**:422–438.

Glücksman, E., Snell, E. A. & Cavalier‐Smith, T. (2013) Phylogeny and evolution of Planomonadida (Sulcozoa): eight new species and new genera *Fabomonas* and *Nutomonas*. *Europ. J. Protistol.,* **49**:179–200.

Hausmann, K., Weitere, M., Wolf, M. & Arndt, H. 2002. *Meteora sporadica* gen. et sp. nov. (Protista incertae sedis) — an extraordinary free‐living protist from the Mediterranean deep sea. *Europ. J. Protistol.,* **38**:171–177.

Janouškovec, J., Tikhonenkov, D. V., Burki, F., Howe, A. T., Rohwer, F. L., Mylnikov, A. P. & Keeling, P. J. (2017). A New Lineage of Eukaryotes Illuminates Early Mitochondrial Genome Reduction. Curr. Biol., **27**(23), 3717–3724.e5. https://doi.org/10.1016/j.cub.2017.10.051

### CRuMs

Brown, M. W., Heiss, A. A., Kamikawa, R., Inagaki, Y., Yabuki, A., Tice A. K., Shiratori, T., Ishida, K., Hashimoto, T., Simpson, A. G. B. & Roger, A. J. 2018. Phylogenomics places orphan protistan lineages in a novel eukaryotic super‐group. *Genome Biol. Evol.,* **10**:427–433.

Brugerolle, G. 2006. Description of a new freshwater heterotrophic flagellate *Sulcomonas lacustris* affiliated to the collodictyonids. *Acta Protozool*., **45**:175–182.

Brugerolle, G., Bricheux, G., Philippe, H. & Coffe, G. 2002. *Collodictyon triciliatum* and *Diphylleia rotans* ( = *Aulacomonas submarina*) form a new family of flagellates (Collodictyonidae) with tubular mitochondrial cristae that is phylogenetically distant from other flagellate groups. *Protist*,** 153**:59–70.

Glücksman, E., Snell, E. A., Berney, C., Chao, E. E., Bass, D. & Cavalier‐Smith, T. 2011. The novel marine gliding zooflagellate genus *Mantamonas* (Mantamonadida ord. n.: Apusozoa). *Protist*,** 162**:207–221.

Yabuki, A., Ishida, K. & Cavalier‐Smith, T. 2013. *Rigifila ramosa* n. gen., n. sp. a filose apusozoan with a distinctive pellicle, is related to *Micronuclearia*. *Protist*,** 164**:75–88.

## Appendix S2. Trophic functional groups across protist diversity.

Trophic functional group assignments facilitate interpretation of community structure and food web assembly from DNA sequence information identification of “operational taxonomic unit” (OTU). Here we tried to provide details of trends in eating habits of non‐parasitic protists. Many genera have never been cultured so their feeding preferences are unknown. For definition of terms, please refer to the main text, page 6.

Across protists, in most cases it is safe to assume that species within a genus are most likely to have the same trophic function, with rare exceptions. However, it is not the case in some taxa, where there can be considerable variation between species within a genus. That is the case, for example, in Dinoflagellata, Arcellinida, and Euglyphida.

In the **Amoebozoa**, genera can be assumed to ingest bacteria by phagocytosis with the following exceptions: *Amphizonella* (Corycida) and *Dermamoeba* also ingest Cyanobacteria; the Euamoebida genera *Amoeba, Cashia, Chaos, Polychaos, Trichamoeba and Deuteramoeba* are also cytotrophic; amoebae of the genera *Polychaos* and *Pseudothecamoeba* ingest diatoms. Some species or genera have a preference for heterotrophic bacteria over of photosynthetic ones, or even discriminate against Cyanobacteria such as *Copromyxa* and *Copromyxella*. In Protosteliida, *Protostelium* spp. prefer yeasts but some will also eat bacteria, and several can eat coccoid green algae, except *P. nocturnum* which only eats bacteria; they can be cannibalistic in culture as can Cavosteliida and some Protosporangiidae. In Schizoplasmodiidae, all do best in culture on a mixture of yeast and bacteria, though some will survive on bacteria alone; when they eat yeasts, they puncture the cell wall and ingest only the protoplast. *Multicilia* (Holomastigida) is cytotrophic on other amoebae. Dictyostelia are primarily bacterivorous but they will eat yeasts in culture, and can also be cannibalistic in culture; zygotes are cannibalistic exclusively. Myxogastria ciliated stage is bacterivorous, their plasmodia are omnivorous and often cannibalistic when they first form. The Entamoebae are mostly commensal or parasitic and can eat bacteria by phagotrophy. A few strains of *Sappinia* and *Acanthamoeba* can be parasitic. Some Archamoebae, *Tricholimax*,* Thecamoeba, Korotnevella, Gocevia, Paragocevia, Leptomyxa* and *Mayorella* species are omnivores. There are some diatom specialists as in *Difflugia* and *Phryganella*. The ability to feed on yeast might be more widespread than reported. Similarly, in their habitat some soil amoebae probably ingest small Cercozoa such as Glissomonads. In the Arcellinida, at least species of *Nebela* and *Cryptodifflugia* are known to prey on nematodes. The tendency to cannibalism amongst some amoebae may be due to crowding in culture.

In Arcellinida certain species of *Cucurbitella, Difflugia, Heleopera, Hyalosphenia* and *Netzelia* form symbiosis with Trebouxiophyta species and can be considered mixotrophic.

Free‐living basal genera of **Holozoa** and **Nucletmycea** are amoeboid, phagotrophic on bacteria. Parasitic ones, including Microsporidia, are intracellular parasites. The Choanoflagellata feed on bacteria by filtration through the collar. The Fungi are saprotrophic on external substrate, obtaining their nutrient by osmotrophy through a chitinous cell wall. Lichenous fungi are capable of photosynthesis. Mycorrhizal fungi receive most or all of their organic molecules from the host plant. Many Fungi are parasitic (see table 2). For details of substrate preference the reader is referred to FUNGuild (Nguyen et al., 2016, https://github.com/UMNFuN/FUNGuild).

In the **Archaeplastida**, ignoring the Embryophyta, all Chloroplastida are photosynthetic with *chl a, b,* with the exception of *Helicosporidium* (insect gut parasite)*, Polytomella, Polytoma, Prototheca* which have 2° lost photosynthesis and are strictly osmotrophic. Among the **Rhodophyceae**, some Cyanidiales are facultative heterotrophs. Amongs the red algae certain species in several genera have lost photosynthesis and are parasites of other red algae. For example: *Gracilariophila oryzoides, Harveyella mirabilis, Choreocolax polysiphoniae, Janczewskia gardneri, Faucheocolax attenuata, Bostrychiocolax australis, Dawsoniocolax bostrychiae, Coccotylus hartzii, Epulo multipedes* (see Blouin and Lane, 2012, Table S1). Nearly all of the parasitic genera end with the term “colax” to indicate the lifestyle. Most parasites are sister species with the hosts, and many of these genera need to be merged with the genus of their hosts, as they were described separately. This is in progress but obviously correcting the taxonomy is a slow process.

The **Cryptista** ingest bacteria prey by phagocytosis, and the photosynthetic ones are mixotrophic. In **Haptophyta**, Coccolithales are autotrophic, while genera without scales can be mixotrophic or heterotrophic on bacteria, with or without haptonema contributing to bacteria capture. The Centroplasthelida are typically cytotrophic.

In the **Apicomplexa**, all species are historically treated as parasites. Recent observation suggest some may be endobionts as commensals or at least not as parasites, especially among some invertebrate hosts. However, there are no free‐living species known. The presence of a relic plastid genome in the Apicomplexa, and apicoplast in some, provides useful metabolic targets for drug treatment development. In contrast, both Dinoflagellata and Ciliophora provide a wide diversity of trophic functional groups.

The **Dinoflagellata** functional assignments vary between closely related genera, and are shown in Appendix 2, Table 2.1.

The **Ciliophora** are to be assumed to ingest bacteria by filtration except as noted below. Some genera of Karyorelictea (eg. Kentrophoridae) can be considered grazers as they live on sand surfaces covered in bacteria, and often harbour symbiotic bacteria. We discriminate between surface‐feeding and water‐column filtration feeding. Surface‐feeding ciliophores spend most of their time gathering food particles from surfaces rather than by swimming in a volume of water. Thus clearing rates are calculated by surface area rather than volume of liquid. Most ciliophores (with exceptions noted in table 2.2) ingest bacteria, and many also ingest small protists (labelled omnivorous), while others can ingest larger protists by predation. Many omnivores can also ingest some detritus if the cytostome permits. In Ciliophora one should not assume that a small cell or a small cytostome implies a bacteria diet. There are parasites, histophagous species, endocommensals, and epibionts. The Stichotrichida contain species that can ingest larger protists and even small ciliophores for example in some species of Oxytrichidae. The Litostomatea are redoubtable predatory cytotrophs, and some can ingest prey larger than themselves. Entodiniomorphida are often (or typically) commensals in rumen. Among the Colpodea a small number of genera are cytotrophic, for example Bursaria (and maybe other Bursariomorphida). In the Nassulida, *Nassula* is phycotrophic by swallowing and can ingest cyanobacteria filaments. In the Prorodontida several genera, eg. *Coleps*, are attracted to dead or wounded invertebrates to the leaking tissues. The Astomatia can be found as symbionts in Oligochaetae gut. Some Tetrahymenida genera are parasitic or histophagous. The Suctoria (Phyllopharyngea) use specialised tentacles to capture and manipulate prey, typically other ciliophores or small invertebrates; they are cytotrophic predators and 3° consumers as predators of invertebrates. The Rhynchodia (Phyllopharyngea) are parasites of bivalves. Among the Scuticociliatia the Philasterida contain cytotrophic genera, for example *Uronema*. See Appendix table 2.2 for more details.

Also in the **Alveolates**, the colpodellids, *Colponema*,* Acavomonas*, and *Oxyrrhis* are cytotrophic predators, while the Perkinsidae are parasitic as trophozoites.

**Stramenopiles** in the Ochrophyta are almost all photosynthetic. The Actinophryidae are cytotrophic. The Opalinata, Bicosoecida, and Placidida are phagotrophic on bacteria, with the exception of the Proteromonada and *Blastocystis* (osmotrophic) which are intestinal commensals or parasites. The Hyphochytriales and Peronosporomycetes are saprotrophs, but the latter contains numerous plant parasites. The Pirsoniales are parasites on diatoms.

**Rhizaria**. The Cercomonadida, Paracercomonadida, and Glissomonadida can be assumed to be phagotrophic predators on bacteria, with the notable exception of the Viridiraptoridae which are phycotrophic (mycotrophy not known) by penetration. The Vampyrellida are mycotrophic or phycotrophic. The Euglyphida show some variation, although most are phagotrophic on bacteria. Observations indicate that the larger the cell the more likely it is to be cytotrophic; in fact the larger species cannot survive on bacteria alone. Many Hyalospheniidae need to prey at least partly on Euglyphida and use the scales to build their own test. Symbiosis occurs between Trebouxiophyta (photosynthetic) with several genera of Rhizaria. This implicates at least some species of *Placocista* (Euglyphida), *Amphitrema* and *Archerella* (Labyrinthulomycetes). Chlorarachnea are photosynthetic but some species are mixotrophic on bacteria. In the Endomyxa, the Phytomyxea are plant root parasites, while the Ascetosporea are parasites of invertebrates.

The Foraminifera are primarily bacteria eaters, but can ingest detritus, other protists, and larger forms can prey on small invertebrates.

Among the **Discoba** (Excavates) Heterolobosea graze bacteria by phagocytosis, except *Stephanopogon* and *Creneis* which are cytotrophic. Some strains of *Naegleria fowleri* can cause primary amoebic meningoencephalitis, penetrating through the nose. The Jakobida and *Tsukubomonas* are predatory on bacteria which are ingested by phagocytosis. The Diplonema are cytotrophic by phagocytosis and have an important role in aphotic marine waters; some species are parasitic or symbiotic with animals. Among the Euglenida (Discoba) the Aphagea (*Rhabdomonas, Gyropaigne, Menoidium, Parmidium and Rhabdospira*), *Astasia* and *Distigma* are osmotrophic; Euglenophyceae (except *Rapaza*) are photosynthetic with *chl a, b* in plastids, or secondarily osmotrophic, and *Rapaza* is cytotrophic; the Peranemids, Anisonemids, Pleotiids, and Petalomonadida are phagotrophic. Certain clades graze on bacteria by phagotrophy or are cytotrophic predators. Among the Kinetoplastea (Discoba) all except the Trypanosomatida are predatory phagotrophs on bacteria. The Trypanosomatida are animal blood and tissue parasites, except *Phytomonas* which lives in plant latex vessels, and all are transferred by insect vectors.

The **Metamonada** (Fornicata, Parabasalia, and Preaxostyla Excavates) are predatory or grazers on bacteria, ingested by phagocytosis. The Preaxostyla are endosymbiont detritivores that phagocytose wood microchips, and acquire many nutrients by osmotrophy from syntrophic bacteria; some genera may also ingest bacteria by phagocytosis. *Giardia* (Diplomonadida) are osmotrophic gut parasites. Several genera have species that can be parasitic in humans and other animals.

**Human parasites.** For protists that are human parasites, see Adl and Matheson (2019) in ASM‐Manual of Clinical Microbiology chapter 132 (Taxonomy and Classification of Human Eukaryotic Parasites), 12th edn.

**Definitions.** We further provide descriptions and clarifications for symbisois, microbiome, holobiont, intra‐ cellular and extracellular locations, that relate to interactions between organisms.

**Symbiosis** is a sustained relationship between at least two individuals (same or different species), living in direct contact or close enough one to the other, during part or the whole live cycle of the two partners. This interaction is transmitted either vertically (from one generation to the other) or horizontally (acquired de novo at each generation). Symbiosis is the antonym of transitory interactions (predatory). A symbiosis can be beneficial for both partners (mutualism) or at the other extreme, deleterious for one of these partners (parasite or parasitoid). In general, the larger species of this association is called the host, the smallest one is the symbiont. Examples of symbiosis: photosymbiosis (corals and *Symbiodinium*, kleptoplast), mycorrhizae, lichens, parasitism and hyperparasitism (parasite of parasite). Some specialist predators may also be considered as symbionts (example: *Helcion* living exclusively on laminarian). A virus is a parasitic symbiont.

The **microbiome** is a microbial community living in, on or at the close vicinity of an individual. Examples: gut community, biosphere of phytoplanktonic cells, microbes living on the skin/cuticule of metazoan, in the rhizopshere, microbes living in specialized tissues, or part of the cell. Unicellular symbionts are part of the microbiome of their host.

**Holobiont**. Ecoevolutionary concept considering an individual together with its microbiome as the true unit on which evolutionary processes will act.

**Intracellular (endocellular) versus extracellular**. Intracellular symbionts are those where the parasite cytoplasm and nucleus enter inside the host and reside inside the host cell. In some extracellular symbionts, part of the cytoplasm (including the mitochondrion) may penetrate inside the host cell, but never the parasite nucleus. Endobionts penetrate the host tissue between cells, or inside a matrix, but do not reside inside host cells. Mycorrhizae are symbiotic endobionts.

## Appendix 2, Table 2.1. Trophic Functional Assignments for Dinoflagellates where known

**L** = photosynthetic, **M** = mixotrophic (photosynthesis and phagotrophy), **B** = phagotrophic on bacteria, **C** = cytotrophic (phagotrophic on protists), **H** = heterotrophic. In general, few dinoflagellates nutritional strategy is really investigated, documented, and understood. For most of the taxa we can only indirectly judge and it is more or less a guess. Some genera are difficult to assign because some species are mixotrophic, and for other species we do not know whether they feed in addition to phototrophy. In *Amphidinium* s.s. for example we have pure heterotrophic but also photosynthetic and mixotrophic species. For these genera there is species level variation in trophic functions. Heterotrophic taxa are the species that have to feed on something, because they are not photosynthetic but we do not know what they feed on (only food bodies or vacuoles are visible but the content cannot be identified and the feeding process has not been recorded).

**Table:** 

•Alveolata
••Dinoflagellata
•••Noctilucales
*Abedinium * **H** *, Cachonodinium * **H** *, Craspedotella * **H** *, Cymbodinium * **H** *, Kofoidinium * **C** *, Leptodiscus * **H** *, Noctiluca * **M C** *, Petalodinium * **H** *, Pomatodinium * **H** *, Scaphodinium * **H** *, Spatulodinium * **M H**.
•••Dinophyceae
••••Gymnodiniphycidae
•••••*Gymnodinium*
*Barrufeta * **M C** *, Dissodinium * **L M C** *, Erythropsidinium * **C** *, Greuetodinium * **C**?*, Gymnodinium * **L M** *, Lepidodinium * **L** *, Nematodinium * **M C** *, Nusuttodinium * **M C** *, Pellucidodinium * **C** *, Polykrikos * **C M** *, Proterythropsis * **C** *, Warnowia * **C**.
•••••*Amphidinium * **L M C**.
••••• *Gyrodinium * **C**.
•••••Kareniaceae
*Brachidinium * **M** *, Karenia * **M B C** *, Karlodinium * **M B C** *, Takayama * **M C**.
•••••Ceratoperidiniaceae
*Ceratoperidinium * **L**,* Kirithra * **L**.
•••••Torodiniales
*Kapelodinium * **C** *, Torodinium * **M**
•••••*Levanderina*
*Levanderina * **M C**.
•••••*Margalefidinium*
*Margalefidinium * **M C**
•••••*Cochlodinium*
*Cochlodinium strangulatum * **M**?
•••••Ptychodiscales
*Achradina * **B** *, Amphitolus, Balechina * **H C**?*, Ptychodiscus * **L** *, Sclerodinium*.
•••••Borghiellaceae.
*Baldinia * **M C** *, Borghiella * **L**.
•••••Tovelliaceae.
*Bernardinium, Esopotrodinium * **M C** *, Jadwigia * **L** *, Tovellia * **L M.**
•••••Suessiaceae
*Ansanella * **L** *, Asulcocephalium * **L** *, Biecheleria * **M C** *, Biecheleriopsis * **L M** *, Leiocephalium * **L** *, Pelagodinium * **L** *, Polarella * **L** *, Prosoaulax * **M C** *, Protodinium, Symbiodinium * **L M B** *, Yihiella * **L M.**
••••Peridiniphycidae
•••••Gonyaulacales
*Alexandrium * **L M C** *, Amylax * **L M C** *, Ceratium * **L M C** *, Ceratocorys * **L** *, Coolia * **L** *, Fukuyoa * **L** *, Fragilidium*
**L M C** *, Gambierdiscus * **L M** *, Goniodoma * **L** *, Gonyaulax * **L M C** *, Lingulodinium * **L M C** *, Ostreopsis * **L M,**
*Pentaplacodinium * **L** *, Peridiniella * **L** *, Protoceratium * **L M, ** *Pyrocystis * **L** *, Pyrodinium * **L** *, Pyrophacus * **L** *, Tripos * **L M C**.
•••••Peridiniales
*Amphidiniopsis * **H C**?*, Archaeperidinium * **C** *, Blastodinium * **M, ** *Diplopelta * **C** *, Diplopsalis * **C** *, Diplopsalopsis * **C** *, Herdmania * **H C**?*, Niea * **C** *, Oblea * **C** *, Palatinus * **L** *, Parvodinium * **L** *, Peridinium * **L** *, Peridiniopsis * **L C** *, Preperidinium * **C** *, Protoperidinium * **C** *, Qia * **C** *, Vulcanodinium * **L**.
•••••Thoracosphaeraceae
*Aduncodinium * **C** *, Amyloodinium, Apocalathium, Blastodinium, Chimonodinium, Cryptoperidiniopsis * **M C** *, Duboscquodinium * **C** *, Ensiculifera * **L** *, Leonella, Luciella * **C** *, Naiadinium * **L M**?*, Paulsenella * **C** *, Pentapharsodinium * **L** *, Pfiesteria * **M C** *, Scrippsiella * **L M C ** *, Stoeckeria * **C** *, Theleodinium * **L M**?*, Thoracosphaera * **L** *, Tintinnophagus * **C** *, Tyrannodinium * **C**.
•••••Podolampadaceae
*Blepharocysta * **C**?*, Gaarderiella, Heterobractum, Lissodinium, Mysticella, Podolampas * **M C**.
•••••Kryptoperidiniaceae
*Blixaea * **L M, ** *Durinskia * **L M, ** *Galeidinium * **L M, ** *Kryptoperidinium * **L M, ** *Unruhdinium * **L**.
•••••*Heterocapsaceae*
*Heterocapsa * **L M C**.
•••••Amphidomataceae
*Amphidoma * **L** *, Azadinium * **L**.
•••••Oxytoxaceae.
*Corythodinium * **M C**?*, Oxytoxum * **L M**?
•••••Centrodiniaceae
*Centrodinium * **L**
••••Dinophysales
*Amphisolenia * **L M, ** *Citharistes * **M B** *, Dinofurcula * **H** *, Dinophysis * **M C** *, Histioneis * **M B** *, Latifascia * **H** *, Metadinophysis * **L** *, Metaphalacroma * **H** *, Ornithocercus * **M B** *, Oxyphysis * **C** *, Parahistioneis * **M B** *, Phalacroma * **(M) C** *, Pseudophalacroma * **H** *, Sinophysis * **H M**? **B** *, Triposolenia * **M.**
••••Prorocentrales
*Mesoporus * **L** *, Prorocentrum * **L M C**.
•••Incertae sedis Dinoflagellata:
e.g., *Adenoides * **L** *, Akashiwo * **M C** *, Amphidiniella * **L M, ** *Ankistrodinium * **C** *, Apicoporus * **M C** *, Bispinodinium * **L M, ** *Bysmatrum * **L M C** *, Cabra * **H C**?*, Cladopyxis, Crypthecodinium * **C** *, Cucumeridinium * **H** *, Dactylodinium * **L M, ** *Dicroerisma * **H** *, Gloeodinium, Grammatodinium * **L M, ** *Gynogonadinium, Gyrodiniellum * **C** *, Halostylodinium * **L** *, Heterodinium * **L**?*, Moestrupia * **L** *, Paragymnodinium * **M C** *, Phytodinium, Pileidinium * **L** *, Plagiodinium * **L** *, Planodinium * **H C**?*, Pseudadenoides * **L** *, Pseudothecadinium * **L** *, Pyramidodinium * **L** *, Roscoffia * **H C**?*, Rhinodinium * **H C**?*, Sabulodinium * **H** *, Sphaerodinium * **L** *, Spiniferodinium * **L M, ** *Testudodinium * **L M, ** *Thecadinium * **L M,** *Thecadiniopsis * **L** *, Togula * **L**.
•••Incertae sedis Dinoflagellata [Blastodiniales Chatton 1906, no longer valid]. Amyloodinium*, Apodinium*, Cachonella, Crepidoodinium **M, ** *Haplozoon, Oodinium * **M, ** *Piscinodinium * **M, ** *Protoodinium * **M** . *ectoparasites

## Appendix 2, Table 2.2. Trophic functional assignments in CILIOPPHORES

All genera feed on bacteria by filtration **(B)** except where noted. Omnivorous genera **(O)** ingest small protists in addition to bacteria. Those that are labelled cytrophic **(C)** need to ingest protists for growth, in addition to bacteria that are co‐ingested. Many omnivores can occasionally ingest detritus **(D)**. Those that feed on fungi **(F)** do so by digesting a small hole, while those that feed on other filaments **(f)** (Cyanobacteria and algae) do so by swallowing. Predators **(P)** are distinguished from those that obtain their food particles by filtration (the default state in ciliophores) or by grazing where noted. Histophagous genera (P*) are distinguished from parasites **(X)** and commensals (*). Osmotrophic **(S)** genera feed extensively, or exclusively, by pinocytosis and other membrane transport mechanisms.

**Table:** 

•Alveolata
••Ciliophora
•••Postodesmatophora
••••Karyorelictea
•••••Kentrophoridae(*Kentrophoros) * **B**
•••••Loxodida
••••••Cryptopharyngidae (*Cryptopharynx) * **O C**
••••••Loxodidae (*Loxodes) * **O C**
•••••Geleiidae (*Geleia*) **O C**
••••Heterotrichea
•••••Blepharismidae (*Blepharisma) * **B**
•••••Climacostomidae (*Climacostomum) * **O**
•••••Condylostomatidae (*Chattonidium*,* Condylostoma) * **B**
•••••Fabreidae (*Fabrea) * **B**
•••••Gruberiidae (*Gruberia) * **B**
•••••Coliphorina
••••••Folliculinidae (*Folliculina) * **B**
••••••Maristentoridae (*Maristentor) * **O**
•••••Peritromidae (*Peritromus) * **B**
•••••Spirostomidae (*Anigsteinia*,* Spirostomum) * **B**
•••••Stentoridae (*Stentor) * **O** or **C** or **P**
•••Intramacronucleata
••••SAL
*Incertae sedis* SAL: Mesodiniidae (*Mesodinium) Incertae sedis* SAL: *Phacodinium*
••••• Spirotrichea
••••••Euplotia
•••••••Euplotida
••••••••Aspidiscidae (*Aspidisca) * **O**
••••••••Certesiidae (*Certesia) * **O**
••••••••Euplotidae (*Euplotes) * **O**
••••••••Gastrocirrhidae (*Gastrocirrhus) * **O**
••••••••Uronychidae (*Diophrys*,* Uronychia) * **O**
•••••••Discocephalida
••••••••Discocephalidae(*Discocephalus*,* Prodiscocephalus*,* Paradiscocephalus) * **O**
••••••••Pseudoamphisiellidae (*Leptoamphisiella*,* Pseudoamphisiella) * **O**
••••••Perilemmaphora
•••••••Hypotrichia
••••••••Stichotrichida
•••••••••Amphisiellidae (*Amphisiella*,* Bistichella) * **O**
•••••••••Atractosidae (*Atractos) * **O**
•••••••••Epiclintidae (*Epiclintes) * **O**
•••••••••Gonostomatidae (*Cotterillia, Gonostomum) * **O**
•••••••••Halteriidae (*Halteria*,* Meseres) * **O**
•••••••••Holostichidae (*Holosticha, Uncinata) * **O**
•••••••••Kahliellidae (*Deviata*,* Kahliella*) **O**
••••••••• Keronidae (*Kerona*) **O**
•••••••••Oxytrichidae (*Cyrtohymena*,* Gastrostyla*,* Oxytricha*,* Stylonychia) * **O**
•••••••••Parabirojimidae (*Parabirojimia*,* Tunicothrix) * **O**
•••••••••Plagiotomidae (*Plagiotoma) * **O**
•••••••••Psammomitridae (*Psammomitra) * **O**
•••••••••Pseudoamphisiellidae (*Pseudoamphisiella) * **O**
•••••••••Psilotrichidae (*Psilotricha, Urospinula) * **O**
•••••••••Schmidingerotrichidae (*Schmidingerothrix) * **O**
•••••••••Spirofilidae (*Spirofilopsis*,* Strongylidium) * **O**
•••••••••Trachelostylidae (*Trachelostyla) * **O**
•••••••••Uroleptidae (*Paruroleptus*,* Uroleptus) * **O**
••••••••Urostylida
•••••••••Bergeriellidae (*Bergeriella, Neourostylopsis) * **O**
•••••••••Hemicycliostylidae (*Hemicycliostyla, Australothrix) * **O**
•••••••••Pseudokeronopsidae (*Apoholosticha*,* Pseudokeronopsis) * **O**
•••••••••Pseudourostylidae (*Pseudourostyla) * **O**
•••••••••Urostylidae (*Bakuella, Diaxonella*,* Urostyla) * **O C**
•••••••Oligotrichea
••••••••Oligotrichida
•••••••••Cyrtostrombidiidae (*Cyrtostrombidium) * **O**
•••••••••Pelagostrombidiidae (*Pelagostrombidium) * **O**
•••••••••Strombidiidae (*Strombidium) * **O**
•••••••••Tontoniidae (*Laboea*,* Tontonia) * **O**
••••••••Choreotrichida
•••••••••Strobilidiina
••••••••••Leegaardiellidae (*Leegaardiella) * **O**
••••••••••Lohmanniellidae (*Lohmanniella) * **O**
••••••••••Strobilidiidae (*Strobilidium) * **O**
••••••••••Strombidinopsidae (*Strombidinopsis) * **O**
•••••••••Tintinnina (particle size ingested is determined by the lorica diameter)
••••••••••Ascampbelliellidae (*Ascampbelliella) * **O**
••••••••••Cyttarocylididae (*Cyttarocylis) * **O**
••••••••••Dictyocystidae (*Dictyocysta) * **O**
••••••••••Epiplocylididae (*Epiplocylis) * **O**
••••••••••Eutintinnidae (*Dartintinnus, Eutintinnus) * **O**
••••••••••Favellidae (*Favella) * **O**
••••••••••Nolaclusiliidae (*Nolaclusilis) * **O**
••••••••••Petalotrichidae (*Petalotricha) * **O**
••••••••••Ptychocylididae (*Cymatocylis*,* Ptychocylis) * **O**
••••••••••Rhabdonellidae (*Metacylis*,* Rhabdonella*,* Schmidingerella * **O**
••••••••••Stenosemellidae (*Stenosemella) * **O**
••••••••••Tintinnidae (*Amphorellopsis*,* Salpingacantha*,* Salpingella*,* Tintinnus) * **O**
••••••••••Tintinnidiidae (*Tintinnidium) * **O**
••••••••••Undellidae (*Undella) * **O**
••••••••••Xystonellidae (*Dadayiella*,* Parafavella*,* Xystonella) * **O**
•••••• Licnophoridae (*Licnophora*,* Prolicnophora) * **O**
••••••Kiitrichidae (*Caryotricha*,* Kiitricha) * **O**
•••••Lamellicorticata
••••••Armophorea
*Incertae sedis* Armophorea: Mylestomatidae (*Mylestoma) * **O**
•••••••Metopida **anaerobic‐aerotolerant**
••••••••Metopidae (*Metopus) * **O**
••••••••Apometopidae (*Cirranter*,* Urostomides) * **O**
••••••••Tropidoatractidae (*Palmarella*,* Tropidoatractus) * **O**
•••••••Clevelandellida anaerobic, gut *
••••••••Clevelandellidae (*Clevelandella) * **O**
••••••••Inferostomatidae (*Inferostoma) * **O**
••••••••Neonyctotheridae (*Neonyctotherus) * **O**
••••••••Nyctotheridae (*Nyctotherus) * **O**
••••••••Sicuophoridae (*Sicuophora) * **O**
•••••••Caenomorphidae (*Caenomorha*) **O**
•••••••Odontostomatida
••••••••Discomorphellidae (*Discomorphella) * **B**
••••••••Epalxellidae *(Epalxella, Saprodinium) * **B**
•••••• Litostomatea
•••••••Rhynchostomatia
••••••••Dileptida
•••••••••Dileptidae (*Apodileptus*,* Dileptus*,* Pseudomonilicaryon) * **P**
•••••••••Dimacrocaryonidae (*Dimacrocaryon, Monomacrocaryon*,* Rimaleptus) * **P**
••••••••Tracheliidae (*Trachelius) * **O P**
•••••••Haptoria
••••••••Lacrymariidae (*Lacrymaria) * **P**
••••••••Haptorida
•••••••••Enchelyodonidae (*Enchelyodon, Fuscheria) * **P**
•••••••••Homalozoonidae (*Homalozoon) * **P**
•••••••••Pleuroplitidae (*Pleuroplites) * **P**
••••••••Didiniidae *(Didinium, Monodinium) * **P**
••••••••Pleurostomatida
•••••••••Amphileptidae (*Amphileptus) * **P**
•••••••••Litonotidae (*Litonotus) * **P**
•••••••••Kentrophyllidae (*Kentrophyllum, Epiphyllum) * **P**
••••••••Spathidiida
•••••••••Acropisthiidae (*Acropisthium*,* Chaenea) * **P**
•••••••••Actinobolinidae (*Actinobolina) * **P**
•••••••••Apertospathulidae (*Apertospathula) * **P**
•••••••••Enchelyidae (*Enchelys) * **P**
•••••••••Pseudoholophryidae (*Pseudoholophrya) * **P**
•••••••••Spathidiidae (*Spathidium) * **P**
•••••••••Trachelophyllidae (*Trachelophyllum) * **P**
•••••••Helicoprorodontidae (*Helicoprorodon) * **P**
•••••••Trichostomatia **(anaerobic endosymbiont** in vertebrates**)**
••••••••Vestibuliferida **(anaerobic endosymbiont** in fish**)**
•••••••••Balantidiidae (*Balantidium, some **X**, Neobalantidium) * **B**
•••••••••Buetschliidae (*Buetschlia) * **B**
•••••••••Paraisotrichidae (*Paraisotrichia) * **B**
•••••••••Protocaviellidae (*Protocaviella) * **B**
•••••••••Protohalliidae (*Protohallia) * **B**
•••••••••Pycnotrichidae (*Pycnothrix*, perhaps *Buxtonella*) **B**
•••••••Isoendo
••••••••Isotrichidae (*Dasytricha*,* Isotricha) * (anaerobic gut * in ungulate ruminants**) B**
••••••••Entodiniomorphida
•••••••••Blepharocorythina (anaerobic gut * in mammals**)**
••••••••••Blepharocorythidae (*Blepharocorys) * **B**
••••••••••Parentodiniidae (*Parentodinium) * **B**
••••••••••Pseudoentodiniidae (*Pseudoentodinium) * **B**
•••••••••Entodiniomorphina
••••••••••Cycloposthiidae (*Cycloposthium) * **B**
••••••••••Gilchristidae (*Gilchristia) * **B**
••••••••••Ophryoscolecidae (*Entodinium*,* Ophryoscolex*,* Polyplastron) * **B**
••••••••••Polydiniellidae (*Polydiniella) * **B**
••••••••••Rhinozetidae (*Rhinozeta) * **B**
••••••••••Spirodiniidae (*Spirodinium) * **B**
••••••••••Telamodiniidae (*Telamodinium) * **B**
••••••••••Troglodytellidae (*Troglodytella) * **B**
••••••••Macropodiniida (anaerobic gut * in fore‐stomach of marsupial macropodids and vombatids**)**
••••••••••Amylovoracidae (*Amylovorax) * **B**
••••••••••Macropodiniidae (*Macropodinium) * **B**
••••••••••Polycostidae (*Polycosta) * **B**
••••CONTHREEP
*Incertae sedis* CON‐threeP: *Askenasia,* Cyclotrichiidae, *Paraspathidium,*
Pseudotrachelocercidae, Discotrichidae. **B**
••••• Phyllopharyngea
••••••Synhymeniida **f**, cyanobacteria
•••••••Nassulopsidae (*Nassulopsis) * **f**
•••••••Orthodonellidae (*Orthodonella*,* Zosterodasys) * **f**
•••••••Scaphidiodontidae (*Chilodontopsis*,* Scaphidiodon) * **f**
•••••••Synhymeniidae (*Synhymenia) * **f**
••••••Subkinetalia
•••••••Cyrtophoria
••••••••Chlamydodontida
•••••••••Chilodonellidae (*Chilodonella) * **O C**
•••••••••Chitonellidae (*Chitonella) * **O C**
•••••••••Chlamydodontidae (*Chlamydodon) * **O C**
•••••••••Gastronautidae (*Gastronauta) * **O C**
•••••••••Kryoprorodontidae (*Gymnozoum) * **O C**
•••••••••Lynchellidae (*Chlamydonella*,* Lynchella) * **O C**
••••••••Dysteriida
•••••••••Dysteriidae (*Dysteria*,* Trochilia) * **O C**
•••••••••Hartmannulidae *Hartmannula*) **O C**
•••••••••Kyaroikeidae (*Kyaroikeus*) **O C**
•••••••••Plesiotrichopidae (*Plesiotrichopus*) **O C**
•••••••••Chonotrichia
••••••••••Exogemmida **?**
•••••••••••Chilodochonidae (*Chilodochona*)
•••••••••••Filichonidae (*Filichona*)
•••••••••••Helichonidae (*Heliochona*)
•••••••••••Lobochonidae (*Lobochona*)
•••••••••••Phyllochonidae (*Phyllochona*)
•••••••••••Spirochonidae (*Spirochona*)
••••••••••Cryptogemmida **?**
•••••••••••Actinichonidae (*Actinichona*)
•••••••••••Echinichonidae (*Echinichona*)
•••••••••••Inversochonidae (*Inversochona*)
•••••••••••Isochonidae (*Isochona*)
•••••••••••Isochonopsidae (*Isochonopsis*)
•••••••••••Stylochonidae (*Stylochona*)
•••••••Rhynchodia
••••••••Hypocomidae (*Hypocoma) * **S, X** in marine invertebrates
••••••••Rhynchodida
•••••••••Ancistrocomidae (*Ancistrocoma) * **C** or **X** in marine invertebrates
•••••••••Sphenophryidae (*Sphenophrya) * **C** or **X** in marine invertebrates
•••••••Suctoria (**P** larger species can capture small invertebrates)
••••••••Exogenida
•••••••••Allantosomatidae (*Allantosoma*) **C P**
•••••••••Dentacinetidae (*Dentacineta*) **C P**
•••••••••Dendrosomididae (*Dendrosomides*) **C P**
•••••••••Ephelotidae (*Ephelota*) **C P**
•••••••••Lecanophryidae (*Lecanophrya*) **C P**
•••••••••Metacinetidae (*Metacineta) * **C P**
•••••••••Manuelophryidae (*Manuelophrya*) **C P**
•••••••••Ophryodendridae (*Ophryodendron*) **C P**
•••••••••Paracinetidae (*Paracineta*) **C P**
•••••••••Phalacrocleptidae (*Phalacrocleptes*)
•••••••••Podophryidae (*Podophrya*) **C P**
•••••••••Praethecacinetidae (*Praethecacineta*) **C P**
•••••••••Rhabdophryidae (*Rhabdophrya*) **C P**
•••••••••Severonidae (*Severonis*) **C P**
•••••••••Spelaeophryidae (*Spelaeophrya*) **C P**
•••••••••Tachyblastonidae (*Tachyblaston*) **C P**
•••••••••Thecacinetidae (*Thecacineta*) **C P**
••••••••Endogenida
•••••••••Acinetidae (*Acineta*) **C P**
•••••••••Acinetopsidae (*Acinetopsis*) **C P**
•••••••••Choanophryidae (*Choanophrya*) **C P**
•••••••••Corynophryidae (*Corynophrya*) **C P**
•••••••••Dactylostomatidae (*Dactylostoma*) **C P**
•••••••••Dendrosomatidae (*Dendrosoma*) **C P**
•••••••••Endosphaeridae (*Endosphaera*) **C P**
•••••••••Erastophryidae (*Erastophrya*) **C P**
•••••••••Pseudogemmidae (*Pseudogemma*) **C P**
•••••••••Rhynchetidae (*Rhyncheta*) **C P**
•••••••••Solenophryidae (*Solenophrya*) **C P**
•••••••••Tokophryidae (*Tokophrya*) **C P**
•••••••••Trichophryidae (*Trichophrya*) **C P**
••••••••Evaginogenida
•••••••••Cometodendridae (*Cometodendron*) **C P**
•••••••••Cyathodiniidae (*Cyathodinium*) **C P**
•••••••••Dendrocometidae (*Dendrocometes*) **C P**
•••••••••Discophryidae (*Discophrya*) **C P**
•••••••••Enchelyomorphidae (*Enchelyomorpha*) **C P**
•••••••••Heliophryidae (*Heliophrya*) **C P**
•••••••••Periacinetidae (*Periacineta*) **C P**
•••••••••Prodiscophryidae (*Prodiscophrya*) **C P**
•••••••••Rhynchophryidae (*Rhynchophrya*) **C P**
•••••••••Stylocometidae (*Stylocometes*) **C P**
•••••••••Trypanococcidae (*Trypanococcus*) **C P**
••••• Colpodea
••••••Bursariomorphida
•••••••Bryometopidae (*Bryometopus*,* Thylakidium*) **P**
•••••••Bursaridiidae (*Bursaridium*,* Paracondylostoma*) **P**
•••••••Bursariidae (*Bursaria*) **P**
••••••Colpodida (smaller species <20 µm are probably ingesting primarily bacteria)
*Incertae sedis* Colpodida: Bardeliellidae, Hausmanniellidae, Ilsiellidae, Marynidae, Pseudochlamydonellidae. **O**
•••••••Bryophryina
••••••••Bryophryidae (*Bryophrya*) **O**
••••••••Sandmanniellidae (*Sandmanniella*) **O**
•••••••Colpodina
••••••••Colpodidae (*Colpoda) * **O**
••••••••Grandoriidae (*Grandoria*) **O**
••••••••Tillinidae (*Tillina*) **O**
••••••• Grossglockneriidae (*Grossglockneria*,* Pseudoplatyophrya) * **F**
••••••Cyrtolophosidida
•••••••Cyrtolophosididae (*Cyrtolophosis) * **O**
•••••••Kreyellidae (*Kreyella*) **O**
••••••Platyophryida (**C** ingest small green algae)
•••••••Ottowphryidae (*Ottowphrya*,* Platyophryides*) **C**
•••••••Platyophryidae (*Platyophrya*) **C**
•••••••Sagittariidae (*Sagittaria*) **C**
•••••••Sorogenidae (*Sorogena*) **C**
•••••••Woodruffiidae (*Etoschophrya*,* Rostrophrya*,* Woodruffia*) **C**
••••• Nassophorea
••••••Colpodidiidae (*Colpodidium) * **B**
••••••Nassulida (**f**, cyanobacteria)
•••••••Furgasoniidae (*Furgasonia*,* Wolfkosia*) **f**
•••••••Nassulidae (*Nassula*,* Obertrumia*) **f**
••••••Microthoracida
•••••••Microthoracidae (*Drepanomonas, Microthorax*) **f**
•••••••Leptopharyngidae (*Pseudomicrothorax, Leptopharynx * **B**) **f**
••••• Prostomatea
•••••• Apsiktratidae (*Apsikrata*) **O**
••••••Prorodontida
•••••••Balanionidae (*Balanion*) **C D**
•••••••Cryptocaryonidae (*Cryptocaryon*) **C D**
•••••••Colepidae (*Coleps*,* Plagiopogon*) **C D**
•••••••Holophryidae (*Holophrya*) **C D**
•••••••Lagynidae (*Lagynus*) **C D**
•••••••Metacystidae (*Metacystis*,* Vasicola*) **C D**
•••••••Placidae (*Placus*) **C D**
•••••••Plagiocampidae (*Plagiocampa*) **C D**
•••••••Prorodontidae (*Prorodon*) **C D**
•••••••Urotrichidae (*Urotricha*) **C D**
••••• Plagiopylea
••••••Plagiopylida
•••••••Epalxellidae (*Epalxella) * **B**
•••••••Plagiopylidae (*Plagiopyla*) **B**
•••••••Sonderiidae (*Sonderia*) **B**
•••••••Trimyemidae (*Trimyema*) **B**
••••• Oligohymenophorea
••••••Apostomatia
•••••••Apostomatida
••••••••Colliniidae (*Collinia*,* Metacollinia*) **S**
••••••••Cyrtocaryidae (*Cyrtocaryum*) **S**
••••••••Foettingeriidae (*Foettingeria*) **S**
••••••••Pseudocolliniidae (*Fusiforma, Pseudocollinia*) **S**
•••••••Astomatophorida
••••••••Opalinopsidae *(Opalinopsis) * **S**
•••••••Pilisuctorida
••••••••Conidophryidae (*Conidophrys) * **S**
••••••Astomatia
•••••••Anoplophryidae 910 (*Anoplophrya*) **S**
•••••••Buetschliellidae (*Buetschliella*) **S**
•••••••Clausilocolidae (*Clausilocola*) **S**
•••••••Contophryidae (*Contophyra*) **S**
•••••••Haptophryidae (*Haptophrya*) **S**
•••••••Hoplitophryidae (*Hoplitophrya*) **S**
•••••••Intoshellinidae (*Intoshellina*) **S**
•••••••Maupasellidae (*Maupasella*) **S**
•••••••Radiophryidae (*Radiophrya*) **S**
••••••Hymenostomatia
•••••••Tetrahymenida (verify each species life cycle, macrostome stage is **C**)
*Incertae sedis* Tetrahymenida: Trichospiridae (*Trichospira) * B P*
••••••••Curimostomatidae (*Curimostoma*) B P*
••••••••Glaucomidae (*Glaucoma*) B P*
••••••••Spirozonidae (*Spirozona*) B P*
••••••••Tetrahymenidae (*Tetrahymena*) B P*
••••••••Turaniellidae (*Colpidium*,* Dexiostoma*,* Turaniella*) B P*
•••••••Ophryoglenida
••••••••Ichthyophthiriidae (*Ichthyophthirius*) P* P
••••••••Ophryoglenidae (*Ophryoglena*) P* P
••••••Peniculia
•••••••Peniculida
••••••••Clathrostomatidae (*Clathrostoma*) **O**
••••••••Frontoniidae (*Disematostoma*,* Frontonia*) **O**
••••••••Lembadionidae (*Lembadion*) **O**
••••••••Maritujidae (*Marituja*) **O**
••••••••Neobursaridiidae (*Neobursaridium*) **O**
••••••••Parameciidae (*Paramecium*) **O**
••••••••Paranassulidae (*Paranassula*) **O**
••••••••Stokesiidae (*Stokesia*) **O**
•••••••Urocentridae (*Urocentrum) * **O**
••••••Peritrichia
•••••••Sessilida
••••••••Astylozoidae (*Astylozoon*,* Hastatella*) **O**
••••••••Ellobiophryidae (*Ellobiophrya*) **O**
••••••••Epistylididae (*Epistylis*) **O**
••••••••Lagenophryidae (*Lagenophrys*) **O**
••••••••Operculariidae (*Opercularia*) **O**
••••••••Rovinjellidae (*Rovinjella*) **O**
••••••••Scyphidiidae Kahl, 133 (*Scyphidia*) **O**
••••••••Termitophryidae (*Termitophrya*) **O**
••••••••Usconophryidae (*Usconophrys*) **O**
••••••••Vaginicolidae (*Cothurnia*,* Pyxicola*,* Thuricola*,* Vaginicola*) **O**
••••••••Vorticellidae (*Carchesium, Vorticella) * **O**
••••••••Zoothamniidae (*Haplocaulus*,* Zoothamnium*) **O**
•••••••Mobilida
••••••••Polycyclidae (*Polycycla*) **O**
••••••••Trichodinidae (*Trichodina*) **O**
••••••••Trichodinopsidae (*Trichodinopsis*) **O**
••••••••Urceolariidae (*Leiotrocha*,* Urceolaria*) **O**
••••••Scuticociliatia
•••••••Philasterida
••••••••Cohnilembidae (*Cohnilembus*) **O**
••••••••Cryptochilidae (*Cryptochilum*.) **O**
••••••••Entodiscidae (*Entodiscus*) **O**
••••••••Entorhipidiidae (*Entorhipidium*) **O**
••••••••Orchitophryidae (*Orchitophrya*) **O**
••••••••Paralembidae (*Anophrys*,* Paralembus*) **O**
••••••••Parauronematidae (*Parauronema*) **O**
••••••••Philasteridae (*Kahlilembus*,* Philaster*) **O**
••••••••Pseudocohnilembidae (*Pseudocohnilembus*) **O**
••••••••Schizocaryidae (*Schizocaryum*) **O**
••••••••Thigmophryidae (*Thigmophrya*) **O**
••••••••Thyrophylacidae (*Thyrophylax*) **O**
••••••••Uronematidae (*Uronema*) **C**
••••••••Urozonidae (*Urozona*) **O**
•••••••Pleuronematida
••••••••Calyptotrichidae (*Calyptotricha*) **O**
••••••••Conchophthiridae (*Conchophthirus*) **O**
••••••••Ctedoctematidae (*Ctedoctema*) **O**
•••••••• Cyclidiidae (*Cristigera*,* Cyclidium*) **O**
••••••••Dragescoidae (*Dragescoa*) **O**
••••••••Eurystomatellidae (*Eurystomatella*) **O**
••••••••Histiobalantiidae (*Histiobalantium*) **O**
••••••••Peniculistomatidae (*Peniculistoma*) **O**
••••••••Pleuronematidae (*Pleuronema*) **O**
••••••••Thigmocomidae (*Thigmocoma*) **O**
•••••••Thigmotrichida
••••••••Ancistridae (*Ancistrum*) **B**
••••••••Hemispeiridae (*Hemispeira*) **B**
••••••••Hysterocinetidae (*Hysterocineta*) **B**
••••••••Paraptychostomidae (*Paraptychostomum*) **B**
•••••••Loxocephalida
••••••••Cinetochilidae (*Cinetochilum*,* Sathrophilus*) **B**
••••••••Loxocephalidae (*Cardiostomatella*,* Dexiotricha*,* Loxocephalus*) **B**

## Appendix 3. Table 3.1.

Protist names and common names for East Asian translations

**Table:** 

**Higher level ranks and supergroups**	**Phylum**	**Important sub‐divisions in Chinese characters**	**Common name translation**
Amoebozoa	Tubulinea	Corycida (皮殼葉状根足綱) 皮壳管状根足纲	Leathery‐shell amoebae
		Echinamoebida (多針葉状根足綱) 多针管状根足纲	Amoebae with spiny pseudopodia
		Elardia (三組葉状根足綱) 三组管状根足纲	Amoeboid group including three groups (**E**uamoebida, **L**eptomyxida, **Ar**cellinida)
		Arcellinida (有殼葉状變形綱) 有壳管状根足纲	Testate amoebae partially enclosed in a simple shell
	Evosea	Variosea (多圓錐型 根足綱) 多圆锥型根足纲	Amoebae with many conical‐shaped pseudopodia
		Eumycetozoa (眞菌根足綱) 真粘菌根足纲	Genuine (mushroom) slime mold amoebae
		Cutosea (眞皮根足綱) 真皮根足纲	Amoebae with a discrete skin
		Archamoebea (原始根足綱) 古变形纲	Ancient (first) amoebae
	Discosea	Stygamoebida (細片根足綱) 细片根足纲	Amoebae with pseudopodia resembling tooth‐pick or splinters
		Centramoebia (中心體根足綱) 中心体根足纲	Centrosome‐bearing amoebae
Incertae sedis Holozoa		Filasterea (星狀絲足綱) 星状丝足纲	Rounded amoebae with pseudopodia like fingers
		Ichtyosporea (孢子型魚病根足綱) 孢子型鱼病根足纲	Fish pathogenic amoebae forming a spore
		Choanoflagellata (立襟鞭毛綱) 领鞭毛纲	Flagellates with a collar‐like ring
	Porifera	Demospongiae (普通海綿綱) 普通海绵纲	Common sponges
		Homoslceromorpha (同骨海綿綱) 同骨海绵纲	Sponge with undifferentiated cytoskeleton
		Calcarea (石灰海綿綱) 石灰海绵纲	Chalk sponges
		Hexactinellida (玻璃海綿綱) 玻璃海绵纲	Glass sponges
Nucletmycea	Opisthosporidia	Aphelidea (藻寄生性根足綱) 藻寄生性根足纲	Amoeboid endobiotic parasitoids of algae
		Microsporidia (原始寄生性擬菌綱) 原始寄生拟菌纲	Primitive fungus‐like parasite
		Blastocladiales(厚壁囊菌綱) 厚壁囊菌纲	Fungi having a thick‐walled resting spore
		Neocallimastigaceae (新多鞭毛菌綱) 新美鞭菌纲	New fungi having many/pretty flagella
		Chytridiomycota (壺狀菌綱) 壶菌纲	Fungi resembling a broken‐cracked egg
		Mucoromycota (粘液菌綱) 粘液菌纲	Mucoid or sugar fungi
		Zoopagomycota (動物生菌綱) 捕虫霉纲	Fungi growing on animals, Fungi able to catch bugs
		Taphrinomycotina (外囊菌綱) 外囊菌纲	Fungi having an outer ascus
		Saccharomycetales (酵母菌綱) 酵母菌纲	Fungi associated with fermentation
		Pezizomycotina (周鉢菌綱) 盘菌纲	(Bowl‐shaped fungi), plate‐shaped fungi
		Agaricomycotina (擔子菌綱) 担子菌纲	Fungi forming a sterigma
		Pucciniomycotina (銹菌綱) 柄锈菌纲	Fungi with stem and is associated with rust disease
		Ustilaginomycotina (黑穗菌綱) 黑穗菌纲	Fungi associated with smut disease
		Wallemiomycotina (無子實體菌綱) 无子实体菌纲	Fungi without palisade of basidia
	Rhodophyceae	Proteorhodophytina (原始紅藻綱) 原始红藻纲	Primitive red algae
		Eurhodophytina (眞正紅藻綱) 真红藻纲	Genuine red alage, which have two phases or three phases
	Chloroplastida	Chlorophyta (綠藻綱) 绿藻纲	Green algae
		Charophyta (輪藻綱) 轮藻纲	Wheel‐shaped green algae
Bigyra		Nanomonadea (矮小鞭毛綱) 微小鞭毛纲	Tiny brown flagellates
		Opalinata (皮下共生鞭毛綱) 蛙片虫纲	Animal endobionts; Flagellates mostly found in frog, with a flat shape like a slice
		Placidida (小突起鞭毛綱) 小突起鞭毛纲	Flagellate having a papilla
		Bicosoecida (毫髮鞭毛綱) 毫发鞭毛纲	Flagellates having tiny flagellar hairs
		Labyrinthulomycetes (網形鞭毛綱) 网丝鞭毛纲	Gliding flagellates producing a network of filaments
		Pseudophyllomitidae (非附着鞭毛綱) 无附着鞭毛纲	Flagellates lacking the two adhering flagella
Gyrista		Developea (後固着鞭毛綱) 后附鞭毛纲	Flagellates with adhering posterior flagellum
		Hyphochytriales (菌絲體鞭毛綱) 菌丝体鞭毛纲	Flagellates having hypha‐like structures
		Peronosporomycetes (植物病原體擬菌綱) 植病拟菌纲	Fungus‐like plant pathogens
		Pirsoniales (硅藻寄生綱) 硅藻寄生纲	Diatom parasites
		Actinophryidae (太陽綱) 太阳纲	Sunlight (or star light)‐shaped protozoa
		Chrysophyceae (黃褐藻綱) 金藻纲	Golden brown algae
		Eustigmatales (眞眼點褐藻綱) 真眼点褐藻纲	Brown algae with a big eye spot
		Phaeophyceae (大褐藻綱) 褐藻纲	The large brown algae
		Phaeothamniophyceae (黃赤藻綱) 褐枝藻纲	Yellow‐red algae with branches
		Raphidophyceae (針鞭毛藻綱) 针胞藻纲	Algae with needle shaped flagellum
		Xanthophyceae (黃綠藻綱) 黄绿藻纲	Yellow‐green algae
		Bolidomonas (迅游泳藻綱) 迅游藻纲	Diatom‐like algae with rapid swimming
	Diatomeae	Diatomeae (硅褐藻綱) 硅褐藻纲	Brown algae like glass box with lid
		Dictyochophyceae (硅質鞭毛藻綱) 硅鞭藻纲	Algae producing a siliceous skeleton
		Pelagophyceae (浮生褐藻綱) 浮生褐藻纲	Filamentous brown algae living in the sea; brown algae living a planktonic habitat
		Pinguiophyceae (脂褐藻綱) 脂褐藻纲	Brown algae containing (a high concentration of) fatty acids
Incertae sedis Alveolata		Colpodellida (捕食性鞭毛綱) 捕食鞭毛纲	Predatory flagellates
	Dinoflagellata	Syndiniales (共生性渦鞭毛藻綱) 共生涡鞭藻纲	Parasitic dinoflagellates, symbiotic dinoflagellates
		Noctilucales (夜光藻綱) 夜光藻纲	Algae with bioluminescence
		Dinophyceae (渦鞭毛藻綱) 涡鞭藻纲	Algae with spiraling motility, algal flagellates with a spiral or girdle groove
	Apicomplexa	Aconoidasida (無圓錐頂端複合體綱) 无圆锥体顶复纲	Apicomplexa lacking a conoid
		Conoidasida (圓錐頂端複合體綱) 圆锥体顶复纲	Apicomplexa possessing a conoid
	Ciliophora	Karyorelictea (核殘跡纖毛綱) 核残迹纤毛纲	Ciliophores having relict of parents’ macronulei
		Heterotrichea (異毛纖毛綱) 异毛纤毛纲	Ciliophores with different length of flagella
		Spirotrichea (型 旋纖毛綱) 旋唇纤毛纲	Ciliophores with spiraling adoral zone of membranelles
		Armophorea (鬪帽纖毛綱) 盔帽纤毛纲	Ciliophores having the appearance of military helmets
		Litostomatea (裂口纖毛綱) 裂口纤毛纲	Ciliophores with a cytostome with oral dome
		Phyllopharyngea (葉咽纖毛綱) 叶咽纤毛纲	Ciliophores with a leaf‐shaped cytopharynx
		Colpodea (腎形纖毛綱) 肾形纤毛纲	Ciliophores with a kidney shape
		Prostomatea (前口纖毛綱) 前口纤毛纲	Ciliophores with cytostome at the anterior pole
		Plagiopylea (斜毛纖毛綱) 斜毛纤毛纲	Ciliophores with oblique slit flagella
		Oligohymenophorea (貧膜纖毛綱) 寡膜纤毛纲	Ciliophores with a small paroral membrane
		Nassophorea (篮口纖毛綱) 篮口纤毛纲	Ciliophores showing a basket‐shaped oral structure
	Cercozoa	Silicofilosea (硅質絲狀根足綱) 硅质丝足纲	Filose amoebae covered by siliceous or glass scales, vase‐shaped shell
	Foraminifera	Monothalamea (單房室有孔綱) 单房室有孔纲	Foraminiferans with single chamber test
		Tubothalamea (管狀有孔綱) 管状有孔纲	Foraminiferans with tubular chamber test
		Lagenida (單層有孔綱) 瓶状有孔纲	Foraminiferans with monolamellar test
	Radiolaria	Acantharia (放射棘綱) 等辐骨纲	Protozoa with axopods and filopodia; protist with radiated spicula of same length
		Polycystinea (多孔囊綱) 多孔纲	Protozoa (with a sac) covered by many pores
	Metamonada	Fornicata (拱門形纖維綱) 拱形纤维纲	Protozoa with an arched B‐fiber
		Parabasalia (副基体綱) 副基体纲	Protozoa with one or more parabasal apparatus
		Preaxostyla (二重纖維綱) 二重纤维纲	Protozoa with I‐fiber with double‐cross matrix
	Discoba	Jakobida (單背翼綱) 单背翼纲	Protozoa with a single dorsal vane in the posterior flagellum
		Heterolobosea (噴出形根足綱) 异叶足纲	Amoebae with eruptive pseudopodia; amoebae with differentiated leaf‐shaped pseudopodia
	Euglenozoa	Kinetoplastid (運動核質鞭毛類) 动质体纲	Protozoa with kinetoplast
		Euglenid (軟豆鞭毛類) 软豆鞭毛类	Yellow‐green flagellates
	Haptophyta	Prymnesiophyceae (碳酸鑛物化藻綱) 碳酸质鳞片藻纲	Algae with CO_2_‐mineralized scales
		Coccolithophorid (圓石藻類) 颗石藻类	Haptophyte with calcareous (chalk) scales
	Centroplasthelida	Pterocystida (無外骨格鞭毛綱) 无外骨骼鞭毛纲	Protozoa without any exoskeletal elements
		Panacanthocystida (石鱗鞭毛綱) 石鳞鞭毛纲	Protozoa with siliceous scales or with organic spicules
	Cryptista	Cryptophyceae (隱鞭毛藻型) 隐藻纲	New algae with prominent ejectisomes; cryptic algae

## Footnotes

1 The ability of a unicellular organism or a cell type in a multicellular organism to actively change the conformation of the entire cell body by extending and retracting pseudopodia; pseudopodia are used for cell movement over the substratum and/or for feeding.

2 Monolayer of scales covering the cell adhering to the substratum from the dorsal surface; the ventral surface of the cell remains free. Known in amoebae of the genus *Cochliopodium*.

3 Layer of fibrous material covering the cell, adhering to the substratum from the dorsal surface; the ventral surface remains free. Known in amoebae of the genera *Gocevia, Paragocevia* and *Ovalopodium*.

4 Single amoeboid cell differentiates into a usually stalked, subaerial structure that supports one to many propagules termed spores. As defined here, this kind of sporocarp has only ever been observed in Amoebozoa and is potentially synapomorphic for Amoebozoa. Should this prove the case, non‐sporocarpic amoebozoans are the products of reductive evolution.

5 Amoebae aggregate into a multicellular mass that develops into a multicellular, subaerial fruiting body consisting of either distinct stalk cells and spores or non‐differentiated encysted cells (usually also called spores). Sorocarpic development is found in two lineages of amoebozoans, the Dictyostelia (Eumycetozoa) and in *Copromyxa* (Tubulinea).

6 The species *Schoutedamoeba minuta* described by Van Vichelen et al. (2016) has a hartmannelid morphology (monopodial cells with pronounced frontal hyaline cap) but in SSU tree it shows affinities with Variosea, although with no support. More robust data are necessary to clarify its position among Amoebozoa.

7 The taxon name *Stereomyxa ramosa* is used in Tekle et al. (2016) and Tekle and Wood (2017) for an isolate that is a distinct genus named *Dracoamoeba* (see Tice et al. 2016). To date, no molecular data on a true *Stereomyxa* are available and thus it remains *incertae sedis*.

8 The name *Unda* is used in Tekle et al. (2016) as well as in Tekle and Wood (2017) for an isolate of *Vannella* as noted in Cavalier‐Smith et al. (2016) and Kang et al. (2017).

9 Variable cell projections, smooth in outline, with rounded tips, which participate in the relocation of the main cytoplasmic mass of the cell and include both the granuloplasm and the hyaloplasm (sensu Smirnov 2008).

10 Schaudinn (1899) reported a complex life cycle in *Trichosphaerium* (Corycida) that included biciliated stages, which undergo copulation; no further confirmation of this observation has been obtained.

11 Strain numbers and source data for these isolates are provided by Kang et al. (2017).

12 The genus *Atrichosa* by Cavalier‐Smith et al. 2016 is considered here a junior synonym of *Trichosphaerium* until the opposite is shown. The position of the genera *Penardochlamys, Microcorycia, Zonomyxa* and *Parmulina*, which were listed by Meisterfeld (2002) under “Microcoryciidae” is not clear; by their morphological characters they may belong to this lineage as well but this requires demonstration by molecular data.

13 The SSU rRNA sequence of *Trigonopyxis arcula* AY848967 is almost identical to *Bullinularia indica* AY848970, and represents probably a contamination.

14 This species typically positioned as sister clade of both Sphaerothecina and Difflugina in beta‐tubulin (Lahr et al. 2011), SSU rRNA (Lara et al. 2008) and multigene phylogenies (Lahr et al. 2013), so it is listed here as a separate lineage.

15 In the most complete version spores from a sporocarp germinate as ciliated‐amoebae, cells that are reversibly amoeboid or ciliated then go on to develop into an obligate amoeboid stage that cannot produce cilia, with the obligate amoeba differentiating into one or more sporocarps. However, some members are always ciliated or obligate amoebae. The kinetid structures of swimming stages are diagrammed in Spiegel et al. (2017), Mikryukov and Mylnikov (1998), Hibberd (1983), and Pánek et al. (2016).

16 Recent papers with relatively broad taxon sampling, based on SSU phylogeny (Berney et al. 2015) and multigene phylogeny (Kang et al. 2017) suggest grouping of some variosean genera into higher rank clades. However, SSU phylogeny shows little or no statistical support for many of these groupings, while many important variosean taxa are not yet represented in the multigene trees. Therefore, we prefer to be cautious about including certain higher level taxa proposed in these studies at this time. Hence, we list most variosean clades under the similarly high level regardless of the traditional ranks until more robust groups are established.

17 The genus *Planoprotostelium* is subsumed into *Protostelium* (Shadwick et al. 2017).

18 This is the only group revealed in the paper by Lahr et al. (2011) which we suggest to apply because it is fully supported in a phylogenomic study by Kang et al. (2017) and combines several monotypic lineages; many of them group with each other in SSU trees as well.

19
*Coenonia* was seen only once by Van Tieghem (1884) who described but never illustrated it. On the basis of his description, it seems reasonable to conclude it was a dictyostelid.

20 Many taxa in need of revision because of rampant paraphyly.

21 The species *Mycamoeba gemmipara* in Blandenier et al. (2017) groups with *Dermamoeba*; however. the phylogenetic analysis in this paper uses a limited number of taxa and does not show Dermamoebida as a clade, so we list this genus as a separate branch until it position is better resolved.

22 Two known species of this genus—*J*. *chaetognathi* (Grassi 1881) and *J. pigmentifera* (Grassi 1881) are not triangular but monopodial in locomotion which may be a consequence of their parasitic life style. No molecular data on this genus are available, so it is left among the Dactylopodida provisionally, basing on the presence of a kinetoplastid intracellular symbiont (PLO), which appeares to have originated only once in the evolution of paramoebids (Sibbald et al. 2017).

23 This genus may be a junior synonym of *Vannella*, being a life form of some *Vannella* species.

24 These genera group together in some SSU phylogenetic trees, but usually they appear as separate branches in phylogenetic studies. In the phylogenomic study of Kang et al. (2017) they do not form a clade, but this is the only region of the tree that is not fully supported. Taking into account the superficial similarity of the ultrastructure, including the unique shape of the mitochondrial cristae, in these two genera we suggest keeping this assemblage as a potential branch in the tree unless the opposite is proven with increased taxon sampling.

25 Teretosporea Torruella et al. 2015 (R) is a monophyletic clade consisting of at least *Corallochytrium* and Ichthyosporea*;* Pluriformea Hehenberger et al. 2017 (R) is a monophyletic clade consisting of at least *Corallochytrium* and *Syssomonas*. These are two competing phylogenetic hypotheses.

26 Choanomonada was an unfortunate error in spelling that was corrected in an erratum, Journal of Eukaryotic Microbiology 60 (3): 321, published online March 11, 2013.

27 The clade that comprises the Metazoa and Choanoflagellata is called Choanozoa Brunet and King 2017 [Choanozoa Cavalier‐Smith et al. 1991].

28 The clade comprising the Porifera, *Trichoplax*, Cnidaria, Ctenophora, and Bilateria is called Metazoa, Metazoa Haeckel 1874.

29 Although monotypic, genetic diversity of isolates indicate there are probably multiple genera, and it is probably a sister clade to Cnidaria (Schierwater and DeSalle 2018; and Srivastava et al. 2008).

30 Nucletmycea and Holomycota were proposed at about the same period and they are to be considered synonymous, but Nucletmycea has nomenclatural priority having been published first. Since, Holomycota has come into more usage in favour of the symmetry with Holozoa.

31 There is no agreement on the definition, thus placement, of a clade named Fungi. Variations differ from the historical understanding of Fungi that included social amoebae, now dispersed across protists, and several other clades also dispersed across protists.

32 Holomycota is the sister lineage to Holozoa and is comprised of Fungi and the group Rotosphaerida containing nucleariids. In this classification, we include Opisthosporidia (Karpov et al. 2014), comprising the endoparasitic lineages Rozellida (Cryptomycota) Aphelida and Microsporidia, in the Fungi, noting that this is an ongoing point of contention. New genomic and transcriptomic data provide modest to poor support for the monophyly of an Opisthosporidia clade (Toruella et al. 2018), but multiple molecular studies nonetheless support the placement of all three lineages at the base of the Fungi (Torruella et al. 2019; Karpov et al. 2017a, 2017b; Letcher et al. 2017; Lopez‐Escardo et al. 2018). A major point of contention has been the fact that Opisthosporidia all have a vegetative phase that is unbounded by a cell wall, unlike most Fungi, and that Aphelida and *Rozella* have an intracellular (parasitic) phagotrophic feeding mode (Karpov et al. 2014; Powell et al. 2017). On the other hand, all of the Opisthosporidia share with other Fungi a chitinous cell wall at some stage in their life cycle. Recent analysis of an aphelid transcriptome (the lineage that has most retained ancestral characters within opisthosporidians) suggests a genomic composition and metabolism more aligned with the free‐living chytrid fungi with degradative enzymes, than other Opisthosporidia (Toruella et al. 2019). But an alternative interpretation places emphasis on Opisthosporida cell biology which is more similar to other Opisthokonta than to Fungi, because of the phagotrophic nature of their nutrition. Ultimately, additional cell biology and genomic sequencing is needed for these poorly known organisms. Because there is no synapomorphy for the Fungi regardless of whether the Opisthosporidia are included (Richards et al. 2017), determining the constituency of the Fungi will rely on which traits are deemed most relevant (presence of chitinous cell wall or osmoheterotrophy) as both traits have been convergently derived across the eukaryote tree. A similar debate is ongoing about the distinction between Rozellida (Cryptomycota) and Microsporidia (Bass et al. 2018) as some rozellids have a spore structure very similar to Microsporidia but a genomic content less reduced than core microsporidia, and more like other Fungi. The stance taken in this classification is to follow the majority opinion of the mycological community to include Opisthosporidia in the Fungi, noting that this consensus may change with the discovery of new taxa and improved phylogenies.

33 The placement of Aphelidea in the Opisthosporidia is unstable and may change.

34 This revision reflects numerous advances in the phylogeny of the diatoms over the last decade. Due to our poor taxon sampling outside of the Mediophyceae and pennate diatoms, and the known and anticipated diversity of all diatoms, many clades appear at a high classification level (and the higher level classification is rather flat). Nomenclature follows the botanical code (ICN). Some of the basal nodes will probably become better resolved in the future, and would permit additional subdivisions. The genera are provided as examples only, and are far from complete lists.

35 The ordering of the major taxa follows the arrangement of most SSU rRNA phylogenies and recent phylogenomic analyses (e.g. Gentekaki et al., 2014). The current classification contains some polyphyletic or paraphyletic taxa marked by (P) which require further investigations (morphologic and/or genetic). Families are arranged alphabetically, unless phylogeny places a family at a higher hierarchical rank.

36 A node including a probably monophyletic clade composed of Sainouridae and Thecofilosea is proposed, called Helkesida Cavalier‐Smith 2018 (R).

37 Ancestral condition probably a gliding heterotrophic cell with two cilia with divergent kinetosomes, and relatively rigid pellicle that helped to define a distinct ventral groove from which filose pseudopods extended, but without the dense extracellular theca of Thecofilosea or internal silica skeleton of Ebriacea and Phaeodaria. Includes Cercozoa with often imbricate silica scales and their closest nonscaly relatives.

38 Centrohelida Kühn was introduced to include both centrohelids and gymnosphaerids; Febvre‐Chavalier and Febvre (1984) were the first who recognized and named this taxon.

39 One more genus name—*Heteroraphidiophrys*, mentioned in Mikrjukov (2002) was never formally introduced and needs to be avoided; the organisms designated needs to be re‐isolated, carefully studied and provided with formal description.

40 Genera *Pseudoraphidiophrys, Pseudoraphidocystis, Pterocystis, Raineriophrys* altogether obviously unify a group of closely related organisms which need a careful taxonomic revision involving morphological and molecular data. The genus name *Echinocystis* sometimes used instead of *Raineriophrys* is multiply preoccupied and should be avoided.

41 For more detailed description of the location and nomenclature of SSU rRNA gene expansions in centrohelids see Shishkin et al., 2018.

42 Separation of this genus from *Heterophrys* requires further justification.

43 Cryptista has been used since 1989 with a variety of definitions, and varying in composition and rank. Unless the authority and date is specified, it is uncertain which meaning, or rank, of Cryptista is meant. However, the term has uses in phylogenetic trees and it has been somewhat adopted in the literature, although without precision over its meaning. For nomenclatural stability Cryptista is defined here according to its latest use in phylogenetic trees.

44 Presently, there is no phylogenetic taxonomy for phagotrophic euglenids as a whole. Traditional systems based on, for example, the presence or absence of conspicuous feeding apparatuses, are artificial. Some possibly monophyletic taxa have been proposed that would cover restricted subsets of the phagotrophic euglenids: for example, the Ploeotiida, including *Ploeotia*,* Entosiphon*,* Lentomonas*, and presumably *Keelungia*; and the Petalomonadida, including *Petalomonas*,* Notosolenus* and *Calycimonas*, and presumably also taxa such as *Sphenomonas*. The limited taxon sampling for molecular sequence data is a significant impediment, especially as many traditional genera are probably polyphyletic.

## References

[jeu12691-bib-0001] Adl, M. S. , Leander, B. S. , Simpson, A. G. B. , Archibald, J. M. , Anderson, O. R. , Barta, J. R. , Bass, D. , Bowser, S. S. , Brugerolle, G. , Farmer, M. A. , Karpov, S. , Kolisko, M. , Lane, C. E. , Lodge, J. , Lynn, D. H. , Mann, D. G. , Meisterfeld, R. , Mendoza, L. , Moestrup, Ø. , Mozley‐Standridge, S. E. , Smirnov, A. V. & Spiegel, F. W. 2007 Diversity, nomenclature and taxonomy of protists. Syst. Biol., 56(4):684–689. 17661235 10.1080/10635150701494127

[jeu12691-bib-0002] Adl, M. S. , Simpson, A. G. B. , Farmer, M. A. , Andersen, R. A. , Anderson, O. R. , Barta, J. R. , Bowser, S. S. , Brugerolle, G. , Fensome, R. A. , Fredericq, S. , James, T. Y. , Karpov, S. , Kugrens, P. , Krug, J. , Lane, C. E. , Lewis, L. A. , Lodge, J. , Lynn, D. H. , Mann, D. G. , McCourt, R. M. , Mendoza, L. , Moestrup, Ø. , Mozley‐Standridge, S. E. , Nerad, T. A. , Shearer, C. A. , Smirnov, A. V. , Spiegel, F. W. & Taylor, F. J. R. 2005 The new classification of eukaryotes with emphasis on the taxonomy of protists. J. Eukaryot. Microbiol., 52(5):399–451. 16248873 10.1111/j.1550-7408.2005.00053.x

[jeu12691-bib-0003] Adl, S. M. , Simpson, A. G. , Lane, C. E. , Lukeš, J. , Bass, D. , Bowser, S. S. , Brown, M. W. , Burki, F. , Dunthorn, M. , Hampl, V. , Heiss, A. , Hoppenrath, M. , Lara, E. , Le Gall, L. , Lynn, D. H. , McManus, H. , Mitchell, E. A. D. , Mozley‐Stanridge, S. E. , Parfrey, L. W. , Pawlowski, J. , Rueckert, S. , Shadwick, L. , Schoch, C. , Smirnov, A. & Spiegel, F. W. 2012 The revised classification of eukaryotes. J. Eukaryot. Microbiol., 59:429–493. 23020233 10.1111/j.1550-7408.2012.00644.x PMC3483872

[jeu12691-bib-9001] Bass, D. , Czech, L. , Williams, B. A. P. , Berney, C. , Dunthorn, M. , Mahé, F. , Torruella, G. , Stentiford, G. D. & Williams, T. A. 2018 Clarifying the relationships between microsporidia and cryptomycota. J. Euk. Microbiol., 65(6):773–782. 29603494 10.1111/jeu.12519 PMC6282948

[jeu12691-bib-0004] Berney, C. , Ciuprina, A. , Bender, S. , Brodie, J. , Edgcomb, V. , Kim, E. , Rajan, J. , Wegener Parfrey, L. , Adl, S. , Audic, S. , Bass, D. , Caron, D. A. , Cochrane, G. , Czech, L. , Dunthorn, M. , Geisen, S. , Oliver Glöckner, F. , Mahé, F. , Quast, C. , Kaye, J. Z. , Simpson, A. G. B. , Stamatakis, A. , del Campo, J. , Yilmaz, P. & de Vargas, C. 2017 UniEuk: time to speak a common language in protistology! J. Eukaryot. Microbiol., 64:407–411. 10.1111/jeu.12414 28337822 PMC5435949

[jeu12691-bib-0005] Berney, C. , Geisen, S. , Van Wichelen, J. , Nitsche, F. , Vanormelingen, P. , Bonkowski, M. & Bass, D. 2015 Expansion of the “reticulosphere”: diversity of novel branching and network‐forming amoebae helps to define Variosea (Amoebozoa). Protist, 166:271–295. 25965302 10.1016/j.protis.2015.04.001

[jeu12691-bib-0006] Blandenier, Q. , Seppey, C. V. W. , Singer, D. , Vlimant, M. , Simon, A. , Duckert, C. & Lara, E. 2017 *Mycamoeba gemmipara* nov. gen., nov. sp., the first cultured member of the environmental Dermamoebidae Clade LKM74 and its unusual life cycle. J. Eukaryot. Microbiol., 64:257–265. 27543384 10.1111/jeu.12357

[jeu12691-bib-0007] Brown, M. W. , Heiss, A. A. , Kamikawa, R. , Inagaki, Y. , Yabuki, A. , Tice, A. K. , Shiratori, T. , Ishida, K. , Hashimoto, T. , Simpson, A. G. B. & Roger, A. J. 2018 Phylogenomics places orphan protistan lineages in a novel eukaryotic super‐group. Genome Biol. Evol., 10:427–433. 29360967 10.1093/gbe/evy014 PMC5793813

[jeu12691-bib-0008] del Campo, J. , Kolisko, M. , Boscaro, V. , Santoferrara, L. F. , Massana, R. , Guillou, L. , Simpson, A. G. B. , Berney, C. , de Vargas, C. , Brown, M. , Keeling, P. & Parfrey, L. W. 2018 EukRef: phylogenetic curation of ribosomal RNA to enhance understanding of eukaryotic diversity and distribution. PLOS Biol. 2018;16:e2005849. 30222734 10.1371/journal.pbio.2005849 PMC6160240

[jeu12691-bib-9000] Cantino, P. D. 1998 Binomials, hyphenated uninomials, and phylogenetic nomenclature. Taxon 47:425–429.

[jeu12691-bib-0009] Cavalier‐Smith, T. , Chao, E. E. & Lewis, R. 2016 187‐Gene phylogeny of protozoan phylum Amoebozoa reveals a new class (Cutosea) of deep‐branching, ultrastructurally unique, enveloped marine Lobosa and clarifies amoeba evolution. Mol. Phylogenet. Evol., 99:275–296. 27001604 10.1016/j.ympev.2016.03.023

[jeu12691-bib-0010] De Vargas, C. , Audic, S. , Henry, N. , Decelle, J. , Mahé, F. , Logares, R. , Lara, E. , Berney, Ć. , Le Bescot, N. , Probert, I. , Carmichael, M. , Poulain, J. , Romac, S. , Colin, S. , Aury, J.‐M. , Bittner, L. , Chaffron, S. , Dunthorn, M. , Engelen, S. , Flegontova, O. , Guidi, L. , Horák, A. , Jaillon, O. , Lima‐Mendez, G. , Lukeš, J. , Malviya, S. , Morard, R. , Mulot, M. , Scalco, E. , Siano, R. , Vincent, F. , Zingone, A. , Dimier, C. , Picheral, M. , Searson, S. , Kandels‐Lewis, S. , Acinas, S. G. , Bork, P. , Bowler, C. , Gorsky, G. , Grimsley, N. , Hingamp, P. , Iudicone, D. , Not, F. , Ogata, H. , Pesant, S. , Raes, J. , Sieracki, M. E. , Speich, S. , Stemmann, L. , Sunagawa, S. , Weissenbach, J. , Wincker, P. , Karsenti, E. , Boss, E. , Follows, M. , Karp‐Boss, L. , Krzic, U. , Reynaud, E. G. , Sardet, C. , Sullivan, M. B. & Velayoudon, D. 2015 Eukaryotic plankton diversity in the sunlit ocean. Science, 348(6237):1261605 10.1126/science.1261605 25999516

[jeu12691-bib-0011] Dutrochet, R. J. H. 1824 Recherches anatomiques et physiologiques sur la structure intime des animaux et des végétaux. Baillière, Paris. PMC5754632 30329773

[jeu12691-bib-0012] Ehrenberg, C. G. 1838 Über das Massenverhältniss der jetzt lebenden Kiesel‐Infusorien und über ein neues Infusorien‐Conglomerat als Polierschiefer von Jastraba in Ungarn. Königlich Akademie der Wissenschaften zu Berlin, Abhandlungen Vol. **1**: p. 109–135, pl.1‐2.

[jeu12691-bib-0013] Fenchel, T. 1986 Protozoan filter feeding. Prog. Protistol., 1:65–114. Eds. Corliss, J.O. & Patterson, D.J. Biopress Ltd.

[jeu12691-bib-0014] Grassi, B. 1881 Intorno ai chetognati. Reale Ist. Lombardo Sci. Lett., 2:185–224.

[jeu12691-bib-0015] Hibberd, D. J. 1983 Ultrastructure of the colonial colouless flagellates *Phalansterium digitatum* Stein (Phalansteriida ord. nov.) and *Spongomonas uvella* (Spongomonadida ord. nov.). Protistologica, 19:523–535.

[jeu12691-bib-3002] Karpov, S. A. , Torruella, G. , Moreira, D. , Mamkaeva, M. A. & López‐García, P. 2017a Molec. Phylogeny of *Paraphelidium letcheri* sp. nov. (Aphelida, Opisthosporidia) J. Euk. Micro. 64(5): 573–578. 10.1111/jeu.12389 PMC5650051 27987526

[jeu12691-bib-30021] Karpov, S. A. , Tcvetkova, V. S. , Mamkaeva, M. A. , Torruella, G. , Timpano, H. , Moreira, D. , Mamanazarova, K. S. & López‐García, P. 2017b Morphological and genetic diversity of opisthosporidia: New aphelid *Paraphelidium tribonemae* gen. et sp. nov. J. Euk. Micro. 64(2):204–212. 10.1111/jeu.12352 PMC5551964 27487286

[jeu12691-bib-0016] Kang, S. , Tice, A. K. , Spiegel, F. W. , Silberman, J. D. , Pánek, T. , Čepička, I. , Kostka, M. , Kosakyan, A. , Alcântara, D. M. , Roger, A. J. , Shadwick, L. L. , Smirnov, A. , Kudryavstev, A. , Lahr, D. J. & Brown, M. W. 2017 Between a pod and a hard test: the deep evolution of amoebae. Mol. Biol. Evol., 34:2258–2270. 10.1093/molbev/msx162 28505375 PMC5850466

[jeu12691-bib-0017] Kar, D. 1990 A pirhemocyton‐like parasite of the blenny, *Blennius pholis* L. (Teleostei: Blennidae) and its relationship to *immune‐plasma* Neumann 1909. Int. J. Parasitol., 3:235e241. 10.1016/0020-7519(73)90028-3 4706573

[jeu12691-bib-0018] Lahr, D. J. G. , Grant, J. R. & Katz, L. A. 2013 Multigene phylogenetic reconstruction of the Tubulinea (Amoebozoa) corroborates four of the six major lineages, while additionally revealing that shell composition does not predict phylogeny in the Arcellinida. Protist, 164:323–339. 23499265 10.1016/j.protis.2013.02.003

[jeu12691-bib-0019] Lahr, D. J. G. , Grant, J. , Nguyen, T. , Lin, J. H. & Katz, L. A. 2011 Comprehensive phylogenetic reconstruction of Amoebozoa based on concatenated analyses of SSU‐rDNA and actin genes. PLoS ONE, 6:e22780 10.1371/journal.pone.0022780 21829512 PMC3145751

[jeu12691-bib-0020] Lahr, J. G. , Lara, E. & Mitchell, E. A. D. 2012 Time to regulate microbial eukaryote nomenclature. Biol. J. Linn. Soc., 107(3):469–476. 10.1111/j.1095-8312.2012.01962.x.

[jeu12691-bib-0021] Lara, E. , Heger, T. J. , Ekelund, F. , Lamentowicz, M. & Mitchell, E. A. D. 2008 Ribosomal RNA genes challenge the monophyly of the Hyalospheniidae (Amoebozoa: Arcellinida). Protist, 159:165–176. 18023614 10.1016/j.protis.2007.09.003

[jeu12691-bib-3004] Letcher, P. M. , Longcore, J. E. , Quandt, C. A. , Leite, D. D. S. , James, T. Y. & Powell, M. J. 2017 Morphological, molecular, and ultrastructural characterization of *Rozella rhizoclosmatii*, a new species in Cryptomycota. Fungal Biol., 121(1):1–10. 28007212 10.1016/j.funbio.2016.08.008

[jeu12691-bib-3005] López‐Escardó, D. , López‐García, P. , Moreira, D. , Ruiz‐Trillo, I. & Torruella, G. 2018 *Parvularia atlantis* gen. et sp. nov., a Nucleariid Filose Amoeba (Holomycota, Opisthokonta). J. of Euk. Microbiol. 65(2): 170–179. 28741861 10.1111/jeu.12450 PMC5708529

[jeu12691-bib-0022] Meisterfeld, R. 2002 Order Arcellinida Kent 1880 *In:* Lee J. J., Leedale G. F. & Bradbury P. (ed.), An Illustrated Guide to the Protozoa, 2nd ed Society of Protozoologists, Lawrence, KS (Year 2000). p. 827–860.

[jeu12691-bib-0023] Mikryukov, K. A. & Mylnikov, A. P. 1998 The fine structure of a carnivorous multiflagellar protist, *Multicilia marina* Cienkowski, 1881 (Flagellata incertae sedis). Eur. J. Protistol., 34:391–401.

[jeu12691-bib-0024] Nguyen, N. H. , Song, Z. , Bates, S. T. , Branco, S. , Tedersoo, L. , Menke, J. , Schilling, J. S. & Kennedy, P. G. 2016 FUNGuild: an open annotation tool for parsing fungal community datasets by ecological guild. Fungal Ecol., 20:241–248.

[jeu12691-bib-0025] Pánek, T. , Zadrobílková, E. , Walker, G. , Brown, M. W. , Gentekaki, E. , Hroudrová, M. , Kang, S. , Roger, A. J. , Tice, A. K. , Vlček, Č. & Čepička, I. 2016 First multigene analysis of Archamoebae (Amoebozoa: Conosa) robustly reveals its phylogeny and shows that Entamoebidae represents a deep lineage of the group. Mol. Phylogenet. Evol., 98:41–51. 26826602 10.1016/j.ympev.2016.01.011

[jeu12691-bib-0026] Pawlowski, J. , Audic, S. , Adl, S. , Bass, D. , Belbahri, L. , Berney, C. , Bowser, S. S. , Cepicka, I. , Decelle, J. , Dunthorn, M. , Fiore‐Donno, A. M. , Gile, G. H. , Holzmann, M. , Jahn, R. , Jirků, M. , Keeling, P. J. , Kostka, M. , Kudryavtsev, A. , Lara, E. , Lukeš, J. , Mann, D. G. , Mitchell, E. A. D. , Nitsche, F. , Romeralo, M. , Saunders, G. W. , Simpson, A. G. B. , Smirnov, A. V. , Spouge, J. L. , Stern, R. F. , Stoeck, T. , Zimmermann, J. & Schindel, D. 2012 CBOL protist working group: barcoding eukaryotic richness beyond the animal, plant, and fungal kingdoms. PLoS Biol., 10(11):e1001419. 23139639 10.1371/journal.pbio.1001419 PMC3491025

[jeu12691-bib-3006] Powell, M. J. , Letcher, P. M. & James, T. Y. 2017 Ultrastructural characterization of the host–parasite interface between *Allomyces anomalus* (Blastocladiomycota) and *Rozella allomycis* (Cryptomycota). Fungal Biol. 121(6–7): 561–572. 28606351 10.1016/j.funbio.2017.03.002

[jeu12691-bib-8008] Pleijel, F. , and G. W. Rouse . 2003 Ceci n'est pas une pipe: Clades and phylogenetic nomenclature. J. Zool. Syst. Evol. Res. 41:162–174.

[jeu12691-bib-8000] Quandt, C. A. , Beaudet, D. , Corsaro, D. , Walochnik, J. , Michel, R. , Corradi, N. , & James, T. Y. 2017 The genome of an intranuclear parasite, Paramicrosporidium saccamoebae, reveals alternative adaptations to obligate intracellular parasitism. Elife, 6. 10.7554/eLife.29594 PMC5701793 29171834

[jeu12691-bib-3007] Richards, T. A. , Leonard, G. & Wideman, J. G. 2017 What defines the “Kingdom” Fungi? Microbiol. Spectrum 01 Jun 2017, 5(3) DOI: 10.1128/microbiolspec.FUNK-0044-2017 PMC11687502 28643626

[jeu12691-bib-0027] Ruggiero, M. A. , Dennis, P. G. , Orrell, T. M. , Bailly, N. , Bourgoin, T. , Brusca, R. C. , Cavalier‐Smith, T. , Guiry, M. D. & Kirk, P. 2015 A higher level classification of all living organisms. PLoS ONE, 10(6):e0130114 10.1371/journal.pone.0119248. 26068874 PMC5159126

[jeu12691-bib-0028] Saint‐Exupéry, A. 1943 Le petit prince. Reynal & Hitchcock, New York, NY.

[jeu12691-bib-0030] Schaudinn, F. 1899 Untersuchungen uber den Generations wechsel von *Trichosphaerium sieboldi*. Schn. Abh. K. Preuss. Akad. Wiss., Berlin, (Suppl):1–93.

[jeu12691-bib-0031] Schierwater, B. & DeSalle, R. 2018 Placozoa. Curr. Biol., 28:R97–R98. 29408263 10.1016/j.cub.2017.11.042

[jeu12691-bib-0032] Schleiden, M. J. 1838 Beiträge zur phytogenesis. Veit et Comp, Berlin.

[jeu12691-bib-0033] Schwann, T. 1839 Mikroskopische Untersuchungen über die Uebereinstimmung in der Struktur und dem Wachsthum der Thiere und Pflanzen, Berlin. 21516882

[jeu12691-bib-0034] Shadwick, J. D. L. , Silberman, J. D. & Spiegel, F. W. 2017 Variation in the SSUrDNA of the genus *Protostelium* leads to a new phylogenetic understanding of the genus and of the species concept for *Protostelium mycophaga* (Protosteliida, Amoebozoa). J. Eukaryot. Microbiol., 65:331–344. 10.1111/jeu.12476 29044743

[jeu12691-bib-0035] Sheikh, S. , MatsThulin Cavender, J. C. , Escalante, R. , Kawakami, S. I. , Lado, C. , Landolt, J. C. , Nanjundiah, V. , Queller, D. C. , Strassmann, J. E. , Spiegel, F. W. , Stephenson, S. L. , Vadell, S. M. & Baldauf, S. L. 2018 A new classification of the dictyostelids. Protist, 169:1–28. 29367151 10.1016/j.protis.2017.11.001

[jeu12691-bib-0036] Sibbald, S. J. , Cenci, U. , Colp, M. , Eglit, Y. , O'Kelly, C. J. & Archibald, J. M. 2017 Diversity and evolution of *Paramoeba* spp. and their kinetoplastid endosymbionts. J. Eukaryot. Microbiol., 64:598–607. 10.1111/jeu.12394 28150358

[jeu12691-bib-0037] Simpson, A. G. B. & Roger, A. J. 2004 The real “kingdoms” of eukaryotes. Curr. Biol., 14:R693–R896. 10.1016/j.cub.2004.08.038. 15341755

[jeu12691-bib-0038] Smirnov, A. 2008 Amoebas, Lobose *In:* Schaechter M. (ed.), Encyclopedia of Microbiology. Elsevier Science Publishers, Oxford p. 558–577.

[jeu12691-bib-0039] Spiegel, F. W. , Shadwick, L. L. , Ndiritu, G. G. , Brown, M. W. , Aguilar, M. & Shadwick, J. D. L. 2017 Protosteloid Amoeboazoa (Protosteliids, Protosporangiida, Cavostellida, Schizoplasmodiida, Fractoviteliida, and sporcarpic members of Vanellida, Centramoebida, and Pellitida) *In:* Archibald J. M., Simpson A. G. B. & Slamovits C. (ed.), Handbook of the Protists (Second Edition of the Handbook of Protoctista by Margulis et al.). Springer Reference Works (e‐book). 10.1007/978-3-319-32669-6_12-1

[jeu12691-bib-0040] Srivastava, M. , Begovic, E. , Chapman, J. , Putnam, N. H. , Hellsten, U. , Kawashima, T. , Kuo, A. , Mitros, T. , Salamov, A. , Carpenter, M. L. , Signorovitch, A. Y. , Moreno, M. A. , Kamm, K. , Grimwood, J. , Schmutz, J. , Shapiro, H. , Grigoriev, I. V. , Buss, L. W. , Schierwater, B. , Dellaporta, S. L. & Rokhsar, D. S. 2008 The *Trichoplax* genome and the nature of placozoans. Nature, 454(7207):955–960. 10.1038/nature07191. 18719581

[jeu12691-bib-0041] Tekle, Y. I. , Anderson, O. R. , Katz, L. A. , Maurer‐Alcala, X. X. , Romero, M. A. & Molestina, R. 2016 Phylogenomics of ‘Discosea’: A new molecular phylogenetic perspective on Amoebozoa with flat body forms. Mol. Phylogenet. Evol., 99:144–154. 27015898 10.1016/j.ympev.2016.03.029

[jeu12691-bib-0042] Tekle, Y. I. & Wood, F. C. 2017 Longamoebia is not monophyletic: phylogenomic and cytoskeleton analyses provide novel and well‐resolved relationships of amoebozoan subclades. Mol. Phylogenet. Evol., 114:249–260. 10.1016/j.ympev.2017.06.019 28669813

[jeu12691-bib-0043] Tice, A. K. , Shadwick, L. L. , Fiore‐Donno, A. M. , Geisen, S. , Kang, S. , Schuler, G. A. , Spiegel, F. W. , Wilkinson, K. A. , Bonkowski, M. , Dumack, K. , Lahr, D. J. G. , Voelcker, E. , Clauβ, S. , Zhang, J. & Brown, M. W. 2016 Expansion of the molecular and morphological diversity of Acanthamoebidae (Centramoebida, Amoebozoa) and identification of a novel life cycle type within the group. Biol. Direct, 11:69 10.1186/s13062-016-0171-0. 28031045 PMC5192571

[jeu12691-bib-0501] Torruella, G. , Grau‐Bové, X. , Moreira, D. , Karpov, S. A. , & Burns, J. A. , Sebé‐Pedrós, A. , Völcker, E. & López‐García, P. 2019 Global transcriptome analysis of the aphelid *Paraphelidium tribonemae* supports the phagotrophic origin of fungi. Communications Biology. In press. 10.1038/s42003-018-0235-z PMC6299283 30588510

[jeu12691-bib-0044] Van Tieghem, M. P. 1884 *Cœnonia*, genre nouveau de Myxomycètes a plasmode agrégé. Bull. Soc. Bot. France, 31:303–306. 10.1080/00378941.1884.10828252.

[jeu12691-bib-0045] Van Vichelen, J. , D'Hondt, S. , Claeys, M. , Vyverman, W. , Berney, C. , Bass, D. & Vanormelingen, P. 2016 A hotspot of amoebae diversity: 8 new naked amoebae associated with the planktonic bloom‐forming cyanobacterium *Microcystis* . Acta Protozool., 55:61–87.

